# Genome-wide analysis of circular RNAs in prenatal and postnatal muscle of sheep

**DOI:** 10.18632/oncotarget.21835

**Published:** 2017-10-12

**Authors:** Cunyuan Li, Xiaoyue Li, Yang Yao, Qiman Ma, Wei Ni, Xiangyu Zhang, Yang Cao, Wureli Hazi, Dawei Wang, Renzhe Quan, Xiaoxu Hou, Zhijin Liu, Qianqian Zhan, Li Liu, Mengdan Zhang, Shuting Yu, Shengwei Hu

**Affiliations:** ^1^ College of Life Sciences, Shihezi University, Shihezi, Xinjiang, 832003, China; ^2^ College of Animal Science and Technology, Shihezi University, Shihezi, Xinjiang, 832003, China

**Keywords:** sheep, circRNAs, muscle, expression profiles, RNA-seq

## Abstract

Circular RNAs (circRNAs), a type of non-coding RNA with circular structure, were generated by back splicing and widely expressed in animals and plants. Recent studies have shown that circRNAs extensively participate in cell proliferation, cell differentiation, cell autophagy and other biological processes. However, the role and expression of circRNAs in the development and growth of muscle have not been studied in sheep. In our study, we first used RNA-seq to study the circRNAs in prenatal and postnatal longissimus dorsi muscle of sheep. A total of 6113 circRNAs were detected from the RNA-seq data. Several circRNAs were identified using reverse transcription PCR, DNA sequencing and RNase R digestion experiments. The expression levels of several circRNAs in prenatal and postnatal muscle were confirmed by Real-Time RT-PCR. The gene ontology (GO) and KEGG enrichment analysis of the host gene of the circRNAs showed that these circRNAs were mainly involved in the growth and development of muscle related signaling pathways. These circRNAs might sponge microRNAs (miRNAs) in predicted circRNA-miRNA-mRNA networks. The circRNAs expression profiles in muscle provided an important reference for the study of circRNAs in sheep.

## INTRODUCTION

CircRNAs were firstly discovered in plant viruses and hepatitis δ virus [1, 2]. Although circRNA has been observed in eukaryotes a few decades ago, it was mostly considered assplicing errors at best and lacking biological function [3, 4]. With breakthroughs in high-throughput sequencing techniques [5], several recent studies have shown that there are many single-stranded exonic circRNAs present in mammals [6–8], and a large number of studies have shown that circRNAs are generated by head-to-tail splicing [9, 10]. There are several proposed models involved in the biogenesis of circRNAs, including spliceosome-dependent circulation path, intron-pairing-driven circularization path, lariat-driven circularization path, protein factors associated circulation path [11–14]. CircRNAs were stable, conserved, tissue- and stage-specific expression [7]. The biological functions of circRNAs are still being indicated, but they are shown to sponge miRNAs [15], regulate gene transcription [16], translate into proteins [14], interact with RNA binding proteins [17] and modulate the stability of mRNAs [16]. A growing number of circRNAs are aberrantly expressed in colorectal cancer [18], hepatocellular carcinoma [19], gastric cancer [20], and laryngeal squamous cell carcinoma [21] and have the potential to become new diagnostic or prognostic biomarkers.

The skeletal muscle has become an important material for studying the mechanism of specific cell differentiation and proliferation in mammal [22]. Classic developmental studies have understood the origin and development of skeletal muscle cells in embryo. It has been known for many years that vertebrate skeletal muscle derived from embryonic mesodermal precursor cells [23]. The medial and lateral domains of somites can produce different muscle groups [22–26]. Longissimus dorsi muscle as the largest part of the ridge of the spine, it is a prime cut of high motion relevance for fresh and cured meet production [27]. Recent studies on skeletal muscle of monkeys in a range of ages [28] and chicken embryo skeletal muscle [29] have shown that circRNAs may affect muscle growth. Previous studies have shown that miRNAs such as miR-133, miR-206 and miR-1 expressed specifically in muscle [30], and long non-coding RNA (LINCMD1) can act as a sponge of miR-133 to regulate muscle development [31]. Recent studies have shown that circRNAs can also act as sponges for adsorbing miRNAs [32].

Sheep has been used as a powerful model organism for human disease studies and is an important animal in agriculture because of its utility in meat production. However, until now the expression and function of circRNAs in the muscle of sheep is unclear. In our study, we first used RNA-seq to study the circRNAs of prenatal and postnatal muscle in sheep. The circRNAs expression profiles of the longissimus dorsi muscle was given by our study, which will facilitate better understanding roles of circRNAs in development and growth of muscle.

## RESULTS

### Deep sequencing of circRNAs in sheep muscle

**Figure 1:**
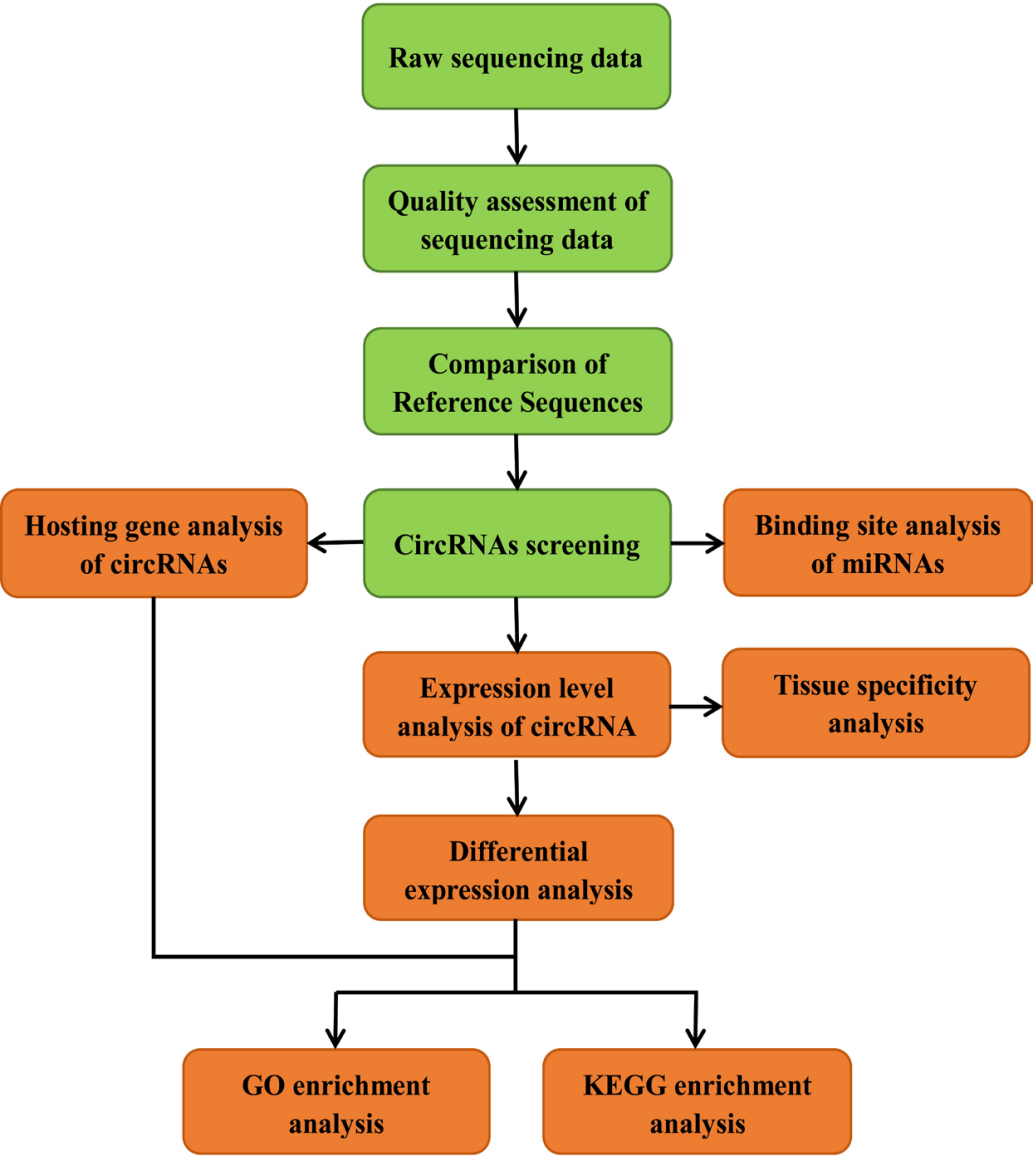
Pipeline for circRNAs identification

**Figure 2:**
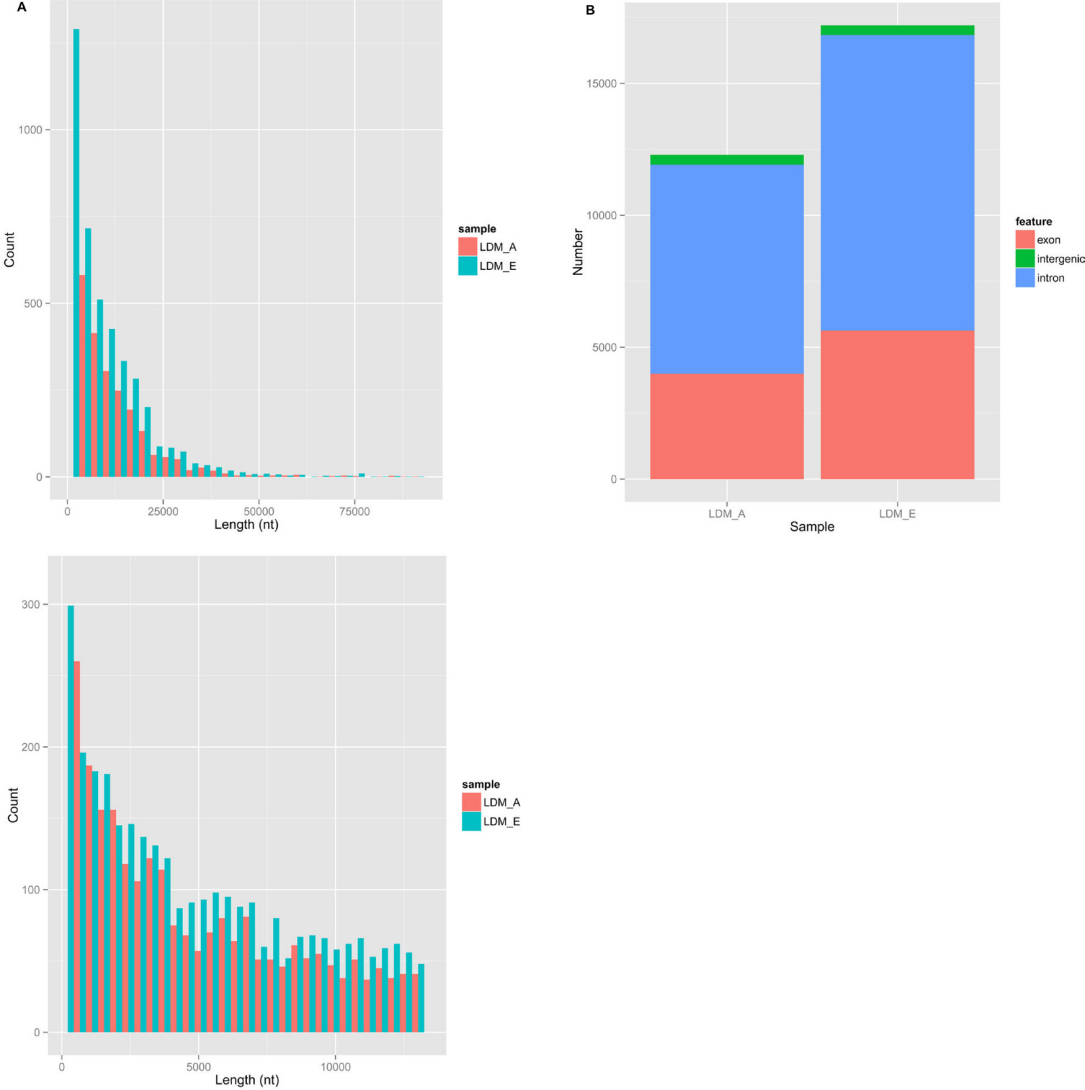
The information of circRNAs in sheep muscle by deep sequencing (**A**) The length of circRNAs in sheep muscle. Light red on behalf of LDM_A and light blue on behalf of LDM_E. (**B**) The number of introns, exons and intergenic of circRNAs in sheep muscle.

In order to understand the identity, abundance and difference of circRNAs in the muscle of embryonic and adult sheep, we performed paired-end ribominus RNA sequencing (RNA-seq) according to the flow chart (Figure 1), and used the FindCirc calculation pipeline to detect the circRNAs [7]. Through the RNA-seq of the embryo longissimus dorsi muscle (LDM_E) and adult longissimus dorsi muscle (LDM_A), and we obtained 15.91GB and 13.94GB clear data from the results respectively. After processing, a total of 6113 circRNAs were identified from these data, and the length of the circRNAs were mainly focused below 13000 nt (Figure 2A). These circRNAs were distributed over 26 autosomes and X chromosomes, and these circRNAs consisted mainly of introns and exons, and only a small fraction are composed of intergenic sequences (Figure 2B). The annotation, chromosomal location, hosting mRNA and so on of all circRNAs are shown in Supplementary File 1.

### Validation of sheep circRNAs

**Figure 3:**
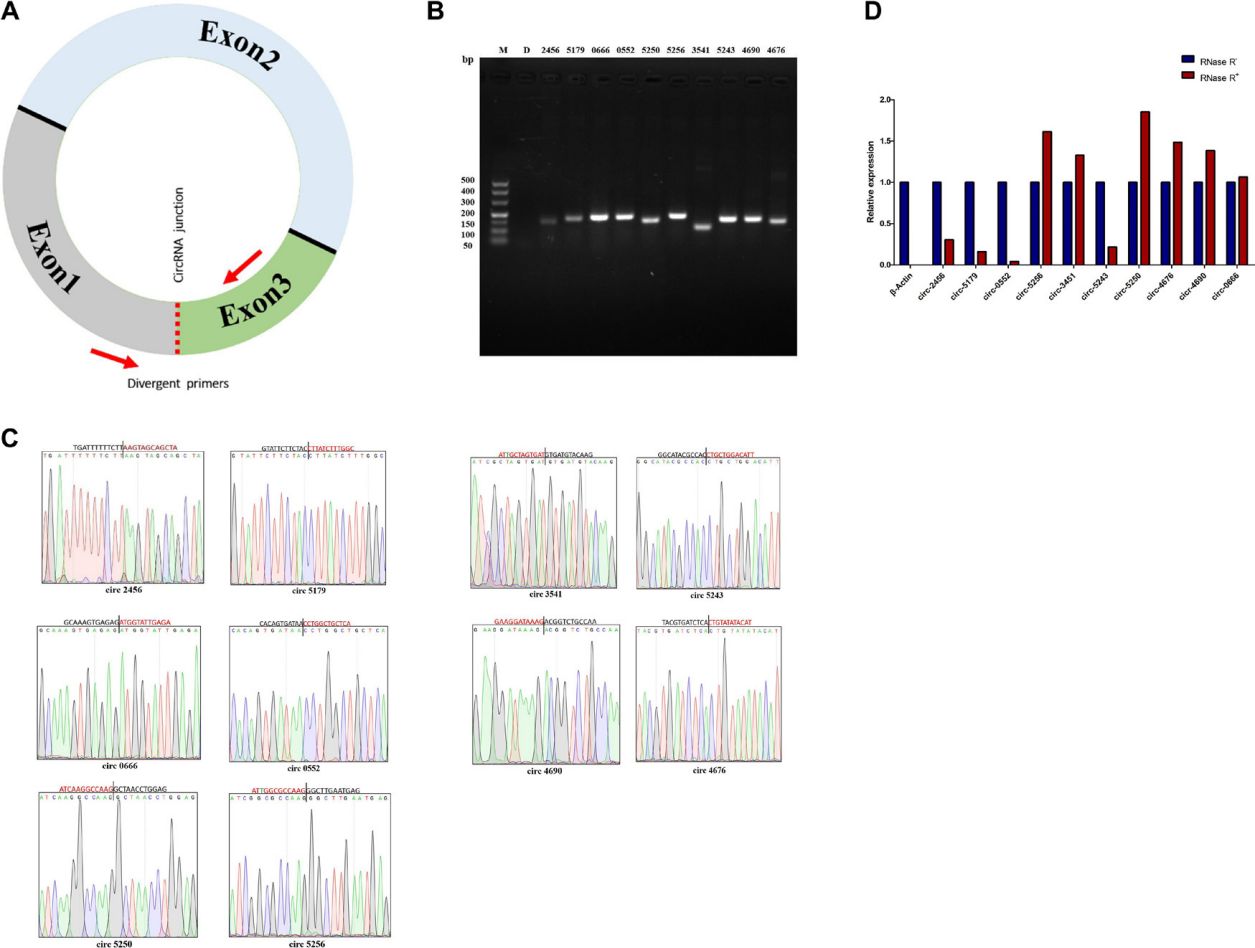
Verification of circRNAs data from RNA-sequencing (**A**) Divergent primers to amplify the circular junctions. Red arrows represent divergent primers. (**B**) RT-PCR amplification of circRNAs with divergent primers. PCR products of circRNA 0002456, circRNA 0005179, circRNA 0000666, circRNA 0000552, circRNA 0005250, circRNA 0005256, circRNA 0003541, circRNA 0005243, circRNA 0004690 and circRNA 0004676 were analysed by gel electrophoresis. (**C**) Head-to-tail junctions were confirmed by DNA sequencing. The unmatched bases in circRNA 0005256 and circRNA 0003541 are indicated in green. (**D**) Resistance test of circRNAs to RNase R digestion by Real-Time RT-PCR. β-Actin was used as a linear control. Error bars indicate ± SD.

We have further done some experimental validation in order to confirm the RNA-seq and expression of circRNAs in sheep. We randomly selected 10 circRNAs and designed divergent primers across the circRNAs junctions (Figure 3A). RT-PCR amplification and DNA sequencing techniques were used to confirm the circRNAs data of RNA-seq. The results of the RT-PCR amplification show a single band of the expected size (Figure 3B). DNA sequencing results confirm the head-to-tail circularization splicing of these circRNAs (Figure 3C). For circRNA 0003451 and circRNA 0005256, there is a single base difference between results of normal DNA and deep sequencing, which may be single nucleotide polymorphism (SNP) similar to some SNP in miRNAs [33]. Real-Time RT-PCR was further used for verifying circRNAs resistance to RNase R digestion experiments. All selected 10 circRNAs showed resistance to RNase R digestion. As expected, linear control of β-Actin was sensitive to the RNase R digestion, and it was completely digested (Figure 3D).

### Analysis and validation of differentially expressed circRNAs

**Figure 4:**
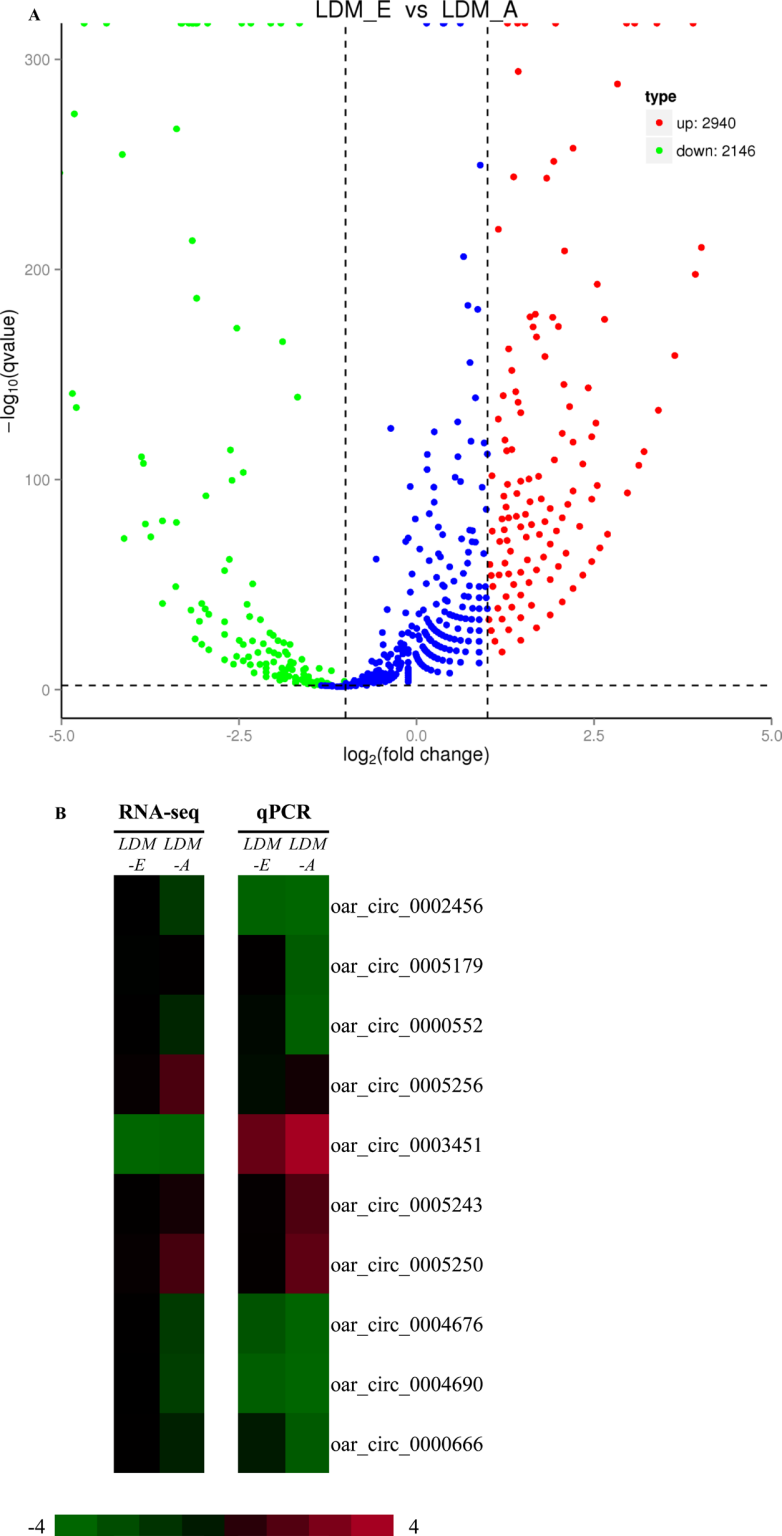
Analysis and validation of differentially expressed circRNAsin LDM_E and LDM_A group (**A**) The volcano plot analysis of all circRNAs between LDM_E and LDM_A group. The logarithm of the significant difference between the two samples was analyzed by log2 (fold change) as the abscissa, and the negative logarithm-log10 (*P*-value) of the *P* value was calculated as the ordinate (*P* < 0.05).Red dots indicate up-regulated genes; green dots indicate down-regulated genes. (**B**) Heapmap analysis of ten differentially expressed genes was performed with data from RNA-seq and Real-Time RT-PCR. Green represents low expression, and red represents high expression, *P < 0.05*.

A total of 5086 differentially expressed circRNAs were found between the embryo longissimus dorsi muscle (LDM_E) and adult longissimus dorsi muscle (LDM_A), including 2940 up-regulated and 2146 down-regulated circRNAs (Figure 4A). For verify the accuracy of these differentially expressed, we randomly selected 10 significant differentially expressed circRNAs using Real-Time RT-PCR (Figure 4B). The expression levels of 9 circRNAs detected by RT-PCR analysis (except for circRNA 0005179) were consistent with the RNA-seq data. CircRNA 0002456, circRNA 0000552, circRNA 0004676, circRNA 0004690 and circRNA 0000666 were higher in the embryonic group (LDM_E) than in adult group (LDM_A), and in contrast, circRNA 0005256, circRNA 0003451, circRNA 0005243 and circRNA 0005250 were highly expressed in the adult group (LDM_A) relative to the embryonic group (LDM_E). Real-Time RT-PCR results showed that the expression level of circRNA 0005179 was higher in the embryonic group (LDM_E) than in adult group (LDM_A), which was contrary with the RNA-seq data. The results show that there is a strong agreement between real-time RT-PCR and RNA-seq data, and it is shown that the identified circRNAs largely reflect the true differential expression *in vivo*.

### Enrichment of differentially expressed circRNAs

**Figure 5:**
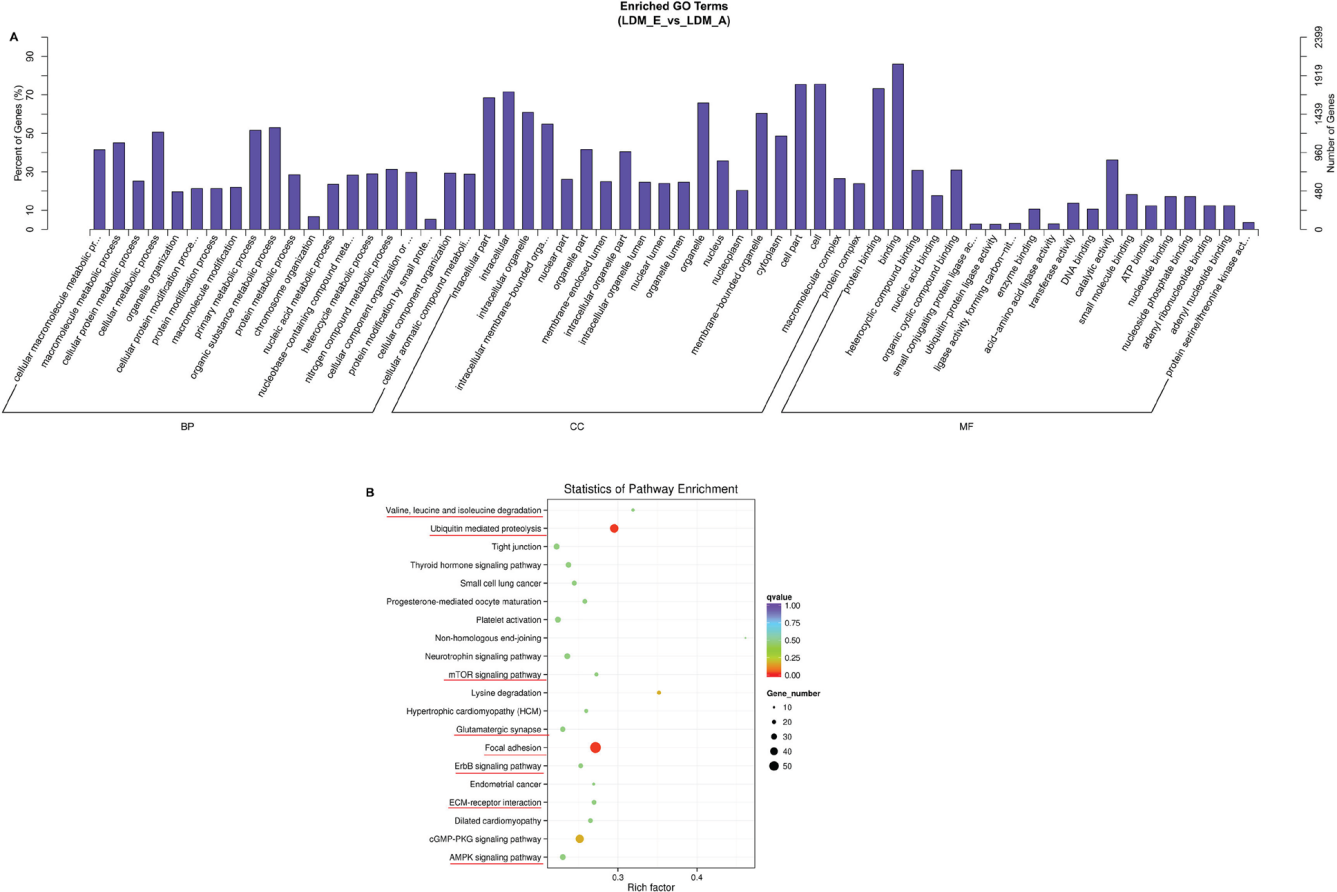
Annotations and enrichment of differentially expressed circRNAs (**A**) The GO analysis showed 897 significantly (LDM_E VS LDM_A) enriched terms (*P* < 0.05) in biological process, molecular function and cellular components category. (**B**) A total of 270 terms were enriched of differentially expressed circRNAs in LDM_E and LDM_A group by KEGG pathway analysis. The AMPK signaling pathway, ECM-receptor interactions, ErbB signaling pathway, focal adhesion, valine, leucine and isoleucine degradation, ubiquitin mediated proteolysis, mTOR signaling pathway and glutamatergic synapse, which were involved muscle growth and development, were labeled with red lines.

For the hosting genes of differentially expressed circRNAs, we performed enrichment analysis of them by GO and KEGG pathways. A total of 897 hosting genes of differentially expressed circRNAs were analyzed by GO analysis (*P* < 0.05, which were enriched separately in biological process, molecular function and cellular components category (Figure 5A). The top 20 genes from each GO category were related in metabolic process, position in cell or organelle, and function of nucleic binding, which suggests that some circRNAs were involved to the basic process of muscle growth and development. A total of 270 terms were enriched by KEGG pathway in which AMPK signaling pathway, ECM-receptor interactions, ErbB signaling pathway, focal adhesion, valine, leucine and isoleucine degradation, ubiquitin mediated proteolysis, mTOR signaling pathway and glutamatergic synapse were related to muscle differentiation and proliferation (Figure 5B) (Supplementary File 2). Analysis of these results indicate that circRNAs may play a vital role in muscle development and growth.

### Predicted functions of sheep circRNAs

Functional study of circRNAs are mainly focused on that circRNAs as an adsorbed miRNAs sponge that interacts with miRNAs [34–37]. Many studies have indicated that cicrRNAs affect gene expression at post-transcriptional levels through miRNA response elements (MRE) [38, 39]. In our study, a total of 286956 interactions were observed with the various miRNAs in the identified 6113 circRNAs, and the interaction between the circRNAs and the miRNAs were analyzed by using miRanda and psRobot (Supplementary File 3). It is noteworthy that several well-known miRNAs are closely related to growth and development of skeletal muscle, which are considered to be promising candidates for future research. For example, circRNAs (circRNA 0000385, circRNA 0000582 and circRNA 0001099 etc) have multiple conservative target sites for muscle development-related miRNAs (miR-143, miR-133 and miR-23etc, respectively). We further analyzed the number of miRNAs and MREs that interacted with these circRNAs (Table 1). This result suggests that it is likely that the process of the development and growth of the muscles were affected by the circRNAs.

**Table 1:** Potential miRNA (muscle development-related miRNAs) targets of circRNAs

CircRNA ID	Regulation (LDM_E VS LDM_A)	Gene symbol	Potential miRNA targets (No. MREs)
oar_circ_0000385	Up	RAD51C	let-7a (12); let-7b (6); let-7c (9); let-7d (3); let-7f (15); let-7g (8); let-7i (1); miR-103 (15); miR-107 (7); miR-10a (1); miR-10b (4); miR-1185-3p (2); miR-1185-5p (1); miR-1193-5p (6); miR-1197-3p (2); miR-1197-5p (2); miR-133 (3); miR-134-3p (4); miR-134-5p (1); miR-136 (4); miR-148a (4); miR-150 (4); miR-152 (5); miR-154b-3p (4); miR-16b (6); miR-181a (9); miR-191 (5); miR-194 (4); miR-199a-3p (5); miR-200a (5); miR-200b (4); miR-200c (7); miR-218a (6); miR-21 (1); miR-23a (5); miR-26a (6); miR-26b (3); miR-299-5p (4); miR-29a (5); miR-29b (5); miR-30a-3p (5); miR-30a-5p (8); miR-30b (7); miR-30c (4); miR-30d (10); miR-323a-3p (4); miR-323a-5p (1); miR-323b (5); miR-323c (1); miR-329a-3p (2); miR-329a-5p (1); miR-329b-3p (1); miR-329b-5p (1); miR-369-3p (3); miR-370-3p (3); miR-376a-3p (2); miR-376a-5p (4); miR-376b-3p (3); miR-376b-5p (1); miR-376d (1); miR-376e-5p (1); miR-380-5p (2); miR-381-3p (1); miR-382-5p (6); miR-3955-3p (1); miR-3955-5p (1); miR-3956-3p (1); miR-3958-5p (1); miR-3959-3p (1); miR-409-3p (4); miR-410-3p (2); miR-411b-3p (2); miR-412-5p (3); miR-485-3p (1); miR-485-5p (3); miR-487a-3p (3); miR-487b-5p (3); miR-493-3p (1); miR-493-5p (6); miR-494-3p (5); miR-495-3p (4); miR-495-5p (1); miR-496-3p (5); miR-496-5p (1); miR-539-3p (5); miR-539-5p (1); miR-541-5p (4); miR-543-3p (3); miR-544-3p (8); miR-654-5p (2); miR-665-3p (3); miR-668-3p (1); miR-758-3p (1);
oar_circ_0000582	Down	YME1L1	miR-103 (2); miR-107 (1); miR-10b (2); miR-1185-3p (1); miR-1197-3p (1); miR-125b (4); miR-134-5p (4); miR-143 (3); miR-150 (1); miR-154a-3p (1); miR-194 (2); miR-200b (2); miR-200c (1); miR-221 (5); miR-23a (4); miR-23b (2); miR-25 (1); miR-26a (8); miR-26b (4); miR-29a (1); miR-29b (2); miR-30a-3p (2); miR-30a-5p (1); miR-30b (6); miR-30c (5); miR-30d (1); miR-323a-3p (2); miR-323b (2); miR-323c (1); miR-329a-5p (3); miR-329b-5p (1); miR-362 (2); miR-374a (4); miR-376a-3p (1); miR-376a-5p (2); miR-376b-3p (1); miR-376b-5p (1); miR-376c-3p (4); miR-376c-5p (6); miR-376d (1); miR-376e-3p (1); miR-376e-5p (1); miR-377-3p (3); miR-382-5p (2); miR-3955-3p (1); miR-3959-5p (1); miR-409-3p (1); miR-412-5p (1); miR-487a-5p (2); miR-494-3p (3); miR-494-5p (2); miR-495-5p (3); miR-496-3p (2); miR-496-5p (2); miR-539-3p (4); miR-539-5p (1); miR-544-3p (3); miR-668-5p (1); miR-758-3p (2)
oar_circ_0001099	Down	C7	miR-134-3p (2); miR-23b (2); miR-377-3p (2); miR-323a-3p (1); miR-329a-3p (1); miR-409-3p (3); miR-493-3p (1); miR-106b (1); miR-106a (1); miR-22-3p (1); miR-23a (3); miR-148a (2); miR-26a (1); miR-380-5p (2); miR-200b (6); miR-376b-5p (1); miR-362 (1); miR-376e-3p (1); miR-221 (1); miR-27a (1); miR-409-5p (1); miR-26b (1); miR-654-3p (1); miR-376e-5p (1); miR-381-3p (2); miR-495-3p (3); miR-30b (1); miR-125b (1); miR-382-3p (2); miR-3956-3p (2); miR-379-5p (1); miR-1193-5p (1); miR-665-3p (2); miR-152 (2); miR-3955-3p (4); miR-376a-5p (1); miR-30c (1); miR-3957-3p (1); miR-30d (1); miR-17-5p (1); miR-30a-5p (1); miR-665-5p (1); miR-1185-3p (1); miR-411b-3p (1); miR-1185-5p (1); miR-376c-3p (1); miR-181a (2); miR-30a-3p (2); miR-376c-5p (1); miR-323c (2); miR-494-3p (2); miR-543-3p (1); miR-487a-5p (1); miR-329b-3p (3); miR-544-5p (1); miR-3959-3p (1); miR-323b (2); miR-544-3p (3); miR-323a-5p (2); miR-199a-3p (3); miR-370-3p (1); miR-410-5p (2); miR-134-5p (1); miR-200c (5); miR-377-5p (1); miR-99a (1); miR-143 (1)
oar_circ_0001413	Up	SEMA7A	miR-181a (1); miR-329a-3p (1); miR-133 (1); miR-329b-3p (1); miR-493-3p (2); miR-107 (1); miR-1185-3p (1); miR-125b (1); miR-103 (1); miR-362 (1); miR-668-5p (1); miR-134-5p (1); miR-665-3p (1); miR-218a (1)
oar_circ_0003451	Down	TTN	miR-410-5p (1); miR-154b-5p (1); miR-758-5p (1); miR-544-3p (1)
oar_circ_0005250	Down	MYH7	miR-134-3p (10); miR-493-3p (6); miR-539-3p (1); miR-376b-5p (1); miR-654-5p (1); miR-409-3p (2); miR-376e-5p (1); let-7d (2); miR-379-3p (1); let-7f (2); let-7g (2); let-7a (2); let-7b (2); let-7c (2); miR-1193-5p (4); miR-323c (1); let-7i (2); miR-3955-3p (3); miR-376a-5p (3); miR-22-3p (3); miR-412-5p (7); miR-3956-3p (3); miR-1197-3p (2); miR-758-5p (1); miR-541-3p (5); miR-3958-5p (5); miR-29b (1); miR-3959-5p (2); miR-370-3p (8); miR-154b-3p (1); miR-377-5p (2); miR-493-5p (2); miR-380-5p (3); miR-495-5p (2); miR-27a (3); miR-433-5p (2); miR-1193-3p (4); miR-152 (3); miR-150 (2); miR-382-5p (1); miR-1197-5p (5); miR-148a (3); miR-411a-3p (1); miR-376c-5p (1); miR-3959-3p (2); miR-323a-5p (1); miR-154b-5p (2); miR-3958-3p (1); miR-143 (2); miR-29a (1); miR-154a-5p (1); miR-23a (1); miR-668-3p (2); miR-543-5p (8); miR-362 (2); miR-433-3p (1); miR-16b (2); miR-380-3p (1); miR-125b (9); miR-495-3p (1); miR-382-3p (1); miR-106b (2); miR-487a-5p (1); miR-106a (2); miR-3957-3p (1); miR-411b-3p (1); miR-1185-5p (2); miR-181a (3); miR-200a (2); miR-200b (3); miR-200c (4); miR-299-3p (3); miR-544-3p (7); miR-30a-3p (2); miR-17-5p (2); miR-23b (1); miR-103 (2); miR-107 (2); miR-431 (1); miR-432 (10); miR-221 (1); miR-154a-3p (1); miR-654-3p (5); miR-3955-5p (2); miR-379-5p (1); miR-487b-5p (1); miR-412-3p (1); miR-136 (3); miR-133 (1); miR-411b-5p (1); miR-665-5p (1); miR-1185-3p (1); miR-10a (2); miR-10b (2); miR-758-3p (1); miR-3957-5p (2); miR-485-5p (5); miR-494-3p (2); miR-370-5p (4); miR-377-3p (2)

MREs: miRNA response elements.

### Bioinformatics analysis of circRNA-miRNA-mRNA networks

**Figure 6:**
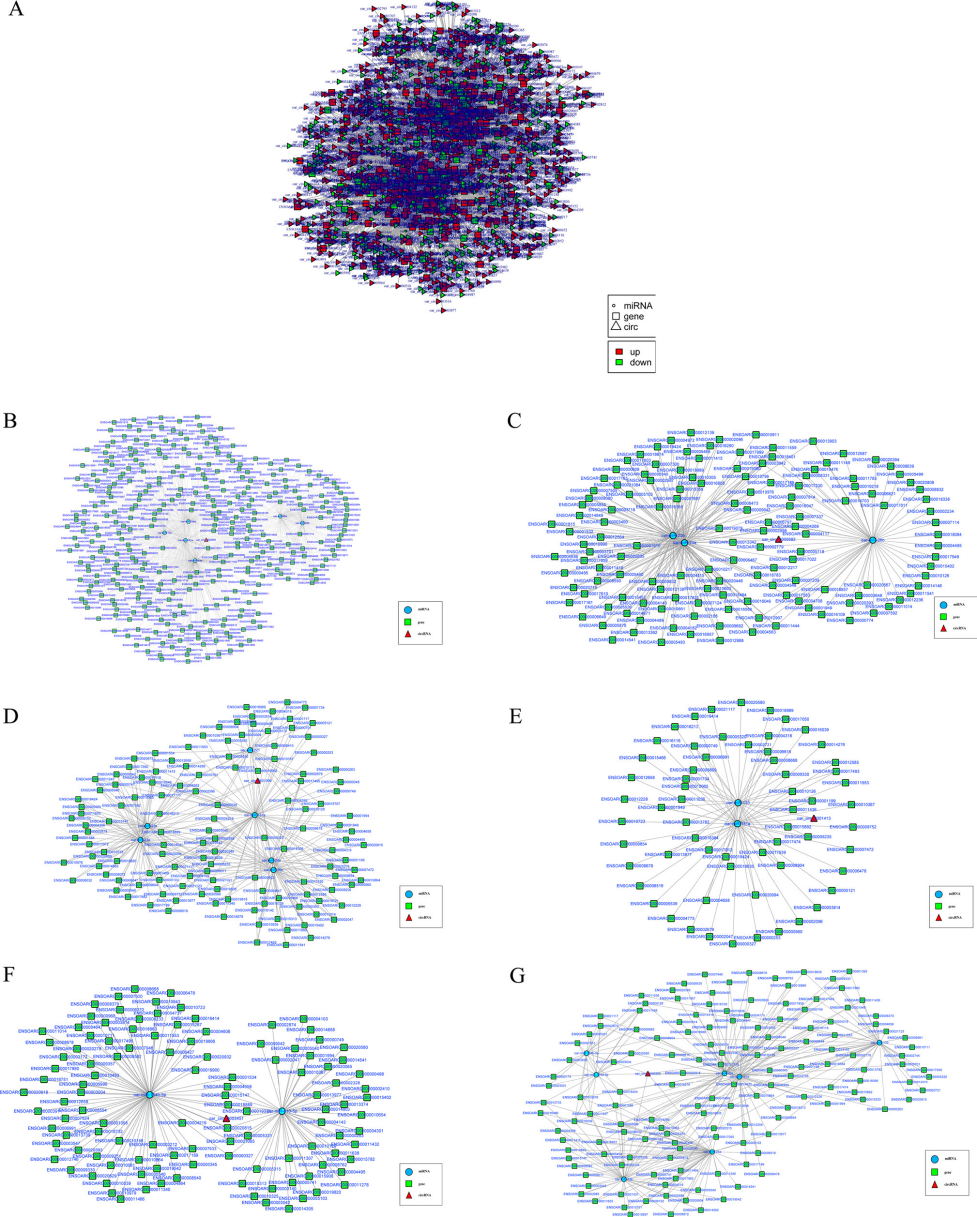
The circRNA-miRNA co-expression network (**A**) The circRNA-miRNA-mRNA interaction network between circRNA and its target gene. Ellipse nodes represent miRNAs, rectangle nodes represent genes and triangle nodes represent circRNAs. Red color and green color represents up and down regulation respectively. (B-G) The circRNA-miRNA-mRNA network with potentially effective circRNAs (circRNA 0000385 (**B**), circRNA 0000582 (**C**), circRNA 0001099 (**D**), circRNA 0001413 (**E**), circRNA 0003451 (**F**), circRNA 0005250 (**G**). Ellipse nodes represent miRNAs (blue), rectangle nodes represent genes (green) and triangle nodes represent circRNAs (red).

**Figure 7:**
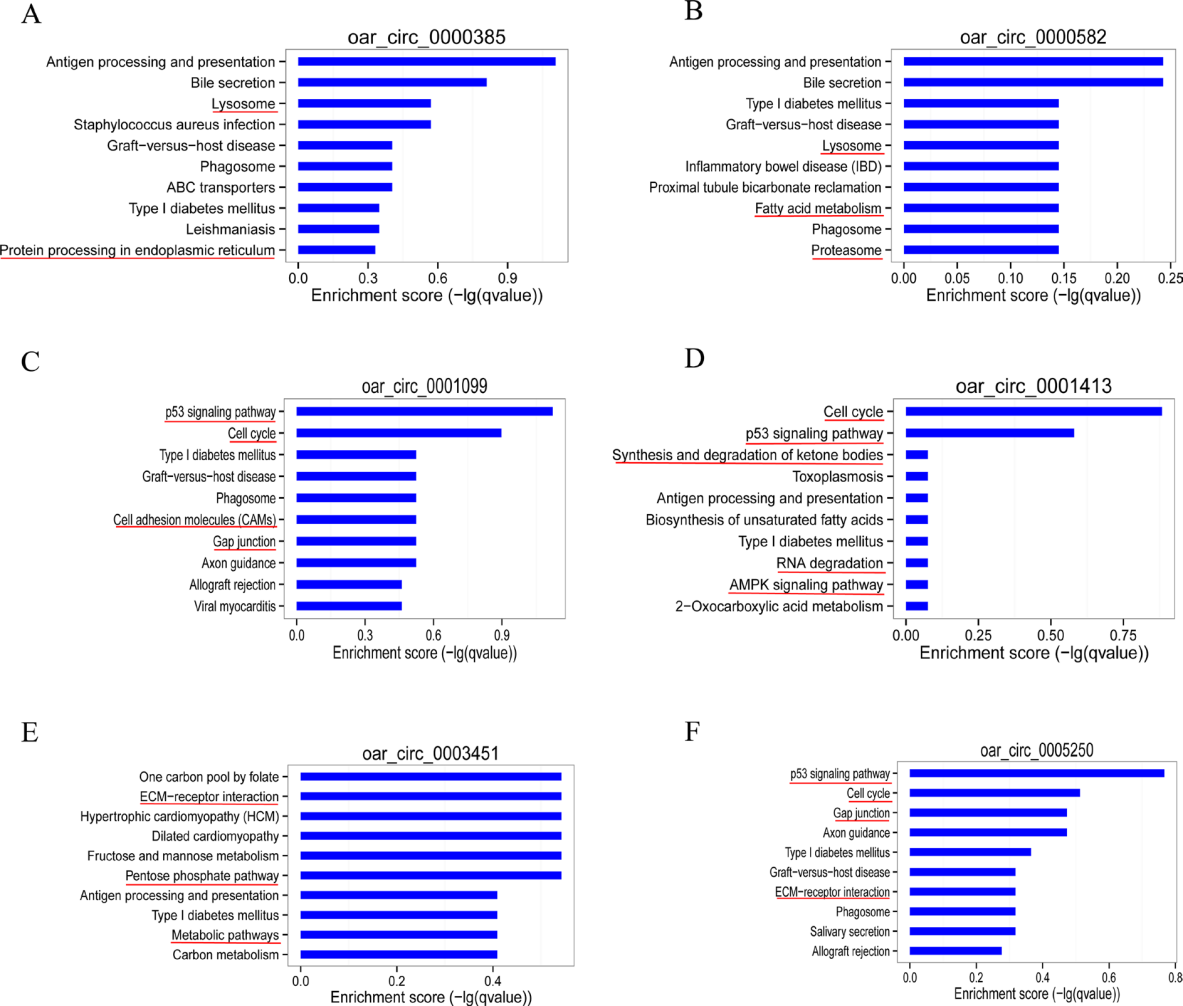
Bioinformatics analysis of circRNA-miRNA-mRNA network with potentially effective circRNAs Target mRNAs of the 6 networks (circRNA 0000385 (**A**), circRNA 0000582 (**B**), circRNA 0001099 (**C**), circRNA 0001413 (**D**), circRNA 0003451 (**E**), circRNA 0005250 (**F**)) are functionally annotated by KOBAS and KEGG pathway analysis. Top 10 significant (*P < 0.05*) enriched pathway terms of the 6 networks. Involving muscle growth and development of the signal pathway, marked with a red line.

To elucidate the function of circRNAs, we used TargetScan and miRanda to predict the differentially expressed circRNA target and downstream regulated mRNA, and establish the basic circRNA-miRNA connectivity. The circRNA-miRNA-mRNA interaction network was established using Cytoscape version 3.5.1 (Figure 6A, Supplementary File 4). In order to further understand the function of circRNA in skeletal muscle growth and development, we had further established a circRNA-miRNA-mRNA network with potentially effective circRNAs (circRNA 0000385, circRNA 0000582 and circRNA 0001099 etc), their predicted miRNA targets and downstream regulated mRNA (Figure 6B–6G). We used KEGG pathway analysis to functionally annotate the predicted mRNA targets within the above networks (As shown in Figure 7). These networks had several significantly enriched pathways related to muscle growth and development. This information may help us to explore the basic mechanism of circRNA in the growth and development of sheep skeletal muscle.

## DISCUSSION

With the development of high-throughput techniques, the deep sequencing of non-polyadenylated RNA populations indicates cumulative signals from certain exons (called excised exons) [47]. Further studies have shown that it is circRNAs [48]. In recent years, the genome of some breeds of sheep has been assembled and annotated, but sheep transcriptome analysis still needs to obtain more data from different tissue samples. Although thousands of unique circRNAs have been identified in cell types of different species, especially in humans and mice [49–53], and some of them have been demonstrated play an important role in animal development and growth. However, the circRNAs of sheep almost do not know. In this study, we used a comprehensive sequencing and analysis of the longest muscle ribosomal deletion of RNA from the embryonic and adult sheep, and 6113 circRNAs were successfully identified in numerous circRNAs.

As an important regulators of gene expression, circRNAs have an important role in growth and development of animal. A total of 5086 differentially expressed circRNAs between sheep embryos and adult muscle were identified by our study. These circRNAs may have specific biological effects on the development of muscle. The development and growth of muscle contains a number of changes in the expression of many genes and non-coding RNAs [54]. Sun et al. reported that lncRNAs played an important role in the development of bovine longissimus dorsi muscle [55]. Recently, Sun et al. reported that 5,566 lncRNA and 4,360 circRNA were differentially expressed in longissimus dorsi muscle of Landrace and Lantang pigs, indicating that there is a potentially post-transcriptional regulation of non-coding RNA involved in the development of pig muscle [56]. Therefore, we predicted that circRNAs may play a new post-transcriptional regulation during growth and development of muscle in sheep.

The current study of the function of circRNAs is mainly focused on competitive endogenous RNA (ceRNA). CircRNAs can function as a sponge for adsorbing miRNAs to influence post-transcriptional regulation [57–60]. In this study, miRNAs target sites were found in sheep circRNAs by using miRanda and psRobot. There are many circRNAs that interact with a lot of muscle-related miRNAs (miR-143, miR-133 and miR-23 etc). Interestingly, we found that some circRNAs contain simultaneously multiple target sites of different miRNAs. For example, oar_circ_0001413 contains target sites of miRNA-133, miRNA-125, miRNA-134, miRNA-103, miRNA-107, miRNA-1185, miRNA-181, miRNA-218, miRNA-329, simultaneously. One study has shown that circRNA (circHIPK3) regulates cell growth by sponging to 9 miRNAs with 18 potential binding sites [61]. Thus, as potential ceRNA, these circRNAs which can interact with multiple miRNAs may play a broad endogenous regulatory role in the growth and development of muscle. Oar_circ_0001413, as potential ceRNA, may be center regulator for growth and development of skeletal muscle, which will be analyzed in future study.

Current studies had shown that some pathways were involved in muscle growth, development and degradation, including AMPK signaling pathway, ECM-receptor interactions, ErbB signaling pathway, focal adhesion, valine, leucine and isoleucine degradation, ubiquitin mediated proteolysis, mTOR signaling pathway and Glutamatergic Synapse [62–69]. However, the report on the role of muscle circRNAs in sheep is limited. Our study clearly showed that circRNAs played an important role in regulating growth and development of muscle in sheep through enriched KEGG pathways and GO pathways. In particular, AMPK signaling pathway, valine, leucine and isoleucine degradation and focal adhesion are significantly enriched.

In conclusion, a number of circRNAs in the muscle of sheep were identified in our study. The differentially expressed circRNAs were screened in prenatal and postnatal muscle of sheep. At the same time, we found several circRNAs involved in the regulation of growth and development of muscle by KEGG pathway analysis. Our study provides valuable resources for circRNAs biology, especially in the muscle of the sheep, and helps for understanding the function of circRNA in sheep.

## MATERIALS AND METHODS

### Ethics statement

All procedures involving animals were approved by the Animal Care Committee of Shihezi University. The study was performed in accordance with the ethical standards laid down in the 1964 declaration of Helsinki and its later amendments.

### Animals

We collected longissimus dorsi muscle samples from three adult kazakh sheep (female) that had been slaughtered at the slaughterhouse. The longissimus dorsi muscle samples of three sheep embryos were carefully collected by surgery from estrus of three pregnant ewes after mating 100 days. Muscle samples were quickly dispensed into the cryopreserved tubes without RNase and immediately placed in liquid nitrogen until RNA isolation. All experiments involving animals were carried out under the protocol approved by the Animal Care Committee of Shihezi University.

### Library construction and circRNAs sequencing analysis

According to the manufacturer’s protocol, total RNAs were extracted from frozen tissues after grinding with liquid nitrogen using TRIzol (Invitrogen, CA, USA). Using Bioanalyzer 2100 and RNA6000 Nano Kit (Agilent, CA, USA) detected quantity and purity of total RNAs. Take the same amount of RNAs from three adult sheep and three embryos sheep were respectively pooled to construct library. In order to establish a cDNA library of circRNAs, we take approximately 10 μg of total RNA and use Rnase R (RNR-07250, epicentre) to digest linear RNA. We used IlluminaHiseq 2000/2500 to perform paired-end sequencing on the cDNA library of circRNAs according to the recommended protocol. CircRNAs analysis was performed following the steps in the pipeline (Figure 1). Low quality data was deleted, and reference genome and gene annotation was downloaded from the genome website (http:/genome.ucsc.edu/). Index of the reference genome was built using Bowtie v2.0.6, and paired-end clean reads were aligned to the reference genome by using TopHat v2.0.9. CircRNAs candidates were predicted as described previously [40]. Only those who contain more than two independent junction-spanning reads and correspond to the GU / AG intron rules can be determined as a circRNA.

### Target site prediction and enrichment analysis

The interaction of the circRNAs and miRNAs were analyzed using miRanda and psRobot [41, 42]. Gene Ontology (GO) analysis was used to host genes of the circRNAs using DAVID software [43], and A KEGG enrichment analysis was used to host genes of circRNAs using KOBAS software [44, 45]. *P* < 0.05 was used as a criterion for the determination of whether the enrichment analysis was significant

### RT-PCR analysis and DNA sequencing

Total RNAs were extracted from sheep muscle using TRIzol (Invitrogen, CA, USA), and 10 μg of purified RNAs were used to synthesize cDNA using RT-PCR kit (Takara, Dalian, China). Ten specific primers (circRNA 0002456, circRNA 0000552, circRNA 0005179, circRNA 0005256, circRNA 0003451, circRNA 0005243, circRNA 0005250, circRNA 0004676, circRNA 0004690 and circRNA 0000666) were used for circRNAs PCR and the primer sequences for these circRNAs were listed in Supplementary Table 1. The PCR products were analyzed by gel electrophoresis and DNA sequencing. We used DNAMAN software to compare the sequence data of PCR products obtained from DNA sequencing with the sheep reference genome and RNA-seq data.

### Real-Time RT-PCR analysis

We used Real-Time RT-PCR to detect the expression levels of 10 circRNAs (circRNA 0002456, circRNA 0000552, circRNA 0005179, circRNA 0005256, circRNA 0003451, circRNA 0005243, circRNA 0005250, circRNA 0004676, circRNA 0004690 and circRNA 0000666). Total RNA was treated with RNase R (RNR-07250, epicenter) prior to cDNA synthesis to detect the resistance of circRNAs to RNase R digestion. Total RNA was synthesized by RT-PCR kit (Takara, Dalian, China) and real-time RT-PCR was used to verify the differentially expressed. All Real-Time RT-PCR analyzes were performed using SYBR Green (TaKaRa Biotech, Dalian) according to the manufacturer’s protocol. We selected linear β-actin as an internal reference to normalize the expression of circRNA [46]. Three independent experiments were performed on triplicate samples.

### Data availability

The datasets generated during and/or analysed during the current study are available from the corresponding author on reasonable request.

## SUPPLEMENTARY MATERIALS FIGURES AND TABLES










