# Supplementary material for: Genome-wide analysis of circular RNAs in prenatal and postnatal muscle of sheep
Source: Oncotarget. 2017 Oct 12;8(57):97165–77. doi: 10.18632/oncotarget.21835 (PMC5722553; doi:10.18632/oncotarget.21835)
Supplement: Supplementary file 1 [file oncotarget-08-97165-s001.pdf]

Genome-wide analysis of circular RNAs in prenatal and postnatal muscle of sheep

SUPPLEMENTARY MATERIALS

Supplementary Table 1: Details of primers and amplicons of the 10 circRNAs used for RT-PCR and β-Actin for Real-Time PCR

Gene name	Primer sequence (forward/reverse)	Annealing Temperature (°C)	Primer length (bp)	Amplicon size (bp)
circ-0002456	AAACCTAAGCATTGCCAGTA	51.30	20	147
	CCTTTCAGGATATTTTCTTCATA	50.64	23	
circ-0005179	TCAGATGTGTTCCACCTTTT	51.30	20	169
	GTTGTTGATGGTTTGGAGTG	53.35	20	
circ-0000666	TGTGGGTACAGCATGTCTCTT	55.61	21	177
	AAACGTAATCTGATTTCGGTCT	52.08	22	
circ-0000552	CATTCCCGAAGTGCCATAAC	55.40	20	176
	TCCACTGGGTTCACTGATAAGAG	57.77	23	
circ-0005250	GCAGCGTGTGAAGCAGAAG	57.32	19	149
	GGTGCTCATTCATCTGGTCCT	57.57	21	
circ-0005256	CCAACACCAACCTGTCCAAG	57.45	20	148
	ATGCGGCAGACCAAGAAGA	55.16	19	
circ-0003541	GTGGACGCCATTCCCCTGC	61.64	19	106
	GAGCCCCAATCATCTTCCCTTT	57.67	22	
circ-0005243	GGTGAAGGTGGGCAACGAGTA	59.52	21	153
	TGGCGAGGCTGCTTGGTC	59.46	18	
circ-0004690	GCTTACCCACCAGAGTATTATCAA	56.15	24	155
	GACAATCTATGCCTGCTACGAG	57.67	22	
circ-0004676	AGTCCAATTTTCACGACCACA	59.50	20	136
	CAGCTACTCCTTGCCACAGG	53.66	21	
β-Actin	CCAACCGTGAGAAGATGACC	57.45	20	97
	CCAGAGGCGTACAGGGACAG	61.55	20	

Supplementary File 1: Detailed comments for all circRNAs. See Supplementary_File_1

Supplementary File 2: KEGG pathway analysis demonstrated 270 terms were enriched. Supplementary_File_2

Supplementary File 3: Interaction relationships between circRNAs and various miRNAs were found. See Supplementary_File_3

Supplementary File 4: The circRNA-miRNA-mRNA interaction network between circRNA and its target gene. See Supplementary_File_4
