# Supplementary material for: Genome-wide analysis of circular RNAs in prenatal and postnatal muscle of sheep
Source: Oncotarget. 2017 Oct 12;8(57):97165–77. doi: 10.18632/oncotarget.21835 (PMC5722553; doi:10.18632/oncotarget.21835)
Supplement: Supplementary file 2 [file oncotarget-08-97165-s002.doc]

***Supplementary material for***

### Genome-wide analysis of circular RNAs [in prenatal and postnatal](http://xueshu.baidu.com/s?wd=paperuri%3A(9e30a81034984a8be3889223ca73f061)&filter=sc_long_sign&sc_ks_para=q%3DThe evolution ofHomo sapiens denisovaandHomo sapiens neanderthalensismiRNA targeting genes in the prenatal and postnatal brain&sc_us=17431911151666729642&tn=SE_baiduxueshu_c1gjeupa&ie=utf-8) longissimus dorsi muscle of sheep

Cunyuan Li^1^†, Xiaoyue Li^1^†, Qiman Ma^1^, Xiangyu Zhang^1^, Yang Cao^1^, Yang Yao^1^, Wureli Hazi^2^, Dawei Wang^1^, Renzhe Quan^1^, Xiaoxu Hou^1^, Zhijin Liu^1^, Qianqian Zhan^1^, Li Liu^1^, Mengdan Zhang^1^, Shuting Yu^1^, Wei Ni^1,*^, Shengwei Hu^1,*^

^1^College of Life Sciences, Shihezi University, Shihezi, Xinjiang, 832003, China

†These authors contributed equally to this work

*Corresponding author: Email: [niweiwonderful@sina.com](mailto:niweiwonderful@sina.com); E-mail: [hushengwei@163.com](mailto:hushengwei@163.com)

**Supporting information**

Supplementary File 1. Detailed comments for all circRNAs

circRNA_id chr start end strand full_length spliced_length gene_id feature samples junction_read non_junction_read Gene_Name

oar_circ_0000001 JH921919.1 3961 4213 - 252 252 -- -- LDM_E 3 0 --

oar_circ_0000002 JH921943.1 822 2128 - 1306 753 ENSOARG00000001368; intron_1:1545-1917,exon_3:1164-1545,; LDM_A 3 2 -

oar_circ_0000003 JH922355.1 2833 3150 - 317 317 -- -- LDM_A 4 0 --

oar_circ_0000004 JH922959.1 19078 19365 + 287 287 -- -- LDM_A 5 0 --

oar_circ_0000005 JH923012.1 12381 13817 - 1436 1436 -- -- LDM_E,LDM_A 38,6 0,0 --

oar_circ_0000006 JH923339.1 4893 5139 - 246 246 -- -- LDM_E 2 0 --

oar_circ_0000007 JH923649.1 2183 7020 + 4837 2550 ENSOARG00000010785;ENSOARG00000010910; exon_3:2183-2579,exon_4:3126-3679,intron_3:2579-3126,;exon_3:5025-5226,exon_1:3892-4306,intron_1:4306-4745,; LDM_E 25 126 -;-

oar_circ_0000008 JH923665.1 776 2421 + 1645 1645 -- -- LDM_E,LDM_A 11,4 0,0 --

oar_circ_0000009 JH923750.1 122 1330 + 1208 1208 -- -- LDM_E 3 0 --

oar_circ_0000010 JH923837.1 9374 16812 + 7438 2538 ENSOARG00000018658; exon_1:10507-13045,; LDM_E 3 273 GPRASP2

oar_circ_0000011 JH923883.1 5787 12370 - 6583 444 ENSOARG00000001434; exon_1:5827-6271,; LDM_E 2 22 KCNH2

oar_circ_0000012 JH923979.1 370 880 + 510 254 ENSOARG00000020080; exon_2:370-624,; LDM_E,LDM_A 59,7 41,8 -

oar_circ_0000013 JH924156.1 285 2817 - 2532 1452 ENSOARG00000000300;ENSOARG00000000308; exon_1:1111-1448,;exon_2:1797-2039,intron_1:2039-2598,exon_1:2598-2912,; LDM_E 26 36 -;-

oar_circ_0000014 JH924192.1 424 6015 + 5591 1069 ENSOARG00000000913; intron_1:4603-4931,intron_3:5153-5894,; LDM_E,LDM_A 23,16 21,5 -

oar_circ_0000015 JH924365.1 453 4998 + 4545 3541 ENSOARG00000002435;ENSOARG00000002437; intron_4:1284-2959,;exon_2:2959-3576,intron_2:3576-4825,; LDM_E,LDM_A 5,2 13,9 -;-

oar_circ_0000016 JH924536.1 141 2019 - 1878 1878 -- -- LDM_A 4 0 --

oar_circ_0000017 JH924672.1 504 1017 - 513 513 ENSOARG00000017645; intron_1:153-1368,; LDM_E,LDM_A 12,32 16,47 -

oar_circ_0000018 chr10 11491038 11496390 - 5352 5063 ENSOARG00000006823; intron_5:11491135-11492650,intron_6:11492700-11493467,intron_8:11493908-11496319,intron_7:11493484-11493854,; LDM_E,LDM_A 32,34 483,440 SUGT1

oar_circ_0000019 chr10 11535093 11536868 - 1775 1667 ENSOARG00000006858; exon_4:11535093-11535261,intron_5:11535261-11536760,; LDM_E,LDM_A 7,114 37,52 ELF1

oar_circ_0000020 chr10 11637371 11639583 + 2212 2088 ENSOARG00000006905; intron_4:11637495-11639415,exon_5:11639415-11639583,; LDM_E 2 60 WBP4

oar_circ_0000021 chr10 11639415 11641384 + 1969 1846 ENSOARG00000006905; exon_5:11639415-11639583,intron_5:11639583-11640804,intron_6:11640851-11641308,; LDM_E 2 59 WBP4

oar_circ_0000022 chr10 11905052 11911441 + 6389 5939 ENSOARG00000007053; intron_14:11905266-11906156,intron_15:11906344-11908382,intron_18:11909464-11911343,intron_17:11909015-11909320,exon_15:11906156-11906344,exon_14:11905052-11905266,intron_16:11908491-11908916,; LDM_A 2 192 NAA16

oar_circ_0000023 chr10 11975779 11978540 + 2761 2593 ENSOARG00000007092; intron_1:11975884-11978477,; LDM_A 2 259 RGCC

oar_circ_0000024 chr10 12293511 12306086 - 12575 12173 ENSOARG00000007167; intron_26:12293583-12298901,intron_27:12299008-12303083,intron_28:12303190-12305970,; LDM_E,LDM_A 2,2 176,139 VWA8

oar_circ_0000025 chr10 12293511 12310305 - 16794 16314 ENSOARG00000007167; intron_26:12293583-12298901,intron_28:12303190-12305970,intron_27:12299008-12303083,intron_29:12306086-12310227,; LDM_E,LDM_A 3,5 237,182 VWA8

oar_circ_0000026 chr10 12293511 12314061 - 20550 20070 ENSOARG00000007167; exon_15:12313892-12314061,intron_27:12299008-12303083,intron_29:12306086-12310227,intron_28:12303190-12305970,intron_30:12310305-12313892,intron_26:12293583-12298901,; LDM_E,LDM_A 5,3 321,226 VWA8

oar_circ_0000027 chr10 12692615 12692889 + 274 274 ENSOARG00000007286; exon_14:12692615-12692889,; LDM_A 3 20 DGKH

oar_circ_0000028 chr10 12693804 12702649 + 8845 8130 ENSOARG00000007286; intron_16:12698844-12701187,intron_15:12693927-12698718,intron_17:12701305-12702301,; LDM_E,LDM_A 11,7 194,51 DGKH

oar_circ_0000029 chr10 12698718 12702649 + 3931 3339 ENSOARG00000007286; intron_17:12701305-12702301,intron_16:12698844-12701187,; LDM_E,LDM_A 2,2 119,28 DGKH

oar_circ_0000030 chr10 12713581 12715341 + 1760 1035 ENSOARG00000007286; intron_22:12714306-12715177,exon_23:12715177-12715341,; LDM_A 5 5 DGKH

oar_circ_0000031 chr10 12787010 12802458 + 15448 15038 ENSOARG00000007363; intron_1:12787120-12792768,exon_5:12802193-12802458,intron_2:12792885-12795683,intron_4:12797376-12802193,intron_3:12795731-12797241,; LDM_E 8 661 AKAP11

oar_circ_0000032 chr10 13964149 13965440 - 1291 1009 ENSOARG00000007449; intron_13:13964287-13965296,; LDM_E 2 11 ENOX1

oar_circ_0000033 chr10 15173686 15173865 + 179 179 -- -- LDM_E 2 0 --

oar_circ_0000034 chr10 15227268 15230839 + 3571 3571 -- -- LDM_A 2 0 --

oar_circ_0000035 chr10 15911336 15916942 + 5606 5162 ENSOARG00000007620; intron_2:15911451-15913514,intron_3:15913662-15915752,intron_4:15915847-15916856,; LDM_E,LDM_A 5,2 162,46 SLC25A30

oar_circ_0000036 chr10 15915752 15919591 + 3839 3437 ENSOARG00000007620; intron_4:15915847-15916856,intron_5:15916942-15918721,intron_6:15918817-15919466,; LDM_E 4 141 SLC25A30

oar_circ_0000037 chr10 15918721 15924533 + 5812 5223 ENSOARG00000007620; intron_7:15919591-15921826,intron_8:15921965-15923322,intron_6:15918817-15919466,intron_9:15923403-15924385,; LDM_E 2 139 SLC25A30

oar_circ_0000038 chr10 15918721 15924546 + 5825 5223 ENSOARG00000007620; intron_7:15919591-15921826,intron_6:15918817-15919466,intron_9:15923403-15924385,intron_8:15921965-15923322,; LDM_E 3 139 SLC25A30

oar_circ_0000039 chr10 15953933 15955727 + 1794 1499 ENSOARG00000007671; intron_1:15954166-15955665,; LDM_E 6 36 COG3

oar_circ_0000040 chr10 15954019 15972992 + 18973 17874 ENSOARG00000007671; intron_3:15957188-15957965,intron_7:15962861-15964520,exon_12:15968860-15969021,intron_5:15959242-15959930,intron_8:15964564-15965033,intron_4:15958040-15959149,intron_2:15955727-15957022,intron_10:15965844-15966971,intron_6:15960056-15962780,exon_3:15957022-15957188,intron_1:15954166-15955665,intron_9:15965160-15965752,intron_12:15969021-15972880,intron_11:15967111-15968860,; LDM_E 3 810 COG3

oar_circ_0000041 chr10 15957022 15972992 + 15970 15080 ENSOARG00000007671; intron_6:15960056-15962780,intron_8:15964564-15965033,intron_12:15969021-15972880,intron_9:15965160-15965752,intron_5:15959242-15959930,intron_4:15958040-15959149,exon_3:15957022-15957188,exon_12:15968860-15969021,intron_3:15957188-15957965,intron_7:15962861-15964520,intron_10:15965844-15966971,intron_11:15967111-15968860,; LDM_A 2 262 COG3

oar_circ_0000042 chr10 15972880 15976873 + 3993 3756 ENSOARG00000007671; intron_13:15972992-15976748,; LDM_E,LDM_A 3,5 66,26 COG3

oar_circ_0000043 chr10 15972880 15988017 + 15137 14357 ENSOARG00000007671; intron_15:15981463-15984792,intron_14:15976873-15981373,intron_13:15972992-15976748,intron_16:15984913-15987685,; LDM_E 2 1140 COG3

oar_circ_0000044 chr10 15981373 15988017 + 6644 6101 ENSOARG00000007671; intron_15:15981463-15984792,intron_16:15984913-15987685,; LDM_E,LDM_A 10,9 775,287 COG3

oar_circ_0000045 chr10 16437893 16461022 - 23129 22989 ENSOARG00000007790; exon_9:16437893-16438358,exon_7:16453870-16454068,exon_6:16459708-16459869,intron_12:16441181-16453870,intron_11:16438358-16440870,intron_14:16459869-16460882,intron_13:16454068-16459708,exon_8:16440870-16441181,; LDM_A 3 484 ZC3H13

oar_circ_0000046 chr10 16453870 16470676 - 16806 16557 ENSOARG00000007790; intron_15:16461022-16470567,intron_14:16459869-16460882,exon_7:16453870-16454068,intron_13:16454068-16459708,exon_6:16459708-16459869,; LDM_E 6 713 ZC3H13

oar_circ_0000047 chr10 16491924 16495204 - 3280 2932 ENSOARG00000007790; intron_18:16494780-16495078,intron_17:16492036-16494670,; LDM_E 3 179 ZC3H13

oar_circ_0000048 chr10 17008271 17009626 + 1355 1355 ENSOARG00000008014; intron_8:17008399-17012745,; LDM_E 2 52 LRCH1

oar_circ_0000049 chr10 18383481 18388421 + 4940 4514 ENSOARG00000008230; exon_3:18385085-18385292,intron_3:18385292-18386126,intron_2:18383610-18385085,exon_5:18386958-18387109,intron_5:18387109-18388235,intron_4:18386237-18386958,; LDM_E 6 531 ITM2B

oar_circ_0000050 chr10 18383481 18392390 + 8909 4514 ENSOARG00000008230; intron_2:18383610-18385085,exon_3:18385085-18385292,exon_5:18386958-18387109,intron_4:18386237-18386958,intron_3:18385292-18386126,intron_5:18387109-18388235,; LDM_E,LDM_A 5,2 531,352 ITM2B

oar_circ_0000051 chr10 18496212 18508838 + 12626 11899 ENSOARG00000008246; intron_18:18507665-18508641,exon_19:18508641-18508838,intron_14:18499832-18505768,intron_15:18505885-18507331,intron_13:18497366-18499744,intron_12:18496322-18497288,; LDM_E,LDM_A 8,7 448,189 RB1

oar_circ_0000052 chr10 18552839 18554603 + 1764 1472 ENSOARG00000008246; intron_21:18552985-18554457,; LDM_E 8 57 RB1

oar_circ_0000053 chr10 19352889 19362511 + 9622 9311 ENSOARG00000008487; intron_7:19356120-19357751,intron_8:19357971-19358496,exon_11:19362185-19362511,intron_9:19358596-19359949,exon_8:19357751-19357971,intron_6:19353006-19355950,intron_10:19360043-19362185,exon_7:19355950-19356120,; LDM_A 2 124 SETDB2

oar_circ_0000054 chr10 19371019 19374121 + 3102 3102 ENSOARG00000008531; intron_1:19371242-19377446,; LDM_E,LDM_A 5,30 55,25 -

oar_circ_0000055 chr10 19371022 19374121 + 3099 3099 ENSOARG00000008531; intron_1:19371242-19377446,; LDM_A 7 25 -

oar_circ_0000056 chr10 19413957 19421661 - 7704 7573 ENSOARG00000008556; intron_1:19414121-19421530,exon_12:19413957-19414121,; LDM_E 2 253 RCBTB1

oar_circ_0000057 chr10 19439465 19448600 - 9135 8796 ENSOARG00000008556; intron_10:19447723-19448242,exon_4:19439465-19439632,intron_9:19439632-19447572,exon_2:19448242-19448412,; LDM_E 3 250 RCBTB1

oar_circ_0000058 chr10 19447572 19448600 - 1028 689 ENSOARG00000008556; exon_2:19448242-19448412,intron_10:19447723-19448242,; LDM_E,LDM_A 7,4 9,3 RCBTB1

oar_circ_0000059 chr10 1970240 2006252 - 36012 35861 ENSOARG00000006244; exon_4:1970240-1970401,intron_12:2001847-2006167,intron_11:1970401-2001781,; LDM_E 4 1629 TDRD3

oar_circ_0000060 chr10 19752733 19753557 - 824 824 -- -- LDM_E 2 0 --

oar_circ_0000061 chr10 19795097 19797596 - 2499 2499 -- -- LDM_E 2 0 --

oar_circ_0000062 chr10 19860459 19882224 + 21765 331 ENSOARG00000021607; exon_1:19866185-19866516,; LDM_E 4 16 DLEU1_2

oar_circ_0000063 chr10 20945843 20946739 + 896 896 ENSOARG00000008823; exon_3:20945843-20946739,; LDM_E 2 25 FAM124A

oar_circ_0000064 chr10 22093247 22104968 + 11721 11356 ENSOARG00000009534; exon_3:22097663-22097819,exon_2:22093247-22093532,intron_5:22101283-22104824,intron_4:22098698-22101203,intron_2:22093532-22097663,intron_3:22097819-22098557,; LDM_A 2 408 MRPS31

oar_circ_0000065 chr10 23486090 23486683 - 593 445 ENSOARG00000009644; intron_6:23486238-23486524,exon_2:23486524-23486683,; LDM_E,LDM_A 24,25 12,3 NHLRC3

oar_circ_0000066 chr10 24891008 24906373 + 15365 14226 ENSOARG00000010140; intron_8:24900871-24902158,intron_2:24894366-24895016,intron_5:24898146-24899155,intron_6:24899259-24900584,exon_13:24906215-24906373,intron_1:24891105-24894330,intron_11:24904064-24905844,intron_9:24902298-24903757,intron_12:24905916-24906215,intron_3:24895075-24895527,exon_10:24903757-24903914,intron_4:24895594-24898019,; LDM_A 2 237 SUPT20H

oar_circ_0000067 chr10 24925276 24929021 - 3745 3292 ENSOARG00000010229; intron_6:24926364-24927074,intron_7:24927120-24928947,intron_4:24925373-24926128,; LDM_E 2 66 EXOSC8

oar_circ_0000068 chr10 25025451 25025708 + 257 257 ENSOARG00000010293; exon_6:25025451-25025708,; LDM_A 2 1 SMAD9

oar_circ_0000069 chr10 25609017 25609364 + 347 347 -- -- LDM_E 6 0 --

oar_circ_0000070 chr10 25693978 25696627 + 2649 2649 -- -- LDM_E 2 0 --

oar_circ_0000071 chr10 26015026 26032511 - 17485 17311 ENSOARG00000010627; exon_54:26015026-26015223,intron_7:26030471-26032318,exon_51:26028336-26028492,exon_49:26032318-26032511,exon_52:26027641-26027812,intron_5:26027812-26028336,intron_3:26015223-26017010,intron_4:26017112-26027641,intron_6:26028492-26030399,; LDM_E 2 1073 NBEA

oar_circ_0000072 chr10 26027641 26032511 - 4870 4798 ENSOARG00000010627; intron_6:26028492-26030399,exon_49:26032318-26032511,exon_51:26028336-26028492,intron_7:26030471-26032318,exon_52:26027641-26027812,intron_5:26027812-26028336,; LDM_E 2 136 NBEA

oar_circ_0000073 chr10 26428190 26430683 - 2493 2360 ENSOARG00000010627; intron_26:26428323-26430528,exon_30:26430528-26430683,; LDM_E 3 152 NBEA

oar_circ_0000074 chr10 26428190 26441967 - 13777 13644 ENSOARG00000010627; exon_29:26441528-26441967,intron_26:26428323-26430528,intron_27:26430683-26441528,exon_30:26430528-26430683,; LDM_E 2 901 NBEA

oar_circ_0000075 chr10 26570635 26592574 - 21939 21226 ENSOARG00000010627; intron_50:26570782-26572633,intron_54:26588775-26589016,exon_1:26592342-26592574,intron_51:26572753-26577798,intron_53:26579923-26588679,intron_55:26589117-26592342,intron_52:26577925-26579801,; LDM_E,LDM_A 8,3 2013,234 NBEA

oar_circ_0000076 chr10 26577798 26592574 - 14776 14330 ENSOARG00000010627; intron_52:26577925-26579801,exon_1:26592342-26592574,intron_55:26589117-26592342,intron_53:26579923-26588679,intron_54:26588775-26589016,; LDM_E 3 1694 NBEA

oar_circ_0000077 chr10 26588679 26592574 - 3895 3698 ENSOARG00000010627; intron_54:26588775-26589016,intron_55:26589117-26592342,exon_1:26592342-26592574,; LDM_E,LDM_A 8,4 639,61 NBEA

oar_circ_0000078 chr10 28577656 28578297 - 641 440 ENSOARG00000010917; exon_31:28577857-28578297,; LDM_E,LDM_A 59,48 330,157 PDS5B

oar_circ_0000079 chr10 28609239 28621459 - 12220 12027 ENSOARG00000010917; intron_15:28617961-28621335,intron_14:28609308-28617802,exon_20:28617802-28617961,; LDM_A 3 223 PDS5B

oar_circ_0000080 chr10 28617802 28621459 - 3657 3533 ENSOARG00000010917; intron_15:28617961-28621335,exon_20:28617802-28617961,; LDM_E,LDM_A 7,3 111,31 PDS5B

oar_circ_0000081 chr10 28638252 28651716 - 13464 12971 ENSOARG00000010917; intron_19:28645129-28646560,intron_20:28646700-28648972,intron_17:28638413-28639771,intron_18:28639877-28645013,intron_21:28649021-28651634,exon_18:28638252-28638413,; LDM_E,LDM_A 2,2 665,189 PDS5B

oar_circ_0000082 chr10 28645013 28660545 - 15532 15031 ENSOARG00000010917; intron_20:28646700-28648972,intron_19:28645129-28646560,exon_11:28660393-28660545,intron_22:28651716-28659040,intron_23:28659154-28660393,intron_21:28649021-28651634,; LDM_E 2 809 PDS5B

oar_circ_0000083 chr10 28988123 28990004 - 1881 1770 ENSOARG00000011408; exon_64:28988123-28988326,intron_2:28988326-28989893,; LDM_E,LDM_A 3,7 59,20 FRY

oar_circ_0000084 chr10 29028872 29029476 - 604 506 ENSOARG00000011408; intron_18:29029043-29029378,exon_48:29028872-29029043,; LDM_A 2 9 FRY

oar_circ_0000085 chr10 29059909 29060791 - 882 735 ENSOARG00000011408; intron_32:29060056-29060534,exon_33:29060534-29060791,; LDM_E,LDM_A 3,15 21,23 FRY

oar_circ_0000086 chr10 29910091 29916863 - 6772 6438 ENSOARG00000011694; intron_3:29910211-29914059,intron_4:29914159-29916749,; LDM_E,LDM_A 5,11 100,21 B3GLCT

oar_circ_0000087 chr10 29935991 29952003 - 16012 15622 ENSOARG00000011694; intron_10:29951425-29951926,intron_8:29936055-29943791,intron_9:29943928-29951313,; LDM_E 2 260 B3GLCT

oar_circ_0000088 chr10 30433202 30433359 - 157 157 -- -- LDM_A 3 0 --

oar_circ_0000089 chr10 30784502 30784853 + 351 351 -- -- LDM_E 2 0 --

oar_circ_0000090 chr10 31242781 31243993 - 1212 993 ENSOARG00000012051; intron_3:31242901-31243894,; LDM_E 5 10 MTUS2

oar_circ_0000091 chr10 31662750 31662964 + 214 214 ENSOARG00000012100; exon_1:31662750-31662964,; LDM_A 3 17 SLC46A3

oar_circ_0000092 chr10 31901546 31914634 + 13088 12595 ENSOARG00000012180; intron_4:31901671-31905603,intron_6:31906016-31909747,intron_9:31913871-31914474,exon_10:31914474-31914634,exon_9:31913701-31913871,exon_7:31909747-31909922,exon_5:31905603-31905766,intron_7:31909922-31911304,intron_8:31911422-31913701,; LDM_E,LDM_A 3,2 257,219 FLT1

oar_circ_0000093 chr10 32017367 32022270 + 4903 4107 ENSOARG00000012180; intron_20:32017456-32021563,; LDM_E 2 8 FLT1

oar_circ_0000094 chr10 32915489 32923386 + 7897 7816 ENSOARG00000012677; intron_4:32915570-32923172,exon_5:32923172-32923386,; LDM_E 4 191 USP12

oar_circ_0000095 chr10 33160169 33166307 - 6138 5886 ENSOARG00000012699; intron_9:33160287-33163946,intron_10:33164104-33166173,exon_4:33163946-33164104,; LDM_E,LDM_A 3,2 65,60 WASF3

oar_circ_0000096 chr10 33312552 33330976 - 18424 17653 ENSOARG00000012732; intron_3:33312907-33313564,exon_12:33312552-33312711,intron_7:33320927-33328513,intron_8:33328645-33330918,intron_6:33318311-33320783,intron_5:33317423-33318241,intron_4:33313662-33317350,; LDM_E 2 840 CDK8

oar_circ_0000097 chr10 33328513 33330976 - 2463 2273 ENSOARG00000012732; intron_8:33328645-33330918,; LDM_A 2 82 CDK8

oar_circ_0000098 chr10 33348341 33362755 - 14414 14086 ENSOARG00000012732; intron_11:33352317-33362679,intron_10:33348482-33352206,; LDM_E,LDM_A 13,10 492,386 CDK8

oar_circ_0000099 chr10 33562771 33563286 + 515 515 -- -- LDM_E,LDM_A 38,13 0,0 --

oar_circ_0000100 chr10 34834920 34837835 + 2915 2656 ENSOARG00000013419; intron_4:34835032-34837688,; LDM_A 2 48 SACS

oar_circ_0000101 chr10 35756780 35763814 + 7034 6689 ENSOARG00000013613; intron_6:35763092-35763693,intron_4:35756901-35761942,intron_5:35762012-35763059,; LDM_A 5 271 ZDHHC20

oar_circ_0000102 chr10 35756780 35773568 + 16788 15795 ENSOARG00000013613; intron_7:35763814-35772920,intron_4:35756901-35761942,intron_6:35763092-35763693,intron_5:35762012-35763059,; LDM_E,LDM_A 17,4 717,412 ZDHHC20

oar_circ_0000103 chr10 35862424 35862947 + 523 522 ENSOARG00000013729; exon_2:35862695-35862947,exon_1:35862424-35862694,; LDM_E,LDM_A 19,11 67,38 LATS2

oar_circ_0000104 chr10 35915241 35924882 + 9641 9254 ENSOARG00000013784; intron_1:35915347-35920542,intron_2:35920684-35924743,; LDM_A 2 179 XPO4

oar_circ_0000105 chr10 35934561 35951600 + 17039 16808 ENSOARG00000013784; intron_7:35951228-35951425,intron_6:35946858-35951070,exon_5:35935373-35935526,intron_4:35934678-35935373,exon_8:35951425-35951600,exon_7:35951070-35951228,intron_5:35935526-35946744,; LDM_A 2 813 XPO4

oar_circ_0000106 chr10 35951070 35951600 + 530 530 ENSOARG00000013784; intron_7:35951228-35951425,exon_8:35951425-35951600,exon_7:35951070-35951228,; LDM_A 2 18 XPO4

oar_circ_0000107 chr10 36468778 36486403 + 17625 17444 ENSOARG00000014163; intron_4:36477501-36481613,exon_3:36468778-36469080,intron_5:36481810-36486318,exon_5:36481613-36481810,intron_3:36469080-36477405,; LDM_E 2 683 PSPC1

oar_circ_0000108 chr10 36490665 36490850 + 185 185 ENSOARG00000014163; intron_7:36490010-36507645,; LDM_A 2 3 PSPC1

oar_circ_0000109 chr10 36678283 36679397 + 1114 946 ENSOARG00000014560; intron_2:36678789-36679273,exon_2:36678327-36678789,; LDM_E 2 88 CENPJ

oar_circ_0000110 chr10 36678327 36679397 + 1070 946 ENSOARG00000014560; exon_2:36678327-36678789,intron_2:36678789-36679273,; LDM_E,LDM_A 16,4 88,21 CENPJ

oar_circ_0000111 chr10 36678327 36690651 + 12324 11706 ENSOARG00000014560; exon_2:36678327-36678789,exon_4:36683210-36683513,intron_4:36683513-36685468,intron_3:36679397-36683210,intron_5:36685580-36686492,exon_7:36686720-36688368,intron_7:36688368-36690497,intron_2:36678789-36679273,; LDM_E 2 436 CENPJ

oar_circ_0000112 chr10 36678327 36700265 + 21938 21235 ENSOARG00000014560; intron_2:36678789-36679273,exon_7:36686720-36688368,intron_5:36685580-36686492,intron_9:36693850-36696250,intron_7:36688368-36690497,exon_9:36693684-36693850,intron_3:36679397-36683210,intron_4:36683513-36685468,exon_10:36696250-36696493,exon_2:36678327-36678789,intron_10:36696493-36700180,intron_8:36690651-36693684,exon_4:36683210-36683513,; LDM_E 6 519 CENPJ

oar_circ_0000113 chr10 46568998 46582245 - 13247 12886 ENSOARG00000015209; intron_3:46572783-46582093,intron_1:46569067-46569560,exon_8:46582093-46582245,intron_2:46569704-46572635,; LDM_E 2 211 DACH1

oar_circ_0000114 chr10 46882629 46883076 - 447 447 ENSOARG00000015209; intron_10:46802806-46994719,; LDM_E 2 15 DACH1

oar_circ_0000115 chr10 47968838 47973036 - 4198 3458 ENSOARG00000015309; intron_15:47970776-47971953,intron_16:47972118-47972868,intron_11:47968940-47969200,exon_6:47971953-47972118,exon_5:47972868-47973036,intron_13:47969843-47970402,intron_12:47969317-47969696,; LDM_E 5 112 DIS3

oar_circ_0000116 chr10 47997326 48007857 + 10531 10310 ENSOARG00000015416; exon_3:48002417-48002616,intron_2:47997427-48002417,intron_3:48002616-48007737,; LDM_A 3 18 PIBF1

oar_circ_0000117 chr10 49128330 49128877 - 547 547 ENSOARG00000015513; exon_4:49128330-49128877,; LDM_E 2 144 KLF12

oar_circ_0000118 chr10 49381063 49384602 - 3539 3539 ENSOARG00000015513; intron_7:49325599-49491065,; LDM_E 2 537 KLF12

oar_circ_0000119 chr10 50841221 50851966 - 10745 10289 ENSOARG00000015582; intron_18:50845827-50846097,intron_17:50841341-50845716,intron_19:50846189-50851833,; LDM_E,LDM_A 4,3 663,58 TBC1D4

oar_circ_0000120 chr10 50845716 50862718 - 17002 16561 ENSOARG00000015582; intron_18:50845827-50846097,intron_19:50846189-50851833,intron_20:50851966-50862613,; LDM_E 2 1199 TBC1D4

oar_circ_0000121 chr10 50862613 50869838 - 7225 6587 ENSOARG00000015582; exon_1:50869604-50869841,intron_21:50862718-50866368,intron_22:50866458-50868841,exon_3:50868841-50869158,; LDM_E 2 791 TBC1D4

oar_circ_0000122 chr10 51289501 51294905 + 5404 5259 ENSOARG00000015818; intron_5:51289532-51290564,intron_6:51290678-51294706,exon_7:51294706-51294905,; LDM_E 2 828 LMO7

oar_circ_0000123 chr10 51301779 51302782 + 1003 1003 ENSOARG00000015818; intron_8:51300820-51314195,; LDM_E,LDM_A 4,2 266,365 LMO7

oar_circ_0000124 chr10 51301779 51304365 + 2586 2586 ENSOARG00000015818; intron_8:51300820-51314195,; LDM_E,LDM_A 15,56 1379,1514 LMO7

oar_circ_0000125 chr10 51301779 51333572 + 31793 31793 ENSOARG00000015818; exon_17:51333410-51333572,intron_14:51319215-51321590,intron_12:51314313-51317366,intron_8:51300820-51314195,intron_16:51332321-51333410,intron_13:51317509-51318788,intron_15:51321899-51332206,exon_14:51318788-51319215,exon_15:51321590-51321899,; LDM_E,LDM_A 222,263 4225,3051 LMO7

oar_circ_0000126 chr10 51303642 51305411 + 1769 1769 ENSOARG00000015818; intron_8:51300820-51314195,; LDM_A 4 1082 LMO7

oar_circ_0000127 chr10 51303642 51333572 + 29930 29930 ENSOARG00000015818; intron_13:51317509-51318788,intron_16:51332321-51333410,exon_15:51321590-51321899,intron_15:51321899-51332206,exon_14:51318788-51319215,intron_14:51319215-51321590,intron_8:51300820-51314195,intron_12:51314313-51317366,exon_17:51333410-51333572,; LDM_E,LDM_A 10,32 3771,2561 LMO7

oar_circ_0000128 chr10 51314195 51321899 + 7704 7443 ENSOARG00000015818; intron_13:51317509-51318788,exon_15:51321590-51321899,intron_12:51314313-51317366,intron_14:51319215-51321590,exon_14:51318788-51319215,; LDM_E,LDM_A 8,2 881,851 LMO7

oar_circ_0000129 chr10 51314195 51333572 + 19377 19001 ENSOARG00000015818; exon_15:51321590-51321899,exon_14:51318788-51319215,intron_15:51321899-51332206,intron_13:51317509-51318788,intron_16:51332321-51333410,intron_12:51314313-51317366,intron_14:51319215-51321590,exon_17:51333410-51333572,; LDM_A 2 1111 LMO7

oar_circ_0000130 chr10 52707956 52708432 - 476 476 ENSOARG00000016305; intron_32:52697694-52710630,; LDM_A 2 19 MYCBP2

oar_circ_0000131 chr10 52723835 52733575 - 9740 9338 ENSOARG00000016305; exon_41:52733349-52733575,exon_45:52726636-52726809,intron_39:52723980-52726636,intron_43:52731819-52733349,intron_41:52729361-52730538,intron_40:52726809-52729311,intron_42:52730626-52731700,; LDM_E 2 254 MYCBP2

oar_circ_0000132 chr10 54265129 54273188 - 8059 7742 ENSOARG00000000201; intron_3:54268690-54270831,intron_4:54270923-54273052,exon_4:54268441-54268690,intron_2:54265218-54268441,; LDM_E 3 196 RNF219

oar_circ_0000133 chr10 55103485 55116709 + 13224 12996 ENSOARG00000000420; intron_3:55108159-55116615,exon_2:55103485-55103651,intron_2:55103651-55108025,; LDM_A 2 397 NDFIP2

oar_circ_0000134 chr10 55108025 55116709 + 8684 8456 ENSOARG00000000420; intron_3:55108159-55116615,; LDM_E 2 246 NDFIP2

oar_circ_0000135 chr10 55127251 55134798 + 7547 7249 ENSOARG00000000420; intron_6:55132007-55134692,intron_5:55127376-55131940,; LDM_A 3 301 NDFIP2

oar_circ_0000136 chr10 68365109 68423321 + 58212 58212 -- -- LDM_A 6 0 --

oar_circ_0000137 chr10 72354301 72355780 - 1479 1348 ENSOARG00000002096; exon_20:72354301-72354479,intron_2:72354479-72355649,; LDM_E 5 15 DZIP1

oar_circ_0000138 chr10 72533022 72545351 - 12329 9512 ENSOARG00000002377; intron_13:72533831-72536492,intron_14:72536572-72543423,; LDM_A 2 10 UGGT2

oar_circ_0000139 chr10 72620129 72638710 - 18581 18157 ENSOARG00000002377; intron_35:72623562-72628029,intron_36:72628142-72630494,intron_37:72630625-72638627,exon_4:72623387-72623562,intron_34:72620226-72623387,; LDM_E 5 385 UGGT2

oar_circ_0000140 chr10 73809571 73810347 + 776 776 ENSOARG00000002685; exon_1:73809571-73810347,; LDM_E,LDM_A 7,5 631,908 MBNL2

oar_circ_0000141 chr10 74247632 74248262 - 630 630 -- -- LDM_A 2 0 --

oar_circ_0000142 chr10 74382842 74393828 + 10986 9963 ENSOARG00000002835; exon_5:74385459-74385652,intron_10:74391506-74393740,intron_8:74389064-74391177,intron_6:74387545-74388642,intron_4:74382923-74385459,intron_5:74385652-74387442,; LDM_E 2 377 IPO5

oar_circ_0000143 chr10 75456272 75459541 + 3269 2911 ENSOARG00000004044; intron_4:75458898-75459431,intron_3:75456400-75458778,; LDM_E 2 16 UBAC2

oar_circ_0000144 chr10 76297439 76313928 + 16489 16061 ENSOARG00000004442; intron_9:76311505-76313777,intron_7:76297518-76306893,intron_8:76306996-76311410,; LDM_E,LDM_A 2,2 306,89 PCCA

oar_circ_0000145 chr10 76332032 76339591 + 7559 7195 ENSOARG00000004442; intron_12:76333596-76337741,intron_11:76332176-76333521,intron_13:76337810-76339515,; LDM_E 2 282 PCCA

oar_circ_0000146 chr10 76590492 76593499 - 3007 1658 ENSOARG00000004561; intron_2:76590562-76592220,; LDM_A 2 4 TMTC4

oar_circ_0000147 chr10 76625536 76636743 - 11207 11105 ENSOARG00000004561; intron_15:76631404-76632691,exon_1:76636527-76636800,exon_3:76631187-76631404,intron_16:76632762-76636527,intron_14:76625624-76631187,; LDM_E 3 120 TMTC4

oar_circ_0000148 chr10 78241461 78269243 + 27782 26274 ENSOARG00000005010; intron_21:78249623-78250943,exon_28:78264937-78265145,intron_29:78266943-78269181,intron_15:78241920-78243850,intron_18:78247631-78247870,exon_22:78250943-78251188,intron_26:78260633-78261663,intron_28:78265145-78266823,intron_20:78248647-78249485,intron_22:78251523-78259338,exon_17:78244660-78244815,intron_19:78247971-78248550,exon_25:78259338-78259490,intron_25:78259490-78260536,intron_17:78244815-78247514,intron_16:78243957-78244660,intron_22:78251188-78251444,intron_27:78261794-78264937,; LDM_A 12 644 TPP2

oar_circ_0000149 chr10 78374373 78374905 - 532 411 ENSOARG00000005232; exon_6:78374373-78374611,exon_5:78374732-78374905,; LDM_E 2 45 KDELC1

oar_circ_0000150 chr10 78374373 78375716 - 1343 974 ENSOARG00000005232; exon_6:78374373-78374611,exon_3:78375510-78375716,intron_6:78374905-78375262,exon_5:78374732-78374905,; LDM_E 2 74 KDELC1

oar_circ_0000151 chr10 78393129 78393727 + 598 598 ENSOARG00000005295; exon_2:78393129-78393727,; LDM_E,LDM_A 4,4 166,59 -

oar_circ_0000152 chr10 78393129 78397536 + 4407 4280 ENSOARG00000005295; exon_2:78393129-78393727,intron_2:78393727-78397409,; LDM_A 2 72 -

oar_circ_0000153 chr10 78393129 78402423 + 9294 8653 ENSOARG00000005295; intron_2:78393727-78397409,intron_3:78397537-78401379,exon_2:78393129-78393727,intron_4:78401474-78402005,; LDM_A 5 116 -

oar_circ_0000154 chr10 78393129 78410895 + 17766 16992 ENSOARG00000005295; intron_6:78402423-78410762,intron_3:78397537-78401379,intron_2:78393727-78397409,exon_2:78393129-78393727,intron_4:78401474-78402005,; LDM_E 2 449 -

oar_circ_0000155 chr10 81672180 81674781 - 2601 2517 ENSOARG00000005479; intron_2:81672264-81674555,exon_2:81674555-81674781,; LDM_E,LDM_A 8,8 2945,1597 ARGLU1

oar_circ_0000156 chr10 84818109 84831862 + 13753 13225 ENSOARG00000007108; intron_5:84818194-84820854,intron_8:84828193-84828855,intron_7:84824759-84828183,intron_6:84820985-84824708,intron_9:84829009-84830191,intron_10:84830199-84831773,; LDM_A 2 168 ARHGEF7

oar_circ_0000157 chr10 84831773 84849824 + 18051 17178 ENSOARG00000007108; intron_11:84831862-84841231,intron_13:84841892-84849701,; LDM_E,LDM_A 3,9 561,172 ARHGEF7

oar_circ_0000158 chr10 84841231 84849824 + 8593 7809 ENSOARG00000007108; intron_13:84841892-84849701,; LDM_E,LDM_A 2,2 384,100 ARHGEF7

oar_circ_0000159 chr10 85942213 85949076 - 6863 6484 ENSOARG00000008284; exon_47:85942213-85942459,intron_3:85942616-85947435,intron_4:85947532-85948951,; LDM_A 2 31 GRTP1

oar_circ_0000160 chr10 86028337 86034832 + 6495 6297 ENSOARG00000008433; intron_7:86028722-86034636,exon_9:86034636-86034832,exon_7:86028337-86028524,; LDM_A 2 378 TMCO3

oar_circ_0000161 chr10 86031060 86034832 + 3772 3772 ENSOARG00000008433; intron_7:86028722-86034636,exon_9:86034636-86034832,; LDM_A 6 232 TMCO3

oar_circ_0000162 chr11 10172909 10187542 + 14633 14396 ENSOARG00000013249; intron_2:10173117-10175768,exon_8:10184474-10184675,exon_6:10179590-10179764,exon_7:10183562-10183760,intron_9:10186836-10187419,exon_2:10172909-10173117,intron_4:10176859-10178922,intron_3:10176037-10176697,intron_5:10179036-10179590,intron_8:10184675-10186683,exon_3:10175768-10176037,intron_7:10183760-10184474,intron_6:10179764-10183562,exon_9:10186683-10186836,exon_4:10176697-10176859,; LDM_E 2 1205 CLTC

oar_circ_0000163 chr11 10247875 10254769 + 6894 6454 ENSOARG00000014000; intron_3:10252102-10253237,intron_4:10253328-10254658,intron_2:10247977-10251966,; LDM_E,LDM_A 9,3 121,38 VMP1

oar_circ_0000164 chr11 10251966 10291674 + 39708 39238 ENSOARG00000014000; intron_4:10253328-10254658,exon_6:10280503-10280671,intron_5:10254769-10280503,intron_6:10280671-10291542,intron_3:10252102-10253237,; LDM_E 5 484 VMP1

oar_circ_0000165 chr11 10280503 10291674 + 11171 11039 ENSOARG00000014000; exon_6:10280503-10280671,intron_6:10280671-10291542,; LDM_E 2 172 VMP1

oar_circ_0000166 chr11 10614479 10617846 + 3367 3367 ENSOARG00000014972; intron_2:10613227-10631082,; LDM_E 4 599 MED13

oar_circ_0000167 chr11 10631082 10646013 + 14931 14485 ENSOARG00000014972; exon_4:10632075-10632273,intron_7:10634898-10645329,intron_3:10631228-10632075,exon_8:10645329-10646013,intron_4:10632273-10633669,intron_5:10633864-10634435,exon_6:10634435-10634598,exon_5:10633669-10633864,; LDM_E 2 1011 MED13

oar_circ_0000168 chr11 10773128 10773831 + 703 249 ENSOARG00000015532; exon_21:10773128-10773377,; LDM_E,LDM_A 4,4 28,8 INTS2

oar_circ_0000169 chr11 10830555 10842499 + 11944 11811 ENSOARG00000016110; exon_6:10830555-10830846,exon_8:10838827-10839027,exon_7:10836515-10836737,intron_7:10836737-10838827,intron_6:10830846-10836515,intron_8:10839027-10842366,; LDM_E 4 180 BRIP1

oar_circ_0000170 chr11 10830555 10865227 + 34672 34288 ENSOARG00000016110; exon_13:10865065-10865227,exon_6:10830555-10830846,exon_7:10836515-10836737,exon_10:10855203-10855358,intron_10:10855358-10858203,intron_7:10836737-10838827,exon_8:10838827-10839027,intron_9:10842499-10855203,exon_11:10858203-10858369,intron_12:10858620-10865065,intron_8:10839027-10842366,intron_6:10830846-10836515,; LDM_E 4 481 BRIP1

oar_circ_0000171 chr11 10855203 10865227 + 10024 9773 ENSOARG00000016110; intron_10:10855358-10858203,intron_12:10858620-10865065,exon_10:10855203-10855358,exon_11:10858203-10858369,exon_13:10865065-10865227,; LDM_E 2 165 BRIP1

oar_circ_0000172 chr11 11552133 11560883 - 8750 8551 ENSOARG00000016753; exon_21:11558186-11558383,intron_2:11552231-11558186,intron_3:11558383-11560782,; LDM_E 2 62 BCAS3

oar_circ_0000173 chr11 12049018 12055563 + 6545 6332 ENSOARG00000017155; intron_2:12049107-12051309,intron_3:12051461-12055439,exon_3:12051309-12051461,; LDM_E,LDM_A 6,3 809,211 APPBP2

oar_circ_0000174 chr11 12300658 12308146 + 7488 7163 ENSOARG00000017636; intron_9:12302767-12308084,intron_7:12300774-12301303,intron_8:12301366-12302683,; LDM_A 2 230 --

oar_circ_0000175 chr11 13501097 13506699 - 5602 5602 ENSOARG00000002998; exon_4:13503208-13503394,intron_13:13504468-13508175,intron_12:13503394-13504337,intron_11:13501241-13503208,; LDM_E 2 109 DDX52

oar_circ_0000176 chr11 14528017 14529156 - 1139 1139 ENSOARG00000006966; intron_14:14528186-14528988,exon_6:14528988-14529156,exon_7:14528017-14528186,; LDM_A 3 160 UNC45B

oar_circ_0000177 chr11 14678747 14679800 - 1053 167 ENSOARG00000008197; exon_5:14679633-14679800,; LDM_E,LDM_A 4,3 10,1 LIG3

oar_circ_0000178 chr11 17350797 17354532 - 3735 3605 ENSOARG00000010476; intron_9:17350992-17354005,exon_7:17350797-17350992,intron_10:17354063-17354460,; LDM_E,LDM_A 3,5 96,134 PSMD11

oar_circ_0000179 chr11 17354005 17354532 - 527 397 ENSOARG00000010476; intron_10:17354063-17354460,; LDM_A 2 7 PSMD11

oar_circ_0000180 chr11 17565531 17570123 - 4592 4032 ENSOARG00000011384; intron_10:17566349-17568517,intron_11:17568638-17569394,intron_9:17565677-17566264,intron_12:17569503-17570024,; LDM_E 2 137 RHOT1

oar_circ_0000181 chr11 17662191 17663603 - 1412 1219 ENSOARG00000011933; intron_7:17662304-17663523,; LDM_A 2 2 ADAP2

oar_circ_0000182 chr11 18263004 18263922 - 918 408 ENSOARG00000014671; intron_6:18263391-18263799,; LDM_E,LDM_A 12,2 18,3 NF1

oar_circ_0000183 chr11 18339957 18349301 - 9344 8841 ENSOARG00000014671; intron_23:18340068-18344588,intron_25:18347234-18348536,exon_31:18349142-18349301,intron_26:18348634-18349142,intron_24:18344735-18347087,; LDM_E 4 418 NF1

oar_circ_0000184 chr11 18371660 18372882 - 1222 986 ENSOARG00000014671; intron_33:18372173-18372765,exon_26:18371660-18371872,exon_25:18371991-18372173,; LDM_E 2 125 NF1

oar_circ_0000185 chr11 18375468 18405977 - 30509 29763 ENSOARG00000014671; intron_45:18400132-18403617,exon_20:18375468-18375909,exon_16:18385552-18385714,intron_39:18383101-18383321,exon_17:18384164-18384414,intron_43:18387244-18388765,intron_40:18383398-18384164,intron_42:18385714-18387120,intron_44:18388845-18400018,intron_46:18403752-18405845,intron_41:18384414-18385552,intron_38:18375909-18383017,; LDM_E 19 2204 NF1

oar_circ_0000186 chr11 18422353 18441415 - 19062 18583 ENSOARG00000014671; intron_52:18424527-18424758,intron_56:18439457-18441271,intron_55:18434938-18439373,intron_53:18424826-18430628,exon_7:18422353-18422511,intron_54:18430735-18434747,intron_51:18422511-18424451,exon_3:18434747-18434938,; LDM_E 3 1013 NF1

oar_circ_0000187 chr11 20390018 20405068 + 15050 14668 ENSOARG00000006793; intron_7:20390132-20394756,exon_12:20404864-20405068,intron_11:20402780-20404864,intron_10:20399968-20402612,exon_11:20402612-20402780,intron_9:20398286-20399886,intron_8:20394848-20398192,; LDM_E 2 838 TAOK1

oar_circ_0000188 chr11 20525229 20554326 - 29097 28287 ENSOARG00000008802; intron_7:20540452-20544142,intron_10:20549625-20554204,intron_4:20529203-20533292,intron_3:20525430-20529056,intron_9:20548769-20549556,intron_5:20533339-20540093,exon_12:20525229-20525430,exon_7:20544142-20544337,intron_8:20544337-20548703,; LDM_E 3 1935 SSH2

oar_circ_0000189 chr11 20540093 20554326 - 14233 13617 ENSOARG00000008802; exon_7:20544142-20544337,intron_7:20540452-20544142,intron_10:20549625-20554204,intron_8:20544337-20548703,intron_9:20548769-20549556,; LDM_E 2 931 SSH2

oar_circ_0000190 chr11 20899504 20901668 + 2164 1926 ENSOARG00000009006; exon_5:20900495-20900703,intron_5:20900703-20901559,intron_4:20899633-20900495,; LDM_E 2 62 NSRP1

oar_circ_0000191 chr11 20991606 20993300 - 1694 1512 ENSOARG00000010429; intron_7:20991699-20993211,; LDM_E 2 38 BLMH

oar_circ_0000192 chr11 21123234 21124906 + 1672 1672 ENSOARG00000010615; intron_5:21123404-21124556,exon_5:21123234-21123404,exon_6:21124556-21124906,; LDM_A 2 78 CPD

oar_circ_0000193 chr11 21125830 21138961 + 13131 12772 ENSOARG00000010615; intron_8:21128336-21132600,exon_8:21128168-21128336,intron_7:21126022-21128168,intron_10:21138332-21138818,exon_7:21125830-21126022,intron_9:21132710-21138226,; LDM_E 2 187 CPD

oar_circ_0000194 chr11 22185159 22185321 - 162 162 -- -- LDM_E 3 0 --

oar_circ_0000195 chr11 22340438 22343639 - 3201 3068 ENSOARG00000013025; intron_10:22340515-22341620,intron_11:22341676-22343431,exon_2:22343431-22343639,; LDM_E,LDM_A 2,8 32,73 SLC43A2

oar_circ_0000196 chr11 22889834 22890535 - 701 590 ENSOARG00000015076; intron_12:22890047-22890424,exon_10:22889834-22890047,; LDM_A 4 16 SMG6

oar_circ_0000197 chr11 22916471 22918691 + 2220 1989 ENSOARG00000015317; intron_2:22916643-22918275,intron_3:22918402-22918587,exon_2:22916471-22916643,; LDM_E 3 56 SRR

oar_circ_0000198 chr11 23165336 23185164 + 19828 19447 ENSOARG00000016011; exon_4:23184957-23185164,intron_3:23184064-23184957,intron_2:23183707-23183989,intron_1:23165557-23183622,; LDM_E 3 1939 PAFAH1B1

oar_circ_0000199 chr11 24416165 24429549 - 13384 13060 ENSOARG00000019021; intron_2:24419202-24422180,exon_3:24419023-24419202,intron_1:24416288-24419023,intron_3:24422278-24429446,; LDM_E,LDM_A 3,8 1586,1690 UBE2G1

oar_circ_0000200 chr11 24865096 24880151 + 15055 14885 ENSOARG00000001510; intron_3:24875246-24877288,exon_6:24879940-24880151,intron_4:24877351-24877761,exon_2:24872718-24873343,intron_1:24865257-24872718,exon_5:24877761-24877977,exon_1:24865096-24865257,intron_5:24877977-24879940,intron_2:24873343-24875139,; LDM_E 5 432 KIAA0753

oar_circ_0000201 chr11 24865100 24880155 + 15055 14724 ENSOARG00000001510; intron_4:24877351-24877761,exon_6:24879940-24880151,intron_1:24865257-24872718,exon_2:24872718-24873343,intron_3:24875246-24877288,intron_2:24873343-24875139,intron_5:24877977-24879940,exon_5:24877761-24877977,; LDM_E 3 430 KIAA0753

oar_circ_0000202 chr11 24902534 24904567 + 2033 1765 ENSOARG00000001510; intron_13:24902720-24904485,; LDM_A 3 5 KIAA0753

oar_circ_0000203 chr11 25795439 25797265 - 1826 1721 ENSOARG00000003979; exon_9:25796797-25797265,intron_9:25795544-25796797,; LDM_A 2 302 RABEP1

oar_circ_0000204 chr11 25802922 25820329 - 17407 17019 ENSOARG00000003979; intron_11:25803054-25805322,exon_3:25820125-25820329,exon_7:25805322-25805501,intron_12:25805501-25807883,intron_14:25814804-25818212,exon_4:25818212-25818373,intron_13:25808019-25814684,intron_15:25818373-25820125,; LDM_A 2 736 RABEP1

oar_circ_0000205 chr11 25802922 25835196 - 32274 31757 ENSOARG00000003979; intron_16:25820329-25835067,exon_7:25805322-25805501,intron_11:25803054-25805322,intron_14:25814804-25818212,intron_12:25805501-25807883,exon_3:25820125-25820329,exon_4:25818212-25818373,intron_15:25818373-25820125,intron_13:25808019-25814684,; LDM_E 5 1162 RABEP1

oar_circ_0000206 chr11 25814684 25820329 - 5645 5525 ENSOARG00000003979; exon_4:25818212-25818373,intron_14:25814804-25818212,intron_15:25818373-25820125,exon_3:25820125-25820329,; LDM_E,LDM_A 3,5 227,187 RABEP1

oar_circ_0000207 chr11 27176596 27177276 + 680 533 ENSOARG00000018387; intron_8:27176833-27177129,exon_8:27176596-27176833,; LDM_E,LDM_A 3,3 8,5 CNTROB

oar_circ_0000208 chr11 27343390 27343898 - 508 377 ENSOARG00000018911; exon_16:27343390-27343570,intron_8:27343570-27343767,; LDM_A 3 25 PER1

oar_circ_0000209 chr11 27680233 27704335 - 24102 23503 ENSOARG00000001617; intron_32:27680329-27680575,intron_35:27684247-27697443,intron_34:27682414-27684183,intron_36:27697536-27704232,intron_33:27680719-27682315,; LDM_A 3 1551 MYH10

oar_circ_0000210 chr11 28516573 28518823 - 2250 2157 ENSOARG00000003306; intron_3:28516666-28518612,exon_3:28518612-28518823,; LDM_E 2 67 DHRS7C

oar_circ_0000211 chr11 28516573 28520586 - 4013 3807 ENSOARG00000003306; exon_3:28518612-28518823,intron_3:28516666-28518612,intron_4:28518823-28520473,; LDM_A 2 410 DHRS7C

oar_circ_0000212 chr11 28953873 28954116 - 243 150 ENSOARG00000007268; exon_40:28953966-28954116,; LDM_E 3 23 MYH8

oar_circ_0000213 chr11 28955437 29031410 - 75973 24751 ENSOARG00000011486;ENSOARG00000009609;ENSOARG00000007268; exon_39:29027984-29031410,;intron_36:29022216-29022953,exon_20:29012115-29012374,intron_35:29019718-29022188,exon_22:29010683-29010860,intron_24:29013475-29014046,exon_21:29011169-29011412,exon_36:29003226-29003446,exon_14:29014953-29015260,exon_3:29022953-29023110,exon_34:29004836-29005040,exon_1:29023460-29023664,intron_32:29018596-29019230,intron_19:29011412-29012115,intron_25:29014129-29014953,intron_26:29015260-29016037,intron_1:29001845-29002846,exon_33:29005674-29005983,exon_25:29009563-29009888,exon_29:29008072-29008269,intron_30:29018148-29018300,;exon_21:28964315-28964558,exon_31:28960076-28960242,exon_20:28966276-28966532,intron_23:28968778-28969126,exon_29:28961638-28961835,intron_5:28956713-28957135,intron_11:28960512-28961638,exon_30:28960328-28960512,intron_14:28962533-28963196,exon_33:28958670-28958979,intron_25:28969418-28971005,exon_34:28957843-28958041,exon_14:28971180-28971490,intron_6:28957138-28957843,intron_37:28977734-28979828,exon_1:28980887-28981097,exon_26:28963196-28963367,exon_37:28956333-28956504,exon_25:28963369-28963567,intron_30:28974479-28975598,exon_22:28964048-28964225,; LDM_A 16 11952 -;--;MYH8

oar_circ_0000214 chr11 28956587 29003397 - 46810 11904 ENSOARG00000007268;ENSOARG00000009609; exon_34:28957843-28958041,intron_25:28969418-28971005,exon_30:28960328-28960512,intron_14:28962533-28963196,exon_33:28958670-28958979,intron_11:28960512-28961638,intron_5:28956713-28957135,exon_29:28961638-28961835,exon_21:28964315-28964558,exon_20:28966276-28966532,exon_31:28960076-28960242,intron_23:28968778-28969126,exon_22:28964048-28964225,intron_30:28974479-28975598,exon_26:28963196-28963367,exon_25:28963369-28963567,exon_1:28980887-28981097,intron_37:28977734-28979828,exon_14:28971180-28971490,intron_6:28957138-28957843,;intron_1:29001845-29002846,exon_36:29003226-29003446,; LDM_E,LDM_A 1351,7 36535,3709 MYH8;--

oar_circ_0000215 chr11 28956587 29034125 - 77538 36812 ENSOARG00000011486;ENSOARG00000007268;ENSOARG00000009609; intron_1:29031410-29033367,exon_39:29027984-29031410,exon_36:29033954-29034125,;exon_34:28957843-28958041,intron_25:28969418-28971005,exon_20:28966276-28966532,intron_27:28971490-28972361,intron_30:28974479-28975598,intron_11:28960512-28961638,exon_31:28960076-28960242,exon_12:28974133-28974283,exon_13:28972361-28972532,intron_13:28962078-28962406,exon_33:28958670-28958979,intron_37:28977734-28979828,intron_21:28966532-28968414,exon_3:28979828-28979985,exon_29:28961638-28961835,intron_39:28980214-28980887,intron_7:28958041-28958670,intron_33:28976187-28977119,intron_8:28958979-28959864,intron_5:28956713-28957135,exon_26:28963196-28963367,intron_6:28957138-28957843,intron_23:28968778-28969126,exon_14:28971180-28971490,exon_25:28963369-28963567,exon_1:28980887-28981097,intron_31:28975737-28975893,exon_21:28964315-28964558,exon_22:28964048-28964225,intron_14:28962533-28963196,intron_28:28972532-28974133,exon_30:28960328-28960512,intron_20:28964558-28966276,;intron_25:29014129-29014953,exon_3:29022953-29023110,exon_14:29014953-29015260,intron_5:29003590-29004836,exon_13:29016037-29016208,intron_10:29006908-29008072,exon_21:29011169-29011412,exon_30:29006724-29006908,exon_25:29009563-29009888,exon_31:29006472-29006638,intron_35:29019718-29022188,intron_36:29022216-29022953,exon_36:29003226-29003446,intron_24:29013475-29014046,exon_1:29023460-29023664,intron_26:29015260-29016037,exon_33:29005674-29005983,intron_22:29012841-29013188,; LDM_E 1417 37954 -;MYH8;--

oar_circ_0000216 chr11 28958670 29034865 - 76195 35062 ENSOARG00000007268;ENSOARG00000011486;ENSOARG00000009609; exon_14:28971180-28971490,exon_1:28980887-28981097,exon_25:28963369-28963567,intron_25:28969418-28971005,intron_23:28968778-28969126,exon_22:28964048-28964225,exon_31:28960076-28960242,exon_12:28974133-28974283,intron_31:28975737-28975893,exon_20:28966276-28966532,intron_27:28971490-28972361,exon_21:28964315-28964558,intron_30:28974479-28975598,intron_11:28960512-28961638,intron_28:28972532-28974133,intron_21:28966532-28968414,intron_37:28977734-28979828,exon_3:28979828-28979985,exon_29:28961638-28961835,exon_13:28972361-28972532,intron_14:28962533-28963196,intron_13:28962078-28962406,exon_33:28958670-28958979,exon_30:28960328-28960512,exon_26:28963196-28963367,intron_20:28964558-28966276,intron_39:28980214-28980887,intron_33:28976187-28977119,intron_8:28958979-28959864,;exon_36:29033954-29034125,exon_39:29027984-29031410,exon_34:29034661-29034865,intron_1:29031410-29033367,;exon_21:29011169-29011412,intron_10:29006908-29008072,intron_25:29014129-29014953,exon_13:29016037-29016208,intron_5:29003590-29004836,exon_3:29022953-29023110,exon_14:29014953-29015260,exon_1:29023460-29023664,intron_24:29013475-29014046,intron_22:29012841-29013188,exon_33:29005674-29005983,intron_26:29015260-29016037,exon_31:29006472-29006638,exon_30:29006724-29006908,exon_25:29009563-29009888,exon_36:29003226-29003446,intron_36:29022216-29022953,intron_35:29019718-29022188,; LDM_E 792 36662 MYH8;-;--

oar_circ_0000217 chr11 28959864 29035260 - 75396 26183 ENSOARG00000007268;ENSOARG00000011486;ENSOARG00000009609; exon_14:28971180-28971490,intron_37:28977734-28979828,exon_1:28980887-28981097,exon_25:28963369-28963567,exon_26:28963196-28963367,intron_30:28974479-28975598,exon_22:28964048-28964225,exon_31:28960076-28960242,exon_20:28966276-28966532,intron_23:28968778-28969126,exon_21:28964315-28964558,exon_29:28961638-28961835,intron_11:28960512-28961638,exon_30:28960328-28960512,intron_14:28962533-28963196,intron_25:28969418-28971005,;exon_36:29033954-29034125,exon_34:29034661-29034865,intron_3:29033686-29033954,intron_5:29034333-29034661,intron_1:29031410-29033367,exon_39:29027984-29031410,exon_33:29034951-29035260,;intron_36:29022216-29022953,intron_35:29019718-29022188,exon_20:29012115-29012374,exon_22:29010683-29010860,exon_21:29011169-29011412,intron_24:29013475-29014046,exon_36:29003226-29003446,exon_3:29022953-29023110,exon_14:29014953-29015260,exon_1:29023460-29023664,exon_34:29004836-29005040,intron_32:29018596-29019230,intron_19:29011412-29012115,intron_25:29014129-29014953,intron_1:29001845-29002846,intron_26:29015260-29016037,exon_33:29005674-29005983,exon_25:29009563-29009888,exon_29:29008072-29008269,intron_30:29018148-29018300,; LDM_E,LDM_A 285,531 30021,38501 MYH8;-;--

oar_circ_0000218 chr11 28961638 29006908 - 45270 18337 ENSOARG00000009609;ENSOARG00000007268; exon_36:29003226-29003446,intron_5:29003590-29004836,exon_33:29005674-29005983,exon_31:29006472-29006638,exon_30:29006724-29006908,;exon_14:28971180-28971490,exon_25:28963369-28963567,exon_1:28980887-28981097,intron_25:28969418-28971005,intron_23:28968778-28969126,exon_22:28964048-28964225,exon_12:28974133-28974283,intron_31:28975737-28975893,intron_27:28971490-28972361,exon_20:28966276-28966532,exon_21:28964315-28964558,intron_30:28974479-28975598,intron_28:28972532-28974133,intron_21:28966532-28968414,intron_37:28977734-28979828,exon_3:28979828-28979985,exon_29:28961638-28961835,exon_13:28972361-28972532,intron_14:28962533-28963196,intron_13:28962078-28962406,exon_26:28963196-28963367,intron_20:28964558-28966276,intron_39:28980214-28980887,intron_33:28976187-28977119,; LDM_E 1610 26874 --;MYH8

oar_circ_0000219 chr11 28961959 29037415 - 75456 34447 ENSOARG00000009609;ENSOARG00000007268;ENSOARG00000011486; exon_14:29014953-29015260,exon_3:29022953-29023110,intron_5:29003590-29004836,exon_13:29016037-29016208,intron_25:29014129-29014953,exon_21:29011169-29011412,intron_10:29006908-29008072,intron_36:29022216-29022953,exon_36:29003226-29003446,intron_35:29019718-29022188,exon_31:29006472-29006638,exon_30:29006724-29006908,exon_25:29009563-29009888,exon_33:29005674-29005983,intron_22:29012841-29013188,intron_26:29015260-29016037,exon_1:29023460-29023664,intron_24:29013475-29014046,;intron_39:28980214-28980887,intron_33:28976187-28977119,intron_20:28964558-28966276,exon_26:28963196-28963367,intron_14:28962533-28963196,intron_13:28962078-28962406,exon_13:28972361-28972532,intron_28:28972532-28974133,intron_21:28966532-28968414,exon_3:28979828-28979985,intron_37:28977734-28979828,intron_30:28974479-28975598,intron_31:28975737-28975893,exon_21:28964315-28964558,intron_27:28971490-28972361,exon_20:28966276-28966532,exon_22:28964048-28964225,exon_12:28974133-28974283,intron_23:28968778-28969126,exon_14:28971180-28971490,intron_25:28969418-28971005,exon_1:28980887-28981097,exon_25:28963369-28963567,;exon_39:29027984-29031410,exon_33:29034951-29035260,exon_29:29037218-29037415,exon_34:29034661-29034865,exon_30:29036308-29036492,intron_7:29035260-29035653,exon_31:29035865-29036031,exon_36:29033954-29034125,intron_9:29036031-29036308,intron_10:29036492-29037218,intron_1:29031410-29033367,; LDM_E 116 26738 --;MYH8;-

oar_circ_0000220 chr11 28963650 29009888 - 46238 18269 ENSOARG00000007268;ENSOARG00000009609; intron_31:28975737-28975893,intron_27:28971490-28972361,exon_20:28966276-28966532,exon_21:28964315-28964558,intron_30:28974479-28975598,exon_22:28964048-28964225,exon_12:28974133-28974283,intron_23:28968778-28969126,exon_14:28971180-28971490,exon_1:28980887-28981097,intron_25:28969418-28971005,intron_39:28980214-28980887,intron_33:28976187-28977119,intron_20:28964558-28966276,exon_13:28972361-28972532,intron_28:28972532-28974133,exon_3:28979828-28979985,intron_37:28977734-28979828,intron_21:28966532-28968414,;exon_36:29003226-29003446,intron_5:29003590-29004836,exon_33:29005674-29005983,intron_10:29006908-29008072,exon_31:29006472-29006638,exon_25:29009563-29009888,exon_30:29006724-29006908,; LDM_E 1043 24963 MYH8;--

oar_circ_0000221 chr11 28963650 29040575 - 76925 35839 ENSOARG00000007268;ENSOARG00000011486;ENSOARG00000009609; exon_22:28964048-28964225,exon_12:28974133-28974283,intron_31:28975737-28975893,intron_27:28971490-28972361,exon_20:28966276-28966532,exon_21:28964315-28964558,intron_30:28974479-28975598,exon_14:28971180-28971490,exon_1:28980887-28981097,intron_25:28969418-28971005,intron_23:28968778-28969126,intron_20:28964558-28966276,intron_39:28980214-28980887,intron_33:28976187-28977119,intron_28:28972532-28974133,exon_3:28979828-28979985,intron_37:28977734-28979828,intron_21:28966532-28968414,exon_13:28972361-28972532,;intron_13:29039598-29040197,intron_10:29036492-29037218,intron_1:29031410-29033367,intron_7:29035260-29035653,exon_30:29036308-29036492,exon_31:29035865-29036031,exon_36:29033954-29034125,intron_9:29036031-29036308,exon_39:29027984-29031410,exon_33:29034951-29035260,exon_26:29040197-29040575,exon_34:29034661-29034865,exon_29:29037218-29037415,intron_12:29037696-29039471,;intron_10:29006908-29008072,exon_21:29011169-29011412,exon_14:29014953-29015260,exon_3:29022953-29023110,exon_13:29016037-29016208,intron_5:29003590-29004836,intron_25:29014129-29014953,intron_26:29015260-29016037,exon_33:29005674-29005983,intron_22:29012841-29013188,intron_24:29013475-29014046,exon_1:29023460-29023664,intron_35:29019718-29022188,intron_36:29022216-29022953,exon_36:29003226-29003446,exon_25:29009563-29009888,exon_30:29006724-29006908,exon_31:29006472-29006638,; LDM_E 972 26281 MYH8;-;--

oar_circ_0000222 chr11 28964315 29010860 - 46545 18092 ENSOARG00000007268;ENSOARG00000009609; intron_30:28974479-28975598,intron_31:28975737-28975893,intron_27:28971490-28972361,exon_21:28964315-28964558,exon_20:28966276-28966532,exon_12:28974133-28974283,intron_23:28968778-28969126,intron_25:28969418-28971005,exon_1:28980887-28981097,exon_14:28971180-28971490,intron_33:28976187-28977119,intron_39:28980214-28980887,intron_20:28964558-28966276,exon_13:28972361-28972532,intron_21:28966532-28968414,intron_37:28977734-28979828,exon_3:28979828-28979985,intron_28:28972532-28974133,;exon_33:29005674-29005983,exon_36:29003226-29003446,intron_5:29003590-29004836,intron_10:29006908-29008072,exon_31:29006472-29006638,exon_25:29009563-29009888,exon_30:29006724-29006908,; LDM_E 226 23758 MYH8;--

oar_circ_0000223 chr11 28964315 29041404 - 77089 35839 ENSOARG00000007268;ENSOARG00000011486;ENSOARG00000009609; intron_33:28976187-28977119,intron_39:28980214-28980887,intron_20:28964558-28966276,exon_13:28972361-28972532,intron_37:28977734-28979828,exon_3:28979828-28979985,intron_21:28966532-28968414,intron_28:28972532-28974133,intron_31:28975737-28975893,intron_27:28971490-28972361,exon_20:28966276-28966532,exon_21:28964315-28964558,intron_30:28974479-28975598,exon_12:28974133-28974283,intron_23:28968778-28969126,exon_1:28980887-28981097,intron_25:28969418-28971005,exon_14:28971180-28971490,;intron_12:29037696-29039471,exon_33:29034951-29035260,exon_39:29027984-29031410,exon_29:29037218-29037415,exon_34:29034661-29034865,exon_26:29040197-29040575,exon_31:29035865-29036031,exon_30:29036308-29036492,intron_7:29035260-29035653,intron_9:29036031-29036308,exon_36:29033954-29034125,exon_23:29041227-29041404,intron_13:29039598-29040197,intron_1:29031410-29033367,intron_10:29036492-29037218,;exon_21:29011169-29011412,intron_10:29006908-29008072,intron_5:29003590-29004836,exon_13:29016037-29016208,exon_14:29014953-29015260,exon_3:29022953-29023110,intron_25:29014129-29014953,intron_26:29015260-29016037,intron_22:29012841-29013188,exon_33:29005674-29005983,intron_24:29013475-29014046,exon_1:29023460-29023664,intron_35:29019718-29022188,exon_36:29003226-29003446,intron_36:29022216-29022953,exon_25:29009563-29009888,exon_30:29006724-29006908,exon_31:29006472-29006638,; LDM_E 257 25098 MYH8;-;--

oar_circ_0000224 chr11 28964348 29011202 - 46854 18092 ENSOARG00000007268;ENSOARG00000009609; exon_12:28974133-28974283,intron_31:28975737-28975893,exon_21:28964315-28964558,intron_27:28971490-28972361,exon_20:28966276-28966532,intron_30:28974479-28975598,exon_14:28971180-28971490,exon_1:28980887-28981097,intron_25:28969418-28971005,intron_23:28968778-28969126,intron_20:28964558-28966276,intron_39:28980214-28980887,intron_33:28976187-28977119,intron_28:28972532-28974133,intron_37:28977734-28979828,exon_3:28979828-28979985,intron_21:28966532-28968414,exon_13:28972361-28972532,;exon_33:29005674-29005983,exon_36:29003226-29003446,intron_5:29003590-29004836,intron_10:29006908-29008072,exon_31:29006472-29006638,exon_25:29009563-29009888,exon_30:29006724-29006908,; LDM_E 30 22255 MYH8;--

oar_circ_0000225 chr11 28964348 29041529 - 77181 35839 ENSOARG00000009609;ENSOARG00000007268;ENSOARG00000011486; intron_25:29014129-29014953,exon_3:29022953-29023110,exon_14:29014953-29015260,intron_5:29003590-29004836,exon_13:29016037-29016208,exon_21:29011169-29011412,intron_10:29006908-29008072,exon_31:29006472-29006638,exon_25:29009563-29009888,exon_30:29006724-29006908,intron_36:29022216-29022953,exon_36:29003226-29003446,intron_35:29019718-29022188,exon_1:29023460-29023664,intron_24:29013475-29014046,exon_33:29005674-29005983,intron_22:29012841-29013188,intron_26:29015260-29016037,;intron_30:28974479-28975598,intron_31:28975737-28975893,exon_20:28966276-28966532,intron_27:28971490-28972361,exon_21:28964315-28964558,exon_12:28974133-28974283,intron_23:28968778-28969126,exon_14:28971180-28971490,intron_25:28969418-28971005,exon_1:28980887-28981097,intron_39:28980214-28980887,intron_33:28976187-28977119,intron_20:28964558-28966276,exon_13:28972361-28972532,intron_28:28972532-28974133,intron_37:28977734-28979828,exon_3:28979828-28979985,intron_21:28966532-28968414,;intron_13:29039598-29040197,exon_23:29041227-29041404,intron_1:29031410-29033367,intron_10:29036492-29037218,exon_30:29036308-29036492,intron_7:29035260-29035653,exon_31:29035865-29036031,exon_36:29033954-29034125,intron_9:29036031-29036308,exon_39:29027984-29031410,exon_33:29034951-29035260,exon_26:29040197-29040575,exon_34:29034661-29034865,exon_29:29037218-29037415,intron_12:29037696-29039471,; LDM_E 24 23595 --;MYH8;-

oar_circ_0000226 chr11 28964369 29011223 + 46854 46854 -- -- LDM_E 40 0 --

oar_circ_0000227 chr11 28966276 29041739 - 75463 34121 ENSOARG00000011486;ENSOARG00000007268;ENSOARG00000009609; exon_39:29027984-29031410,exon_26:29040197-29040575,exon_34:29034661-29034865,intron_12:29037696-29039471,intron_13:29039598-29040197,intron_10:29036492-29037218,intron_1:29031410-29033367,intron_7:29035260-29035653,exon_31:29035865-29036031,intron_9:29036031-29036308,exon_33:29034951-29035260,exon_29:29037218-29037415,exon_22:29041496-29041739,exon_23:29041227-29041404,exon_30:29036308-29036492,exon_36:29033954-29034125,;intron_23:28968778-28969126,exon_14:28971180-28971490,exon_1:28980887-28981097,intron_25:28969418-28971005,intron_31:28975737-28975893,intron_27:28971490-28972361,exon_20:28966276-28966532,intron_30:28974479-28975598,exon_12:28974133-28974283,exon_13:28972361-28972532,intron_28:28972532-28974133,exon_3:28979828-28979985,intron_21:28966532-28968414,intron_37:28977734-28979828,intron_39:28980214-28980887,intron_33:28976187-28977119,;intron_26:29015260-29016037,intron_22:29012841-29013188,exon_33:29005674-29005983,intron_24:29013475-29014046,exon_1:29023460-29023664,intron_35:29019718-29022188,exon_36:29003226-29003446,intron_36:29022216-29022953,exon_30:29006724-29006908,exon_25:29009563-29009888,exon_31:29006472-29006638,exon_21:29011169-29011412,intron_10:29006908-29008072,intron_5:29003590-29004836,exon_13:29016037-29016208,exon_3:29022953-29023110,exon_14:29014953-29015260,intron_25:29014129-29014953,; LDM_E 107 20624 -;MYH8;--

oar_circ_0000228 chr11 28972361 29015260 - 42899 8763 ENSOARG00000007268;ENSOARG00000009609; intron_37:28977734-28979828,intron_30:28974479-28975598,exon_1:28980887-28981097,;exon_20:29012115-29012374,exon_14:29014953-29015260,exon_34:29004836-29005040,exon_22:29010683-29010860,intron_19:29011412-29012115,intron_24:29013475-29014046,intron_25:29014129-29014953,exon_21:29011169-29011412,intron_1:29001845-29002846,exon_33:29005674-29005983,exon_25:29009563-29009888,exon_36:29003226-29003446,exon_29:29008072-29008269,; LDM_A 302 3252 MYH8;--

oar_circ_0000229 chr11 28972361 29046396 - 74035 29843 ENSOARG00000011486;ENSOARG00000009609;ENSOARG00000007268; intron_3:29033686-29033954,intron_7:29035260-29035653,intron_23:29044855-29045923,exon_33:29034951-29035260,exon_31:29035865-29036031,exon_22:29041496-29041739,intron_18:29041739-29043571,exon_21:29043571-29043827,intron_1:29031410-29033367,intron_13:29039598-29040197,intron_9:29036031-29036308,exon_23:29041227-29041404,exon_34:29034661-29034865,intron_12:29037696-29039471,exon_30:29036308-29036492,exon_15:29046086-29046396,intron_15:29040757-29041003,intron_5:29034333-29034661,intron_10:29036492-29037218,intron_21:29044271-29044568,exon_39:29027984-29031410,intron_11:29037415-29037577,exon_29:29037218-29037415,exon_36:29033954-29034125,exon_26:29040197-29040575,;exon_34:29004836-29005040,exon_1:29023460-29023664,exon_14:29014953-29015260,exon_3:29022953-29023110,intron_25:29014129-29014953,intron_19:29011412-29012115,intron_32:29018596-29019230,intron_26:29015260-29016037,intron_1:29001845-29002846,exon_33:29005674-29005983,exon_29:29008072-29008269,exon_25:29009563-29009888,intron_30:29018148-29018300,intron_35:29019718-29022188,exon_20:29012115-29012374,intron_36:29022216-29022953,exon_22:29010683-29010860,exon_21:29011169-29011412,intron_24:29013475-29014046,exon_36:29003226-29003446,;exon_1:28980887-28981097,intron_30:28974479-28975598,intron_37:28977734-28979828,; LDM_A 463 85174 -;--;MYH8

oar_circ_0000230 chr11 28974133 29016208 - 42075 12345 ENSOARG00000007268;ENSOARG00000009609; exon_12:28974133-28974283,intron_39:28980214-28980887,intron_33:28976187-28977119,intron_31:28975737-28975893,intron_30:28974479-28975598,exon_1:28980887-28981097,intron_37:28977734-28979828,exon_3:28979828-28979985,;intron_24:29013475-29014046,intron_22:29012841-29013188,exon_33:29005674-29005983,exon_21:29011169-29011412,intron_26:29015260-29016037,intron_10:29006908-29008072,exon_31:29006472-29006638,intron_25:29014129-29014953,exon_30:29006724-29006908,exon_25:29009563-29009888,exon_13:29016037-29016208,intron_5:29003590-29004836,exon_36:29003226-29003446,exon_14:29014953-29015260,; LDM_E 303 8307 MYH8;--

oar_circ_0000231 chr11 28974133 29047343 - 73210 31508 ENSOARG00000009609;ENSOARG00000011486;ENSOARG00000007268; intron_25:29014129-29014953,exon_3:29022953-29023110,exon_14:29014953-29015260,exon_13:29016037-29016208,intron_5:29003590-29004836,intron_10:29006908-29008072,exon_21:29011169-29011412,exon_31:29006472-29006638,exon_30:29006724-29006908,exon_25:29009563-29009888,intron_36:29022216-29022953,exon_36:29003226-29003446,intron_35:29019718-29022188,exon_1:29023460-29023664,intron_24:29013475-29014046,exon_33:29005674-29005983,intron_22:29012841-29013188,intron_26:29015260-29016037,;intron_25:29046396-29047172,intron_10:29036492-29037218,intron_1:29031410-29033367,intron_13:29039598-29040197,intron_9:29036031-29036308,intron_7:29035260-29035653,exon_31:29035865-29036031,exon_26:29040197-29040575,exon_34:29034661-29034865,exon_39:29027984-29031410,intron_18:29041739-29043571,intron_12:29037696-29039471,exon_15:29046086-29046396,exon_21:29043571-29043827,exon_23:29041227-29041404,exon_36:29033954-29034125,exon_30:29036308-29036492,exon_14:29047172-29047343,exon_29:29037218-29037415,intron_23:29044855-29045923,exon_33:29034951-29035260,exon_22:29041496-29041739,;intron_31:28975737-28975893,intron_33:28976187-28977119,intron_30:28974479-28975598,intron_39:28980214-28980887,exon_12:28974133-28974283,exon_3:28979828-28979985,exon_1:28980887-28981097,intron_37:28977734-28979828,; LDM_E 355 9705 --;-;MYH8

oar_circ_0000232 chr11 28975598 29048803 - 73205 30751 ENSOARG00000009609;ENSOARG00000011486;ENSOARG00000007268; exon_33:29005674-29005983,intron_30:29018148-29018300,exon_29:29008072-29008269,exon_25:29009563-29009888,exon_34:29004836-29005040,exon_1:29023460-29023664,exon_3:29022953-29023110,exon_14:29014953-29015260,intron_1:29001845-29002846,intron_26:29015260-29016037,intron_25:29014129-29014953,intron_32:29018596-29019230,intron_19:29011412-29012115,exon_36:29003226-29003446,exon_22:29010683-29010860,exon_20:29012115-29012374,intron_35:29019718-29022188,intron_36:29022216-29022953,intron_24:29013475-29014046,exon_21:29011169-29011412,;exon_21:29043571-29043827,intron_1:29031410-29033367,exon_15:29046086-29046396,intron_12:29037696-29039471,exon_13:29048444-29048594,exon_30:29036308-29036492,exon_23:29041227-29041404,exon_34:29034661-29034865,intron_9:29036031-29036308,intron_13:29039598-29040197,exon_31:29035865-29036031,exon_33:29034951-29035260,intron_23:29044855-29045923,intron_7:29035260-29035653,intron_3:29033686-29033954,intron_18:29041739-29043571,exon_22:29041496-29041739,exon_29:29037218-29037415,exon_39:29027984-29031410,intron_11:29037415-29037577,exon_26:29040197-29040575,intron_26:29047343-29048444,exon_36:29033954-29034125,intron_25:29046396-29047172,intron_5:29034333-29034661,intron_15:29040757-29041003,intron_21:29044271-29044568,intron_10:29036492-29037218,;intron_37:28977734-28979828,exon_1:28980887-28981097,; LDM_A 2770 85348 --;-;MYH8

oar_circ_0000233 chr11 28977119 29049586 - 72467 31099 ENSOARG00000007268;ENSOARG00000011486;ENSOARG00000009609; intron_37:28977734-28979828,exon_1:28980887-28981097,;intron_10:29036492-29037218,intron_21:29044271-29044568,intron_29:29049138-29049290,intron_5:29034333-29034661,intron_15:29040757-29041003,intron_25:29046396-29047172,intron_26:29047343-29048444,exon_36:29033954-29034125,exon_26:29040197-29040575,intron_28:29048803-29048999,intron_11:29037415-29037577,exon_39:29027984-29031410,exon_29:29037218-29037415,intron_18:29041739-29043571,exon_22:29041496-29041739,intron_23:29044855-29045923,exon_31:29035865-29036031,exon_33:29034951-29035260,intron_3:29033686-29033954,intron_7:29035260-29035653,intron_9:29036031-29036308,intron_13:29039598-29040197,intron_12:29037696-29039471,exon_34:29034661-29034865,exon_13:29048444-29048594,exon_23:29041227-29041404,exon_15:29046086-29046396,exon_30:29036308-29036492,intron_1:29031410-29033367,exon_21:29043571-29043827,;exon_33:29005674-29005983,intron_30:29018148-29018300,exon_29:29008072-29008269,exon_25:29009563-29009888,exon_1:29023460-29023664,exon_34:29004836-29005040,exon_3:29022953-29023110,exon_14:29014953-29015260,intron_26:29015260-29016037,intron_1:29001845-29002846,intron_25:29014129-29014953,intron_19:29011412-29012115,intron_32:29018596-29019230,exon_36:29003226-29003446,exon_22:29010683-29010860,intron_35:29019718-29022188,exon_20:29012115-29012374,intron_36:29022216-29022953,exon_21:29011169-29011412,intron_24:29013475-29014046,; LDM_A 64 85359 MYH8;-;--

oar_circ_0000234 chr11 28977313 29019294 - 41981 9988 ENSOARG00000009609;ENSOARG00000007268; intron_24:29013475-29014046,intron_26:29015260-29016037,exon_21:29011169-29011412,intron_10:29006908-29008072,intron_22:29012841-29013188,exon_33:29005674-29005983,exon_25:29009563-29009888,exon_30:29006724-29006908,intron_25:29014129-29014953,exon_31:29006472-29006638,exon_36:29003226-29003446,exon_13:29016037-29016208,intron_5:29003590-29004836,exon_14:29014953-29015260,;intron_39:28980214-28980887,exon_1:28980887-28981097,exon_3:28979828-28979985,intron_37:28977734-28979828,; LDM_E 65 7642 --;MYH8

oar_circ_0000235 chr11 28977498 29019517 - 42019 9988 ENSOARG00000007268;ENSOARG00000009609; intron_39:28980214-28980887,exon_3:28979828-28979985,exon_1:28980887-28981097,intron_37:28977734-28979828,;exon_21:29011169-29011412,intron_10:29006908-29008072,intron_26:29015260-29016037,intron_22:29012841-29013188,exon_33:29005674-29005983,intron_24:29013475-29014046,intron_5:29003590-29004836,exon_13:29016037-29016208,exon_36:29003226-29003446,exon_14:29014953-29015260,exon_25:29009563-29009888,exon_30:29006724-29006908,exon_31:29006472-29006638,intron_25:29014129-29014953,; LDM_E 48 7642 MYH8;--

oar_circ_0000236 chr11 28977498 29050517 - 73019 30901 ENSOARG00000011486;ENSOARG00000007268;ENSOARG00000009609; exon_22:29041496-29041739,intron_31:29049586-29050235,exon_33:29034951-29035260,exon_29:29037218-29037415,intron_23:29044855-29045923,exon_14:29047172-29047343,exon_30:29036308-29036492,exon_36:29033954-29034125,exon_21:29043571-29043827,exon_23:29041227-29041404,exon_15:29046086-29046396,intron_26:29047343-29048444,intron_12:29037696-29039471,intron_18:29041739-29043571,exon_39:29027984-29031410,exon_34:29034661-29034865,exon_26:29040197-29040575,exon_31:29035865-29036031,intron_7:29035260-29035653,intron_9:29036031-29036308,intron_13:29039598-29040197,intron_1:29031410-29033367,intron_10:29036492-29037218,intron_25:29046396-29047172,;exon_3:28979828-28979985,intron_37:28977734-28979828,exon_1:28980887-28981097,intron_39:28980214-28980887,;exon_21:29011169-29011412,intron_10:29006908-29008072,intron_5:29003590-29004836,exon_13:29016037-29016208,exon_14:29014953-29015260,exon_3:29022953-29023110,intron_25:29014129-29014953,intron_26:29015260-29016037,intron_22:29012841-29013188,exon_33:29005674-29005983,intron_24:29013475-29014046,exon_1:29023460-29023664,intron_35:29019718-29022188,exon_36:29003226-29003446,intron_36:29022216-29022953,exon_30:29006724-29006908,exon_25:29009563-29009888,exon_31:29006472-29006638,; LDM_E 121 9048 -;MYH8;--

oar_circ_0000237 chr11 28980070 29023110 - 43040 11101 ENSOARG00000009609;ENSOARG00000007268; exon_31:29006472-29006638,exon_30:29006724-29006908,exon_25:29009563-29009888,exon_36:29003226-29003446,intron_36:29022216-29022953,intron_35:29019718-29022188,intron_24:29013475-29014046,intron_22:29012841-29013188,exon_33:29005674-29005983,intron_26:29015260-29016037,intron_25:29014129-29014953,exon_13:29016037-29016208,intron_5:29003590-29004836,exon_14:29014953-29015260,exon_3:29022953-29023110,exon_21:29011169-29011412,intron_10:29006908-29008072,;intron_39:28980214-28980887,exon_1:28980887-28981097,; LDM_E 98 4904 --;MYH8

oar_circ_0000238 chr11 28980070 29052759 - 72689 30664 ENSOARG00000011486;ENSOARG00000007268;ENSOARG00000009609; intron_10:29036492-29037218,intron_1:29031410-29033367,intron_25:29046396-29047172,intron_13:29039598-29040197,intron_9:29036031-29036308,exon_31:29035865-29036031,intron_7:29035260-29035653,exon_34:29034661-29034865,exon_26:29040197-29040575,intron_18:29041739-29043571,exon_39:29027984-29031410,exon_15:29046086-29046396,intron_26:29047343-29048444,intron_12:29037696-29039471,intron_35:29051511-29052602,exon_21:29043571-29043827,exon_23:29041227-29041404,exon_36:29033954-29034125,exon_14:29047172-29047343,intron_34:29050717-29051483,exon_30:29036308-29036492,exon_29:29037218-29037415,intron_23:29044855-29045923,exon_4:29052602-29052759,exon_33:29034951-29035260,intron_31:29049586-29050235,exon_22:29041496-29041739,;exon_1:28980887-28981097,intron_39:28980214-28980887,;exon_13:29016037-29016208,intron_5:29003590-29004836,exon_3:29022953-29023110,exon_14:29014953-29015260,intron_25:29014129-29014953,exon_21:29011169-29011412,intron_10:29006908-29008072,exon_36:29003226-29003446,intron_36:29022216-29022953,intron_35:29019718-29022188,exon_31:29006472-29006638,exon_25:29009563-29009888,exon_30:29006724-29006908,intron_22:29012841-29013188,exon_33:29005674-29005983,intron_26:29015260-29016037,exon_1:29023460-29023664,intron_24:29013475-29014046,; LDM_E 125 6325 -;MYH8;--

oar_circ_0000239 chr11 29008072 29036492 - 28420 16420 ENSOARG00000009609;ENSOARG00000011486; exon_22:29010683-29010860,intron_36:29022216-29022953,exon_20:29012115-29012374,intron_35:29019718-29022188,intron_24:29013475-29014046,exon_21:29011169-29011412,intron_30:29018148-29018300,exon_25:29009563-29009888,exon_29:29008072-29008269,exon_3:29022953-29023110,exon_14:29014953-29015260,exon_1:29023460-29023664,intron_26:29015260-29016037,intron_32:29018596-29019230,intron_19:29011412-29012115,intron_25:29014129-29014953,;exon_34:29034661-29034865,exon_30:29036308-29036492,intron_9:29036031-29036308,exon_36:29033954-29034125,intron_1:29031410-29033367,exon_39:29027984-29031410,exon_33:29034951-29035260,exon_31:29035865-29036031,intron_5:29034333-29034661,intron_7:29035260-29035653,intron_3:29033686-29033954,; LDM_A 2044 40042 --;-

oar_circ_0000240 chr11 29008218 29037364 + 29146 29146 -- -- LDM_E 8 0 --

oar_circ_0000241 chr11 29010323 29040757 - 30434 19735 ENSOARG00000009609;ENSOARG00000011486; intron_26:29015260-29016037,intron_25:29014129-29014953,exon_21:29011169-29011412,intron_24:29013475-29014046,intron_19:29011412-29012115,intron_32:29018596-29019230,exon_1:29023460-29023664,exon_22:29010683-29010860,exon_14:29014953-29015260,exon_3:29022953-29023110,exon_20:29012115-29012374,intron_35:29019718-29022188,intron_36:29022216-29022953,intron_30:29018148-29018300,;intron_5:29034333-29034661,intron_10:29036492-29037218,exon_29:29037218-29037415,exon_39:29027984-29031410,intron_11:29037415-29037577,exon_26:29040197-29040575,exon_36:29033954-29034125,intron_7:29035260-29035653,intron_3:29033686-29033954,exon_33:29034951-29035260,exon_31:29035865-29036031,intron_1:29031410-29033367,exon_30:29036308-29036492,intron_12:29037696-29039471,exon_34:29034661-29034865,intron_13:29039598-29040197,intron_9:29036031-29036308,; LDM_A 1494 40842 --;-

oar_circ_0000242 chr11 29012717 29044059 - 31342 21107 ENSOARG00000011486;ENSOARG00000009609; exon_31:29035865-29036031,exon_33:29034951-29035260,intron_7:29035260-29035653,intron_3:29033686-29033954,intron_18:29041739-29043571,exon_22:29041496-29041739,exon_21:29043571-29043827,intron_1:29031410-29033367,intron_12:29037696-29039471,exon_34:29034661-29034865,exon_30:29036308-29036492,exon_23:29041227-29041404,intron_13:29039598-29040197,intron_9:29036031-29036308,intron_5:29034333-29034661,intron_15:29040757-29041003,intron_10:29036492-29037218,exon_29:29037218-29037415,exon_39:29027984-29031410,intron_11:29037415-29037577,exon_26:29040197-29040575,exon_36:29033954-29034125,;intron_24:29013475-29014046,intron_25:29014129-29014953,intron_32:29018596-29019230,intron_26:29015260-29016037,intron_35:29019718-29022188,intron_36:29022216-29022953,exon_1:29023460-29023664,exon_3:29022953-29023110,exon_14:29014953-29015260,intron_30:29018148-29018300,; LDM_E,LDM_A 26,130 1361,71110 -;--

oar_circ_0000243 chr11 29012717 29073142 - 60425 42745 ENSOARG00000011486;ENSOARG00000009609;ENSOARG00000012656; intron_3:29033686-29033954,intron_7:29035260-29035653,exon_33:29034951-29035260,intron_12:29037696-29039471,exon_13:29048444-29048594,exon_15:29046086-29046396,intron_31:29049586-29050235,intron_10:29036492-29037218,intron_21:29044271-29044568,intron_34:29050717-29051483,intron_38:29053349-29055034,intron_26:29047343-29048444,exon_4:29052602-29052759,exon_2:29053105-29053349,exon_39:29027984-29031410,exon_22:29041496-29041739,intron_18:29041739-29043571,intron_23:29044855-29045923,exon_31:29035865-29036031,intron_13:29039598-29040197,intron_9:29036031-29036308,exon_30:29036308-29036492,exon_34:29034661-29034865,exon_23:29041227-29041404,exon_21:29043571-29043827,intron_1:29031410-29033367,intron_29:29049138-29049290,intron_15:29040757-29041003,intron_5:29034333-29034661,exon_36:29033954-29034125,intron_25:29046396-29047172,exon_26:29040197-29040575,intron_28:29048803-29048999,intron_11:29037415-29037577,intron_35:29051511-29052602,exon_29:29037218-29037415,;intron_30:29018148-29018300,exon_3:29022953-29023110,exon_14:29014953-29015260,exon_1:29023460-29023664,intron_36:29022216-29022953,intron_35:29019718-29022188,intron_26:29015260-29016037,intron_32:29018596-29019230,intron_24:29013475-29014046,intron_25:29014129-29014953,;exon_25:29068372-29068747,intron_1:29058187-29059848,intron_19:29071585-29073005,intron_13:29067275-29068372,intron_7:29064488-29064654,exon_33:29063889-29064093,exon_30:29064878-29065044,exon_28:29066023-29066220,exon_38:29058034-29058187,intron_12:29066472-29067148,intron_18:29069762-29071329,exon_29:29065126-29065310,exon_22:29069231-29069408,intron_10:29065310-29066023,exon_21:29069519-29069762,exon_20:29071329-29071585,intron_5:29060628-29063889,exon_32:29064179-29064488,exon_35:29060251-29060422,; LDM_E,LDM_A 17,163 4429,109920 -;--;MYH1

oar_circ_0000244 chr11 29023184 29052829 - 29645 21191 ENSOARG00000011486;ENSOARG00000009609; intron_11:29037415-29037577,intron_35:29051511-29052602,exon_29:29037218-29037415,intron_25:29046396-29047172,exon_36:29033954-29034125,exon_26:29040197-29040575,intron_28:29048803-29048999,intron_15:29040757-29041003,intron_5:29034333-29034661,intron_29:29049138-29049290,intron_1:29031410-29033367,exon_21:29043571-29043827,intron_9:29036031-29036308,intron_13:29039598-29040197,exon_23:29041227-29041404,exon_30:29036308-29036492,exon_34:29034661-29034865,intron_23:29044855-29045923,exon_31:29035865-29036031,exon_22:29041496-29041739,intron_18:29041739-29043571,exon_4:29052602-29052759,exon_39:29027984-29031410,intron_26:29047343-29048444,intron_34:29050717-29051483,intron_31:29049586-29050235,intron_10:29036492-29037218,intron_21:29044271-29044568,exon_13:29048444-29048594,intron_12:29037696-29039471,exon_15:29046086-29046396,intron_3:29033686-29033954,intron_7:29035260-29035653,exon_33:29034951-29035260,;exon_1:29023460-29023664,; LDM_A 13 84008 -;--

oar_circ_0000245 chr11 29023250 29052895 + 29645 29645 -- -- LDM_A 8 0 --

oar_circ_0000246 chr11 29035653 29064488 - 28835 21619 ENSOARG00000012656;ENSOARG00000011486; exon_35:29060251-29060422,exon_32:29064179-29064488,intron_5:29060628-29063889,intron_1:29058187-29059848,exon_38:29058034-29058187,exon_33:29063889-29064093,;intron_38:29053349-29055034,intron_34:29050717-29051483,intron_15:29040757-29041003,intron_21:29044271-29044568,intron_10:29036492-29037218,intron_31:29049586-29050235,intron_29:29049138-29049290,exon_29:29037218-29037415,intron_35:29051511-29052602,intron_11:29037415-29037577,exon_4:29052602-29052759,exon_2:29053105-29053349,intron_28:29048803-29048999,exon_26:29040197-29040575,intron_25:29046396-29047172,intron_26:29047343-29048444,exon_31:29035865-29036031,intron_23:29044855-29045923,intron_18:29041739-29043571,exon_22:29041496-29041739,exon_21:29043571-29043827,exon_13:29048444-29048594,intron_12:29037696-29039471,exon_30:29036308-29036492,exon_23:29041227-29041404,exon_15:29046086-29046396,intron_9:29036031-29036308,intron_13:29039598-29040197,; LDM_A 7926 66797 MYH1;-

oar_circ_0000247 chr11 29035865 29064779 - 28914 21785 ENSOARG00000012656;ENSOARG00000011486; exon_38:29058034-29058187,intron_1:29058187-29059848,exon_33:29063889-29064093,intron_7:29064488-29064654,exon_35:29060251-29060422,intron_5:29060628-29063889,exon_32:29064179-29064488,;exon_31:29035865-29036031,intron_23:29044855-29045923,intron_18:29041739-29043571,exon_22:29041496-29041739,exon_21:29043571-29043827,intron_12:29037696-29039471,exon_13:29048444-29048594,exon_23:29041227-29041404,exon_30:29036308-29036492,exon_15:29046086-29046396,intron_9:29036031-29036308,intron_13:29039598-29040197,intron_38:29053349-29055034,intron_34:29050717-29051483,intron_15:29040757-29041003,intron_21:29044271-29044568,intron_10:29036492-29037218,intron_31:29049586-29050235,intron_29:29049138-29049290,intron_35:29051511-29052602,exon_29:29037218-29037415,intron_11:29037415-29037577,exon_4:29052602-29052759,exon_2:29053105-29053349,exon_26:29040197-29040575,intron_28:29048803-29048999,intron_25:29046396-29047172,intron_26:29047343-29048444,; LDM_E,LDM_A 9,2668 1968,66798 MYH1;-

oar_circ_0000248 chr11 29041227 29041404 - 177 177 ENSOARG00000011486; exon_23:29041227-29041404,; LDM_A 2 4459 -

oar_circ_0000249 chr11 29041496 29041655 - 159 159 ENSOARG00000011486; exon_22:29041496-29041739,; LDM_A 2 1314 -

oar_circ_0000250 chr11 29041496 29069408 - 27912 20483 ENSOARG00000011486;ENSOARG00000012656; intron_23:29044855-29045923,exon_22:29041496-29041739,intron_18:29041739-29043571,exon_21:29043571-29043827,exon_15:29046086-29046396,exon_13:29048444-29048594,intron_34:29050717-29051483,intron_38:29053349-29055034,intron_31:29049586-29050235,intron_29:29049138-29049290,intron_21:29044271-29044568,exon_2:29053105-29053349,exon_4:29052602-29052759,intron_35:29051511-29052602,intron_28:29048803-29048999,intron_26:29047343-29048444,intron_25:29046396-29047172,;exon_35:29060251-29060422,exon_28:29066023-29066220,intron_5:29060628-29063889,exon_32:29064179-29064488,intron_13:29067275-29068372,intron_10:29065310-29066023,exon_30:29064878-29065044,exon_33:29063889-29064093,intron_7:29064488-29064654,exon_29:29065126-29065310,exon_22:29069231-29069408,exon_25:29068372-29068747,intron_1:29058187-29059848,exon_38:29058034-29058187,intron_12:29066472-29067148,; LDM_A 2878 59706 -;MYH1

oar_circ_0000251 chr11 29043571 29069762 - 26191 18651 ENSOARG00000012656;ENSOARG00000011486; intron_1:29058187-29059848,exon_25:29068372-29068747,exon_30:29064878-29065044,exon_33:29063889-29064093,intron_7:29064488-29064654,intron_13:29067275-29068372,exon_28:29066023-29066220,intron_12:29066472-29067148,exon_38:29058034-29058187,exon_29:29065126-29065310,exon_22:29069231-29069408,exon_21:29069519-29069762,intron_10:29065310-29066023,exon_35:29060251-29060422,exon_32:29064179-29064488,intron_5:29060628-29063889,;intron_21:29044271-29044568,intron_31:29049586-29050235,intron_29:29049138-29049290,intron_38:29053349-29055034,intron_23:29044855-29045923,intron_34:29050717-29051483,intron_28:29048803-29048999,exon_13:29048444-29048594,exon_15:29046086-29046396,intron_26:29047343-29048444,intron_25:29046396-29047172,intron_35:29051511-29052602,exon_21:29043571-29043827,exon_2:29053105-29053349,exon_4:29052602-29052759,; LDM_A 1617 50641 MYH1;-

oar_circ_0000252 chr11 29044147 29073142 - 28995 21638 ENSOARG00000011486;ENSOARG00000012656; intron_23:29044855-29045923,intron_38:29053349-29055034,intron_34:29050717-29051483,intron_21:29044271-29044568,intron_29:29049138-29049290,intron_31:29049586-29050235,intron_35:29051511-29052602,exon_4:29052602-29052759,exon_2:29053105-29053349,intron_26:29047343-29048444,intron_25:29046396-29047172,intron_28:29048803-29048999,exon_13:29048444-29048594,exon_15:29046086-29046396,;intron_19:29071585-29073005,intron_1:29058187-29059848,exon_25:29068372-29068747,exon_28:29066023-29066220,intron_13:29067275-29068372,exon_30:29064878-29065044,exon_33:29063889-29064093,intron_7:29064488-29064654,exon_22:29069231-29069408,exon_29:29065126-29065310,exon_38:29058034-29058187,intron_18:29069762-29071329,intron_12:29066472-29067148,exon_20:29071329-29071585,exon_35:29060251-29060422,exon_32:29064179-29064488,intron_5:29060628-29063889,intron_10:29065310-29066023,exon_21:29069519-29069762,; LDM_E,LDM_A 35,5935 3068,38810 -;MYH1

oar_circ_0000253 chr11 29044568 29073354 - 28786 21341 ENSOARG00000011486;ENSOARG00000012656; intron_25:29046396-29047172,intron_26:29047343-29048444,intron_28:29048803-29048999,exon_15:29046086-29046396,exon_13:29048444-29048594,exon_2:29053105-29053349,exon_4:29052602-29052759,intron_35:29051511-29052602,intron_29:29049138-29049290,intron_31:29049586-29050235,intron_34:29050717-29051483,intron_38:29053349-29055034,intron_23:29044855-29045923,;intron_13:29067275-29068372,exon_33:29063889-29064093,exon_30:29064878-29065044,intron_7:29064488-29064654,exon_28:29066023-29066220,intron_1:29058187-29059848,exon_25:29068372-29068747,intron_19:29071585-29073005,intron_10:29065310-29066023,exon_21:29069519-29069762,exon_20:29071329-29071585,exon_35:29060251-29060422,exon_32:29064179-29064488,intron_5:29060628-29063889,exon_38:29058034-29058187,intron_18:29069762-29071329,intron_12:29066472-29067148,exon_29:29065126-29065310,exon_22:29069231-29069408,; LDM_A 6 38792 -;MYH1

oar_circ_0000254 chr11 29044767 29074129 - 29362 21998 ENSOARG00000012656;ENSOARG00000011486; intron_21:29073354-29074011,exon_25:29068372-29068747,intron_1:29058187-29059848,intron_19:29071585-29073005,exon_33:29063889-29064093,exon_30:29064878-29065044,intron_7:29064488-29064654,intron_13:29067275-29068372,exon_28:29066023-29066220,intron_18:29069762-29071329,intron_12:29066472-29067148,exon_38:29058034-29058187,exon_22:29069231-29069408,exon_29:29065126-29065310,exon_21:29069519-29069762,intron_10:29065310-29066023,exon_35:29060251-29060422,exon_32:29064179-29064488,intron_5:29060628-29063889,exon_20:29071329-29071585,;intron_34:29050717-29051483,intron_23:29044855-29045923,intron_38:29053349-29055034,intron_31:29049586-29050235,intron_29:29049138-29049290,exon_2:29053105-29053349,exon_4:29052602-29052759,intron_35:29051511-29052602,intron_28:29048803-29048999,exon_15:29046086-29046396,exon_13:29048444-29048594,intron_25:29046396-29047172,intron_26:29047343-29048444,; LDM_A 671 38793 MYH1;-

oar_circ_0000255 chr11 29048444 29076254 - 27810 20497 ENSOARG00000012656;ENSOARG00000011486; intron_23:29074304-29075133,intron_19:29071585-29073005,intron_1:29058187-29059848,exon_25:29068372-29068747,exon_14:29075648-29075958,intron_21:29073354-29074011,exon_28:29066023-29066220,intron_7:29064488-29064654,exon_30:29064878-29065044,intron_24:29075204-29075648,exon_33:29063889-29064093,exon_13:29076083-29076254,intron_13:29067275-29068372,exon_22:29069231-29069408,exon_29:29065126-29065310,intron_18:29069762-29071329,intron_12:29066472-29067148,exon_38:29058034-29058187,intron_5:29060628-29063889,exon_32:29064179-29064488,exon_35:29060251-29060422,exon_20:29071329-29071585,exon_21:29069519-29069762,intron_10:29065310-29066023,;intron_29:29049138-29049290,intron_31:29049586-29050235,intron_34:29050717-29051483,intron_38:29053349-29055034,intron_28:29048803-29048999,exon_13:29048444-29048594,exon_2:29053105-29053349,exon_4:29052602-29052759,intron_35:29051511-29052602,; LDM_E,LDM_A 56,5891 3574,30895 MYH1;-

oar_circ_0000256 chr11 29050602 29080724 - 30122 22633 ENSOARG00000012656;ENSOARG00000011486; exon_20:29071329-29071585,intron_29:29078425-29079394,exon_35:29060251-29060422,exon_32:29064179-29064488,intron_5:29060628-29063889,intron_10:29065310-29066023,exon_21:29069519-29069762,intron_32:29080231-29080631,exon_22:29069231-29069408,exon_29:29065126-29065310,exon_38:29058034-29058187,intron_30:29079498-29079951,intron_12:29066472-29067148,intron_18:29069762-29071329,exon_28:29066023-29066220,intron_13:29067275-29068372,exon_13:29076083-29076254,exon_33:29063889-29064093,intron_24:29075204-29075648,exon_30:29064878-29065044,intron_7:29064488-29064654,intron_19:29071585-29073005,intron_26:29076254-29077565,intron_23:29074304-29075133,exon_12:29077565-29077715,intron_21:29073354-29074011,intron_1:29058187-29059848,exon_14:29075648-29075958,exon_25:29068372-29068747,;intron_35:29051511-29052602,intron_38:29053349-29055034,exon_2:29053105-29053349,exon_4:29052602-29052759,intron_34:29050717-29051483,; LDM_A 577 30550 MYH1;-

oar_circ_0000257 chr11 29051483 29080920 - 29437 21867 ENSOARG00000011486;ENSOARG00000012656; intron_38:29053349-29055034,intron_35:29051511-29052602,exon_2:29053105-29053349,exon_4:29052602-29052759,;exon_28:29066023-29066220,exon_33:29063889-29064093,intron_24:29075204-29075648,exon_30:29064878-29065044,intron_7:29064488-29064654,intron_13:29067275-29068372,exon_13:29076083-29076254,intron_23:29074304-29075133,exon_12:29077565-29077715,intron_19:29071585-29073005,intron_26:29076254-29077565,intron_21:29073354-29074011,exon_25:29068372-29068747,intron_1:29058187-29059848,exon_14:29075648-29075958,exon_35:29060251-29060422,intron_5:29060628-29063889,exon_32:29064179-29064488,exon_20:29071329-29071585,intron_29:29078425-29079394,exon_21:29069519-29069762,intron_10:29065310-29066023,exon_22:29069231-29069408,exon_29:29065126-29065310,intron_32:29080231-29080631,intron_30:29079498-29079951,intron_18:29069762-29071329,intron_12:29066472-29067148,exon_38:29058034-29058187,; LDM_A 3 30282 -;MYH1

oar_circ_0000258 chr11 29052693 29081877 - 29184 21374 ENSOARG00000012656;ENSOARG00000011486; exon_28:29066023-29066220,intron_24:29075204-29075648,exon_33:29063889-29064093,exon_30:29064878-29065044,intron_7:29064488-29064654,exon_13:29076083-29076254,intron_13:29067275-29068372,intron_23:29074304-29075133,exon_12:29077565-29077715,intron_19:29071585-29073005,intron_26:29076254-29077565,intron_21:29073354-29074011,intron_1:29058187-29059848,exon_14:29075648-29075958,exon_25:29068372-29068747,exon_35:29060251-29060422,exon_32:29064179-29064488,intron_5:29060628-29063889,exon_20:29071329-29071585,intron_29:29078425-29079394,exon_21:29069519-29069762,intron_10:29065310-29066023,exon_29:29065126-29065310,exon_22:29069231-29069408,intron_32:29080231-29080631,intron_30:29079498-29079951,intron_18:29069762-29071329,intron_12:29066472-29067148,intron_35:29081031-29081786,exon_38:29058034-29058187,;intron_38:29053349-29055034,exon_2:29053105-29053349,; LDM_A 43 28925 MYH1;-

oar_circ_0000259 chr11 29060502 29151487 - 90985 20099 ENSOARG00000012656;ENSOARG00000013899; exon_28:29066023-29066220,exon_3:29081786-29081943,exon_13:29076083-29076254,intron_13:29067275-29068372,intron_7:29064488-29064654,intron_36:29081943-29083502,intron_24:29075204-29075648,exon_33:29063889-29064093,exon_30:29064878-29065044,intron_26:29076254-29077565,intron_19:29071585-29073005,exon_12:29077565-29077715,intron_23:29074304-29075133,exon_14:29075648-29075958,exon_25:29068372-29068747,intron_21:29073354-29074011,intron_29:29078425-29079394,exon_20:29071329-29071585,intron_5:29060628-29063889,exon_32:29064179-29064488,intron_10:29065310-29066023,exon_21:29069519-29069762,intron_32:29080231-29080631,exon_1:29083734-29083938,exon_22:29069231-29069408,exon_29:29065126-29065310,intron_35:29081031-29081786,intron_18:29069762-29071329,intron_12:29066472-29067148,intron_30:29079498-29079951,;intron_1:29149903-29150622,; LDM_A 143 21912 MYH1;MYH3

oar_circ_0000260 chr11 29066023 29067275 - 1252 873 ENSOARG00000012656; intron_12:29066472-29067148,exon_28:29066023-29066220,; LDM_A 7 791 MYH1

oar_circ_0000261 chr11 29076083 29160914 - 84831 10695 ENSOARG00000013899;ENSOARG00000012656; intron_1:29149903-29150622,intron_9:29153696-29153978,intron_23:29159000-29159590,intron_20:29157991-29158310,exon_29:29155245-29155442,exon_20:29158310-29158566,exon_34:29152475-29152658,exon_22:29157491-29157668,exon_25:29156530-29156728,intron_5:29151487-29152332,exon_33:29152774-29153083,exon_14:29160607-29160914,exon_30:29154722-29154906,;intron_29:29078425-29079394,exon_3:29081786-29081943,exon_13:29076083-29076254,intron_36:29081943-29083502,intron_26:29076254-29077565,intron_32:29080231-29080631,exon_12:29077565-29077715,exon_1:29083734-29083938,intron_35:29081031-29081786,intron_30:29079498-29079951,; LDM_E,LDM_A 411,869 18591,2644 MYH3;MYH1

oar_circ_0000262 chr11 29083502 29166772 - 83270 6976 ENSOARG00000013899;ENSOARG00000012656; intron_1:29149903-29150622,intron_9:29153696-29153978,intron_23:29159000-29159590,exon_29:29155245-29155442,exon_34:29152475-29152658,exon_25:29156530-29156728,intron_34:29163770-29164411,exon_30:29154722-29154906,intron_36:29165193-29165528,exon_13:29161013-29161184,intron_37:29165556-29166615,intron_20:29157991-29158310,exon_20:29158310-29158566,exon_22:29157491-29157668,intron_5:29151487-29152332,exon_14:29160607-29160914,exon_33:29152774-29153083,;exon_1:29083734-29083938,; LDM_E,LDM_A 21,119 20900,1716 MYH3;MYH1

oar_circ_0000263 chr11 30273832 30301943 + 28111 27888 ENSOARG00000015404; intron_2:30273935-30292522,exon_3:30292522-30292697,intron_3:30292697-30301823,; LDM_E,LDM_A 13,14 1167,456 MAP2K4

oar_circ_0000264 chr11 30273832 30311013 + 37181 36658 ENSOARG00000015404; intron_4:30301943-30306440,exon_3:30292522-30292697,intron_6:30308938-30310885,intron_3:30292697-30301823,intron_5:30306560-30308886,intron_2:30273935-30292522,; LDM_E,LDM_A 15,4 1679,707 MAP2K4

oar_circ_0000265 chr11 30301823 30311013 + 9190 8770 ENSOARG00000015404; intron_5:30306560-30308886,intron_4:30301943-30306440,intron_6:30308938-30310885,; LDM_E 2 512 MAP2K4

oar_circ_0000266 chr11 32398054 32402115 - 4061 3977 ENSOARG00000016545; exon_2:32401507-32402115,intron_6:32398138-32401507,; LDM_E,LDM_A 24,9 63,11 TEKT3

oar_circ_0000267 chr11 32901113 32912226 + 11113 10979 ENSOARG00000017147; intron_1:32901291-32904645,intron_2:32904779-32912033,exon_3:32912033-32912226,exon_1:32901113-32901291,; LDM_E 2 1049 NCOR1

oar_circ_0000268 chr11 32985583 32986129 + 546 224 ENSOARG00000017147; exon_40:32985905-32986129,; LDM_E 4 30 NCOR1

oar_circ_0000269 chr11 32992808 32996615 + 3807 3807 ENSOARG00000017147; exon_45:32996435-32996615,exon_44:32992808-32993030,intron_44:32993030-32996435,; LDM_A 2 331 NCOR1

oar_circ_0000270 chr11 33360250 33360735 + 485 415 ENSOARG00000017770; intron_3:33360283-33360698,; LDM_E 2 18 ULK2

oar_circ_0000271 chr11 33795896 33813203 - 17307 15225 ENSOARG00000018414; intron_9:33802422-33803563,intron_8:33801837-33802391,exon_9:33801655-33801837,intron_11:33804015-33808546,intron_15:33810097-33813028,intron_7:33796032-33801655,intron_10:33803646-33803909,; LDM_E 2 23 SLC5A10

oar_circ_0000272 chr11 34522130 34523189 - 1059 809 ENSOARG00000002206; intron_17:34522238-34523047,; LDM_E 2 21 MPRIP

oar_circ_0000273 chr11 35015791 35033524 - 17733 17169 ENSOARG00000002805; intron_9:35027381-35033456,intron_8:35025331-35027292,intron_5:35015882-35016180,intron_6:35016281-35024941,exon_8:35024941-35025116,; LDM_E 2 232 UTP18

oar_circ_0000274 chr11 35075216 35082570 + 7354 7010 ENSOARG00000002872; exon_2:35075216-35075418,intron_2:35075418-35079733,intron_3:35079867-35081542,intron_4:35081666-35082484,; LDM_E 3 446 MBTD1

oar_circ_0000275 chr11 35095825 35097826 + 2001 1176 ENSOARG00000002872; exon_9:35096489-35096724,intron_9:35096724-35097328,intron_10:35097384-35097721,; LDM_E,LDM_A 3,5 69,25 MBTD1

oar_circ_0000276 chr11 35427534 35428161 + 627 627 ENSOARG00000004446; exon_1:35427675-35428704,; LDM_A 2 1039 TOB1

oar_circ_0000277 chr11 35529196 35543042 - 13846 11895 ENSOARG00000003331; intron_8:35538839-35540071,intron_3:35534516-35535212,intron_9:35540111-35542975,intron_6:35537397-35538245,exon_11:35530085-35530767,intron_7:35538320-35538694,intron_5:35536042-35537292,exon_8:35535212-35535496,exon_7:35535880-35536042,exon_9:35534355-35534516,intron_2:35531013-35534355,; LDM_A 2 1090 LUC7L3

oar_circ_0000278 chr11 35530138 35530767 - 629 629 ENSOARG00000003331; exon_11:35530085-35530767,; LDM_E 2 453 LUC7L3

oar_circ_0000279 chr11 36366174 36368310 - 2136 2025 ENSOARG00000005653; exon_9:36366174-36366366,intron_7:36366366-36368199,; LDM_E,LDM_A 2,3 44,42 KAT7

oar_circ_0000280 chr11 36370710 36386067 - 15357 14949 ENSOARG00000005653; exon_2:36381299-36381488,intron_11:36375300-36379660,intron_12:36379900-36381299,intron_10:36372326-36375217,exon_4:36379660-36379900,intron_9:36370809-36372236,intron_13:36381476-36385919,; LDM_E 2 433 KAT7

oar_circ_0000281 chr11 37154394 37157812 - 3418 3102 ENSOARG00000006622; intron_2:37154485-37155255,intron_3:37155368-37157700,; LDM_A 2 36 UBE2Z

oar_circ_0000282 chr11 37973136 37973328 + 192 192 -- -- LDM_A 2 0 --

oar_circ_0000283 chr11 37980430 37980617 - 187 187 -- -- LDM_E 2 0 --

oar_circ_0000284 chr11 39047953 39055351 + 7398 7205 ENSOARG00000010065; intron_3:39048038-39055243,; LDM_E 2 284 LASP1

oar_circ_0000285 chr11 40115809 40118277 + 2468 458 ENSOARG00000014063; exon_3:40116790-40117072,exon_5:40117648-40117824,; LDM_E,LDM_A 12,2 62,4 CDC6

oar_circ_0000286 chr11 40115809 40118291 + 2482 2251 ENSOARG00000014063; intron_4:40117396-40117648,exon_2:40115809-40116000,intron_5:40117824-40118184,intron_2:40116000-40116790,exon_3:40116790-40117072,exon_5:40117648-40117824,exon_4:40117196-40117396,; LDM_E 26 62 CDC6

oar_circ_0000287 chr11 40115809 40119414 + 3605 2251 ENSOARG00000014063; intron_2:40116000-40116790,exon_3:40116790-40117072,exon_5:40117648-40117824,exon_4:40117196-40117396,intron_4:40117396-40117648,exon_2:40115809-40116000,intron_5:40117824-40118184,; LDM_E 3 62 CDC6

oar_circ_0000288 chr11 41746719 41747200 - 481 358 ENSOARG00000018768; intron_3:41746842-41747048,exon_2:41747048-41747200,; LDM_A 40 8 RAB5C

oar_circ_0000289 chr11 41819636 41820647 - 1011 627 ENSOARG00000018964; intron_8:41819729-41820356,; LDM_A 2 16 --

oar_circ_0000290 chr11 41835246 41836950 + 1704 1704 -- -- LDM_A 2 0 --

oar_circ_0000291 chr11 42064266 42064489 + 223 223 ENSOARG00000001896; intron_5:42059831-42065465,; LDM_E 2 19 ATP6V0A1

oar_circ_0000292 chr11 42166671 42211534 + 44863 726 ENSOARG00000002687; exon_3:42166671-42166839,exon_11:42170198-42170404,intron_8:42169436-42169788,; LDM_E,LDM_A 20,42 52,37 -

oar_circ_0000293 chr11 42170206 42215331 + 45125 2075 ENSOARG00000002762;ENSOARG00000002687; intron_3:42212271-42212782,exon_9:42215169-42215331,intron_7:42214581-42214933,intron_5:42213403-42214247,;exon_11:42170198-42170404,; LDM_E,LDM_A 27,29 88,39 TUBG2;-

oar_circ_0000294 chr11 42260180 42265001 - 4821 4477 ENSOARG00000003316; exon_6:42261396-42261573,intron_15:42261573-42263376,intron_14:42260283-42261396,intron_16:42263497-42264881,; LDM_A 2 56 EZH1

oar_circ_0000295 chr11 42360044 42392716 - 32672 32672 -- -- LDM_E,LDM_A 48,18 0,0 --

oar_circ_0000296 chr11 42360144 42373888 - 13744 13744 -- -- LDM_E,LDM_A 4,2 0,0 --

oar_circ_0000297 chr11 42360144 42385562 - 25418 25418 -- -- LDM_E,LDM_A 5,3 0,0 --

oar_circ_0000298 chr11 42373751 42385425 + 11674 11674 ENSOARG00000004027; intron_2:42370140-42392554,; LDM_E,LDM_A 3,3 68,26 AOC3

oar_circ_0000299 chr11 42373785 42385459 + 11674 11674 ENSOARG00000004027; intron_2:42370140-42392554,; LDM_E,LDM_A 3,3 71,27 AOC3

oar_circ_0000300 chr11 42373785 42392984 + 19199 19199 ENSOARG00000004027; intron_2:42370140-42392554,exon_3:42392554-42393695,; LDM_E 11 221 AOC3

oar_circ_0000301 chr11 42373811 42385485 + 11674 11674 ENSOARG00000004027; intron_2:42370140-42392554,; LDM_E,LDM_A 3,3 73,27 AOC3

oar_circ_0000302 chr11 42373811 42393010 + 19199 19199 ENSOARG00000004027; exon_3:42392554-42393695,intron_2:42370140-42392554,; LDM_E 12 230 AOC3

oar_circ_0000303 chr11 42385459 42392984 + 7525 7525 ENSOARG00000004027; intron_2:42370140-42392554,exon_3:42392554-42393695,; LDM_E,LDM_A 14,7 125,63 AOC3

oar_circ_0000304 chr11 42385485 42393010 + 7525 7525 ENSOARG00000004027; intron_2:42370140-42392554,exon_3:42392554-42393695,; LDM_E,LDM_A 16,7 125,57 AOC3

oar_circ_0000305 chr11 42480605 42481093 - 488 488 -- -- LDM_E 2 0 --

oar_circ_0000306 chr11 42621396 42623965 + 2569 2491 ENSOARG00000005012; exon_7:42622472-42622687,intron_8:42623334-42623755,exon_8:42623166-42623334,intron_6:42621982-42622472,intron_7:42622687-42623166,intron_5:42621591-42621904,exon_5:42621396-42621591,exon_9:42623755-42623965,; LDM_E,LDM_A 2,2 239,162 NBR1

oar_circ_0000307 chr11 42623755 42623965 + 210 210 ENSOARG00000005012; exon_9:42623755-42623965,; LDM_E 2 64 NBR1

oar_circ_0000308 chr11 42837837 42839234 + 1397 1152 ENSOARG00000005409; intron_2:42837923-42838424,intron_3:42838497-42839148,; LDM_E,LDM_A 3,4 8,7 DHX8

oar_circ_0000309 chr11 43085581 43086275 - 694 694 -- -- LDM_A 2 0 --

oar_circ_0000310 chr11 43321705 43321944 - 239 239 ENSOARG00000006589; intron_3:43310737-43324701,; LDM_E 2 3 LSM12

oar_circ_0000311 chr11 43626661 43626828 - 167 167 -- -- LDM_E 2 0 --

oar_circ_0000312 chr11 43629426 43632267 - 2841 2841 ENSOARG00000008370; intron_30:43619327-43632123,; LDM_E 2 330 GPATCH8

oar_circ_0000313 chr11 43948395 43950578 - 2183 1191 ENSOARG00000000133; exon_1:43948434-43949625,; LDM_E 4 352 GJC1

oar_circ_0000314 chr11 43949061 43950578 - 1517 1191 ENSOARG00000000133; exon_1:43948434-43949625,; LDM_E 4 137 GJC1

oar_circ_0000315 chr11 43949061 43958730 - 9669 1191 ENSOARG00000000133; exon_1:43948434-43949625,; LDM_E 2 137 GJC1

oar_circ_0000316 chr11 44974444 44980934 - 6490 6306 ENSOARG00000010947; intron_14:44974600-44977046,exon_4:44980767-44980934,intron_16:44977554-44980767,exon_7:44974444-44974600,intron_15:44977122-44977446,; LDM_E 2 102 NSF

oar_circ_0000317 chr11 45446905 45448238 - 1333 1184 ENSOARG00000011438; intron_6:45447054-45447388,exon_10:45447388-45447577,exon_9:45448055-45448238,intron_7:45447577-45448055,; LDM_A 2 44 KANSL1

oar_circ_0000318 chr11 45471093 45472659 - 1566 1566 ENSOARG00000011438; intron_9:45457151-45472962,; LDM_A 2 199 KANSL1

oar_circ_0000319 chr11 45472962 45474066 - 1104 991 ENSOARG00000011438; intron_10:45473158-45473953,exon_7:45472962-45473158,; LDM_E,LDM_A 6,7 191,94 KANSL1

oar_circ_0000320 chr11 45472962 45482106 - 9144 8929 ENSOARG00000011438; exon_7:45472962-45473158,intron_10:45473158-45473953,intron_11:45474066-45482004,; LDM_E,LDM_A 3,8 1297,751 KANSL1

oar_circ_0000321 chr11 45472962 45493299 - 20337 19980 ENSOARG00000011438; exon_7:45472962-45473158,intron_10:45473158-45473953,intron_11:45474066-45482004,intron_12:45482106-45493157,; LDM_E,LDM_A 18,5 3198,1739 KANSL1

oar_circ_0000322 chr11 45562423 45563795 - 1372 1372 ENSOARG00000011438; exon_3:45562423-45563795,; LDM_E,LDM_A 11,9 1248,957 KANSL1

oar_circ_0000323 chr11 45639518 45658148 - 18630 17825 ENSOARG00000011644; exon_13:45640660-45640813,exon_6:45651574-45651729,intron_6:45639727-45640660,intron_15:45652558-45656321,exon_11:45644109-45644317,exon_14:45639518-45639727,intron_8:45643528-45644109,intron_11:45647864-45650146,intron_9:45644317-45645254,intron_7:45640813-45643358,intron_14:45651729-45652460,exon_7:45651257-45651469,exon_12:45643358-45643531,intron_12:45650261-45651257,intron_16:45656447-45658000,intron_10:45645354-45647748,; LDM_E 2 996 CDC27

oar_circ_0000324 chr11 45715470 45717927 + 2457 2379 ENSOARG00000011750; exon_3:45715470-45715620,intron_4:45717346-45717849,intron_3:45715620-45717172,exon_4:45717172-45717346,; LDM_E 46 1475 MYL4

oar_circ_0000325 chr11 46380524 46383451 + 2927 2645 ENSOARG00000012181; intron_2:46380596-46381515,intron_4:46382635-46383355,intron_3:46381585-46382591,; LDM_A 3 79 TLK2

oar_circ_0000326 chr11 46380524 46393617 + 13093 12811 ENSOARG00000012181; intron_5:46383451-46393449,exon_6:46393449-46393617,intron_2:46380596-46381515,intron_4:46382635-46383355,intron_3:46381585-46382591,; LDM_E 2 846 TLK2

oar_circ_0000327 chr11 46393449 46406881 + 13432 13243 ENSOARG00000012181; intron_7:46405481-46406788,intron_6:46393617-46405385,exon_6:46393449-46393617,; LDM_E 2 1031 TLK2

oar_circ_0000328 chr11 46393449 46411789 + 18340 17903 ENSOARG00000012181; intron_8:46406881-46410325,exon_6:46393449-46393617,intron_9:46410436-46411652,intron_7:46405481-46406788,intron_6:46393617-46405385,; LDM_E,LDM_A 7,6 1305,553 TLK2

oar_circ_0000329 chr11 46405385 46411789 + 6404 5967 ENSOARG00000012181; intron_7:46405481-46406788,intron_8:46406881-46410325,intron_9:46410436-46411652,; LDM_E,LDM_A 2,2 373,171 TLK2

oar_circ_0000330 chr11 47030186 47038069 + 7883 7734 ENSOARG00000012739; exon_7:47037882-47038069,intron_6:47030335-47037882,; LDM_E 7 229 TANC2

oar_circ_0000331 chr11 47086040 47091671 + 5631 5497 ENSOARG00000012739; exon_10:47086040-47086322,intron_10:47086322-47091537,; LDM_E 5 217 TANC2

oar_circ_0000332 chr11 47363436 47386038 + 22602 22133 ENSOARG00000013329; intron_4:47363536-47368708,intron_6:47376488-47385904,intron_5:47368822-47376367,; LDM_A 3 438 MAP3K3

oar_circ_0000333 chr11 4737774 4741095 + 3321 2106 ENSOARG00000004707; intron_5:4738779-4739766,intron_6:4739859-4740978,; LDM_E 5 22 TOM1L1

oar_circ_0000334 chr11 47626902 47642330 + 15428 15428 -- -- LDM_A 2 0 --

oar_circ_0000335 chr11 47626919 47642347 + 15428 15428 -- -- LDM_A 2 0 --

oar_circ_0000336 chr11 4762817 4767645 + 4828 4515 ENSOARG00000004707; intron_8:4762951-4767102,intron_9:4767163-4767527,; LDM_E,LDM_A 2,3 52,17 TOM1L1

oar_circ_0000337 chr11 4762817 4769218 + 6401 5991 ENSOARG00000004707; intron_9:4767163-4767527,intron_10:4767645-4769121,intron_8:4762951-4767102,; LDM_E 4 97 TOM1L1

oar_circ_0000338 chr11 47642177 47650809 - 8632 3291 ENSOARG00000014212; exon_3:47642177-47642498,exon_2:47643745-47644003,intron_2:47642498-47643745,intron_3:47644003-47645468,; LDM_A 3 62 -

oar_circ_0000339 chr11 47643745 47651789 - 8044 1723 ENSOARG00000014212; intron_3:47644003-47645468,exon_2:47643745-47644003,; LDM_A 9 40 -

oar_circ_0000340 chr11 47643745 47665806 - 22061 1723 ENSOARG00000014212; intron_3:47644003-47645468,exon_2:47643745-47644003,; LDM_E,LDM_A 5,5 43,40 -

oar_circ_0000341 chr11 47830281 47833417 - 3136 3136 ENSOARG00000014649; exon_4:47830281-47830529,exon_2:47833216-47833417,exon_3:47831787-47832127,intron_9:47832127-47833216,intron_8:47830529-47831787,; LDM_E 3 184 TEX2

oar_circ_0000342 chr11 48078439 48088918 - 10479 9696 ENSOARG00000014976; intron_3:48084149-48086374,exon_4:48087597-48087771,intron_1:48078557-48080365,intron_5:48087771-48088576,intron_4:48086515-48087597,intron_2:48080466-48084068,; LDM_E 3 333 POLG2

oar_circ_0000343 chr11 48079271 48079478 - 207 207 ENSOARG00000014976; intron_1:48078557-48080365,; LDM_E 7 8 POLG2

oar_circ_0000344 chr11 48080365 48088918 - 8553 7888 ENSOARG00000014976; intron_3:48084149-48086374,exon_4:48087597-48087771,intron_4:48086515-48087597,intron_5:48087771-48088576,intron_2:48080466-48084068,; LDM_E 2 235 POLG2

oar_circ_0000345 chr11 48107115 48115594 + 8479 8038 ENSOARG00000015133; intron_4:48109806-48114030,intron_3:48107223-48109695,intron_5:48114136-48115478,; LDM_E,LDM_A 19,19 423,237 CEP95

oar_circ_0000346 chr11 48134156 48137231 + 3075 2528 ENSOARG00000015133; intron_12:48134296-48135120,intron_14:48136119-48136874,exon_15:48136874-48137044,intron_13:48135210-48135989,; LDM_A 2 51 CEP95

oar_circ_0000347 chr11 48170425 48179745 - 9320 9151 ENSOARG00000015245; exon_9:48170425-48170584,intron_11:48174516-48176778,intron_10:48170584-48174431,exon_7:48176778-48176981,intron_12:48176981-48179661,; LDM_A 2 253 SMURF2

oar_circ_0000348 chr11 48170425 48189043 - 18618 18055 ENSOARG00000015245; intron_11:48174516-48176778,exon_7:48176778-48176981,intron_14:48182535-48184152,exon_9:48170425-48170584,intron_16:48185247-48188934,intron_13:48179745-48182450,intron_10:48170584-48174431,intron_15:48184218-48185113,intron_12:48176981-48179661,; LDM_A 2 558 SMURF2

oar_circ_0000349 chr11 48411054 48413175 - 2121 2012 ENSOARG00000015595; exon_17:48412928-48413175,intron_12:48412189-48412928,exon_18:48412037-48412189,intron_11:48411163-48412037,; LDM_E 3 202 BPTF

oar_circ_0000350 chr11 49418788 49432461 - 13673 12903 ENSOARG00000016414; exon_5:49421807-49422001,intron_7:49422001-49426948,intron_6:49421054-49421807,exon_8:49418788-49418998,intron_8:49427095-49429484,intron_9:49429632-49432266,exon_6:49420869-49421054,exon_7:49419473-49419770,intron_5:49419770-49420869,exon_2:49432266-49432461,; LDM_E,LDM_A 5,2 456,260 FOXK2

oar_circ_0000351 chr11 49426948 49432461 - 5513 5218 ENSOARG00000016414; intron_9:49429632-49432266,intron_8:49427095-49429484,exon_2:49432266-49432461,; LDM_A 3 101 FOXK2

oar_circ_0000352 chr11 49521252 49521470 + 218 218 ENSOARG00000016619; exon_2:49521245-49521470,; LDM_E 4 14 OGFOD3

oar_circ_0000353 chr11 49767810 49768036 - 226 226 -- -- LDM_E 2 0 --

oar_circ_0000354 chr11 50204248 50204758 + 510 201 ENSOARG00000018038; exon_7:50204557-50204758,; LDM_A 2 90 P4HB

oar_circ_0000355 chr11 52789315 52789490 + 175 175 -- -- LDM_E 2 0 --

oar_circ_0000356 chr11 54025668 54045371 - 19703 18817 ENSOARG00000006580; intron_5:54027987-54033507,intron_6:54033548-54037268,intron_10:54041629-54042193,intron_12:54044717-54045197,intron_8:54038000-54041344,intron_4:54025794-54027921,intron_7:54037413-54037941,exon_2:54045197-54045371,intron_11:54042301-54044661,; LDM_A 2 297 MFSD11

oar_circ_0000357 chr11 54409664 54412914 + 3250 2932 ENSOARG00000007435; exon_3:54409664-54409837,intron_3:54409837-54411406,intron_5:54412053-54412768,intron_4:54411522-54411997,; LDM_E 2 147 PRPSAP1

oar_circ_0000358 chr11 54490310 54493002 + 2692 2692 ENSOARG00000007710; intron_1:54467550-54492883,; LDM_A 2 10 RNF157

oar_circ_0000359 chr11 55548882 55549819 + 937 937 -- -- LDM_A 2 0 --

oar_circ_0000360 chr11 57834667 57835576 + 909 909 -- -- LDM_A 2 0 --

oar_circ_0000361 chr11 60025688 60041512 - 15824 15316 ENSOARG00000013869; intron_4:60040372-60041384,intron_3:60038421-60040294,intron_1:60025804-60031894,intron_2:60031940-60038281,; LDM_E 2 172 MAP2K6

oar_circ_0000362 chr11 60205408 60211278 + 5870 5639 ENSOARG00000013972; intron_7:60205597-60207148,exon_7:60205408-60205597,intron_8:60207296-60209700,exon_9:60209700-60209869,intron_9:60209869-60211195,; LDM_E 2 30 ABCA5

oar_circ_0000363 chr11 60214452 60222339 + 7887 6760 ENSOARG00000013972; intron_15:60216340-60216774,intron_13:60214572-60215593,exon_19:60222169-60222339,intron_18:60221618-60222169,exon_18:60221416-60221618,intron_16:60216914-60217953,intron_17:60218073-60221416,; LDM_A 2 31 ABCA5

oar_circ_0000364 chr11 60322247 60331579 + 9332 8657 ENSOARG00000014385; exon_6:60322720-60322947,exon_8:60330967-60331153,intron_6:60322947-60329600,intron_7:60329742-60330967,intron_5:60322354-60322720,; LDM_A 5 85 ABCA6

oar_circ_0000365 chr11 60344107 60345841 + 1734 1477 ENSOARG00000014385; intron_17:60344247-60345724,; LDM_A 2 13 ABCA6

oar_circ_0000366 chr11 60611047 60616015 - 4968 4962 ENSOARG00000015024; exon_1:60615835-60616009,exon_2:60611047-60611218,intron_9:60611218-60615835,; LDM_E 3 521 PRKAR1A

oar_circ_0000367 chr11 60705417 60711067 - 5650 5352 ENSOARG00000015185; intron_11:60707053-60708072,exon_3:60710879-60711067,intron_12:60708120-60710879,intron_10:60705555-60706941,; LDM_A 4 12 ARSG

oar_circ_0000368 chr11 61240087 61248481 - 8394 8181 ENSOARG00000015593; intron_5:61240237-61248418,; LDM_E,LDM_A 2,2 89,8 CEP112

oar_circ_0000369 chr11 61402148 61412493 - 10345 9827 ENSOARG00000015593; intron_13:61412025-61412338,intron_12:61409408-61411895,intron_10:61402228-61408394,intron_11:61408454-61409315,; LDM_E,LDM_A 8,6 112,20 CEP112

oar_circ_0000370 chr11 61408394 61412493 - 4099 3816 ENSOARG00000015593; intron_13:61412025-61412338,intron_12:61409408-61411895,intron_11:61408454-61409315,exon_12:61412338-61412493,; LDM_E 2 38 CEP112

oar_circ_0000371 chr11 61427267 61437340 - 10073 9623 ENSOARG00000015593; intron_15:61427411-61429503,intron_16:61429622-61434598,intron_17:61434698-61437253,; LDM_E,LDM_A 24,4 178,42 CEP112

oar_circ_0000372 chr11 61427267 61447118 - 19851 19275 ENSOARG00000015593; intron_18:61437340-61446739,intron_17:61434698-61437253,intron_16:61429622-61434598,intron_15:61427411-61429503,intron_19:61446817-61447070,; LDM_E,LDM_A 2,2 522,73 CEP112

oar_circ_0000373 chr11 61427267 61457852 - 30585 29840 ENSOARG00000015593; intron_15:61427411-61429503,intron_21:61455449-61457761,intron_19:61446817-61447070,intron_18:61437340-61446739,intron_17:61434698-61437253,intron_16:61429622-61434598,intron_20:61447118-61455371,; LDM_E 16 923 CEP112

oar_circ_0000374 chr11 61446739 61457852 - 11113 10818 ENSOARG00000015593; intron_19:61446817-61447070,intron_20:61447118-61455371,intron_21:61455449-61457761,; LDM_E,LDM_A 7,3 405,56 CEP112

oar_circ_0000375 chr11 61455371 61484369 - 28998 28715 ENSOARG00000015593; exon_3:61478409-61478582,exon_2:61480766-61480957,intron_22:61457852-61478409,intron_21:61455449-61457761,intron_23:61478582-61480766,intron_24:61480957-61484255,; LDM_E 3 2151 CEP112

oar_circ_0000376 chr11 62117691 62126005 - 8314 8314 ENSOARG00000015884; intron_27:62121956-62127918,intron_24:62117799-62118249,exon_9:62118249-62118448,intron_25:62118448-62120420,intron_26:62120496-62121904,; LDM_A 2 358 HELZ

oar_circ_0000377 chr11 7213195 7223048 + 9853 9383 ENSOARG00000006616; exon_3:7213195-7213355,intron_6:7218301-7218678,intron_3:7213355-7215011,intron_4:7215131-7217760,exon_6:7218143-7218301,intron_5:7217904-7218143,intron_7:7218730-7222894,; LDM_E 2 286 DGKE

oar_circ_0000378 chr11 7328527 7330374 - 1847 1594 ENSOARG00000007115; exon_3:7329263-7330374,intron_4:7328575-7329058,; LDM_E,LDM_A 13,7 446,341 COIL

oar_circ_0000379 chr11 8227533 8233896 - 6363 5901 ENSOARG00000008102; intron_9:8227661-8233291,exon_2:8233291-8233562,; LDM_E 2 76 CUEDC1

oar_circ_0000380 chr11 8345969 8347361 - 1392 1392 -- -- LDM_E,LDM_A 4,8 0,0 --

oar_circ_0000381 chr11 8937568 8938638 - 1070 1070 -- -- LDM_A 2 0 --

oar_circ_0000382 chr11 8973559 8978980 - 5421 5323 ENSOARG00000010417; exon_4:8975591-8976113,intron_5:8976113-8978882,exon_5:8973559-8973737,intron_4:8973737-8975591,; LDM_E 3 149 HSF5

oar_circ_0000383 chr11 9028666 9029338 - 672 461 ENSOARG00000010822; intron_8:9028790-9029251,; LDM_E 3 4 SEPT4

oar_circ_0000384 chr11 9222390 9222761 + 371 167 ENSOARG00000011574; exon_3:9222594-9222761,; LDM_A 2 3 RAD51C

oar_circ_0000385 chr11 9222594 9239228 + 16634 15531 ENSOARG00000011574; intron_5:9232587-9238391,intron_3:9222761-9224896,exon_3:9222594-9222761,intron_4:9225030-9232455,; LDM_E,LDM_A 19,16 408,72 RAD51C

oar_circ_0000386 chr11 9242453 9243131 + 678 678 ENSOARG00000011574; intron_7:9239228-9244883,; LDM_E 2 17 RAD51C

oar_circ_0000387 chr11 9555838 9562383 - 6545 5430 ENSOARG00000011845; exon_15:9561186-9561405,intron_10:9555975-9561186,; LDM_A 4 15 TRIM37

oar_circ_0000388 chr11 9608650 9625916 - 17266 16738 ENSOARG00000011845; intron_20:9624759-9625828,intron_17:9608775-9622110,intron_18:9622178-9623004,intron_19:9623128-9624636,; LDM_A 3 35 TRIM37

oar_circ_0000389 chr11 9647177 9657315 - 10138 7969 ENSOARG00000012076; exon_3:9647177-9647354,intron_2:9647354-9654961,intron_3:9655048-9655233,; LDM_E 2 411 SKA2

oar_circ_0000390 chr12 10307213 10343241 + 36028 34883 ENSOARG00000009587; intron_10:10338258-10343133,intron_3:10309359-10322567,intron_2:10307277-10309253,intron_7:10324631-10328597,intron_6:10323054-10324567,intron_8:10328700-10335324,intron_9:10335438-10338159,; LDM_E 4 990 UCHL5

oar_circ_0000391 chr12 10566656 10576468 + 9812 9812 -- -- LDM_E 2 0 --

oar_circ_0000392 chr12 10574294 10576468 + 2174 2174 -- -- LDM_A 6 0 --

oar_circ_0000393 chr12 1564408 1572324 + 7916 7558 ENSOARG00000002783; exon_7:1572163-1572324,intron_3:1564542-1565517,intron_4:1565573-1569156,intron_5:1569224-1569882,intron_6:1569982-1572163,; LDM_E,LDM_A 2,2 606,694 -

oar_circ_0000394 chr12 16944677 16958432 + 13755 13520 ENSOARG00000010181; intron_6:16944818-16945259,intron_7:16945353-16958241,exon_8:16958241-16958432,; LDM_A 2 153 KCTD3

oar_circ_0000395 chr12 18458685 18460813 - 2128 2128 -- -- LDM_A 2 0 --

oar_circ_0000396 chr12 18691704 18692014 - 310 310 -- -- LDM_A 2 0 --

oar_circ_0000397 chr12 19992275 20021404 + 29129 28761 ENSOARG00000011583; exon_2:19992275-19992439,exon_5:20019411-20019589,exon_6:20021247-20021404,intron_4:20017921-20019411,intron_5:20019589-20021247,intron_2:19992439-20017553,; LDM_E 6 2265 TGFB2

oar_circ_0000398 chr12 20017553 20019589 + 2036 1668 ENSOARG00000011583; intron_4:20017921-20019411,exon_5:20019411-20019589,; LDM_E,LDM_A 11,4 137,14 TGFB2

oar_circ_0000399 chr12 20017553 20021404 + 3851 3483 ENSOARG00000011583; exon_6:20021247-20021404,exon_5:20019411-20019589,intron_4:20017921-20019411,intron_5:20019589-20021247,; LDM_E,LDM_A 55,11 224,28 TGFB2

oar_circ_0000400 chr12 21637146 21641884 - 4738 4490 ENSOARG00000011829; intron_18:21637354-21638345,exon_15:21637146-21637354,intron_19:21638482-21641773,; LDM_A 5 111 EPRS

oar_circ_0000401 chr12 21647992 21650058 - 2066 2066 ENSOARG00000011829; intron_23:21648226-21649886,exon_9:21649886-21650058,exon_10:21647992-21648226,; LDM_E 3 146 EPRS

oar_circ_0000402 chr12 21752360 21756745 + 4385 3874 ENSOARG00000012333; exon_21:21756554-21756745,intron_17:21752486-21753097,intron_19:21754155-21755282,intron_18:21753229-21754048,intron_20:21755428-21756554,; LDM_E,LDM_A 25,11 142,89 IARS2

oar_circ_0000403 chr12 2246930 2249840 - 2910 2533 ENSOARG00000003554; intron_6:2249356-2249710,intron_4:2247044-2248640,exon_7:2249199-2249356,intron_5:2248773-2249199,; LDM_E 2 83 DSTYK

oar_circ_0000404 chr12 23315440 23315812 - 372 372 ENSOARG00000013213; exon_2:23315440-23315812,; LDM_A 6 119 DUSP10

oar_circ_0000405 chr12 24260253 24297165 - 36912 36316 ENSOARG00000013880; intron_4:24262483-24282246,intron_7:24288566-24288976,intron_8:24289030-24297095,intron_6:24282660-24288511,intron_3:24260376-24262360,intron_5:24282353-24282596,; LDM_E,LDM_A 9,7 1662,789 AIDA

oar_circ_0000406 chr12 24262360 24297165 - 34805 34332 ENSOARG00000013880; intron_7:24288566-24288976,intron_8:24289030-24297095,intron_4:24262483-24282246,intron_5:24282353-24282596,intron_6:24282660-24288511,; LDM_E,LDM_A 18,12 1608,754 AIDA

oar_circ_0000407 chr12 24282246 24297165 - 14919 14569 ENSOARG00000013880; intron_7:24288566-24288976,intron_8:24289030-24297095,intron_6:24282660-24288511,intron_5:24282353-24282596,; LDM_E,LDM_A 47,46 740,352 AIDA

oar_circ_0000408 chr12 24307725 24308298 + 573 573 -- -- LDM_A 2 0 --

oar_circ_0000409 chr12 24605526 24609457 + 3931 3483 ENSOARG00000014169; intron_5:24606604-24606964,intron_6:24607062-24609359,intron_4:24605650-24606476,; LDM_E,LDM_A 5,6 226,40 DISP1

oar_circ_0000410 chr12 25566747 25573148 - 6401 6104 ENSOARG00000016018; intron_12:25568783-25573003,exon_15:25566747-25566966,intron_10:25566966-25568463,exon_13:25568615-25568783,; LDM_E,LDM_A 2,3 131,63 NVL

oar_circ_0000411 chr12 25635144 25637667 + 2523 2269 ENSOARG00000016234; intron_3:25635257-25637526,; LDM_A 4 13 CNIH4

oar_circ_0000412 chr12 25659113 25675627 - 16514 15759 ENSOARG00000016377; intron_6:25665469-25668587,intron_4:25663019-25663310,intron_3:25662669-25662940,intron_5:25663456-25665349,intron_7:25668728-25675488,intron_2:25659299-25662539,exon_14:25659113-25659299,; LDM_A 2 707 WDR26

oar_circ_0000413 chr12 26691285 26692697 - 1412 1412 ENSOARG00000017329; exon_15:26691073-26694000,; LDM_A 2 2021 ENAH

oar_circ_0000414 chr12 26757010 26766292 - 9282 9282 ENSOARG00000017329; exon_1:26766126-26766294,exon_2:26757010-26757188,intron_14:26757188-26766126,; LDM_E,LDM_A 4,2 1041,246 ENAH

oar_circ_0000415 chr12 27018734 27021453 + 2719 2719 ENSOARG00000017468; intron_2:27018922-27021272,exon_3:27021272-27021453,exon_2:27018734-27018922,; LDM_A 14 35 EPHX1

oar_circ_0000416 chr12 27336704 27345330 - 8626 8485 ENSOARG00000000040; exon_5:27342166-27342325,intron_5:27342325-27345189,intron_3:27336891-27340277,exon_6:27340277-27340452,exon_7:27336704-27336891,intron_4:27340452-27342166,; LDM_E 6 419 ACBD3

oar_circ_0000417 chr12 27336704 27346189 - 9485 9202 ENSOARG00000000040; intron_5:27342325-27345189,intron_6:27345330-27346047,exon_5:27342166-27342325,intron_4:27340452-27342166,exon_6:27340277-27340452,exon_7:27336704-27336891,intron_3:27336891-27340277,; LDM_E 2 446 ACBD3

oar_circ_0000418 chr12 28143292 28149824 - 6532 6107 ENSOARG00000002215; intron_7:28147248-28149726,intron_5:28144198-28147163,intron_5:28143410-28144074,; LDM_E,LDM_A 2,2 179,60 CDC42BPA

oar_circ_0000419 chr12 28251645 28267902 - 16257 16054 ENSOARG00000002215; intron_28:28251768-28263772,intron_30:28267098-28267653,intron_29:28263939-28267018,exon_8:28267653-28267902,exon_10:28263772-28263939,; LDM_E 3 562 CDC42BPA

oar_circ_0000420 chr12 28263772 28267902 - 4130 4050 ENSOARG00000002215; exon_10:28263772-28263939,exon_8:28267653-28267902,intron_29:28263939-28267018,intron_30:28267098-28267653,; LDM_E,LDM_A 12,18 232,88 CDC42BPA

oar_circ_0000421 chr12 28263772 28270484 - 6712 6632 ENSOARG00000002215; exon_10:28263772-28263939,exon_8:28267653-28267902,intron_31:28267902-28270283,exon_7:28270283-28270484,intron_30:28267098-28267653,intron_29:28263939-28267018,; LDM_E,LDM_A 9,7 356,130 CDC42BPA

oar_circ_0000422 chr12 28597924 28617855 + 19931 19187 ENSOARG00000003217; intron_3:28601378-28604638,intron_6:28611370-28612667,intron_10:28616425-28616824,intron_4:28604819-28609302,exon_4:28604638-28604819,exon_10:28616243-28616425,intron_5:28609510-28611253,exon_8:28614089-28614240,intron_11:28616886-28617726,intron_7:28612752-28614089,exon_3:28601124-28601378,intron_2:28598052-28601124,exon_5:28609302-28609510,intron_8:28614240-28616020,; LDM_E 2 883 AHCTF1

oar_circ_0000423 chr12 28609302 28617855 + 8553 7937 ENSOARG00000003217; intron_7:28612752-28614089,intron_11:28616886-28617726,intron_5:28609510-28611253,exon_8:28614089-28614240,intron_6:28611370-28612667,exon_5:28609302-28609510,exon_10:28616243-28616425,intron_10:28616425-28616824,intron_8:28614240-28616020,; LDM_E,LDM_A 2,2 466,253 AHCTF1

oar_circ_0000424 chr12 29182774 29186339 + 3565 3262 ENSOARG00000004388; intron_4:29184447-29186202,intron_3:29182882-29184389,; LDM_E,LDM_A 8,6 40,13 SMYD3

oar_circ_0000425 chr12 29636292 29640903 + 4611 4348 ENSOARG00000004482; intron_7:29636380-29640728,; LDM_E 2 6 --

oar_circ_0000426 chr12 30359279 30359475 - 196 196 -- -- LDM_A 4 0 --

oar_circ_0000427 chr12 30584800 30587638 + 2838 2838 -- -- LDM_E 2 0 --

oar_circ_0000428 chr12 31585835 31588248 + 2413 2413 ENSOARG00000006076; intron_1:31520381-31644129,; LDM_E 5 171 AKT3

oar_circ_0000429 chr12 31644129 31679355 + 35226 34843 ENSOARG00000006076; intron_3:31673594-31679210,intron_2:31644255-31673482,; LDM_E,LDM_A 12,9 986,294 AKT3

oar_circ_0000430 chr12 31694661 31703451 + 8790 8523 ENSOARG00000006076; intron_5:31694793-31702855,intron_6:31702921-31703382,; LDM_A 3 129 AKT3

oar_circ_0000431 chr12 31855552 31867835 - 12283 11914 ENSOARG00000006316; intron_4:31865623-31867707,intron_3:31855684-31865514,; LDM_E,LDM_A 2,2 144,53 SDCCAG8

oar_circ_0000432 chr12 31855552 31874163 - 18611 18611 ENSOARG00000006316; intron_5:31867835-31901843,intron_4:31865623-31867707,intron_3:31855684-31865514,; LDM_A 3 68 SDCCAG8

oar_circ_0000433 chr12 31984424 31987914 - 3490 3425 ENSOARG00000006316; intron_11:31984613-31987849,exon_8:31984424-31984613,; LDM_A 3 13 SDCCAG8

oar_circ_0000434 chr12 31984424 31997327 - 12903 12697 ENSOARG00000006316; intron_12:31987914-31997186,intron_11:31984613-31987849,exon_8:31984424-31984613,; LDM_E,LDM_A 4,14 65,23 SDCCAG8

oar_circ_0000435 chr12 31984424 32003474 - 19050 18721 ENSOARG00000006316; exon_8:31984424-31984613,intron_12:31987914-31997186,intron_13:31997327-32003351,intron_11:31984613-31987849,; LDM_E,LDM_A 5,2 92,31 SDCCAG8

oar_circ_0000436 chr12 33439962 33447333 + 7371 7260 ENSOARG00000008730; exon_4:33441766-33441943,intron_3:33440073-33441766,exon_5:33444066-33444249,exon_6:33447167-33447333,intron_4:33441943-33444066,intron_5:33444249-33447167,; LDM_E,LDM_A 5,2 151,251 FH

oar_circ_0000437 chr12 33439962 33449067 + 9105 8994 ENSOARG00000008730; intron_3:33440073-33441766,intron_5:33444249-33447167,exon_4:33441766-33441943,intron_4:33441943-33444066,exon_6:33447167-33447333,exon_5:33444066-33444249,exon_7:33448863-33449067,intron_6:33447333-33448863,; LDM_E 2 206 FH

oar_circ_0000438 chr12 3448916 3449822 + 906 777 ENSOARG00000005343; exon_5:3448916-3449132,intron_5:3449132-3449693,; LDM_E 4 29 --

oar_circ_0000439 chr12 3448916 3462494 + 13578 13350 ENSOARG00000005343; exon_7:3457013-3457238,exon_9:3462296-3462494,intron_7:3457238-3461183,intron_5:3449132-3449693,intron_8:3461285-3462296,intron_6:3449822-3457016,exon_5:3448916-3449132,; LDM_E 3 560 --

oar_circ_0000440 chr12 35198078 35200512 + 2434 2305 ENSOARG00000009636; exon_6:35200292-35200512,intron_5:35198207-35200292,; LDM_A 3 112 BLZF1

oar_circ_0000441 chr12 35601241 35602114 - 873 873 ENSOARG00000005115; exon_1:35601641-35602763,; LDM_A 2 8 METTL18

oar_circ_0000442 chr12 35759579 35811814 - 52235 51598 ENSOARG00000011666; exon_14:35777318-35777484,intron_4:35770930-35772683,intron_6:35776704-35777318,exon_18:35759579-35759769,exon_10:35811651-35811814,intron_9:35779628-35787282,intron_7:35777484-35777928,intron_3:35759769-35770844,intron_10:35787383-35811651,intron_5:35772782-35776575,intron_8:35778041-35779519,; LDM_E 6 2011 KIFAP3

oar_circ_0000443 chr12 35772683 35824492 - 51809 51168 ENSOARG00000011666; intron_6:35776704-35777318,exon_14:35777318-35777484,intron_8:35778041-35779519,intron_5:35772782-35776575,exon_9:35818710-35818889,intron_10:35787383-35811651,intron_11:35811814-35818710,intron_7:35777484-35777928,intron_9:35779628-35787282,intron_12:35818889-35824402,exon_10:35811651-35811814,; LDM_E 8 2475 KIFAP3

oar_circ_0000444 chr12 35811651 35827930 - 16279 15949 ENSOARG00000011666; intron_13:35824510-35826314,intron_11:35811814-35818710,exon_10:35811651-35811814,intron_14:35826436-35827830,exon_9:35818710-35818889,intron_12:35818889-35824402,; LDM_E,LDM_A 7,6 1167,298 KIFAP3

oar_circ_0000445 chr12 35824402 35827930 - 3528 3198 ENSOARG00000011666; intron_14:35826436-35827830,intron_13:35824510-35826314,; LDM_E 2 265 KIFAP3

oar_circ_0000446 chr12 36002647 36012380 + 9733 9733 -- -- LDM_A 2 0 --

oar_circ_0000447 chr12 37600675 37602528 + 1853 1703 ENSOARG00000013843; intron_1:37600825-37602324,exon_2:37602324-37602528,; LDM_E 4 22 -

oar_circ_0000448 chr12 38188757 38206407 + 17650 17168 ENSOARG00000014227; intron_2:38188866-38190535,intron_6:38202188-38206283,intron_5:38201800-38202034,exon_4:38193003-38193164,intron_4:38193164-38201662,exon_6:38202034-38202188,intron_3:38190646-38193003,; LDM_E 2 872 SUCO

oar_circ_0000449 chr12 38188757 38208300 + 19543 18868 ENSOARG00000014227; intron_8:38207183-38208232,exon_4:38193003-38193164,intron_6:38202188-38206283,exon_6:38202034-38202188,intron_4:38193164-38201662,intron_5:38201800-38202034,intron_7:38206407-38207058,intron_2:38188866-38190535,intron_3:38190646-38193003,; LDM_E 7 1005 SUCO

oar_circ_0000450 chr12 38201662 38217046 + 15384 14715 ENSOARG00000014227; intron_7:38206407-38207058,intron_6:38202188-38206283,intron_8:38207183-38208232,intron_5:38201800-38202034,intron_9:38208300-38214859,intron_10:38214967-38216940,exon_6:38202034-38202188,; LDM_E 4 1141 SUCO

oar_circ_0000451 chr12 38214859 38233023 + 18164 17468 ENSOARG00000014227; exon_18:38232265-38233023,intron_15:38227532-38228474,intron_14:38221645-38227463,intron_13:38220637-38221579,exon_17:38231859-38232157,intron_11:38217046-38219774,intron_16:38228565-38231859,intron_10:38214967-38216940,intron_12:38219830-38220545,; LDM_E 3 1716 SUCO

oar_circ_0000452 chr12 38231859 38233023 + 1164 1056 ENSOARG00000014227; exon_18:38232265-38233023,exon_17:38231859-38232157,; LDM_E 4 451 SUCO

oar_circ_0000453 chr12 38231859 38242099 + 10240 10240 ENSOARG00000014227; intron_18:38233023-38239202,exon_17:38231859-38232157,intron_19:38239290-38243694,exon_18:38232265-38233023,; LDM_E 2 835 SUCO

oar_circ_0000454 chr12 38231859 38243799 + 11940 11639 ENSOARG00000014227; exon_17:38231859-38232157,intron_19:38239290-38243694,exon_18:38232265-38233023,intron_18:38233023-38239202,; LDM_E 2 868 SUCO

oar_circ_0000455 chr12 38231859 38246368 + 14509 14116 ENSOARG00000014227; intron_20:38243799-38245676,intron_18:38233023-38239202,intron_21:38245831-38246276,intron_19:38239290-38243694,exon_18:38232265-38233023,exon_17:38231859-38232157,exon_21:38245676-38245831,; LDM_E 2 946 SUCO

oar_circ_0000456 chr12 39292332 39294242 + 1910 1910 ENSOARG00000014744; intron_4:39288579-39294116,; LDM_E,LDM_A 3,8 18,8 DHRS3

oar_circ_0000457 chr12 39395923 39399231 - 3308 3175 ENSOARG00000015889; intron_4:39396119-39399098,exon_66:39395923-39396119,; LDM_E,LDM_A 14,5 73,33 VPS13D

oar_circ_0000458 chr12 39436382 39437819 - 1437 1302 ENSOARG00000015889; exon_64:39436382-39436550,intron_6:39436550-39437684,; LDM_A 5 14 VPS13D

oar_circ_0000459 chr12 39472807 39485291 - 12484 12120 ENSOARG00000015889; exon_53:39476169-39476405,intron_19:39482414-39485160,intron_18:39477321-39482238,intron_16:39472916-39476169,intron_17:39476405-39477197,exon_51:39482238-39482414,; LDM_E 3 393 VPS13D

oar_circ_0000460 chr12 39569401 39575394 - 5993 5522 ENSOARG00000015889; intron_54:39572569-39573746,intron_55:39573917-39574358,exon_15:39573746-39573917,intron_53:39569534-39572438,intron_56:39574434-39575263,; LDM_E 4 154 VPS13D

oar_circ_0000461 chr12 39577784 39580801 - 3017 2783 ENSOARG00000015889; exon_9:39580632-39580801,exon_11:39577784-39577986,intron_60:39578220-39580632,; LDM_E,LDM_A 5,11 71,64 VPS13D

oar_circ_0000462 chr12 39724057 39733545 - 9488 8816 ENSOARG00000017371; intron_8:39724210-39727828,intron_11:39730307-39733395,intron_10:39729845-39730213,intron_9:39727945-39729687,; LDM_E 2 31 TNFRSF8

oar_circ_0000463 chr12 39990299 39992692 - 2393 2133 ENSOARG00000018297; intron_15:39991336-39992624,intron_14:39990432-39991277,; LDM_E,LDM_A 4,2 52,18 CLCN6

oar_circ_0000464 chr12 40974590 40982522 + 7932 7932 -- -- LDM_E 3 0 --

oar_circ_0000465 chr12 41400826 41401568 + 742 742 -- -- LDM_A 2 0 --

oar_circ_0000466 chr12 41431476 41431665 - 189 189 -- -- LDM_E 2 0 --

oar_circ_0000467 chr12 41441709 41446565 - 4856 4600 ENSOARG00000005770; intron_43:41445805-41446386,intron_41:41441775-41442488,intron_42:41442566-41445693,exon_5:41446386-41446565,; LDM_E,LDM_A 2,2 1629,1703 KIF1B

oar_circ_0000468 chr12 41442488 41445805 - 3317 3127 ENSOARG00000005770; intron_42:41442566-41445693,; LDM_A 2 1399 KIF1B

oar_circ_0000469 chr12 41580093 41591118 - 11025 10653 ENSOARG00000006405; intron_29:41582517-41583806,intron_28:41580322-41582369,intron_30:41583894-41590982,exon_7:41580093-41580322,; LDM_E 2 421 UBE4B

oar_circ_0000470 chr12 41583806 41591118 - 7312 7088 ENSOARG00000006405; intron_30:41583894-41590982,; LDM_E 2 280 UBE4B

oar_circ_0000471 chr12 41793375 41799428 + 6053 5847 ENSOARG00000007130; intron_2:41793490-41799337,; LDM_A 2 17 CLSTN1

oar_circ_0000472 chr12 41901666 41903180 + 1514 1392 ENSOARG00000007130; intron_10:41901915-41903058,exon_10:41901666-41901915,; LDM_E 2 91 CLSTN1

oar_circ_0000473 chr12 42897046 42897515 + 469 469 ENSOARG00000009688; exon_1:42897046-42897515,; LDM_E 2 135 RERE

oar_circ_0000474 chr12 42986758 43003148 + 16390 16082 ENSOARG00000009688; intron_5:42988389-43003043,intron_4:42986864-42988292,; LDM_E,LDM_A 35,48 1775,218 RERE

oar_circ_0000475 chr12 43039822 43041132 + 1310 1085 ENSOARG00000009688; intron_8:43039947-43041032,; LDM_E 3 48 RERE

oar_circ_0000476 chr12 43824030 43830940 - 6910 6648 ENSOARG00000010701; intron_13:43824165-43830813,; LDM_E 2 4 CAMTA1

oar_circ_0000477 chr12 45213845 45214973 + 1128 1039 ENSOARG00000012693; intron_5:45213934-45214803,exon_6:45214803-45214973,; LDM_E 2 6 GPR153

oar_circ_0000478 chr12 4586358 4591798 + 5440 5254 ENSOARG00000007971; exon_6:4591609-4591798,intron_5:4587567-4591609,intron_4:4586458-4587481,; LDM_E 2 53 CD55

oar_circ_0000479 chr12 47376157 47379524 + 3367 3121 ENSOARG00000014909; intron_5:47376234-47379355,; LDM_E 2 56 CEP104

oar_circ_0000480 chr12 48940454 48941494 + 1040 1040 -- -- LDM_E 2 0 --

oar_circ_0000481 chr12 48963219 48968912 + 5693 5444 ENSOARG00000001850; intron_1:48963322-48967229,intron_2:48967268-48968805,; LDM_E 3 590 GNB1

oar_circ_0000482 chr12 48963219 48974016 + 10797 10484 ENSOARG00000001850; intron_2:48967268-48968805,intron_3:48968912-48972724,exon_5:48973853-48974016,intron_1:48963322-48967229,intron_4:48972788-48973853,; LDM_E,LDM_A 2,3 1329,403 GNB1

oar_circ_0000483 chr12 49765638 49769541 - 3903 3903 ENSOARG00000008477; exon_3:49765638-49766115,intron_15:49766115-49769220,exon_2:49769220-49769550,; LDM_A 2 586 SPEN

oar_circ_0000484 chr12 49960772 49964175 - 3403 3146 ENSOARG00000009189; intron_4:49960901-49964047,; LDM_E,LDM_A 2,4 247,162 DDI2

oar_circ_0000485 chr12 49960772 49972664 - 11892 11378 ENSOARG00000009189; intron_6:49967909-49969689,intron_5:49964175-49967782,intron_4:49960901-49964047,exon_3:49969689-49969926,intron_7:49969926-49972534,; LDM_E,LDM_A 2,5 823,456 DDI2

oar_circ_0000486 chr12 49967782 49972664 - 4882 4625 ENSOARG00000009189; exon_3:49969689-49969926,intron_6:49967909-49969689,intron_7:49969926-49972534,; LDM_E 2 319 DDI2

oar_circ_0000487 chr12 50032673 50040526 - 7853 7617 ENSOARG00000009524; exon_6:50039024-50039183,intron_10:50032804-50039024,intron_11:50039183-50040421,; LDM_E 2 47 DNAJC16

oar_circ_0000488 chr12 50053520 50055223 - 1703 1636 ENSOARG00000009524; intron_14:50053860-50055156,exon_3:50053520-50053860,; LDM_E 2 49 DNAJC16

oar_circ_0000489 chr12 50053520 50059807 - 6287 6220 ENSOARG00000009524; exon_3:50053520-50053860,intron_15:50055223-50059622,exon_1:50059622-50059807,intron_14:50053860-50055156,; LDM_E,LDM_A 3,2 139,78 DNAJC16

oar_circ_0000490 chr12 50075773 50082572 + 6799 6451 ENSOARG00000009701; intron_4:50075950-50076581,exon_7:50081667-50081847,intron_6:50077979-50081667,intron_5:50076671-50077831,intron_7:50081847-50082462,exon_4:50075773-50075950,; LDM_E 2 93 CASP9

oar_circ_0000491 chr12 50581393 50582320 - 927 927 -- -- LDM_A 2 0 --

oar_circ_0000492 chr12 52942499 52943967 - 1468 1468 -- -- LDM_E 2 0 --

oar_circ_0000493 chr12 53329457 53342256 - 12799 12542 ENSOARG00000013011; intron_8:53339026-53339581,intron_9:53339952-53341507,intron_6:53332388-53334450,exon_11:53339581-53339952,exon_10:53341507-53341722,exon_14:53332174-53332388,exon_13:53334450-53334603,intron_5:53331719-53332174,intron_3:53329631-53331373,intron_10:53341722-53342063,exon_12:53338858-53339026,exon_17:53329457-53329631,exon_9:53342063-53342345,intron_7:53334603-53338858,; LDM_A 2 736 RC3H1

oar_circ_0000494 chr12 53329457 53342345 - 12888 12542 ENSOARG00000013011; exon_11:53339581-53339952,intron_9:53339952-53341507,intron_6:53332388-53334450,exon_10:53341507-53341722,intron_8:53339026-53339581,exon_12:53338858-53339026,intron_7:53334603-53338858,exon_9:53342063-53342345,exon_17:53329457-53329631,exon_13:53334450-53334603,exon_14:53332174-53332388,intron_10:53341722-53342063,intron_5:53331719-53332174,intron_3:53329631-53331373,; LDM_E,LDM_A 2,2 697,802 RC3H1

oar_circ_0000495 chr12 53356765 53359372 - 2607 2486 ENSOARG00000013011; exon_4:53358100-53358276,intron_17:53358884-53359251,intron_15:53356966-53358100,exon_3:53358644-53358884,exon_5:53356765-53356966,intron_16:53358276-53358644,; LDM_E 5 134 RC3H1

oar_circ_0000496 chr12 53639830 53649256 + 9426 9256 ENSOARG00000013220; exon_9:53649089-53649256,intron_7:53639897-53645218,intron_8:53645321-53649089,; LDM_A 3 50 RABGAP1L

oar_circ_0000497 chr12 55270113 55276851 - 6738 6549 ENSOARG00000014362; intron_2:55273819-55276733,intron_1:55270274-55273694,exon_18:55270059-55270274,; LDM_E 5 372 RFWD2

oar_circ_0000498 chr12 55343201 55347754 - 4553 4302 ENSOARG00000014362; intron_8:55343337-55347639,; LDM_A 2 78 RFWD2

oar_circ_0000499 chr12 55343201 55361717 - 18516 18207 ENSOARG00000014362; intron_8:55343337-55347639,intron_9:55347754-55361659,; LDM_E,LDM_A 3,2 876,288 RFWD2

oar_circ_0000500 chr12 55404305 55405169 - 864 667 ENSOARG00000014362; intron_14:55404425-55405092,; LDM_E 4 12 RFWD2

oar_circ_0000501 chr12 55404305 55414729 - 10424 10129 ENSOARG00000014362; intron_14:55404425-55405092,intron_15:55405169-55414631,; LDM_E 2 219 RFWD2

oar_circ_0000502 chr12 55404305 55424229 - 19924 19569 ENSOARG00000014362; intron_16:55414729-55424169,intron_14:55404425-55405092,intron_15:55405169-55414631,; LDM_E 3 727 RFWD2

oar_circ_0000503 chr12 55948753 55949310 + 557 557 -- -- LDM_A 2 0 --

oar_circ_0000504 chr12 57605829 57606228 + 399 399 -- -- LDM_A 2 0 --

oar_circ_0000505 chr12 57907528 57907711 + 183 183 ENSOARG00000015973; intron_5:57907251-57929847,; LDM_E 3 2 RASAL2

oar_circ_0000506 chr12 57933357 57934561 + 1204 1105 ENSOARG00000015973; intron_7:57933456-57934006,exon_8:57934006-57934561,; LDM_A 2 84 RASAL2

oar_circ_0000507 chr12 58253668 58261056 + 7388 7087 ENSOARG00000016177; intron_2:58259661-58261005,intron_1:58253813-58259556,; LDM_E 3 642 RALGPS2

oar_circ_0000508 chr12 58259556 58277468 + 17912 17672 ENSOARG00000016177; intron_3:58261056-58277384,intron_2:58259661-58261005,; LDM_E 3 1175 RALGPS2

oar_circ_0000509 chr12 58271500 58295746 + 24246 24246 ENSOARG00000016177; intron_5:58286328-58290714,intron_6:58290807-58295619,intron_3:58261056-58277384,intron_4:58277468-58286238,; LDM_E 3 1874 RALGPS2

oar_circ_0000510 chr12 58277384 58295746 + 18362 17968 ENSOARG00000016177; intron_5:58286328-58290714,intron_6:58290807-58295619,intron_4:58277468-58286238,; LDM_E 7 1523 RALGPS2

oar_circ_0000511 chr12 58286238 58295746 + 9508 9198 ENSOARG00000016177; intron_5:58286328-58290714,intron_6:58290807-58295619,; LDM_E 2 794 RALGPS2

oar_circ_0000512 chr12 58474385 58480503 + 6118 6031 ENSOARG00000016354; exon_2:58474385-58474888,intron_2:58474888-58480416,; LDM_E 2 175 FAM20B

oar_circ_0000513 chr12 58551344 58554949 - 3605 3605 ENSOARG00000016598; intron_2:58552366-58554859,intron_1:58551578-58552192,exon_10:58549854-58551578,exon_9:58552192-58552366,; LDM_A 2 142 ABL2

oar_circ_0000514 chr12 58554859 58555971 - 1112 869 ENSOARG00000016598; intron_3:58554949-58555818,; LDM_A 2 48 ABL2

oar_circ_0000515 chr12 58558957 58560376 - 1419 1334 ENSOARG00000016598; intron_7:58559042-58560103,exon_3:58560103-58560376,; LDM_A 2 67 ABL2

oar_circ_0000516 chr12 59259959 59265726 + 5767 5495 ENSOARG00000017949; intron_2:59261140-59264610,intron_3:59264725-59265566,exon_4:59265566-59265726,intron_1:59260069-59261093,; LDM_E 3 421 CEP350

oar_circ_0000517 chr12 59259983 59265726 + 5743 5495 ENSOARG00000017949; intron_1:59260069-59261093,exon_4:59265566-59265726,intron_2:59261140-59264610,intron_3:59264725-59265566,; LDM_E,LDM_A 26,9 421,207 CEP350

oar_circ_0000518 chr12 59309624 59318925 + 9301 8786 ENSOARG00000017949; intron_17:59314174-59314639,exon_16:59310283-59310447,exon_15:59309624-59309839,intron_18:59314780-59317861,intron_16:59310447-59314060,intron_15:59309839-59310283,intron_19:59317998-59318802,; LDM_A 2 197 CEP350

oar_circ_0000519 chr12 61513907 61515755 - 1848 1848 -- -- LDM_E,LDM_A 9,3 0,0 --

oar_circ_0000520 chr12 61576846 61583963 - 7117 7117 ENSOARG00000018757; exon_1:61583748-61584551,intron_1:61575897-61583748,; LDM_A 3 4558 GLUL

oar_circ_0000521 chr12 61581696 61584543 + 2847 2847 -- -- LDM_A 2 0 --

oar_circ_0000522 chr12 61581712 61584559 + 2847 2847 -- -- LDM_A 3 0 --

oar_circ_0000523 chr12 61582967 61584564 - 1597 1597 ENSOARG00000018757; exon_1:61583748-61584551,intron_1:61575897-61583748,; LDM_A 2 5736 GLUL

oar_circ_0000524 chr12 61583206 61584564 - 1358 1358 ENSOARG00000018757; intron_1:61575897-61583748,exon_1:61583748-61584551,; LDM_A 32 5563 GLUL

oar_circ_0000525 chr12 62270559 62275141 + 4582 4034 ENSOARG00000019180; exon_25:62274941-62275141,intron_23:62273592-62273859,intron_21:62270697-62271211,intron_22:62271356-62273442,intron_24:62273974-62274941,; LDM_E,LDM_A 6,3 425,177 LAMC1

oar_circ_0000526 chr12 62270559 62276547 + 5988 5590 ENSOARG00000019180; intron_23:62273592-62273859,intron_24:62273974-62274941,exon_23:62273442-62273592,intron_22:62271356-62273442,exon_25:62274941-62275141,intron_21:62270697-62271211,exon_26:62276388-62276547,intron_25:62275141-62276388,; LDM_E 5 481 LAMC1

oar_circ_0000527 chr12 62273442 62273974 + 532 417 ENSOARG00000019180; intron_23:62273592-62273859,exon_23:62273442-62273592,; LDM_E 4 17 LAMC1

oar_circ_0000528 chr12 62678712 62679379 + 667 667 ENSOARG00000001352; intron_9:62678875-62679222,exon_10:62679222-62679379,exon_9:62678712-62678875,; LDM_E 5 18 SMG7

oar_circ_0000529 chr12 62773475 62773784 - 309 309 -- -- LDM_E,LDM_A 2,2 0,0 --

oar_circ_0000530 chr12 63017912 63035753 + 17841 17475 ENSOARG00000002070; intron_10:63032617-63035666,intron_9:63025344-63032527,intron_8:63021892-63025259,exon_7:63017912-63018128,intron_7:63018128-63021788,; LDM_E,LDM_A 4,2 560,192 RGL1

oar_circ_0000531 chr12 63088371 63115489 - 27118 26561 ENSOARG00000002398; intron_5:63088478-63091894,intron_6:63091971-63101264,intron_8:63108175-63113348,intron_7:63101384-63107970,intron_9:63113483-63115371,exon_5:63107970-63108175,; LDM_E 3 533 COLGALT2

oar_circ_0000532 chr12 63603127 63603292 - 165 165 -- -- LDM_A 2 0 --

oar_circ_0000533 chr12 63838568 63845268 - 6700 6700 ENSOARG00000002779; intron_3:63843769-63845076,intron_2:63838754-63843603,exon_17:63845076-63845268,exon_19:63838568-63838754,exon_18:63843603-63843769,; LDM_A 2 109 EDEM3

oar_circ_0000534 chr12 63843603 63857735 - 14132 14132 ENSOARG00000002779; intron_11:63855553-63856888,intron_5:63847542-63848419,exon_18:63843603-63843769,intron_9:63854175-63855140,exon_16:63847388-63847542,intron_3:63843769-63845076,intron_12:63856986-63862445,intron_7:63848985-63853427,exon_14:63848819-63848985,exon_17:63845076-63845268,intron_4:63845268-63847388,intron_8:63853552-63854091,; LDM_A 2 283 EDEM3

oar_circ_0000535 chr12 63845050 63845233 - 183 183 ENSOARG00000002779; exon_17:63845076-63845268,; LDM_E 2 2 EDEM3

oar_circ_0000536 chr12 64231122 64247269 - 16147 15523 ENSOARG00000003786; intron_7:64232920-64233321,exon_8:64233321-64233571,intron_10:64238737-64240123,exon_10:64231122-64231313,intron_6:64231313-64232707,intron_9:64238137-64238613,intron_12:64241889-64245279,intron_8:64233571-64238057,intron_13:64245393-64247158,exon_9:64232707-64232920,intron_11:64240253-64241824,; LDM_E 2 756 TRMT1L

oar_circ_0000537 chr12 64284891 64296238 + 11347 10804 ENSOARG00000004156; intron_14:64294996-64296099,intron_12:64284985-64293214,intron_13:64293360-64294832,; LDM_A 3 61 SWT1

oar_circ_0000538 chr12 64368718 64370613 - 1895 1565 ENSOARG00000004429; intron_12:64369089-64369310,exon_1:64370391-64370613,exon_3:64368919-64369089,intron_13:64369439-64370391,; LDM_E,LDM_A 3,2 106,348 IVNS1ABP

oar_circ_0000539 chr12 65520583 65524045 - 3462 3199 ENSOARG00000006631; exon_31:65520583-65520794,intron_22:65521729-65523913,intron_21:65520794-65521598,; LDM_A 2 68 TPR

oar_circ_0000540 chr12 65520583 65531104 - 10521 9640 ENSOARG00000006631; exon_25:65526717-65526921,intron_30:65529875-65530938,intron_22:65521729-65523913,intron_24:65524285-65525862,intron_28:65528566-65528992,intron_21:65520794-65521598,exon_21:65530938-65531104,exon_31:65520583-65520794,exon_22:65529708-65529875,intron_25:65526010-65526419,intron_27:65526921-65528449,exon_23:65528992-65529145,exon_26:65526419-65526604,intron_29:65529145-65529708,; LDM_E 2 404 TPR

oar_circ_0000541 chr12 65559197 65570278 + 11081 10444 ENSOARG00000007338; intron_4:65568027-65569333,intron_2:65562029-65565271,intron_3:65565367-65567920,intron_1:65559318-65561894,intron_5:65569370-65570137,; LDM_E 4 269 C1orf27

oar_circ_0000542 chr12 65567920 65582737 + 14817 14147 ENSOARG00000007338; intron_8:65572639-65577735,intron_9:65577864-65578653,intron_6:65570278-65571638,intron_5:65569370-65570137,intron_10:65578744-65582551,intron_4:65568027-65569333,exon_11:65582551-65582737,intron_7:65571734-65572570,; LDM_E 2 262 C1orf27

oar_circ_0000543 chr12 65578653 65582737 + 4084 3993 ENSOARG00000007338; exon_11:65582551-65582737,intron_10:65578744-65582551,; LDM_E,LDM_A 17,4 98,43 C1orf27

oar_circ_0000544 chr12 66151581 66159353 + 7772 7772 ENSOARG00000007935; exon_14:66151581-66151824,intron_14:66151824-66159168,exon_15:66159168-66159353,; LDM_E 3 42 PLA2G4A

oar_circ_0000545 chr12 66536888 66539326 - 2438 2090 ENSOARG00000008173; intron_3:66537075-66539165,; LDM_A 2 1 KCNK2

oar_circ_0000546 chr12 67241594 67249740 + 8146 8146 ENSOARG00000008871; exon_4:67249570-67249740,intron_3:67238293-67249570,; LDM_A 2 343 PTPN14

oar_circ_0000547 chr12 67326170 67329821 + 3651 3572 ENSOARG00000008871; intron_14:67326249-67328346,exon_15:67328346-67329821,; LDM_E 2 269 PTPN14

oar_circ_0000548 chr12 67382822 67386461 - 3639 3342 ENSOARG00000009207; intron_8:67382947-67383664,intron_9:67383725-67386350,; LDM_A 2 65 -

oar_circ_0000549 chr12 67696151 67711004 - 14853 14747 ENSOARG00000009324; intron_2:67696346-67702288,exon_4:67696151-67696346,exon_2:67709210-67711004,intron_3:67702394-67709210,; LDM_E 3 531 PROX1

oar_circ_0000550 chr12 68598407 68650271 - 51864 51533 ENSOARG00000009437; intron_11:68613080-68624137,intron_10:68598770-68612986,exon_6:68598407-68598770,intron_12:68624253-68650150,; LDM_E 14 1146 RPS6KC1

oar_circ_0000551 chr12 68612986 68650271 - 37285 36954 ENSOARG00000009437; intron_11:68613080-68624137,intron_12:68624253-68650150,; LDM_E 18 804 RPS6KC1

oar_circ_0000552 chr12 68716250 68720208 + 3958 3773 ENSOARG00000009633; intron_6:68716672-68717566,exon_9:68720044-68720208,exon_6:68716250-68716672,intron_8:68718142-68720044,intron_7:68717693-68718084,; LDM_E,LDM_A 128,41 382,173 ANGEL2

oar_circ_0000553 chr12 69638335 69654564 + 16229 15994 ENSOARG00000011102; intron_7:69639262-69654446,exon_6:69638329-69638535,intron_6:69638535-69639139,; LDM_A 2 105 INTS7

oar_circ_0000554 chr12 69817014 69828988 + 11974 11632 ENSOARG00000011368; exon_6:69825261-69825535,intron_6:69825535-69828861,intron_5:69822544-69825261,intron_4:69817133-69822448,; LDM_E,LDM_A 3,7 300,323 LPGAT1

oar_circ_0000555 chr12 71608353 71611831 - 3478 3438 ENSOARG00000012524; intron_9:71608524-71610217,intron_10:71610458-71611791,exon_4:71610217-71610458,exon_5:71608353-71608524,; LDM_A 2 25 DIEXF

oar_circ_0000556 chr12 73261009 73268453 + 7444 7444 ENSOARG00000013487; exon_1:73261009-73262277,exon_2:73268270-73268453,intron_1:73262277-73268270,; LDM_E 14 810 PLXNA2

oar_circ_0000557 chr12 73587832 73588014 - 182 182 -- -- LDM_A 2 0 --

oar_circ_0000558 chr12 74155362 74155557 - 195 195 ENSOARG00000014713; exon_24:74155362-74155557,; LDM_E 3 8 ASPM

oar_circ_0000559 chr12 74165459 74168186 - 2727 2454 ENSOARG00000014713; intron_23:74166534-74168044,exon_17:74165459-74165635,intron_22:74165635-74166403,; LDM_E 5 23 ASPM

oar_circ_0000560 chr12 74629369 74640629 - 11260 10894 ENSOARG00000015345; intron_5:74630390-74631766,intron_6:74631835-74632559,intron_7:74632600-74640538,intron_4:74629477-74630333,; LDM_A 2 115 DENND1B

oar_circ_0000561 chr12 74673058 74696043 - 22985 22610 ENSOARG00000015345; intron_12:74689586-74695942,intron_11:74684922-74689540,intron_10:74673184-74684820,; LDM_E 4 426 DENND1B

oar_circ_0000562 chr12 75234430 75242661 + 8231 7916 ENSOARG00000015482; intron_2:75234571-75240991,intron_3:75241054-75242550,; LDM_E 7 363 NEK7

oar_circ_0000563 chr12 77366771 77370846 - 4075 3568 ENSOARG00000016116; intron_11:77367509-77369186,intron_12:77369313-77370693,intron_10:77366904-77367415,; LDM_E 6 40 KIF14

oar_circ_0000564 chr12 77415379 77421406 - 6027 5655 ENSOARG00000016307; intron_4:77417225-77421316,intron_2:77415508-77416056,exon_5:77416973-77417225,intron_3:77416209-77416973,; LDM_E 2 106 DDX59

oar_circ_0000565 chr12 77543279 77553842 + 10563 10563 ENSOARG00000016523; intron_4:77546205-77556576,intron_3:77543441-77546121,exon_3:77543279-77543441,; LDM_E 3 949 CAMSAP2

oar_circ_0000566 chr12 77543279 77557176 + 13897 13531 ENSOARG00000016523; intron_4:77546205-77556576,intron_5:77556718-77557036,intron_3:77543441-77546121,exon_3:77543279-77543441,; LDM_E 3 1331 CAMSAP2

oar_circ_0000567 chr12 77973957 77975908 + 1951 1951 ENSOARG00000018166; exon_4:77974692-77974879,exon_5:77975723-77975908,intron_4:77974879-77975723,exon_3:77973957-77974129,intron_3:77974129-77974692,; LDM_A 4 80 ADIPOR1

oar_circ_0000568 chr12 78075426 78078654 + 3228 3228 ENSOARG00000018421; exon_4:78078483-78078654,intron_3:78077700-78078483,intron_2:78076276-78077577,intron_1:78045061-78076198,; LDM_E 3 367 KDM5B

oar_circ_0000569 chr12 78076198 78078654 + 2456 2255 ENSOARG00000018421; intron_2:78076276-78077577,intron_3:78077700-78078483,exon_4:78078483-78078654,; LDM_E 4 329 KDM5B

oar_circ_0000570 chr12 922515 923947 + 1432 1312 ENSOARG00000001858; intron_2:922715-923827,exon_2:922515-922715,; LDM_E,LDM_A 9,9 211,97 ZC3H11A

oar_circ_0000571 chr13 10975746 10978755 - 3009 2665 ENSOARG00000013242; intron_13:10977234-10978592,intron_11:10975876-10977020,exon_4:10978592-10978755,; LDM_A 3 95 PRKCQ

oar_circ_0000572 chr13 10987112 10996092 - 8980 8789 ENSOARG00000013242; exon_2:10991185-10991385,intron_15:10987177-10991185,intron_16:10991385-10995966,; LDM_A 2 140 PRKCQ

oar_circ_0000573 chr13 11529432 11540165 - 10733 10546 ENSOARG00000013446; exon_5:11538162-11538409,intron_17:11538409-11540076,intron_16:11529530-11538162,; LDM_E,LDM_A 2,4 381,135 SFMBT2

oar_circ_0000574 chr13 11589922 11599244 - 9322 9076 ENSOARG00000013446; intron_20:11593536-11599093,intron_19:11590163-11593441,exon_3:11589922-11590163,; LDM_E,LDM_A 5,3 72,36 SFMBT2

oar_circ_0000575 chr13 15522066 15523703 + 1637 1417 ENSOARG00000014323; intron_1:15522188-15523605,; LDM_E 2 45 ECHDC3

oar_circ_0000576 chr13 15666629 15672469 - 5840 5636 ENSOARG00000014587; exon_18:15666629-15666797,intron_5:15672072-15672364,intron_4:15666797-15671973,; LDM_A 2 96 UPF2

oar_circ_0000577 chr13 15721099 15729230 - 8131 7941 ENSOARG00000014587; intron_15:15721185-15723759,intron_17:15725262-15725722,exon_3:15729069-15729230,exon_5:15725112-15725262,intron_16:15723863-15725112,intron_18:15725920-15729069,exon_4:15725722-15725920,; LDM_E,LDM_A 4,8 264,130 UPF2

oar_circ_0000578 chr13 1577838 1587828 + 9990 9990 ENSOARG00000009536; intron_1:1577950-1676305,; LDM_E,LDM_A 3,4 481,98 PLCB4

oar_circ_0000579 chr13 15785021 15791533 + 6512 6136 ENSOARG00000014833; exon_7:15786381-15786580,exon_8:15788200-15788513,intron_8:15788513-15789806,intron_9:15789891-15790398,intron_7:15786580-15788200,intron_10:15790538-15791382,intron_6:15785193-15786381,exon_6:15785021-15785193,; LDM_E,LDM_A 2,3 81,49 DHTKD1

oar_circ_0000580 chr13 15868749 15898518 + 29769 29022 ENSOARG00000015281; intron_8:15880744-15882141,intron_11:15892202-15898380,intron_10:15883201-15892073,intron_9:15882264-15883172,intron_6:15871176-15880250,intron_7:15880299-15880668,intron_5:15868845-15871069,; LDM_E 3 637 CDC123

oar_circ_0000581 chr13 1632043 1632198 + 155 155 -- -- LDM_E 2 0 --

oar_circ_0000582 chr13 16747679 16754570 - 6891 6328 ENSOARG00000015762; intron_16:16748110-16748567,intron_17:16748730-16754438,exon_3:16748567-16748730,; LDM_E,LDM_A 4,6 269,141 YME1L1

oar_circ_0000583 chr13 16959733 16964151 - 4418 4101 ENSOARG00000016202; intron_9:16962971-16964086,intron_8:16959864-16962850,; LDM_E,LDM_A 4,4 9,3 PDSS1

oar_circ_0000584 chr13 16974665 16991220 + 16555 13935 ENSOARG00000016337; intron_4:16976495-16977129,intron_16:16986635-16990070,intron_2:16974784-16975412,intron_10:16981927-16982669,intron_7:16977531-16978151,intron_3:16975538-16976492,intron_12:16984289-16985080,intron_13:16985210-16986205,intron_11:16982742-16984205,intron_9:16979210-16981857,intron_17:16990150-16991176,; LDM_E 2 242 -

oar_circ_0000585 chr13 17258170 17270110 - 11940 11222 ENSOARG00000016854; intron_5:17258354-17261264,intron_6:17261269-17266555,intron_8:17266937-17269963,; LDM_E 2 231 CREM

oar_circ_0000586 chr13 17266555 17270110 - 3555 3026 ENSOARG00000016854; intron_8:17266937-17269963,; LDM_E,LDM_A 4,8 45,67 CREM

oar_circ_0000587 chr13 17591302 17603848 + 12546 12546 ENSOARG00000017196; intron_1:17591404-17717065,; LDM_A 3 629 PARD3

oar_circ_0000588 chr13 17717065 17719702 + 2637 2637 ENSOARG00000017196; intron_2:17717243-17754745,exon_2:17717065-17717243,; LDM_E 3 260 PARD3

oar_circ_0000589 chr13 17717065 17754921 + 37856 37856 ENSOARG00000017196; intron_2:17717243-17754745,exon_3:17754745-17754921,exon_2:17717065-17717243,; LDM_E 4 2059 PARD3

oar_circ_0000590 chr13 17850442 17870238 + 19796 19645 ENSOARG00000017196; exon_15:17867371-17867561,intron_13:17852392-17860768,exon_13:17852221-17852392,exon_16:17870086-17870238,intron_12:17850631-17852221,intron_15:17867561-17870086,exon_12:17850442-17850631,intron_14:17860919-17867371,; LDM_E 2 1356 PARD3

oar_circ_0000591 chr13 17920542 17930781 + 10239 10128 ENSOARG00000017196; intron_20:17920653-17930538,exon_21:17930538-17930781,; LDM_E,LDM_A 2,2 708,210 PARD3

oar_circ_0000592 chr13 19007387 19011018 + 3631 3445 ENSOARG00000017638; exon_6:19009648-19009804,intron_6:19009804-19010542,exon_5:19007387-19007626,intron_7:19010638-19010928,intron_5:19007626-19009648,; LDM_E,LDM_A 3,7 599,340 ITGB1

oar_circ_0000593 chr13 1944447 1951354 + 6907 6613 ENSOARG00000009536; intron_33:1945895-1947650,intron_32:1944563-1945819,intron_34:1947826-1951252,exon_34:1947650-1947826,; LDM_E 3 182 PLCB4

oar_circ_0000594 chr13 20547556 20555501 + 7945 7945 ENSOARG00000000344; exon_2:20555289-20555501,intron_1:20373008-20555289,; LDM_A 2 76 PLXDC2

oar_circ_0000595 chr13 20678019 20684068 + 6049 5807 ENSOARG00000000344; intron_5:20678142-20683949,; LDM_E,LDM_A 2,3 211,56 PLXDC2

oar_circ_0000596 chr13 20759613 20779422 + 19809 19619 ENSOARG00000000344; intron_11:20759764-20761193,exon_13:20779261-20779422,intron_12:20761232-20779261,; LDM_E,LDM_A 2,2 507,206 PLXDC2

oar_circ_0000597 chr13 21914622 21944546 + 29924 29534 ENSOARG00000000698; intron_3:21914726-21916805,intron_4:21916899-21918786,intron_5:21918882-21944450,; LDM_E 4 1730 MLLT10

oar_circ_0000598 chr13 22011468 22040665 + 29197 28493 ENSOARG00000000698; intron_17:22031986-22032986,intron_19:22038758-22040471,intron_15:22030985-22031402,exon_19:22038539-22038758,intron_12:22025349-22026715,exon_17:22031624-22031986,intron_14:22029002-22030796,exon_11:22011468-22011647,exon_15:22030796-22030985,exon_14:22028847-22029002,intron_18:22033083-22038539,intron_13:22026788-22028847,intron_11:22011647-22025231,; LDM_E 5 1611 MLLT10

oar_circ_0000599 chr13 24131471 24131755 + 284 284 ENSOARG00000002785; exon_2:24131471-24131755,; LDM_E 2 98 KIAA1217

oar_circ_0000600 chr13 24468864 24475372 + 6508 6075 ENSOARG00000002785; intron_19:24474736-24475249,exon_19:24473134-24474736,intron_17:24468984-24472944,; LDM_A 3 1118 KIAA1217

oar_circ_0000601 chr13 28526355 28533421 + 7066 6655 ENSOARG00000007085; intron_5:28526461-28527380,intron_6:28527477-28528442,intron_8:28529762-28531362,exon_9:28531362-28531518,intron_9:28531518-28533318,exon_8:28529600-28529762,intron_7:28528547-28529600,; LDM_A 2 98 HSPA14

oar_circ_0000602 chr13 28587282 28598445 - 11163 10065 ENSOARG00000007601; intron_6:28587419-28593914,intron_7:28594016-28597586,; LDM_E,LDM_A 5,4 44,28 DCLRE1C

oar_circ_0000603 chr13 28736335 28744636 - 8301 7486 ENSOARG00000008019; intron_10:28739739-28744500,intron_9:28736869-28739594,; LDM_A 3 72 NMT2

oar_circ_0000604 chr13 28864782 28871043 - 6261 6169 ENSOARG00000008187; intron_7:28864875-28870815,exon_1:28870815-28871044,; LDM_E 6 57 FAM171A1

oar_circ_0000605 chr13 29389019 29440886 - 51867 51180 ENSOARG00000008810; intron_7:29414561-29427282,intron_8:29427362-29428233,intron_13:29436937-29440806,exon_4:29435068-29435242,intron_11:29431836-29435068,intron_12:29435242-29436876,intron_5:29389092-29409257,intron_10:29430911-29431784,intron_9:29428307-29430796,intron_6:29409338-29414490,; LDM_E,LDM_A 6,6 2344,841 FAM188A

oar_circ_0000606 chr13 29409257 29440886 - 31629 31015 ENSOARG00000008810; intron_12:29435242-29436876,intron_11:29431836-29435068,exon_4:29435068-29435242,intron_6:29409338-29414490,intron_10:29430911-29431784,intron_9:29428307-29430796,intron_7:29414561-29427282,intron_8:29427362-29428233,intron_13:29436937-29440806,; LDM_E,LDM_A 18,8 1431,437 FAM188A

oar_circ_0000607 chr13 30289693 30300198 - 10505 10067 ENSOARG00000009068; intron_5:30292610-30300077,intron_3:30289808-30290083,intron_4:30290166-30292491,; LDM_E,LDM_A 4,3 332,100 RSU1

oar_circ_0000608 chr13 30658575 30686014 - 27439 26772 ENSOARG00000011090; exon_8:30661861-30662190,intron_10:30668617-30685904,intron_7:30664737-30665428,intron_6:30664156-30664667,intron_3:30658705-30659740,intron_8:30665494-30666113,intron_9:30666185-30668540,intron_4:30659798-30661861,intron_5:30662190-30664072,; LDM_E 6 1071 TRDMT1

oar_circ_0000609 chr13 31153804 31163353 - 9549 9021 ENSOARG00000011587; intron_3:31161358-31162867,intron_2:31156565-31161236,exon_5:31156386-31156565,intron_5:31163055-31163235,intron_1:31153904-31156386,; LDM_E 6 611 HACD1

oar_circ_0000610 chr13 31161236 31163353 - 2117 1509 ENSOARG00000011587; intron_3:31161358-31162867,; LDM_E,LDM_A 6,6 56,6 HACD1

oar_circ_0000611 chr13 31162867 31163353 - 486 180 ENSOARG00000011587; intron_5:31163055-31163235,; LDM_E 3 1 HACD1

oar_circ_0000612 chr13 31291294 31297994 + 6700 6485 ENSOARG00000011778; intron_2:31291367-31297852,; LDM_E,LDM_A 5,7 5,15 TMEM236

oar_circ_0000613 chr13 32022516 32028324 + 5808 5471 ENSOARG00000012882; intron_4:32025201-32028247,intron_3:32022639-32025064,; LDM_E 6 313 CACNB2

oar_circ_0000614 chr13 32022516 32048905 + 26389 25668 ENSOARG00000012882; intron_5:32028324-32034855,intron_6:32034989-32038688,intron_4:32025201-32028247,intron_3:32022639-32025064,intron_7:32038769-32039231,intron_8:32039290-32048795,; LDM_E 4 1101 CACNB2

oar_circ_0000615 chr13 32105474 32110788 - 5314 5112 ENSOARG00000013212; intron_6:32105594-32110706,; LDM_A 3 19 NSUN6

oar_circ_0000616 chr13 32129748 32144957 - 15209 14865 ENSOARG00000013212; intron_8:32129902-32130780,intron_9:32130890-32144877,; LDM_E,LDM_A 4,3 201,43 NSUN6

oar_circ_0000617 chr13 32151040 32153046 - 2006 2006 ENSOARG00000013212; intron_11:32150277-32153210,; LDM_E 2 14 NSUN6

oar_circ_0000618 chr13 32285683 32287398 + 1715 1310 ENSOARG00000013354; intron_6:32285843-32286816,exon_6:32285683-32285843,exon_7:32286816-32286993,; LDM_E,LDM_A 3,4 118,65 EPC1

oar_circ_0000619 chr13 32298292 32298903 + 611 364 ENSOARG00000013354; exon_13:32298539-32298903,; LDM_A 2 57 EPC1

oar_circ_0000620 chr13 32518847 32519611 + 764 764 ENSOARG00000013756; exon_2:32518847-32519611,; LDM_E 2 250 ARHGAP12

oar_circ_0000621 chr13 32560216 32560480 + 264 264 ENSOARG00000013756; exon_3:32560216-32560480,; LDM_E 2 34 ARHGAP12

oar_circ_0000622 chr13 32560216 32568995 + 8779 8557 ENSOARG00000013756; intron_4:32567732-32568914,intron_3:32560480-32567591,exon_3:32560216-32560480,; LDM_E,LDM_A 3,3 329,107 ARHGAP12

oar_circ_0000623 chr13 32560216 32577326 + 17110 16762 ENSOARG00000013756; intron_5:32568995-32577200,exon_3:32560216-32560480,intron_4:32567732-32568914,intron_3:32560480-32567591,; LDM_E 2 1152 ARHGAP12

oar_circ_0000624 chr13 32560216 32588299 + 28083 27598 ENSOARG00000013756; intron_6:32577326-32580242,intron_3:32560480-32567591,intron_4:32567732-32568914,exon_3:32560216-32560480,intron_5:32568995-32577200,intron_7:32580317-32588237,; LDM_E 3 1994 ARHGAP12

oar_circ_0000625 chr13 32567591 32588299 + 20708 20223 ENSOARG00000013756; intron_4:32567732-32568914,intron_6:32577326-32580242,intron_7:32580317-32588237,intron_5:32568995-32577200,; LDM_E 4 1714 ARHGAP12

oar_circ_0000626 chr13 32856874 32858685 - 1811 1811 ENSOARG00000013971; exon_7:32856874-32858685,; LDM_E 6 371 ZEB1

oar_circ_0000627 chr13 32866943 32871042 - 4099 3993 ENSOARG00000013971; exon_5:32870839-32871042,intron_3:32867049-32870839,; LDM_A 2 92 ZEB1

oar_circ_0000628 chr13 32866943 32908013 - 41070 40901 ENSOARG00000013971; exon_5:32870839-32871042,exon_2:32907812-32908013,exon_4:32879322-32879484,intron_5:32879484-32886849,intron_4:32871042-32879322,intron_6:32886912-32907812,intron_3:32867049-32870839,; LDM_E,LDM_A 25,10 2816,675 ZEB1

oar_circ_0000629 chr13 32879322 32908013 - 28691 28628 ENSOARG00000013971; exon_2:32907812-32908013,exon_4:32879322-32879484,intron_5:32879484-32886849,intron_6:32886912-32907812,; LDM_E 15 2145 ZEB1

oar_circ_0000630 chr13 32907812 33001090 - 93278 93278 ENSOARG00000013971; exon_2:32907812-32908013,intron_7:32908013-33050405,; LDM_E,LDM_A 20,17 11881,3369 ZEB1

oar_circ_0000631 chr13 32987271 33001090 - 13819 13819 ENSOARG00000013971; intron_7:32908013-33050405,; LDM_E,LDM_A 218,131 3030,1017 ZEB1

oar_circ_0000632 chr13 33202507 33207979 + 5472 5472 -- -- LDM_E 4 0 --

oar_circ_0000633 chr13 3332762 3342183 + 9421 6632 ENSOARG00000010370; intron_1:3335430-3342062,; LDM_A 2 14 SLX4IP

oar_circ_0000634 chr13 33843145 33869527 - 26382 26382 -- -- LDM_E,LDM_A 3,5 0,0 --

oar_circ_0000635 chr13 34331570 34344788 - 13218 13218 ENSOARG00000014807; exon_2:34344615-34344788,intron_4:34331797-34334396,exon_6:34331570-34331797,exon_4:34342035-34342260,exon_5:34334396-34334608,intron_7:34344094-34344615,intron_5:34334608-34342035,exon_3:34343860-34344094,intron_6:34342260-34343860,; LDM_E 2 239 MTPAP

oar_circ_0000636 chr13 35185056 35194954 - 9898 9562 ENSOARG00000015283; intron_8:35185365-35189769,intron_10:35190578-35194847,exon_5:35185056-35185365,intron_9:35189882-35190462,; LDM_E,LDM_A 3,3 2052,999 WAC

oar_circ_0000637 chr13 35189769 35194954 - 5185 4849 ENSOARG00000015283; intron_10:35190578-35194847,intron_9:35189882-35190462,; LDM_E,LDM_A 2,3 1043,458 WAC

oar_circ_0000638 chr13 35217740 35218030 - 290 290 ENSOARG00000015283; intron_11:35194954-35237566,; LDM_E 2 14 WAC

oar_circ_0000639 chr13 35767005 35777353 + 10348 9513 ENSOARG00000015684; intron_11:35767784-35772525,exon_12:35772525-35772768,intron_14:35776108-35777110,exon_15:35777110-35777353,exon_11:35767574-35767784,intron_13:35773452-35775953,intron_12:35772768-35773341,; LDM_E 2 103 ARMC4

oar_circ_0000640 chr13 35801790 35818911 + 17121 17006 ENSOARG00000015684; exon_17:35816582-35816771,intron_16:35801905-35816582,exon_18:35818689-35818911,intron_17:35816771-35818689,; LDM_E 3 79 ARMC4

oar_circ_0000641 chr13 35816582 35818911 + 2329 2329 ENSOARG00000015684; exon_17:35816582-35816771,exon_18:35818689-35818911,intron_17:35816771-35818689,; LDM_E 2 20 ARMC4

oar_circ_0000642 chr13 37551296 37591457 + 40161 40161 ENSOARG00000018436; intron_3:37547743-37594922,; LDM_E 10 351 ZNF133

oar_circ_0000643 chr13 38335676 38340013 + 4337 4073 ENSOARG00000001399; intron_5:38339162-38339909,intron_4:38335751-38339077,; LDM_E 2 49 SLC24A3

oar_circ_0000644 chr13 39752996 39763249 + 10253 9587 ENSOARG00000004443; intron_23:39754395-39761009,intron_25:39761309-39763118,intron_22:39753778-39754338,intron_21:39753094-39753698,; LDM_E 2 246 XRN2

oar_circ_0000645 chr13 42072068 42074566 - 2498 2112 ENSOARG00000008366; exon_23:42072068-42072250,intron_4:42073758-42074440,intron_2:42072250-42072681,intron_3:42072787-42073604,; LDM_A 2 65 FAM208B

oar_circ_0000646 chr13 42072068 42081562 - 9494 9262 ENSOARG00000008366; exon_18:42081138-42081562,exon_23:42072068-42072250,intron_2:42072250-42072681,intron_6:42080558-42081138,intron_3:42072787-42073604,exon_19:42076805-42080558,intron_5:42074566-42076805,exon_21:42073604-42073758,intron_4:42073758-42074440,; LDM_E 2 1328 FAM208B

oar_circ_0000647 chr13 42091604 42113242 - 21638 20082 ENSOARG00000008366; intron_13:42095092-42095965,intron_18:42104771-42107271,intron_19:42107363-42111565,intron_12:42094097-42094988,intron_10:42092324-42093863,intron_20:42111638-42112709,intron_21:42112805-42113096,exon_15:42091604-42092324,intron_14:42096066-42104061,; LDM_A 3 557 FAM208B

oar_circ_0000648 chr13 42104061 42113242 - 9181 8064 ENSOARG00000008366; intron_19:42107363-42111565,intron_18:42104771-42107271,intron_20:42111638-42112709,intron_21:42112805-42113096,; LDM_E,LDM_A 16,4 579,297 FAM208B

oar_circ_0000649 chr13 42111565 42113242 - 1677 1362 ENSOARG00000008366; intron_21:42112805-42113096,intron_20:42111638-42112709,; LDM_E,LDM_A 2,3 97,56 FAM208B

oar_circ_0000650 chr13 42418511 42477951 + 59440 22236 ENSOARG00000009642;ENSOARG00000009762;ENSOARG00000010028;ENSOARG00000009277; intron_10:42437555-42439176,intron_1:42430006-42433264,exon_10:42437389-42437555,;intron_9:42461799-42464499,intron_8:42459459-42461716,;intron_4:42475320-42475741,exon_2:42473339-42473507,intron_2:42473507-42473832,intron_1:42470439-42473339,exon_7:42477785-42477951,intron_3:42473949-42475242,intron_6:42476875-42477785,intron_5:42475864-42476765,;intron_8:42418594-42420645,exon_9:42420645-42423744,; LDM_A 6 270 -;-;-;-

oar_circ_0000651 chr13 42473339 42495889 + 22550 9896 ENSOARG00000010028; intron_3:42473949-42475242,intron_6:42476875-42477785,intron_5:42475864-42476765,intron_2:42473507-42473832,exon_2:42473339-42473507,intron_4:42475320-42475741,intron_7:42477951-42481575,exon_7:42477785-42477951,intron_8:42481658-42483746,; LDM_A 8 257 -

oar_circ_0000652 chr13 42473832 42498080 + 24248 11426 ENSOARG00000010221;ENSOARG00000010028; intron_1:42495889-42497912,;intron_4:42475320-42475741,intron_3:42473949-42475242,intron_7:42477951-42481575,intron_5:42475864-42476765,intron_6:42476875-42477785,exon_7:42477785-42477951,intron_8:42481658-42483746,; LDM_A 44 218 -;-

oar_circ_0000653 chr13 42476765 42500328 + 23563 10041 ENSOARG00000010221;ENSOARG00000010028; intron_1:42495889-42497912,intron_3:42498482-42499712,;intron_6:42476875-42477785,intron_7:42477951-42481575,intron_8:42481658-42483746,exon_7:42477785-42477951,; LDM_A 14 179 -;-

oar_circ_0000654 chr13 42476765 42561246 + 84481 31173 ENSOARG00000010355;ENSOARG00000010449;ENSOARG00000010221;ENSOARG00000010028; intron_10:42536684-42539566,intron_6:42532415-42533132,intron_9:42534101-42536601,;intron_1:42554105-42558702,;intron_7:42505612-42509849,intron_5:42500328-42504445,intron_3:42498482-42499712,intron_1:42495889-42497912,intron_8:42509932-42512014,;intron_7:42477951-42481575,intron_6:42476875-42477785,exon_7:42477785-42477951,intron_8:42481658-42483746,; LDM_A 12 191 -;-;-;-

oar_circ_0000655 chr13 44252453 44254463 + 2010 1454 ENSOARG00000013136; intron_10:44253531-44254093,intron_9:44252908-44253402,intron_8:44252580-44252819,intron_11:44254207-44254366,; LDM_E 4 24 PITRM1

oar_circ_0000656 chr13 45593223 45613188 + 19965 19265 ENSOARG00000015320; intron_2:45594040-45612683,intron_1:45593358-45593980,; LDM_E,LDM_A 6,11 2487,906 LARP4B

oar_circ_0000657 chr13 45634324 45635830 + 1506 1291 ENSOARG00000015320; intron_7:45634428-45635719,; LDM_E 2 216 LARP4B

oar_circ_0000658 chr13 46006828 46062810 - 55982 55149 ENSOARG00000016155; intron_4:46008422-46008783,exon_3:46033383-46033545,exon_14:46006828-46007014,intron_11:46017257-46017703,intron_5:46008991-46011647,intron_14:46043930-46062675,intron_9:46014405-46017047,intron_3:46007593-46008353,exon_13:46007320-46007593,intron_6:46011766-46012587,intron_13:46033545-46043770,exon_2:46043770-46043930,intron_8:46013838-46014317,intron_7:46012665-46013782,intron_2:46007014-46007320,intron_12:46017781-46033383,exon_11:46008783-46008991,; LDM_E,LDM_A 12,23 4705,2442 ZMYND11

oar_circ_0000659 chr13 46033383 46062810 - 29427 29292 ENSOARG00000016155; exon_3:46033383-46033545,exon_2:46043770-46043930,intron_13:46033545-46043770,intron_14:46043930-46062675,; LDM_E 18 2102 ZMYND11

oar_circ_0000660 chr13 46043770 46062810 - 19040 18905 ENSOARG00000016155; intron_14:46043930-46062675,exon_2:46043770-46043930,; LDM_E,LDM_A 2,6 955,386 ZMYND11

oar_circ_0000661 chr13 46982841 46999393 - 16552 16025 ENSOARG00000017627; intron_8:46990360-46994777,intron_7:46987665-46990282,intron_5:46982907-46985414,intron_6:46985476-46987625,intron_10:46998917-46999260,intron_9:46994870-46998862,; LDM_E 2 689 GPCPD1

oar_circ_0000662 chr13 46990282 46999393 - 9111 8752 ENSOARG00000017627; intron_8:46990360-46994777,intron_9:46994870-46998862,intron_10:46998917-46999260,; LDM_A 3 210 GPCPD1

oar_circ_0000663 chr13 46990282 47002093 - 11811 11452 ENSOARG00000017627; exon_7:47001861-47002093,intron_9:46994870-46998862,intron_10:46998917-46999260,exon_8:46999889-47000052,intron_11:46999393-46999889,intron_8:46990360-46994777,intron_12:47000052-47001861,; LDM_E 2 840 GPCPD1

oar_circ_0000664 chr13 46994777 47002093 - 7316 7035 ENSOARG00000017627; intron_11:46999393-46999889,intron_12:47000052-47001861,intron_10:46998917-46999260,intron_9:46994870-46998862,exon_7:47001861-47002093,exon_8:46999889-47000052,; LDM_E 4 649 GPCPD1

oar_circ_0000665 chr13 46998862 47008442 - 9580 9268 ENSOARG00000017627; intron_13:47002093-47008318,intron_12:47000052-47001861,intron_11:46999393-46999889,exon_8:46999889-47000052,intron_10:46998917-46999260,exon_7:47001861-47002093,; LDM_E 4 741 GPCPD1

oar_circ_0000666 chr13 46999889 47008442 - 8553 8429 ENSOARG00000017627; intron_13:47002093-47008318,intron_12:47000052-47001861,exon_7:47001861-47002093,exon_8:46999889-47000052,; LDM_E,LDM_A 116,44 681,251 GPCPD1

oar_circ_0000667 chr13 50509607 50511766 + 2159 2159 -- -- LDM_A 2 0 --

oar_circ_0000668 chr13 50612920 50632647 - 19727 15383 ENSOARG00000000750; exon_5:50625799-50625976,exon_3:50632294-50632647,intron_4:50625976-50627006,exon_4:50627006-50627260,intron_1:50617014-50620902,intron_5:50627260-50632294,intron_2:50621028-50623503,intron_3:50623627-50625799,; LDM_E,LDM_A 4,2 1622,569 PANK2

oar_circ_0000669 chr13 50612924 50632647 - 19723 15383 ENSOARG00000000750; intron_5:50627260-50632294,intron_1:50617014-50620902,intron_2:50621028-50623503,exon_5:50625799-50625976,exon_4:50627006-50627260,exon_3:50632294-50632647,intron_3:50623627-50625799,intron_4:50625976-50627006,; LDM_E 2 1622 PANK2

oar_circ_0000670 chr13 50616784 50632647 - 15863 15383 ENSOARG00000000750; intron_3:50623627-50625799,intron_2:50621028-50623503,intron_5:50627260-50632294,intron_1:50617014-50620902,exon_4:50627006-50627260,exon_3:50632294-50632647,intron_4:50625976-50627006,exon_5:50625799-50625976,; LDM_E,LDM_A 7,3 1622,569 PANK2

oar_circ_0000671 chr13 50616784 50636608 - 19824 19824 ENSOARG00000000750; intron_1:50617014-50620902,intron_5:50627260-50632294,intron_2:50621028-50623503,intron_6:50632647-50645112,intron_3:50623627-50625799,intron_4:50625976-50627006,exon_5:50625799-50625976,exon_3:50632294-50632647,exon_4:50627006-50627260,; LDM_E 3 1906 PANK2

oar_circ_0000672 chr13 50620902 50627260 - 6358 6108 ENSOARG00000000750; exon_5:50625799-50625976,exon_4:50627006-50627260,intron_4:50625976-50627006,intron_2:50621028-50623503,intron_3:50623627-50625799,; LDM_E,LDM_A 9,2 597,202 PANK2

oar_circ_0000673 chr13 50620902 50632647 - 11745 11495 ENSOARG00000000750; exon_5:50625799-50625976,exon_4:50627006-50627260,intron_5:50627260-50632294,exon_3:50632294-50632647,intron_2:50621028-50623503,intron_4:50625976-50627006,intron_3:50623627-50625799,; LDM_E,LDM_A 42,20 1346,502 PANK2

oar_circ_0000674 chr13 50620902 50636608 - 15706 15706 ENSOARG00000000750; exon_5:50625799-50625976,intron_4:50625976-50627006,exon_3:50632294-50632647,exon_4:50627006-50627260,intron_5:50627260-50632294,intron_2:50621028-50623503,intron_3:50623627-50625799,intron_6:50632647-50645112,; LDM_E,LDM_A 27,15 1630,568 PANK2

oar_circ_0000675 chr13 50684403 50684628 + 225 225 -- -- LDM_E 2 0 --

oar_circ_0000676 chr13 50929957 50934786 - 4829 4655 ENSOARG00000002627; exon_23:50929957-50930115,intron_6:50930115-50932405,intron_7:50932484-50934691,; LDM_E,LDM_A 3,2 222,64 ATRN

oar_circ_0000677 chr13 50929957 50945802 - 15845 15386 ENSOARG00000002627; intron_8:50934786-50940195,intron_9:50940248-50942367,intron_10:50942461-50945664,intron_7:50932484-50934691,intron_6:50930115-50932405,exon_23:50929957-50930115,; LDM_E,LDM_A 3,5 558,263 ATRN

oar_circ_0000678 chr13 50929957 50954983 - 25026 24409 ENSOARG00000002627; intron_9:50940248-50942367,intron_11:50945802-50954032,intron_12:50954266-50954797,intron_7:50932484-50934691,intron_8:50934786-50940195,exon_17:50954032-50954266,exon_16:50954797-50954983,intron_10:50942461-50945664,intron_6:50930115-50932405,; LDM_E 3 956 ATRN

oar_circ_0000679 chr13 50993536 51003423 - 9887 9560 ENSOARG00000002627; intron_27:51001095-51003339,intron_26:50993665-51000981,; LDM_E,LDM_A 5,4 219,141 ATRN

oar_circ_0000680 chr13 50993603 51003423 - 9820 9560 ENSOARG00000002627; intron_26:50993665-51000981,intron_27:51001095-51003339,; LDM_A 2 141 ATRN

oar_circ_0000681 chr13 51152013 51154493 + 2480 2151 ENSOARG00000003100; intron_3:51152138-51153265,intron_4:51153362-51154386,; LDM_E,LDM_A 6,6 95,21 C20orf194

oar_circ_0000682 chr13 51273708 51274218 + 510 328 ENSOARG00000003100; intron_31:51273815-51274143,; LDM_A 4 4 C20orf194

oar_circ_0000683 chr13 51506899 51508527 - 1628 1332 ENSOARG00000004955; intron_16:51507036-51507242,intron_17:51507316-51508442,; LDM_E,LDM_A 18,15 46,13 PTPRA

oar_circ_0000684 chr13 51584039 51584785 - 746 746 -- -- LDM_A 2 0 --

oar_circ_0000685 chr13 52340782 52341532 - 750 750 ENSOARG00000007289; exon_3:52340782-52341532,; LDM_E 2 120 STK35

oar_circ_0000686 chr13 54242975 54243402 + 427 163 ENSOARG00000013521; exon_67:54242975-54243138,; LDM_A 2 1 LAMA5

oar_circ_0000687 chr13 54369138 54369398 + 260 260 -- -- LDM_E 2 0 --

oar_circ_0000688 chr13 57012343 57015563 - 3220 2843 ENSOARG00000017053; intron_5:57014327-57015422,exon_4:57014161-57014327,intron_4:57013127-57014161,intron_3:57012487-57013035,; LDM_E,LDM_A 5,6 173,55 STX16

oar_circ_0000689 chr13 57235611 57254059 - 18448 18375 ENSOARG00000017143; exon_1:57253906-57254171,intron_4:57241269-57253906,exon_4:57235611-57235788,intron_3:57237651-57241165,intron_2:57235788-57237570,; LDM_E 5 1169 VAPB

oar_circ_0000690 chr13 59833975 59857609 + 23634 22529 ENSOARG00000019053; intron_4:59841254-59842389,intron_7:59848711-59849454,exon_1:59833972-59834186,intron_10:59854144-59855369,intron_6:59843992-59848600,intron_11:59855456-59857460,intron_5:59842449-59843908,intron_3:59840676-59841203,intron_2:59836564-59840574,intron_1:59834186-59836452,intron_9:59853257-59853995,intron_8:59849556-59853156,; LDM_E,LDM_A 3,3 2191,904 -

oar_circ_0000691 chr13 59836452 59849556 + 13104 12482 ENSOARG00000019053; intron_2:59836564-59840574,intron_3:59840676-59841203,intron_6:59843992-59848600,intron_5:59842449-59843908,intron_7:59848711-59849454,intron_4:59841254-59842389,; LDM_E,LDM_A 20,14 1479,565 -

oar_circ_0000692 chr13 60669594 60679267 + 9673 8544 ENSOARG00000001573; intron_10:60678490-60678835,intron_8:60674945-60678188,intron_6:60672531-60674597,intron_5:60671869-60672437,exon_11:60678835-60678988,exon_4:60669594-60669893,intron_4:60669893-60671763,; LDM_A 2 1632 MYLK2

oar_circ_0000693 chr13 60678835 60679267 + 432 153 ENSOARG00000001573; exon_11:60678835-60678988,; LDM_A 2 8 MYLK2

oar_circ_0000694 chr13 61118130 61123077 + 4947 4947 -- -- LDM_A 2 0 --

oar_circ_0000695 chr13 6170844 6180174 + 9330 9224 ENSOARG00000010864; intron_7:6170950-6179954,exon_8:6179954-6180174,; LDM_A 2 36 SPTLC3

oar_circ_0000696 chr13 62283518 62294614 - 11096 10661 ENSOARG00000007566; intron_2:62290209-62294464,exon_11:62294464-62294614,intron_1:62283812-62290068,; LDM_E,LDM_A 3,2 273,110 CDK5RAP1

oar_circ_0000697 chr13 62290068 62294614 - 4546 4405 ENSOARG00000007566; intron_2:62290209-62294464,exon_11:62294464-62294614,; LDM_E,LDM_A 9,17 183,59 CDK5RAP1

oar_circ_0000698 chr13 62846503 62852296 + 5793 5793 -- -- LDM_A 3 0 --

oar_circ_0000699 chr13 63400938 63411776 + 10838 10558 ENSOARG00000009759; intron_6:63409678-63410351,exon_6:63409520-63409678,intron_5:63403878-63409520,intron_7:63410541-63411680,intron_4:63401076-63403832,exon_7:63410351-63410541,; LDM_E,LDM_A 4,4 878,396 ITCH

oar_circ_0000700 chr13 63668844 63676994 - 8150 7888 ENSOARG00000010365; intron_13:63669000-63672495,intron_14:63672777-63676888,exon_3:63672495-63672777,; LDM_E,LDM_A 4,7 296,109 NCOA6

oar_circ_0000701 chr13 63672495 63676994 - 4499 4393 ENSOARG00000010365; intron_14:63672777-63676888,exon_3:63672495-63672777,; LDM_E,LDM_A 5,5 119,52 NCOA6

oar_circ_0000702 chr13 63691313 63698141 - 6828 6828 ENSOARG00000010365; intron_15:63676994-63692957,; LDM_A 2 33 NCOA6

oar_circ_0000703 chr13 64178512 64199758 - 21246 21006 ENSOARG00000012269; intron_5:64193714-64199685,intron_4:64178621-64193656,; LDM_A 3 405 UQCC1

oar_circ_0000704 chr13 64436141 64440699 - 4558 4558 ENSOARG00000014537; intron_15:64416760-64437815,intron_16:64437958-64440614,exon_1:64435086-64437936,; LDM_E 4 1591 RBM12

oar_circ_0000705 chr13 64437815 64440699 - 2884 2656 ENSOARG00000014537; intron_16:64437958-64440614,; LDM_E 2 490 RBM12

oar_circ_0000706 chr13 64456399 64461342 - 4943 4714 ENSOARG00000014653; exon_5:64461189-64461342,intron_8:64458171-64461189,intron_7:64456534-64458077,; LDM_A 2 8 -

oar_circ_0000707 chr13 64545746 64551804 + 6058 6058 ENSOARG00000015104; intron_2:64545861-64570761,; LDM_A 6 4317 PHF20

oar_circ_0000708 chr13 64545746 64572141 + 26395 26195 ENSOARG00000015104; intron_2:64545861-64570761,exon_3:64570761-64570933,intron_3:64570933-64572056,; LDM_E 4 982 PHF20

oar_circ_0000709 chr13 64580912 64590857 + 9945 9751 ENSOARG00000015104; intron_5:64580992-64584145,exon_6:64584145-64584530,intron_8:64590151-64590681,intron_7:64588880-64589958,intron_6:64584530-64588766,exon_8:64589958-64590151,exon_9:64590681-64590857,; LDM_E 2 593 PHF20

oar_circ_0000710 chr13 64611851 64632063 + 20212 20113 ENSOARG00000015104; exon_13:64631884-64632063,exon_12:64630345-64630510,exon_10:64611851-64612130,intron_11:64629336-64630345,intron_12:64630510-64631884,intron_10:64612130-64629237,; LDM_E 3 658 PHF20

oar_circ_0000711 chr13 66516141 66524623 + 8482 8242 ENSOARG00000018435; intron_4:66516254-66517930,exon_6:66524447-66524623,intron_5:66518057-66524447,; LDM_E,LDM_A 20,4 173,77 RPRD1B

oar_circ_0000712 chr13 69696676 69709210 - 12534 12072 ENSOARG00000002597; intron_34:69702998-69703182,intron_31:69696853-69698045,intron_35:69703269-69709092,exon_14:69698045-69698289,intron_32:69698289-69699540,exon_15:69696676-69696853,intron_33:69699662-69702863,; LDM_E 2 544 CHD6

oar_circ_0000713 chr13 69709092 69710835 - 1743 1503 ENSOARG00000002597; intron_37:69709923-69710772,intron_36:69709210-69709864,; LDM_E 2 54 CHD6

oar_circ_0000714 chr13 69727031 69729084 - 2053 1755 ENSOARG00000002597; intron_39:69727181-69728936,; LDM_E,LDM_A 6,4 34,6 CHD6

oar_circ_0000715 chr13 72864918 72866531 + 1613 1376 ENSOARG00000004487; intron_4:72865082-72866005,intron_5:72866101-72866390,exon_4:72864918-72865082,; LDM_E,LDM_A 13,7 67,41 YWHAB

oar_circ_0000716 chr13 72933378 72949626 + 16248 15569 ENSOARG00000004730; exon_6:72941555-72941723,intron_8:72948326-72949439,intron_7:72944729-72948194,intron_6:72941723-72944591,intron_3:72933507-72936616,exon_9:72949439-72949626,intron_4:72936731-72939206,intron_5:72939371-72941555,; LDM_A 4 109 STK4

oar_circ_0000717 chr13 75400847 75427917 - 27070 26070 ENSOARG00000009419; intron_18:75404391-75405185,exon_14:75400847-75401207,intron_16:75401207-75404178,intron_22:75411637-75412783,intron_19:75405339-75406719,intron_25:75418387-75427768,intron_21:75408124-75411549,intron_20:75406797-75408068,intron_24:75415480-75418170,exon_4:75418225-75418387,intron_23:75412876-75415366,; LDM_A 3 241 -

oar_circ_0000718 chr13 75411549 75418387 - 6838 6488 ENSOARG00000009419; intron_23:75412876-75415366,intron_24:75415480-75418170,exon_4:75418225-75418387,intron_22:75411637-75412783,; LDM_A 2 65 -

oar_circ_0000719 chr13 75701361 75703035 + 1674 1299 ENSOARG00000009818; exon_5:75701361-75701536,exon_6:75701987-75702176,intron_7:75702410-75702894,intron_5:75701536-75701987,; LDM_A 2 22 NCOA3

oar_circ_0000720 chr13 76476128 76477510 - 1382 976 ENSOARG00000010543; intron_35:76476276-76476665,intron_36:76476804-76477391,; LDM_A 2 5 PREX1

oar_circ_0000721 chr13 76705590 76711693 + 6103 5751 ENSOARG00000011349; intron_7:76705659-76707478,intron_8:76707630-76710144,exon_10:76711458-76711693,intron_9:76710275-76711458,; LDM_E 3 79 ARFGEF2

oar_circ_0000722 chr13 76716134 76719089 + 2955 2843 ENSOARG00000011349; exon_16:76718883-76719089,intron_14:76716303-76718161,intron_15:76718273-76718883,exon_14:76716134-76716303,; LDM_A 4 30 ARFGEF2

oar_circ_0000723 chr13 76795009 76804652 + 9643 8047 ENSOARG00000012031; intron_14:76797416-76798519,intron_13:76795071-76797279,intron_16:76800391-76801556,intron_20:76802731-76803245,intron_15:76798623-76800293,intron_21:76803327-76804505,exon_18:76801852-76802061,; LDM_E 3 361 CSE1L

oar_circ_0000724 chr13 76832966 76847923 - 14957 14719 ENSOARG00000012204; intron_9:76833065-76844453,intron_10:76844619-76847784,exon_5:76844453-76844619,; LDM_E,LDM_A 7,4 802,369 STAU1

oar_circ_0000725 chr13 76832966 76855116 - 22150 21912 ENSOARG00000012204; exon_1:76854827-76855116,intron_9:76833065-76844453,intron_10:76844619-76847784,exon_5:76844453-76844619,intron_11:76847923-76854827,; LDM_E,LDM_A 12,3 1388,648 STAU1

oar_circ_0000726 chr13 76832966 76865691 - 32725 21746 ENSOARG00000012204; intron_11:76847923-76854827,intron_9:76833065-76844453,intron_10:76844619-76847784,exon_1:76854827-76855116,; LDM_E 7 1388 STAU1

oar_circ_0000727 chr13 76923983 76924355 + 372 178 ENSOARG00000012345; exon_13:76924177-76924355,; LDM_A 2 3 DDX27

oar_circ_0000728 chr13 77504757 77510624 + 5867 5555 ENSOARG00000013014; intron_12:77507647-77510512,intron_11:77504874-77507564,; LDM_E,LDM_A 4,8 163,81 SLC9A8

oar_circ_0000729 chr13 77564854 77565313 + 459 459 -- -- LDM_A 2 0 --

oar_circ_0000730 chr13 78388436 78388817 - 381 381 ENSOARG00000013988; intron_3:78385999-78389094,; LDM_A 2 3 DPM1

oar_circ_0000731 chr13 78845346 78846642 - 1296 1296 ENSOARG00000014239; exon_8:78845346-78845519,intron_7:78845519-78846439,exon_7:78846439-78846642,; LDM_E 3 10 NFATC2

oar_circ_0000732 chr13 78887844 78893075 - 5231 4455 ENSOARG00000014239; exon_1:78892476-78893076,intron_9:78888016-78890863,intron_10:78890974-78891424,intron_12:78891616-78892174,; LDM_E 2 30 NFATC2

oar_circ_0000733 chr13 79048581 79057813 - 9232 8864 ENSOARG00000014351; intron_26:79055503-79057668,intron_25:79048690-79055389,; LDM_E,LDM_A 20,16 105,44 ATP9A

oar_circ_0000734 chr13 80148632 80151956 - 3324 3324 -- -- LDM_A 2 0 --

oar_circ_0000735 chr13 9750059 9752949 - 2890 2580 ENSOARG00000011950; intron_14:9751787-9752829,intron_13:9750197-9751735,; LDM_E 3 90 KIF16B

oar_circ_0000736 chr13 9843121 9854137 - 11016 10577 ENSOARG00000011950; intron_23:9844038-9846227,intron_24:9846344-9854023,intron_22:9843231-9843940,; LDM_A 2 99 KIF16B

oar_circ_0000737 chr14 1008334 1011119 + 2785 2587 ENSOARG00000004938; exon_4:1010944-1011119,intron_3:1009465-1010944,intron_2:1008417-1009350,; LDM_E 2 54 COG4

oar_circ_0000738 chr14 10886415 10889005 + 2590 2590 -- -- LDM_E,LDM_A 2,2 0,0 --

oar_circ_0000739 chr14 1123216 1149434 - 26218 26218 ENSOARG00000005163; intron_2:1123454-1150282,exon_9:1123216-1123454,; LDM_E,LDM_A 56,50 1474,698 -

oar_circ_0000740 chr14 1123226 1149970 + 26744 26744 -- -- LDM_E 3 0 --

oar_circ_0000741 chr14 1126806 1150460 - 23654 23654 ENSOARG00000005163; exon_8:1150282-1150460,intron_2:1123454-1150282,; LDM_E,LDM_A 26,29 1357,610 -

oar_circ_0000742 chr14 1132224 1155235 - 23011 23011 ENSOARG00000005163; intron_2:1123454-1150282,intron_4:1151946-1152516,intron_3:1150460-1151831,intron_5:1152619-1154733,exon_8:1150282-1150460,; LDM_E,LDM_A 44,16 1359,582 -

oar_circ_0000743 chr14 12423678 12424493 - 815 740 ENSOARG00000011734; intron_5:12423753-12424325,exon_5:12424325-12424493,; LDM_A 2 33 FBXO31

oar_circ_0000744 chr14 12423678 12429491 - 5813 5661 ENSOARG00000011734; exon_5:12424325-12424493,intron_6:12424493-12429414,intron_5:12423753-12424325,; LDM_A 3 62 FBXO31

oar_circ_0000745 chr14 12826017 12854753 - 28736 10045 ENSOARG00000012129;ENSOARG00000012206; exon_1:12826017-12826768,;intron_3:12847551-12851425,intron_2:12844896-12847395,intron_5:12851713-12854634,; LDM_E 10 67 SLC7A5;CA5A

oar_circ_0000746 chr14 12842740 12847551 - 4811 2499 ENSOARG00000012206; intron_2:12844896-12847395,; LDM_E 3 11 CA5A

oar_circ_0000747 chr14 12900999 12907292 + 6293 6062 ENSOARG00000012330; exon_3:12907092-12907292,intron_2:12904802-12907092,intron_1:12901138-12904710,; LDM_E,LDM_A 7,6 143,63 BANP

oar_circ_0000748 chr14 12900999 12920978 + 19979 19631 ENSOARG00000012330; exon_3:12907092-12907292,intron_2:12904802-12907092,intron_4:12919479-12920802,exon_5:12920802-12920978,intron_3:12907292-12919362,intron_1:12901138-12904710,; LDM_E 7 344 BANP

oar_circ_0000749 chr14 13281736 13296110 + 14374 14374 ENSOARG00000012499; intron_9:13291298-13292072,intron_10:13292075-13302529,intron_6:13281845-13283290,intron_7:13283486-13291007,; LDM_E 10 25 ZFPM1

oar_circ_0000750 chr14 13472546 13475304 - 2758 2758 ENSOARG00000013343; exon_25:13472546-13472790,intron_29:13473041-13473708,exon_24:13472887-13473041,intron_30:13473813-13479339,; LDM_E 14 39 PIEZO1

oar_circ_0000751 chr14 13619458 13619705 - 247 247 -- -- LDM_A 2 0 --

oar_circ_0000752 chr14 1418105 1418292 + 187 187 -- -- LDM_E 2 0 --

oar_circ_0000753 chr14 14500189 14508393 - 8204 7118 ENSOARG00000015518; exon_10:14506251-14506459,intron_5:14503697-14505685,intron_6:14505841-14506251,exon_14:14500801-14501041,exon_13:14501744-14501924,exon_11:14505685-14505841,intron_3:14501041-14501744,intron_7:14506459-14508042,intron_4:14501924-14503574,; LDM_A 6 204 VPS35

oar_circ_0000754 chr14 14511265 14518028 - 6763 6249 ENSOARG00000015518; exon_5:14512875-14513089,intron_10:14511375-14511823,intron_12:14513089-14514910,intron_15:14516627-14517929,intron_11:14511907-14512875,intron_13:14515093-14515718,intron_14:14515842-14516530,exon_4:14514910-14515093,; LDM_E 3 245 VPS35

oar_circ_0000755 chr14 14515718 14518028 - 2310 1990 ENSOARG00000015518; intron_15:14516627-14517929,intron_14:14515842-14516530,; LDM_E,LDM_A 4,4 29,16 VPS35

oar_circ_0000756 chr14 14530700 14533596 + 2896 1209 ENSOARG00000015649; intron_4:14531171-14532380,; LDM_E,LDM_A 3,2 13,4 ORC6

oar_circ_0000757 chr14 15364798 15387348 - 22550 22176 ENSOARG00000016143; intron_13:15364893-15384461,intron_14:15384536-15385742,intron_15:15385800-15387202,; LDM_A 5 117 ITFG1

oar_circ_0000758 chr14 15425775 15468461 + 42686 42055 ENSOARG00000016405; intron_4:15435678-15444665,intron_6:15448081-15468345,intron_5:15444773-15448000,intron_2:15425862-15430496,intron_3:15430635-15435578,; LDM_E,LDM_A 7,7 865,843 PHKB

oar_circ_0000759 chr14 15503500 15509835 + 6335 6103 ENSOARG00000016405; exon_10:15504814-15505012,intron_10:15505012-15506824,exon_13:15509676-15509835,intron_9:15503596-15504814,intron_11:15506882-15507682,intron_12:15507760-15509676,; LDM_A 2 323 PHKB

oar_circ_0000760 chr14 15507682 15509835 + 2153 2075 ENSOARG00000016405; intron_12:15507760-15509676,exon_13:15509676-15509835,; LDM_E,LDM_A 4,2 81,85 PHKB

oar_circ_0000761 chr14 15570607 15570817 - 210 210 -- -- LDM_E 2 0 --

oar_circ_0000762 chr14 15579034 15592747 + 13713 13161 ENSOARG00000016405; intron_22:15584215-15585338,exon_26:15592544-15592747,intron_25:15589717-15592544,intron_23:15585420-15587415,intron_24:15587473-15589626,intron_20:15579410-15583905,exon_22:15584052-15584215,intron_19:15579117-15579319,; LDM_A 2 728 PHKB

oar_circ_0000763 chr14 16205432 16207408 + 1976 1844 ENSOARG00000016864; exon_2:16205432-16205667,intron_2:16205667-16207276,; LDM_E 2 60 LONP2

oar_circ_0000764 chr14 16205432 16229392 + 23960 23468 ENSOARG00000016864; intron_4:16208740-16210234,exon_7:16220417-16220676,intron_3:16207408-16208617,intron_5:16210398-16213394,intron_7:16220676-16229250,exon_2:16205432-16205667,intron_6:16213489-16220417,exon_5:16210234-16210398,intron_2:16205667-16207276,; LDM_E 5 875 LONP2

oar_circ_0000765 chr14 16208617 16213489 + 4872 4654 ENSOARG00000016864; intron_4:16208740-16210234,exon_5:16210234-16210398,intron_5:16210398-16213394,; LDM_E 2 98 LONP2

oar_circ_0000766 chr14 16229250 16245836 + 16586 16032 ENSOARG00000016864; intron_10:16242345-16245702,intron_9:16240214-16242218,intron_8:16229392-16240063,; LDM_E 2 537 LONP2

oar_circ_0000767 chr14 16240063 16245836 + 5773 5512 ENSOARG00000016864; exon_9:16240063-16240214,intron_9:16240214-16242218,intron_10:16242345-16245702,; LDM_A 2 45 LONP2

oar_circ_0000768 chr14 1697963 1709103 - 11140 10710 ENSOARG00000006324; intron_20:1698052-1702477,intron_21:1702515-1704718,intron_23:1705986-1708967,intron_22:1704799-1705900,; LDM_E 2 218 WDR59

oar_circ_0000769 chr14 1762509 1763557 + 1048 1048 ENSOARG00000006385; intron_1:1750653-1827303,; LDM_A 7 30 ZNRF1

oar_circ_0000770 chr14 17973493 17977199 + 3706 3259 ENSOARG00000017079; intron_5:17973772-17977031,; LDM_E 2 79 CNEP1R1

oar_circ_0000771 chr14 18232901 18235478 - 2577 2377 ENSOARG00000017248; exon_7:18235293-18235478,intron_10:18234017-18235293,intron_9:18232977-18233893,; LDM_E,LDM_A 2,3 81,51 BRD7

oar_circ_0000772 chr14 2086930 2101466 - 14536 14173 ENSOARG00000006663; exon_3:2099187-2099407,intron_4:2098878-2099187,intron_5:2099407-2101348,intron_3:2087050-2098753,; LDM_E 5 147 CFDP1

oar_circ_0000773 chr14 2098753 2101466 - 2713 2161 ENSOARG00000006663; intron_5:2099407-2101348,exon_3:2099187-2099407,; LDM_A 3 31 CFDP1

oar_circ_0000774 chr14 21124600 21126220 + 1620 1620 ENSOARG00000017664; exon_1:21124600-21126220,; LDM_E 4 1267 CHD9

oar_circ_0000775 chr14 2119135 2147163 - 28028 4591 ENSOARG00000006717; intron_1:2143397-2147988,; LDM_E 5 691 -

oar_circ_0000776 chr14 21211941 21221891 + 9950 9749 ENSOARG00000017664; exon_22:21221214-21221374,intron_20:21217226-21217678,intron_22:21221374-21221801,intron_19:21212052-21217029,intron_21:21217878-21221214,exon_20:21217029-21217226,exon_21:21217678-21217878,; LDM_E 6 865 CHD9

oar_circ_0000777 chr14 21237175 21237424 - 249 249 -- -- LDM_A 2 0 --

oar_circ_0000778 chr14 21692645 21702656 + 10011 9787 ENSOARG00000017939; intron_5:21692725-21702512,; LDM_A 3 44 FTO

oar_circ_0000779 chr14 21692645 21710921 + 18276 17932 ENSOARG00000017939; intron_5:21692725-21702512,intron_6:21702656-21710801,; LDM_E,LDM_A 21,8 359,100 FTO

oar_circ_0000780 chr14 21702512 21710921 + 8409 8145 ENSOARG00000017939; intron_6:21702656-21710801,; LDM_E 2 203 FTO

oar_circ_0000781 chr14 21976016 21977045 + 1029 1029 -- -- LDM_A 2 0 --

oar_circ_0000782 chr14 22720458 22721710 - 1252 1252 -- -- LDM_E,LDM_A 2,2 0,0 --

oar_circ_0000783 chr14 23443509 23477769 + 34260 15580 ENSOARG00000018232; intron_11:23459069-23459812,intron_5:23444893-23446607,intron_10:23458217-23458921,intron_4:23443643-23444739,intron_7:23449265-23451837,intron_8:23451876-23456475,intron_6:23446712-23449160,exon_14:23461218-23461402,intron_9:23456616-23458136,; LDM_A 13 33 -

oar_circ_0000784 chr14 23461218 23498468 + 37250 9606 ENSOARG00000018268;ENSOARG00000018232; intron_12:23492840-23497356,intron_5:23481003-23482095,intron_9:23488303-23490025,intron_10:23490166-23492258,;exon_14:23461218-23461402,; LDM_A 4 22 -;-

oar_circ_0000785 chr14 23507445 23517317 - 9872 9872 ENSOARG00000018341; intron_2:23506732-23518045,; LDM_A 4 9 -

oar_circ_0000786 chr14 23660323 23666917 - 6594 6294 ENSOARG00000018419; intron_10:23660424-23662848,intron_11:23662901-23666771,; LDM_E,LDM_A 4,2 163,75 OGFOD1

oar_circ_0000787 chr14 23725371 23727408 + 2037 1933 ENSOARG00000018465; intron_8:23725562-23727304,exon_8:23725371-23725562,; LDM_E,LDM_A 37,31 107,83 AMFR

oar_circ_0000788 chr14 24614059 24627764 - 13705 12657 ENSOARG00000018905; intron_3:24621833-24626506,intron_2:24614124-24621704,intron_4:24626630-24627034,; LDM_A 4 89 FAM192A

oar_circ_0000789 chr14 24887613 24888173 - 560 560 -- -- LDM_A 2 0 --

oar_circ_0000790 chr14 25764570 25767124 - 2554 2426 ENSOARG00000001567; exon_12:25764570-25764811,intron_37:25764811-25766996,; LDM_E 2 176 CNOT1

oar_circ_0000791 chr14 25868922 25869487 - 565 565 ENSOARG00000001717; exon_10:25868549-25869505,; LDM_A 2 822 GOT2

oar_circ_0000792 chr14 33816167 33821221 - 5054 4652 ENSOARG00000002145; exon_5:33821051-33821221,intron_6:33816227-33816677,intron_8:33819072-33820023,intron_7:33816789-33818936,intron_9:33820117-33821051,; LDM_E,LDM_A 3,2 451,158 DYNC1LI2

oar_circ_0000793 chr14 34434120 34434895 + 775 775 -- -- LDM_E,LDM_A 12,12 0,0 --

oar_circ_0000794 chr14 34510326 34510492 + 166 166 ENSOARG00000003053; exon_2:34509702-34510492,; LDM_A 2 10 CTCF

oar_circ_0000795 chr14 34573460 34573806 - 346 346 ENSOARG00000003135; exon_1:34573460-34573806,; LDM_E,LDM_A 3,7 48,25 GFOD2

oar_circ_0000796 chr14 34887369 34890433 + 3064 3064 ENSOARG00000003342; intron_2:34888504-34890270,exon_3:34890270-34890433,exon_2:34887369-34888504,; LDM_E,LDM_A 6,5 496,449 NFATC3

oar_circ_0000797 chr14 34887369 34907538 + 20169 20169 ENSOARG00000003342; intron_2:34888504-34890270,intron_3:34890433-34907338,exon_3:34890270-34890433,exon_2:34887369-34888504,exon_4:34907338-34907538,; LDM_E,LDM_A 8,11 899,754 NFATC3

oar_circ_0000798 chr14 35756639 35756853 + 214 214 ENSOARG00000003518; exon_8:35756637-35756853,; LDM_A 5 20 SNTB2

oar_circ_0000799 chr14 35756639 35774611 + 17972 17831 ENSOARG00000003518; intron_8:35756853-35767868,intron_9:35768079-35774468,exon_9:35767868-35768079,exon_8:35756637-35756853,; LDM_E 2 1081 SNTB2

oar_circ_0000800 chr14 39522401 39524131 + 1730 1677 ENSOARG00000004041; intron_2:39522454-39523904,exon_3:39523904-39524131,; LDM_A 2 24 POP4

oar_circ_0000801 chr14 41902227 41904116 - 1889 1889 -- -- LDM_E 4 0 --

oar_circ_0000802 chr14 42152534 42159926 + 7392 7159 ENSOARG00000004147; intron_1:42154672-42156104,exon_1:42152534-42154672,intron_2:42156222-42159811,; LDM_E 2 628 ZNF507

oar_circ_0000803 chr14 42498301 42509383 + 11082 10524 ENSOARG00000004254; intron_3:42500450-42502130,intron_4:42502250-42504419,intron_5:42504475-42505872,intron_6:42505958-42509193,intron_2:42498460-42500313,exon_7:42509193-42509383,; LDM_E 2 96 TDRD12

oar_circ_0000804 chr14 42547946 42562381 + 14435 10946 ENSOARG00000004254; intron_26:42557307-42558667,intron_25:42551683-42557197,intron_24:42549427-42551510,intron_23:42548157-42549334,exon_25:42551510-42551683,intron_30:42559327-42559755,exon_23:42547946-42548157,; LDM_E 2 197 TDRD12

oar_circ_0000805 chr14 42625757 42628097 - 2340 2108 ENSOARG00000004306; exon_16:42627929-42628097,intron_3:42625847-42627246,intron_4:42627388-42627929,; LDM_E 2 24 CEP89

oar_circ_0000806 chr14 42707707 42714664 - 6957 4322 ENSOARG00000004339; intron_6:42709425-42711140,intron_8:42711890-42714497,; LDM_A 2 11 RHPN2

oar_circ_0000807 chr14 42815558 42817029 + 1471 1206 ENSOARG00000004365; intron_14:42816504-42816862,intron_13:42815686-42816367,exon_15:42816862-42817029,; LDM_E 2 16 GPATCH1

oar_circ_0000808 chr14 43925394 43927920 + 2526 2396 ENSOARG00000004475; intron_2:43925558-43927790,exon_2:43925394-43925558,; LDM_A 2 101 LSM14A

oar_circ_0000809 chr14 43945132 43946939 + 1807 1695 ENSOARG00000004475; exon_9:43946707-43946939,intron_8:43945599-43946707,exon_7:43945132-43945315,exon_8:43945427-43945599,; LDM_E,LDM_A 11,2 196,109 LSM14A

oar_circ_0000810 chr14 44136180 44150626 + 14446 13633 ENSOARG00000004533; intron_4:44136281-44137769,intron_6:44141405-44141884,intron_11:44148034-44150470,exon_9:44145652-44145819,intron_8:44144738-44145652,intron_5:44137891-44141337,exon_12:44150470-44150626,intron_9:44145819-44147734,intron_7:44142006-44144638,; LDM_E 2 679 UBA2

oar_circ_0000811 chr14 44136180 44152279 + 16099 15189 ENSOARG00000004533; intron_5:44137891-44141337,intron_8:44144738-44145652,intron_7:44142006-44144638,intron_9:44145819-44147734,exon_12:44150470-44150626,intron_12:44150626-44152182,intron_6:44141405-44141884,intron_4:44136281-44137769,exon_9:44145652-44145819,intron_11:44148034-44150470,; LDM_E 2 717 UBA2

oar_circ_0000812 chr14 44643189 44644777 - 1588 1526 ENSOARG00000004631; exon_4:44643251-44644777,; LDM_E,LDM_A 2,4 110,102 ZNF599

oar_circ_0000813 chr14 45867559 45872199 + 4640 4133 ENSOARG00000005231; intron_16:45871391-45872123,intron_14:45867685-45871086,; LDM_E 2 29 WDR62

oar_circ_0000814 chr14 46079291 46080020 - 729 729 ENSOARG00000005318; exon_4:46078596-46080020,; LDM_A 2 1 ZNF382

oar_circ_0000815 chr14 46326190 46326676 - 486 486 -- -- LDM_E 4 0 --

oar_circ_0000816 chr14 46326190 46339325 - 13135 12853 ENSOARG00000005350; intron_3:46326676-46336660,exon_1:46339052-46339325,intron_4:46336719-46339052,intron_2:46326286-46326549,; LDM_E,LDM_A 4,2 431,156 ZNF546

oar_circ_0000817 chr14 46326190 46342688 - 16498 12853 ENSOARG00000005350; intron_3:46326676-46336660,intron_4:46336719-46339052,exon_1:46339052-46339325,intron_2:46326286-46326549,; LDM_E,LDM_A 8,2 431,156 ZNF546

oar_circ_0000818 chr14 46476529 46477094 + 565 565 -- -- LDM_E 2 0 --

oar_circ_0000819 chr14 4653277 4657452 + 4175 4052 ENSOARG00000007916; intron_3:4654816-4657273,intron_2:4653342-4654758,exon_4:4657273-4657452,; LDM_A 3 7 WWOX

oar_circ_0000820 chr14 46573251 46573781 - 530 307 ENSOARG00000005402; intron_2:46573347-46573654,; LDM_E 5 5 ZNF268

oar_circ_0000821 chr14 46775964 46781418 - 5454 3580 ENSOARG00000005441; intron_3:46777945-46779770,intron_2:46776063-46777818,; LDM_E 2 29 -

oar_circ_0000822 chr14 47461749 47462980 + 1231 1098 ENSOARG00000005704; intron_35:47461882-47462819,exon_36:47462819-47462980,; LDM_E,LDM_A 2,5 22,51 RYR1

oar_circ_0000823 chr14 47509894 47535720 + 25826 25162 ENSOARG00000005704; intron_91:47509975-47513961,intron_95:47517228-47519546,intron_96:47519734-47535378,exon_96:47519546-47519734,intron_94:47514462-47517146,exon_97:47535378-47535720,; LDM_A 4 2611 RYR1

oar_circ_0000824 chr14 47513961 47519734 + 5773 5190 ENSOARG00000005704; intron_94:47514462-47517146,exon_96:47519546-47519734,intron_95:47517228-47519546,; LDM_A 5 755 RYR1

oar_circ_0000825 chr14 48840020 48841815 - 1795 1504 ENSOARG00000006598; intron_3:48840173-48841677,; LDM_E 3 16 C19orf47

oar_circ_0000826 chr14 48840020 48845040 - 5020 4647 ENSOARG00000006598; intron_3:48840173-48841677,intron_4:48841815-48844958,; LDM_E 2 54 C19orf47

oar_circ_0000827 chr14 49146028 49148874 + 2846 2746 ENSOARG00000006947; exon_3:49148660-49148874,intron_2:49146128-49148660,; LDM_E 4 26 ITPKC

oar_circ_0000828 chr14 49150303 49150508 + 205 205 ENSOARG00000006947; exon_4:49150303-49150508,; LDM_A 2 7 ITPKC

oar_circ_0000829 chr14 49974399 49975573 - 1174 171 ENSOARG00000007781; exon_44:49975402-49975573,; LDM_E,LDM_A 2,3 8,3 MEGF8

oar_circ_0000830 chr14 50380158 50380887 - 729 729 -- -- LDM_E 4 0 --

oar_circ_0000831 chr14 50888527 50888683 - 156 156 -- -- LDM_E 2 0 --

oar_circ_0000832 chr14 51211508 51224148 + 12640 12640 ENSOARG00000009036; intron_4:51222585-51224021,intron_3:51220803-51222523,exon_3:51220633-51220803,intron_2:51207467-51220633,; LDM_E 3 519 ZNF112

oar_circ_0000833 chr14 51220633 51224148 + 3515 3156 ENSOARG00000009036; intron_4:51222585-51224021,intron_3:51220803-51222523,; LDM_A 5 6 ZNF112

oar_circ_0000834 chr14 51867079 51868448 + 1369 279 ENSOARG00000009634; intron_3:51867125-51867404,; LDM_E 5 3 PPP1R37

oar_circ_0000835 chr14 52023495 52023730 + 235 235 -- -- LDM_A 2 0 --

oar_circ_0000836 chr14 52023606 52023814 - 208 208 -- -- LDM_A 2 0 --

oar_circ_0000837 chr14 52023829 52024529 + 700 700 -- -- LDM_A 2 0 --

oar_circ_0000838 chr14 52029055 52030355 + 1300 1300 -- -- LDM_A 2 0 --

oar_circ_0000839 chr14 52654814 52656213 + 1399 1005 ENSOARG00000010539; intron_9:52654933-52655938,; LDM_E 5 12 HIF3A

oar_circ_0000840 chr14 52762701 52763100 - 399 399 -- -- LDM_E 4 0 --

oar_circ_0000841 chr14 53056022 53059899 + 3877 3877 ENSOARG00000010829; exon_1:53056022-53059899,; LDM_A 3 1727 ARHGAP35

oar_circ_0000842 chr14 53246130 53254427 + 8297 7942 ENSOARG00000010941; intron_2:53246242-53250158,intron_3:53250332-53252571,exon_3:53250158-53250332,intron_4:53252714-53254327,; LDM_E,LDM_A 4,19 192,120 SAE1

oar_circ_0000843 chr14 53336447 53337106 + 659 305 ENSOARG00000010972; exon_12:53336447-53336752,; LDM_E,LDM_A 3,4 23,33 CCDC9

oar_circ_0000844 chr14 53690308 53690721 + 413 413 ENSOARG00000011391; exon_3:53690308-53690721,; LDM_E 2 438 EHD2

oar_circ_0000845 chr14 54056221 54057580 + 1359 1235 ENSOARG00000011673; exon_5:54056221-54057456,; LDM_E 7 68 ZNF114

oar_circ_0000846 chr14 54531500 54532042 + 542 186 ENSOARG00000012427; exon_7:54531500-54531686,; LDM_E,LDM_A 27,9 27,13 NUCB1

oar_circ_0000847 chr14 54589698 54590252 - 554 242 ENSOARG00000012463; intron_12:54589843-54590085,; LDM_E,LDM_A 22,60 2,7 GYS1

oar_circ_0000848 chr14 54983088 54983712 - 624 624 -- -- LDM_A 3 0 --

oar_circ_0000849 chr14 55220850 55238321 + 17471 3500 ENSOARG00000013588;ENSOARG00000013644; intron_19:55221735-55222180,;intron_2:55234399-55234935,intron_3:55234949-55236336,intron_7:55237000-55238132,; LDM_E,LDM_A 7,5 372,170 MED25;PTOV1

oar_circ_0000850 chr14 55636098 55647336 + 11238 9906 ENSOARG00000013964; exon_20:55642198-55642366,exon_26:55647156-55647351,intron_25:55645189-55647156,intron_23:55643313-55644507,intron_19:55640705-55642198,intron_24:55644667-55645052,exon_18:55639931-55640120,intron_15:55638062-55639139,exon_22:55642736-55642932,intron_17:55639501-55639931,intron_18:55640120-55640570,exon_24:55644507-55644667,intron_14:55637449-55637902,intron_22:55642932-55643173,intron_13:55636187-55637335,exon_16:55639139-55639299,; LDM_A 2 4054 MYBPC2

oar_circ_0000851 chr14 56362846 56363675 - 829 829 ENSOARG00000014733; exon_8:56362846-56363675,; LDM_A 2 3 -

oar_circ_0000852 chr14 56370505 56373586 - 3081 2856 ENSOARG00000014733; exon_2:56373427-56373586,intron_5:56372367-56373106,intron_4:56370664-56372304,exon_5:56370505-56370664,exon_3:56373106-56373265,; LDM_E,LDM_A 3,3 44,75 -

oar_circ_0000853 chr14 56648626 56652061 - 3435 3435 ENSOARG00000014940; intron_3:56649594-56656530,intron_2:56648722-56649467,; LDM_E,LDM_A 2,4 88,26 -

oar_circ_0000854 chr14 56686592 56688899 + 2307 2087 ENSOARG00000014990; intron_3:56688522-56688806,intron_2:56686800-56688395,exon_2:56686592-56686800,; LDM_A 2 11 ZNF613

oar_circ_0000855 chr14 56710965 56711301 + 336 336 ENSOARG00000014998; exon_4:56709894-56711615,; LDM_E 2 12 ZNF432

oar_circ_0000856 chr14 56774361 56785051 + 10690 10690 -- -- LDM_E 2 0 --

oar_circ_0000857 chr14 57395388 57419695 + 24307 24307 -- -- LDM_E 3 0 --

oar_circ_0000858 chr14 57673222 57691805 + 18583 9858 ENSOARG00000000192;ENSOARG00000000154; intron_2:57686062-57691678,;intron_6:57673710-57676283,intron_4:57671947-57673383,exon_6:57673477-57673710,; LDM_E 2 392 ZNF836;-

oar_circ_0000859 chr14 57674626 57699420 - 24794 24794 -- -- LDM_E 4 0 --

oar_circ_0000860 chr14 58033630 58062642 + 29012 29012 -- -- LDM_E 5 0 --

oar_circ_0000861 chr14 58922613 58924560 + 1947 1734 ENSOARG00000000955; intron_2:58922688-58924422,; LDM_A 5 5 CACNG6

oar_circ_0000862 chr14 60308908 60309107 - 199 199 ENSOARG00000002981; exon_14:60308623-60309107,; LDM_A 2 4 CNOT3

oar_circ_0000863 chr14 60658580 60659008 - 428 205 ENSOARG00000003315; intron_2:60658676-60658881,; LDM_A 2 1 ZNF583

oar_circ_0000864 chr14 60800291 60833946 - 33655 33655 ENSOARG00000003373; intron_5:60788214-60838713,; LDM_E 4 8286 -

oar_circ_0000865 chr14 60802404 60833103 - 30699 30699 ENSOARG00000003373; intron_5:60788214-60838713,; LDM_E 4 5388 -

oar_circ_0000866 chr14 60832330 60832527 - 197 197 ENSOARG00000003373; intron_5:60788214-60838713,; LDM_E 3 347 -

oar_circ_0000867 chr14 60832726 60832905 - 179 179 ENSOARG00000003373; intron_5:60788214-60838713,; LDM_E 2 151 -

oar_circ_0000868 chr14 60832839 60833037 - 198 198 ENSOARG00000003373; intron_5:60788214-60838713,; LDM_E 2 186 -

oar_circ_0000869 chr14 60832839 60833103 - 264 264 ENSOARG00000003373; intron_5:60788214-60838713,; LDM_E 3 425 -

oar_circ_0000870 chr14 60980344 61000412 - 20068 7334 ENSOARG00000003405;ENSOARG00000003399; exon_6:60993369-60993552,intron_3:60998035-61000306,intron_1:60993552-60995756,intron_2:60995848-60997945,;intron_5:60980439-60981018,; LDM_E 14 72 -;-

oar_circ_0000871 chr14 60987694 61003240 - 15546 8681 ENSOARG00000003405; exon_6:60993369-60993552,intron_4:61000412-61002338,intron_3:60998035-61000306,intron_1:60993552-60995756,intron_2:60995848-60997945,; LDM_E 5 83 -

oar_circ_0000872 chr14 60995249 61001808 + 6559 6559 -- -- LDM_E 2 0 --

oar_circ_0000873 chr14 61151448 61153809 + 2361 2361 -- -- LDM_E 2 0 --

oar_circ_0000874 chr14 61543680 61558470 - 14790 2888 ENSOARG00000003623;ENSOARG00000003618; exon_3:61557298-61558470,;intron_2:61543807-61545523,; LDM_E,LDM_A 3,3 263,108 -;-

oar_circ_0000875 chr14 61899971 61900139 + 168 168 ENSOARG00000003710; intron_1:61856706-61901074,; LDM_E 2 5 -

oar_circ_0000876 chr14 62018830 62019082 - 252 252 -- -- LDM_E 2 0 --

oar_circ_0000877 chr14 7083985 7090974 - 6989 6667 ENSOARG00000008122; intron_1:7084122-7086651,intron_2:7086723-7090861,; LDM_E 3 318 CMC2

oar_circ_0000878 chr14 800013 804610 + 4597 3464 ENSOARG00000004573; intron_2:800638-801426,intron_3:801489-801942,intron_5:802600-802853,intron_4:802050-802490,intron_7:804111-804457,exon_2:800470-800638,intron_6:802960-803976,; LDM_E 2 24 VAC14

oar_circ_0000879 chr14 8860770 8867027 + 6257 6257 ENSOARG00000009566; intron_1:8805252-8966055,; LDM_A 2 142 CDH13

oar_circ_0000880 chr14 9074365 9074955 - 590 590 -- -- LDM_E 2 0 --

oar_circ_0000881 chr14 9865244 9879317 - 14073 14073 ENSOARG00000010156; intron_10:9871194-9871407,intron_6:9868053-9868741,intron_8:9869715-9870285,intron_7:9868866-9869558,exon_12:9871407-9871596,intron_5:9865385-9867975,intron_11:9871596-9874765,intron_12:9874910-9886577,exon_15:9869558-9869715,exon_13:9871021-9871194,; LDM_E,LDM_A 28,11 376,129 MBTPS1

oar_circ_0000882 chr15 1019492 1026449 - 6957 6749 ENSOARG00000002115; intron_11:1023647-1026277,exon_8:1026277-1026449,intron_10:1019619-1023566,; LDM_E 2 114 MRE11A

oar_circ_0000883 chr15 1112449 1122459 - 10010 9830 ENSOARG00000002602; intron_3:1121656-1122337,intron_2:1114985-1121494,exon_4:1121494-1121656,exon_6:1112435-1112600,intron_1:1112600-1114913,; LDM_E 3 446 AASDHPPT

oar_circ_0000884 chr15 1126401 1126630 + 229 229 -- -- LDM_E 2 0 --

oar_circ_0000885 chr15 13639617 13641140 + 1523 1523 -- -- LDM_E 2 0 --

oar_circ_0000886 chr15 13890793 13907031 + 16238 15520 ENSOARG00000008648; intron_6:13895178-13896247,exon_9:13906842-13907031,intron_7:13896331-13905951,intron_8:13906101-13906842,intron_5:13891175-13895076,; LDM_E,LDM_A 4,3 390,173 MTMR2

oar_circ_0000887 chr15 13905951 13907031 + 1080 930 ENSOARG00000008648; exon_9:13906842-13907031,intron_8:13906101-13906842,; LDM_E,LDM_A 2,2 18,10 MTMR2

oar_circ_0000888 chr15 14737901 14738909 + 1008 1008 ENSOARG00000009085; exon_4:14737901-14738138,exon_5:14738734-14738909,intron_4:14738138-14738734,; LDM_E,LDM_A 19,6 301,41 SESN3

oar_circ_0000889 chr15 16569356 16589289 + 19933 18474 ENSOARG00000010653; intron_2:16569458-16587932,; LDM_A 2 15 ELMOD1

oar_circ_0000890 chr15 16569356 16593787 + 24431 18474 ENSOARG00000010653; intron_2:16569458-16587932,; LDM_A 3 15 ELMOD1

oar_circ_0000891 chr15 17153406 17156819 + 3413 2962 ENSOARG00000010875; exon_4:17153406-17153583,intron_5:17154983-17156510,intron_4:17153583-17154841,; LDM_E,LDM_A 3,2 98,80 CUL5

oar_circ_0000892 chr15 17173364 17177979 + 4615 4008 ENSOARG00000010875; intron_8:17173458-17174709,intron_10:17175157-17177914,; LDM_E 2 123 CUL5

oar_circ_0000893 chr15 17245582 17246378 + 796 475 ENSOARG00000011112; intron_4:17246074-17246277,intron_3:17245700-17245972,; LDM_E 2 14 ACAT1

oar_circ_0000894 chr15 17249707 17251492 + 1785 1545 ENSOARG00000011112; intron_6:17249851-17250288,intron_7:17250439-17251396,exon_7:17250288-17250439,; LDM_A 6 102 ACAT1

oar_circ_0000895 chr15 17303335 17307367 - 4032 3353 ENSOARG00000011428; intron_15:17305712-17307306,intron_14:17304099-17305639,exon_6:17303335-17303554,; LDM_E,LDM_A 20,11 202,52 NPAT

oar_circ_0000896 chr15 17303335 17319921 - 16586 16292 ENSOARG00000011428; intron_15:17305712-17307306,intron_14:17304099-17305639,exon_6:17303335-17303554,intron_13:17303554-17304058,intron_16:17307367-17319802,; LDM_E 3 713 NPAT

oar_circ_0000897 chr15 17382382 17395402 + 13020 12388 ENSOARG00000011894; exon_24:17392542-17392716,exon_26:17395155-17395402,exon_25:17393572-17393742,exon_20:17386208-17386364,intron_23:17390896-17392542,exon_17:17382382-17382554,intron_17:17382554-17383373,intron_18:17383573-17386028,intron_25:17393742-17395155,intron_22:17387512-17390781,exon_18:17383373-17383573,intron_20:17386364-17387175,intron_24:17392716-17393572,; LDM_E 2 633 ATM

oar_circ_0000898 chr15 17865627 17883974 + 18347 17880 ENSOARG00000012714; intron_4:17866949-17868012,intron_8:17877703-17883889,intron_5:17868133-17868999,exon_6:17868999-17869189,intron_6:17869189-17874224,exon_4:17866790-17866949,exon_8:17877540-17877703,intron_7:17874354-17877540,intron_3:17865758-17866790,; LDM_E 3 234 DDX10

oar_circ_0000899 chr15 17877540 17913843 + 36303 35930 ENSOARG00000012714; intron_8:17877703-17883889,intron_12:17910799-17913362,intron_11:17907790-17910710,intron_10:17905983-17907693,intron_9:17883974-17905881,exon_8:17877540-17877703,exon_13:17913362-17913843,; LDM_A 4 370 DDX10

oar_circ_0000900 chr15 19682862 19693361 + 10499 10499 ENSOARG00000012999; exon_2:19692612-19693361,intron_1:19647875-19692612,; LDM_E,LDM_A 2,3 665,1136 ZC3H12C

oar_circ_0000901 chr15 19949212 19967066 + 17854 17854 ENSOARG00000026813;ENSOARG00000013370; intron_1:19931895-19949968,;intron_2:19949337-19966936,; LDM_A 2 387 --;FDX1

oar_circ_0000902 chr15 23198991 23199159 + 168 168 ENSOARG00000016809; exon_3:23198940-23199159,; LDM_E 3 12 NCAM1

oar_circ_0000903 chr15 23226569 23229284 + 2715 2486 ENSOARG00000016809; exon_13:23226977-23227148,intron_12:23226666-23226977,intron_13:23227148-23229152,; LDM_E 2 88 NCAM1

oar_circ_0000904 chr15 24061736 24063074 + 1338 1338 -- -- LDM_A 5 0 --

oar_circ_0000905 chr15 24245984 24246284 + 300 300 -- -- LDM_A 2 0 --

oar_circ_0000906 chr15 25250996 25262590 - 11594 11150 ENSOARG00000001539; intron_8:25251155-25253604,exon_3:25260707-25260860,intron_10:25260860-25262443,intron_9:25253742-25260707,; LDM_A 2 57 CADM1

oar_circ_0000907 chr15 26904051 26904678 - 627 185 ENSOARG00000001906; exon_9:26904051-26904236,; LDM_A 2 4 BUD13

oar_circ_0000908 chr15 26974374 26989096 + 14722 14722 -- -- LDM_E 2 0 --

oar_circ_0000909 chr15 27082721 27120444 - 37723 37542 ENSOARG00000003139; intron_21:27082883-27116675,exon_5:27082721-27082883,intron_22:27116739-27120327,; LDM_E,LDM_A 4,4 1158,776 SIK3

oar_circ_0000910 chr15 27405103 27406332 - 1229 1094 ENSOARG00000004501; exon_4:27405103-27405269,intron_13:27405269-27406197,; LDM_E,LDM_A 10,5 4,3 PCSK7

oar_circ_0000911 chr15 28157865 28158771 + 906 791 ENSOARG00000007625; exon_3:28158580-28158771,intron_2:28157980-28158580,; LDM_A 2 9 IL10RA

oar_circ_0000912 chr15 28556550 28563762 + 7212 6755 ENSOARG00000009505; exon_13:28559576-28559764,intron_13:28559764-28561752,intron_11:28556855-28558413,exon_16:28563587-28563762,exon_11:28556550-28556855,intron_12:28558546-28559576,intron_15:28562076-28563587,; LDM_E 2 246 UBE4A

oar_circ_0000913 chr15 29146465 29147359 + 894 351 ENSOARG00000012802; exon_11:29147197-29147359,exon_10:29146903-29147092,; LDM_E 2 10 VPS11

oar_circ_0000914 chr15 29146903 29148387 + 1484 991 ENSOARG00000012802; intron_11:29147359-29147777,exon_10:29146903-29147092,exon_14:29148165-29148387,exon_11:29147197-29147359,; LDM_A 2 21 VPS11

oar_circ_0000915 chr15 29321902 29328585 + 6683 5863 ENSOARG00000015025; intron_6:29325712-29327602,exon_4:29324139-29324296,intron_7:29327690-29328056,intron_5:29325241-29325574,intron_3:29322049-29324139,intron_4:29324296-29325119,exon_9:29328381-29328585,; LDM_E 3 201 CBL

oar_circ_0000916 chr15 31197464 31211496 + 14032 12054 ENSOARG00000018098; exon_3:31205399-31205581,intron_4:31207137-31209808,exon_4:31206880-31207137,intron_2:31199802-31205399,intron_1:31197614-31199662,intron_3:31205581-31206880,; LDM_E,LDM_A 12,4 166,16 TBCEL

oar_circ_0000917 chr15 31706485 31707563 + 1078 863 ENSOARG00000018274; intron_15:31706614-31707477,; LDM_E 2 3 SORL1

oar_circ_0000918 chr15 33086177 33087116 - 939 939 -- -- LDM_A 2 0 --

oar_circ_0000919 chr15 34945228 34949838 + 4610 3682 ENSOARG00000004372; intron_17:34949300-34949657,exon_18:34949657-34949838,exon_15:34947986-34948175,intron_15:34948175-34948630,intron_14:34945858-34947986,intron_13:34945340-34945712,; LDM_E 2 81 PIK3C2A

oar_circ_0000920 chr15 34947986 34957627 + 9641 8500 ENSOARG00000004372; intron_21:34955489-34957484,intron_20:34953948-34955359,intron_18:34949838-34952957,intron_15:34948175-34948630,exon_15:34947986-34948175,intron_19:34953062-34953855,exon_18:34949657-34949838,intron_17:34949300-34949657,; LDM_E 2 188 PIK3C2A

oar_circ_0000921 chr15 35624191 35641118 + 16927 16927 ENSOARG00000005819; intron_1:35537561-35689457,; LDM_A 2 559 SOX6

oar_circ_0000922 chr15 35689457 35689698 + 241 241 ENSOARG00000005819; exon_2:35689457-35689698,; LDM_A 2 36 SOX6

oar_circ_0000923 chr15 35882413 35885440 + 3027 2958 ENSOARG00000005819; intron_5:35882586-35885371,exon_5:35882413-35882586,; LDM_A 11 126 SOX6

oar_circ_0000924 chr15 35961415 35975386 + 13971 13644 ENSOARG00000005819; intron_7:35961539-35973698,intron_8:35973778-35975263,; LDM_A 3 431 SOX6

oar_circ_0000925 chr15 35961418 35975386 + 13968 13644 ENSOARG00000005819; intron_7:35961539-35973698,intron_8:35973778-35975263,; LDM_A 3 431 SOX6

oar_circ_0000926 chr15 36010417 36020176 + 9759 9759 ENSOARG00000005819; exon_10:36010417-36010567,exon_12:36019988-36020176,intron_10:36010567-36016763,intron_11:36016947-36019988,exon_11:36016763-36016947,; LDM_E,LDM_A 4,4 494,188 SOX6

oar_circ_0000927 chr15 36010420 36020176 + 9756 9609 ENSOARG00000005819; exon_12:36019988-36020176,intron_10:36010567-36016763,intron_11:36016947-36019988,exon_11:36016763-36016947,; LDM_A 2 187 SOX6

oar_circ_0000928 chr15 36087070 36090013 + 2943 2943 ENSOARG00000005819; intron_14:36087304-36089796,exon_14:36087070-36087304,exon_15:36089796-36090013,; LDM_A 2 45 SOX6

oar_circ_0000929 chr15 36858187 36858371 - 184 184 -- -- LDM_A 2 0 --

oar_circ_0000930 chr15 37465036 37479271 + 14235 14093 ENSOARG00000006429; exon_3:37478638-37479271,intron_2:37465178-37478638,; LDM_E,LDM_A 3,2 481,80 CYP2R1

oar_circ_0000931 chr15 37465036 37483557 + 18521 18258 ENSOARG00000006429; intron_2:37465178-37478638,intron_3:37479271-37480835,exon_3:37478638-37479271,intron_4:37481165-37483436,exon_4:37480835-37481165,; LDM_E 5 729 CYP2R1

oar_circ_0000932 chr15 37478638 37481165 + 2527 2527 ENSOARG00000006429; exon_4:37480835-37481165,exon_3:37478638-37479271,intron_3:37479271-37480835,; LDM_E 8 407 CYP2R1

oar_circ_0000933 chr15 37478638 37483557 + 4919 4798 ENSOARG00000006429; intron_3:37479271-37480835,intron_4:37481165-37483436,exon_4:37480835-37481165,exon_3:37478638-37479271,; LDM_E 15 547 CYP2R1

oar_circ_0000934 chr15 37528885 37539988 - 11103 10659 ENSOARG00000006517; intron_7:37530304-37532629,exon_8:37533757-37533912,exon_6:37539786-37539988,intron_10:37539214-37539786,intron_8:37532767-37533757,intron_9:37533912-37539134,intron_6:37528999-37530192,; LDM_E 2 374 PDE3B

oar_circ_0000935 chr15 37539134 37539988 - 854 774 ENSOARG00000006517; intron_10:37539214-37539786,exon_6:37539786-37539988,; LDM_E 2 27 PDE3B

oar_circ_0000936 chr15 37827046 37827868 + 822 822 ENSOARG00000006938; exon_3:37827698-37827868,exon_2:37827043-37827276,intron_2:37827276-37827698,; LDM_E 2 79 COPB1

oar_circ_0000937 chr15 37827046 37836422 + 9376 8758 ENSOARG00000006938; exon_9:37836239-37836422,intron_7:37834722-37836084,intron_4:37829610-37830815,intron_2:37827276-37827698,intron_5:37830908-37832622,intron_6:37832760-37834602,exon_3:37827698-37827868,intron_3:37827868-37829495,exon_2:37827043-37827276,; LDM_E,LDM_A 10,5 341,225 COPB1

oar_circ_0000938 chr15 37832622 37836422 + 3800 3387 ENSOARG00000006938; intron_6:37832760-37834602,exon_9:37836239-37836422,intron_7:37834722-37836084,; LDM_E,LDM_A 12,3 73,59 COPB1

oar_circ_0000939 chr15 37837711 37839399 + 1688 1445 ENSOARG00000006938; intron_10:37837857-37839302,; LDM_E,LDM_A 5,2 73,33 COPB1

oar_circ_0000940 chr15 38016002 38017379 + 1377 906 ENSOARG00000007137; intron_1:38016090-38016996,; LDM_E,LDM_A 14,18 64,50 RRAS2

oar_circ_0000941 chr15 38016002 38026331 + 10329 9739 ENSOARG00000007137; intron_1:38016090-38016996,intron_3:38017379-38026212,; LDM_A 2 345 RRAS2

oar_circ_0000942 chr15 38016002 38028562 + 12560 11978 ENSOARG00000007137; intron_4:38026331-38028399,intron_3:38017379-38026212,intron_2:38017099-38017270,intron_1:38016090-38016996,; LDM_E 8 797 RRAS2

oar_circ_0000943 chr15 38601314 38611540 - 10226 10226 ENSOARG00000007397; intron_10:38601490-38611344,exon_1:38611344-38611540,exon_2:38601314-38601490,; LDM_A 2 170 FAR1

oar_circ_0000944 chr15 38835168 38836134 + 966 966 ENSOARG00000007619; intron_1:38832877-38835979,exon_2:38835979-38836134,; LDM_E 2 30 BTBD10

oar_circ_0000945 chr15 39543819 39544075 - 256 256 ENSOARG00000008069; exon_2:39543819-39544075,; LDM_E,LDM_A 3,9 120,89 TEAD1

oar_circ_0000946 chr15 39831051 39847531 - 16480 15991 ENSOARG00000008298; intron_9:39831192-39844299,intron_10:39844402-39847286,; LDM_E 2 169 PARVA

oar_circ_0000947 chr15 4027108 4031881 + 4773 4773 ENSOARG00000003293; exon_2:4027108-4027313,intron_2:4027313-4031700,exon_3:4031700-4031881,; LDM_E 3 97 PDGFD

oar_circ_0000948 chr15 40291854 40294853 + 2999 2777 ENSOARG00000009341; intron_2:40291992-40294769,; LDM_E 3 25 DKK3

oar_circ_0000949 chr15 40349541 40350145 - 604 523 ENSOARG00000009607; exon_23:40349969-40350145,intron_7:40349622-40349969,; LDM_A 4 70 USP47

oar_circ_0000950 chr15 40404654 40415123 - 10469 10219 ENSOARG00000009607; exon_4:40409803-40410007,intron_25:40404793-40406157,intron_27:40410007-40415063,intron_26:40406271-40409866,; LDM_E 12 1371 USP47

oar_circ_0000951 chr15 42196390 42199846 + 3456 3116 ENSOARG00000011668; intron_3:42197947-42199740,intron_2:42196513-42197836,; LDM_A 3 30 SBF2

oar_circ_0000952 chr15 42224224 42225704 + 1480 1238 ENSOARG00000011668; intron_5:42224357-42225595,; LDM_A 2 9 SBF2

oar_circ_0000953 chr15 42224224 42228029 + 3805 3449 ENSOARG00000011668; intron_5:42224357-42225595,intron_6:42225704-42227915,; LDM_E 4 141 SBF2

oar_circ_0000954 chr15 42224224 42236846 + 12622 11306 ENSOARG00000011668; intron_10:42232874-42236747,intron_8:42230983-42232091,intron_7:42228029-42230905,intron_6:42225704-42227915,intron_5:42224357-42225595,; LDM_E,LDM_A 2,3 513,151 SBF2

oar_circ_0000955 chr15 42227915 42236846 + 8931 7857 ENSOARG00000011668; intron_10:42232874-42236747,intron_8:42230983-42232091,intron_7:42228029-42230905,; LDM_A 4 111 SBF2

oar_circ_0000956 chr15 42230905 42256002 + 25097 24417 ENSOARG00000011668; intron_13:42253819-42255852,intron_10:42232874-42236747,intron_8:42230983-42232091,intron_12:42251230-42253709,intron_11:42236846-42251025,intron_9:42232205-42232745,exon_12:42251025-42251230,; LDM_E 4 965 SBF2

oar_circ_0000957 chr15 42236747 42251230 + 14483 14384 ENSOARG00000011668; exon_12:42251025-42251230,intron_11:42236846-42251025,; LDM_E 3 617 SBF2

oar_circ_0000958 chr15 42380226 42395585 + 15359 15011 ENSOARG00000011668; intron_19:42381718-42383179,exon_24:42395386-42395585,exon_22:42389889-42390065,intron_20:42383375-42388218,intron_23:42393190-42395386,exon_18:42380226-42380399,intron_22:42390065-42393044,intron_21:42388346-42389889,exon_20:42383179-42383375,intron_18:42380399-42381644,; LDM_E 2 822 SBF2

oar_circ_0000959 chr15 42498972 42503060 - 4088 3832 ENSOARG00000012146; exon_7:42498972-42499154,intron_6:42499154-42501693,intron_7:42501802-42502913,; LDM_A 2 26 SWAP70

oar_circ_0000960 chr15 42693664 42702301 - 8637 8511 ENSOARG00000012477; exon_10:42700357-42700537,intron_5:42693892-42700357,exon_11:42693664-42693892,intron_6:42700537-42702175,; LDM_A 2 128 ZNF143

oar_circ_0000961 chr15 42757639 42759541 - 1902 1399 ENSOARG00000012687; intron_9:42758468-42759412,intron_7:42757737-42758192,; LDM_E 2 56 IPO7

oar_circ_0000962 chr15 42763930 42777447 - 13517 12536 ENSOARG00000012687; intron_14:42764047-42764398,intron_16:42765345-42766003,intron_21:42771969-42775306,intron_15:42764475-42765245,exon_4:42775306-42775465,intron_22:42775465-42777293,exon_5:42771812-42771969,intron_17:42766138-42768012,intron_19:42768320-42770537,intron_20:42770627-42771812,; LDM_A 2 767 IPO7

oar_circ_0000963 chr15 42974317 42974975 + 658 658 ENSOARG00000013078; exon_3:42974317-42974975,; LDM_E 2 316 DENND5A

oar_circ_0000964 chr15 42992445 42998654 + 6209 5983 ENSOARG00000013078; intron_9:42993362-42996517,intron_10:42996649-42998501,intron_8:42992596-42993268,exon_8:42992445-42992596,exon_11:42998501-42998654,; LDM_E 2 327 DENND5A

oar_circ_0000965 chr15 43013667 43017097 + 3430 2958 ENSOARG00000013078; exon_18:43014855-43015037,intron_17:43014488-43014855,exon_21:43016928-43017097,intron_19:43015726-43016187,intron_18:43015037-43015643,intron_20:43016311-43016928,intron_16:43013813-43014369,; LDM_E,LDM_A 6,2 168,59 DENND5A

oar_circ_0000966 chr15 43952036 43955833 + 3797 3627 ENSOARG00000015030; exon_2:43952036-43952263,intron_2:43952263-43953449,intron_3:43953525-43955739,; LDM_A 2 55 RIC3

oar_circ_0000967 chr15 43952036 43963664 + 11628 11309 ENSOARG00000015030; intron_3:43953525-43955739,intron_2:43952263-43953449,exon_2:43952036-43952263,intron_4:43955833-43963515,; LDM_A 17 130 RIC3

oar_circ_0000968 chr15 44606308 44622789 - 16481 16142 ENSOARG00000015882; intron_19:44606440-44618593,intron_20:44618707-44622696,; LDM_E,LDM_A 2,2 394,105 PPFIBP2

oar_circ_0000969 chr15 44822153 44826243 - 4090 3839 ENSOARG00000016115; intron_1:44822283-44824352,exon_4:44824352-44824524,intron_2:44824524-44826122,; LDM_E 8 41 SYT9

oar_circ_0000970 chr15 44900845 44901392 - 547 547 ENSOARG00000016115; exon_2:44900845-44901392,; LDM_E 10 90 SYT9

oar_circ_0000971 chr15 44900845 44911669 - 10824 10824 ENSOARG00000016115; exon_1:44911317-44911669,intron_4:44901392-44911317,exon_2:44900845-44901392,; LDM_E 6 194 SYT9

oar_circ_0000972 chr15 45714607 45750754 - 36147 36147 -- -- LDM_A 7 0 --

oar_circ_0000973 chr15 46059800 46061085 - 1285 1057 ENSOARG00000018629; intron_5:46059890-46060947,; LDM_E 2 1 SMPD1

oar_circ_0000974 chr15 47567058 47610631 + 43573 1440 ENSOARG00000019174;ENSOARG00000019163; exon_2:47610408-47610631,exon_1:47609515-47610279,;exon_3:47567058-47567511,; LDM_E 15 2907 -;-

oar_circ_0000975 chr15 4800999 4815148 - 14149 13812 ENSOARG00000003665; exon_81:4806035-4806244,intron_10:4812941-4814992,intron_8:4801104-4806035,intron_9:4806244-4812865,; LDM_E 3 73 DYNC2H1

oar_circ_0000976 chr15 4874489 4891593 - 17104 15844 ENSOARG00000003665; intron_28:4886415-4888665,intron_29:4888792-4891506,intron_26:4876317-4885592,intron_25:4874613-4876218,; LDM_E 2 93 DYNC2H1

oar_circ_0000977 chr15 49579710 49592805 + 13095 12727 ENSOARG00000002200; exon_14:49592617-49592805,intron_11:49579803-49585997,intron_12:49586138-49590863,intron_13:49590997-49592617,; LDM_E 4 953 NUP98

oar_circ_0000978 chr15 49585997 49592805 + 6808 6533 ENSOARG00000002200; exon_14:49592617-49592805,intron_13:49590997-49592617,intron_12:49586138-49590863,; LDM_A 7 267 NUP98

oar_circ_0000979 chr15 49585997 49605985 + 19988 19343 ENSOARG00000002200; exon_16:49601913-49602212,intron_15:49600747-49601913,intron_16:49602212-49604517,intron_12:49586138-49590863,intron_13:49590997-49592617,intron_14:49592805-49600630,intron_17:49604631-49605846,exon_14:49592617-49592805,; LDM_E,LDM_A 2,5 862,646 NUP98

oar_circ_0000980 chr15 49815246 49821955 + 6709 6480 ENSOARG00000003290; intron_5:49818987-49821834,intron_4:49815401-49818879,exon_4:49815246-49815401,; LDM_E 4 255 RNF121

oar_circ_0000981 chr15 5017266 5030311 - 13045 11766 ENSOARG00000003665; exon_6:5029155-5029388,exon_11:5024673-5024849,exon_12:5021516-5021712,intron_84:5029388-5030166,intron_83:5026899-5029155,intron_78:5021712-5024673,intron_77:5017362-5021516,intron_79:5024849-5025861,; LDM_E 2 160 DYNC2H1

oar_circ_0000982 chr15 5037842 5038551 - 709 171 ENSOARG00000003665; exon_2:5038380-5038551,; LDM_A 2 3 DYNC2H1

oar_circ_0000983 chr15 50699503 50704639 - 5136 4818 ENSOARG00000006995; intron_14:50700741-50704505,intron_13:50699632-50700686,; LDM_E,LDM_A 12,3 171,28 FCHSD2

oar_circ_0000984 chr15 52114417 52123859 + 9442 9059 ENSOARG00000010224; intron_9:52117894-52123749,intron_8:52114583-52117787,; LDM_A 2 37 POLD3

oar_circ_0000985 chr15 52419693 52422158 + 2465 2246 ENSOARG00000010824; intron_2:52419777-52420376,intron_3:52420549-52422023,exon_3:52420376-52420549,; LDM_E,LDM_A 3,6 116,88 SPCS2

oar_circ_0000986 chr15 52419693 52431914 + 12221 11193 ENSOARG00000010824; intron_2:52419777-52420376,exon_3:52420376-52420549,intron_4:52422158-52430918,intron_3:52420549-52422023,exon_5:52430918-52431105,; LDM_E 3 693 SPCS2

oar_circ_0000987 chr15 54056600 54073127 + 16527 16333 ENSOARG00000012864; exon_14:54058797-54058996,intron_14:54058996-54067086,intron_16:54069112-54070120,intron_15:54067251-54068956,exon_13:54056600-54056774,intron_17:54070162-54072975,exon_16:54068956-54069112,intron_13:54056774-54058797,exon_15:54067086-54067251,; LDM_A 2 386 C11orf30

oar_circ_0000988 chr15 57136039 57143033 + 6994 6994 ENSOARG00000015410; exon_4:57136039-57136231,intron_4:57136231-57142854,exon_5:57142854-57143033,; LDM_E 2 62 METTL15

oar_circ_0000989 chr15 5793590 5802277 - 8687 8687 -- -- LDM_E 3 0 --

oar_circ_0000990 chr15 5864726 5864897 + 171 171 ENSOARG00000006266; exon_5:5864675-5864897,; LDM_E 4 44 TMEM123

oar_circ_0000991 chr15 5868874 5870327 + 1453 1453 -- -- LDM_E 4 0 --

oar_circ_0000992 chr15 5913778 5921335 - 7557 7557 ENSOARG00000006356; intron_1:5861885-5954522,; LDM_E 2 1422 BIRC3

oar_circ_0000993 chr15 5920651 5920917 - 266 266 ENSOARG00000006356; intron_1:5861885-5954522,; LDM_E 2 77 BIRC3

oar_circ_0000994 chr15 59269922 59271786 + 1864 1736 ENSOARG00000015515; intron_1:59270410-59271658,exon_1:59269922-59270410,; LDM_A 10 216 ARL14EP

oar_circ_0000995 chr15 5963983 5969077 - 5094 3077 ENSOARG00000006356; exon_1:5965886-5967347,intron_7:5964062-5965678,; LDM_E 2 433 BIRC3

oar_circ_0000996 chr15 60455322 60461015 - 5693 5343 ENSOARG00000015999; intron_3:60455449-60458886,intron_4:60458975-60460881,; LDM_A 2 113 IMMP1L

oar_circ_0000997 chr15 61505932 61507540 + 1608 1020 ENSOARG00000016580; intron_3:61506930-61507224,intron_2:61506065-61506791,; LDM_E 6 52 EIF3M

oar_circ_0000998 chr15 61505932 61518433 + 12501 11503 ENSOARG00000016580; intron_3:61506930-61507224,intron_2:61506065-61506791,intron_8:61514869-61518289,intron_5:61507540-61513158,intron_7:61514211-61514787,intron_6:61513242-61514111,; LDM_E 4 409 EIF3M

oar_circ_0000999 chr15 61866502 61867642 + 1140 1012 ENSOARG00000017028; intron_2:61866630-61867452,exon_3:61867452-61867642,; LDM_E 2 3 DEPDC7

oar_circ_0001000 chr15 62112602 62113701 + 1099 1099 ENSOARG00000017375; exon_2:62112602-62113701,; LDM_E,LDM_A 68,6 1525,1520 HIPK3

oar_circ_0001001 chr15 62145615 62156777 + 11162 10707 ENSOARG00000017375; intron_6:62155336-62156089,intron_3:62145739-62153849,intron_4:62153969-62154573,intron_7:62156249-62156653,exon_6:62155151-62155336,exon_7:62156089-62156249,intron_5:62154660-62155151,; LDM_E 2 1162 HIPK3

oar_circ_0001002 chr15 62153849 62156777 + 2928 2597 ENSOARG00000017375; intron_5:62154660-62155151,exon_7:62156089-62156249,exon_6:62155151-62155336,intron_4:62153969-62154573,intron_7:62156249-62156653,intron_6:62155336-62156089,; LDM_E 2 305 HIPK3

oar_circ_0001003 chr15 62167686 62168090 - 404 404 -- -- LDM_A 2 0 --

oar_circ_0001004 chr15 63189746 63195585 + 5839 5608 ENSOARG00000018566; intron_7:63194974-63195432,intron_5:63189851-63190441,intron_6:63190567-63194782,exon_8:63195432-63195585,exon_7:63194782-63194974,; LDM_E,LDM_A 4,7 131,127 CAT

oar_circ_0001005 chr15 63199260 63202071 + 2811 2541 ENSOARG00000018566; intron_9:63199399-63201940,; LDM_A 7 17 CAT

oar_circ_0001006 chr15 63666930 63678629 + 11699 11499 ENSOARG00000018745; intron_3:63667031-63676780,exon_4:63676780-63676980,intron_4:63676980-63678530,; LDM_A 2 246 PDHX

oar_circ_0001007 chr15 65106516 65115129 + 8613 8253 ENSOARG00000019047; intron_5:65106608-65111073,intron_6:65111214-65115002,; LDM_E 4 71 PRR5L

oar_circ_0001008 chr15 6981617 6982392 + 775 775 ENSOARG00000007395; exon_1:6981286-6982392,; LDM_E 2 42 TRPC6

oar_circ_0001009 chr15 71636561 71639255 + 2694 2534 ENSOARG00000000187; exon_2:71636561-71636723,intron_3:71638013-71638510,intron_2:71636723-71637919,intron_4:71638576-71639103,exon_5:71639103-71639255,; LDM_E,LDM_A 6,2 106,62 API5

oar_circ_0001010 chr15 71705542 71709223 + 3681 3204 ENSOARG00000000479; exon_15:71708998-71709223,intron_12:71707289-71707445,intron_11:71705689-71707173,exon_13:71707445-71707611,intron_13:71707611-71708784,; LDM_E 2 166 TTC17

oar_circ_0001011 chr15 71705542 71716126 + 10584 10107 ENSOARG00000000479; exon_15:71708998-71709223,intron_15:71709223-71715939,intron_12:71707289-71707445,intron_11:71705689-71707173,exon_13:71707445-71707611,exon_16:71715939-71716126,intron_13:71707611-71708784,; LDM_E 2 534 TTC17

oar_circ_0001012 chr15 71705542 71736492 + 30950 30317 ENSOARG00000000479; exon_15:71708998-71709223,exon_13:71707445-71707611,intron_13:71707611-71708784,intron_11:71705689-71707173,exon_17:71736321-71736492,exon_16:71715939-71716126,intron_15:71709223-71715939,intron_16:71716126-71736321,; LDM_E,LDM_A 6,9 1714,605 TTC17

oar_circ_0001013 chr15 71707173 71709223 + 2050 1564 ENSOARG00000000479; intron_13:71707611-71708784,exon_15:71708998-71709223,exon_13:71707445-71707611,; LDM_A 2 57 TTC17

oar_circ_0001014 chr15 71749498 71759012 + 9514 9149 ENSOARG00000000479; intron_19:71750429-71755628,exon_18:71749498-71749758,exon_19:71750275-71750429,intron_18:71749758-71750275,intron_20:71755749-71757837,intron_21:71757997-71758928,; LDM_A 2 139 TTC17

oar_circ_0001015 chr15 72174254 72186381 + 12127 11934 ENSOARG00000001123; exon_7:72186171-72186381,intron_5:72174358-72175918,intron_6:72176007-72186171,; LDM_E 2 412 ALKBH3

oar_circ_0001016 chr15 73018176 73027447 + 9271 9271 -- -- LDM_E 4 0 --

oar_circ_0001017 chr15 73428110 73429507 + 1397 1168 ENSOARG00000002066; intron_2:73428235-73428559,intron_3:73428663-73429244,exon_4:73429244-73429507,; LDM_E 4 29 PRDM11

oar_circ_0001018 chr15 74339151 74346193 - 7042 6858 ENSOARG00000003032; intron_16:74339294-74346152,; LDM_E 2 354 PHF21A

oar_circ_0001019 chr15 74659081 74660186 - 1105 1105 ENSOARG00000003608; exon_13:74659997-74660186,intron_6:74659236-74659997,exon_14:74659081-74659236,; LDM_E,LDM_A 13,9 22,18 AMBRA1

oar_circ_0001020 chr15 74716866 74729560 - 12694 12512 ENSOARG00000003608; exon_9:74729380-74729560,intron_9:74716967-74717418,intron_10:74717499-74729380,; LDM_E 7 322 AMBRA1

oar_circ_0001021 chr15 74716866 74733992 - 17126 16857 ENSOARG00000003608; intron_10:74717499-74729380,intron_9:74716967-74717418,intron_11:74729560-74733905,exon_9:74729380-74729560,; LDM_E 2 461 AMBRA1

oar_circ_0001022 chr15 74729380 74733992 - 4612 4525 ENSOARG00000003608; exon_9:74729380-74729560,intron_11:74729560-74733905,; LDM_A 5 64 AMBRA1

oar_circ_0001023 chr15 7490919 7491225 + 306 306 ENSOARG00000007837; exon_2:7490919-7491225,; LDM_A 3 8 PGR

oar_circ_0001024 chr15 74913110 74914146 - 1036 1036 -- -- LDM_E 2 0 --

oar_circ_0001025 chr15 74929192 74930689 - 1497 1497 ENSOARG00000004217; exon_33:74930516-74930689,intron_11:74929366-74930516,exon_34:74929192-74929366,; LDM_E 11 68 CKAP5

oar_circ_0001026 chr15 75033795 75034291 - 496 219 ENSOARG00000004702; exon_32:75034072-75034291,; LDM_A 2 4 LRP4

oar_circ_0001027 chr15 75041729 75046943 - 5214 4587 ENSOARG00000004702; exon_17:75043802-75044011,intron_26:75046001-75046388,exon_21:75041729-75041931,intron_21:75041931-75042675,intron_27:75046565-75046725,intron_23:75042923-75043546,intron_25:75044011-75045878,exon_14:75046725-75046943,exon_15:75046388-75046565,; LDM_E 2 129 LRP4

oar_circ_0001028 chr15 75765625 75771128 - 5503 4172 ENSOARG00000007967; intron_17:75769153-75770055,intron_14:75766891-75767609,intron_13:75766302-75766771,intron_16:75768362-75768993,intron_15:75767708-75768292,intron_18:75770126-75770994,; LDM_E 2 41 NUP160

oar_circ_0001029 chr15 77743599 77743960 + 361 361 -- -- LDM_E,LDM_A 2,4 0,0 --

oar_circ_0001030 chr15 77743599 77744023 + 424 424 -- -- LDM_E,LDM_A 3,3 0,0 --

oar_circ_0001031 chr15 77743646 77744070 - 424 424 -- -- LDM_E,LDM_A 3,3 0,0 --

oar_circ_0001032 chr15 80446075 80446889 - 814 192 ENSOARG00000013282; exon_18:80446697-80446889,; LDM_E,LDM_A 5,7 14,8 NCAPD3

oar_circ_0001033 chr16 10022944 10029821 - 6877 6364 ENSOARG00000005347; exon_27:10023694-10024090,exon_21:10029613-10029821,intron_18:10028681-10029613,intron_17:10025537-10028544,intron_15:10024082-10024889,exon_23:10025335-10025537,intron_14:10023070-10023694,exon_24:10024889-10025077,; LDM_E,LDM_A 13,6 430,193 BDP1

oar_circ_0001034 chr16 10028544 10029821 - 1277 1140 ENSOARG00000005347; intron_18:10028681-10029613,exon_21:10029613-10029821,; LDM_A 2 28 BDP1

oar_circ_0001035 chr16 10051670 10064168 - 12498 12204 ENSOARG00000005347; intron_31:10056021-10057721,intron_30:10051940-10055877,intron_32:10057776-10064073,exon_10:10051670-10051940,; LDM_E 2 431 BDP1

oar_circ_0001036 chr16 10068758 10069931 - 1173 1003 ENSOARG00000005347; intron_36:10068818-10069821,; LDM_A 2 2 BDP1

oar_circ_0001037 chr16 10243830 10250681 - 6851 6572 ENSOARG00000005551; intron_7:10247819-10250564,intron_6:10243992-10247140,exon_2:10247140-10247819,; LDM_E 2 51 OCLN

oar_circ_0001038 chr16 10337018 10341156 - 4138 3526 ENSOARG00000005629; intron_4:10337168-10338385,intron_7:10339249-10341044,exon_11:10339063-10339249,intron_6:10338735-10339063,; LDM_E 2 75 RAD17

oar_circ_0001039 chr16 10354099 10354987 - 888 258 ENSOARG00000005629; exon_3:10354729-10354987,; LDM_E,LDM_A 6,8 19,6 RAD17

oar_circ_0001040 chr16 10462143 10463651 - 1508 816 ENSOARG00000005750; intron_1:10462835-10463487,exon_8:10463487-10463651,; LDM_E 11 14 CENPH

oar_circ_0001041 chr16 10528227 10534713 - 6486 6210 ENSOARG00000005799; intron_14:10529775-10534590,intron_13:10528313-10529708,; LDM_A 2 26 SLC30A5

oar_circ_0001042 chr16 11469839 11472152 - 2313 614 ENSOARG00000005870; exon_2:11469839-11470453,; LDM_A 2 841 PIK3R1

oar_circ_0001043 chr16 12423450 12423912 - 462 462 ENSOARG00000005937; intron_1:12423124-12423978,; LDM_E 2 11 MAST4

oar_circ_0001044 chr16 12479304 12486351 - 7047 6869 ENSOARG00000005937; exon_10:12481406-12481616,intron_21:12481616-12483498,exon_8:12486193-12486351,intron_20:12479406-12481406,intron_22:12483574-12486193,; LDM_E,LDM_A 5,4 324,367 MAST4

oar_circ_0001045 chr16 12538761 12540035 - 1274 1274 ENSOARG00000005937; intron_25:12498178-12539946,; LDM_A 2 177 MAST4

oar_circ_0001046 chr16 13545458 13548873 - 3415 3415 ENSOARG00000006023; intron_7:13547001-13548689,exon_20:13548689-13548873,exon_21:13545458-13547001,; LDM_A 3 755 ERBB2IP

oar_circ_0001047 chr16 13548689 13555435 - 6746 6746 ENSOARG00000006023; exon_20:13548689-13548873,intron_8:13548873-13550979,exon_18:13554499-13554685,intron_9:13551094-13554499,intron_10:13554685-13555737,; LDM_E,LDM_A 6,3 852,335 ERBB2IP

oar_circ_0001048 chr16 13581009 13600616 - 19607 19199 ENSOARG00000006023; intron_20:13581073-13585206,intron_23:13589999-13600498,intron_22:13587462-13589920,intron_21:13585263-13587372,; LDM_A 5 759 ERBB2IP

oar_circ_0001049 chr16 13585206 13600616 - 15410 15066 ENSOARG00000006023; intron_23:13589999-13600498,intron_21:13585263-13587372,intron_22:13587462-13589920,; LDM_E,LDM_A 8,5 1818,556 ERBB2IP

oar_circ_0001050 chr16 13587372 13602986 - 15614 15327 ENSOARG00000006023; intron_24:13600616-13602788,exon_3:13602788-13602986,intron_22:13587462-13589920,intron_23:13589999-13600498,; LDM_E 2 1755 ERBB2IP

oar_circ_0001051 chr16 13786806 13790245 - 3439 3303 ENSOARG00000006075; exon_8:13786806-13787173,intron_6:13787173-13790109,; LDM_A 2 22 NLN

oar_circ_0001052 chr16 13969872 13985646 - 15774 15231 ENSOARG00000006289; intron_9:13983870-13985543,exon_7:13975136-13975326,exon_6:13976159-13976350,intron_6:13976350-13980358,intron_3:13971057-13972217,intron_7:13980806-13983512,exon_8:13972217-13972399,intron_2:13970055-13970975,exon_5:13980358-13980806,exon_10:13969872-13970055,intron_5:13975326-13976159,intron_4:13972399-13975136,; LDM_E 3 424 PPWD1

oar_circ_0001053 chr16 14664068 14683182 - 19114 18930 ENSOARG00000006458; intron_8:14668657-14683112,exon_10:14664068-14664226,intron_6:14664226-14667566,intron_7:14667600-14668577,; LDM_A 3 138 CWC27

oar_circ_0001054 chr16 16910079 16929316 - 19237 18769 ENSOARG00000006763; intron_7:16910160-16910585,intron_9:16914637-16921437,intron_8:16910665-16914560,intron_10:16921553-16929202,; LDM_E 2 281 IPO11

oar_circ_0001055 chr16 16979478 16993911 - 14433 14115 ENSOARG00000006763; intron_27:16981555-16989372,intron_26:16979682-16981482,intron_28:16989473-16993767,exon_4:16979478-16979682,; LDM_E,LDM_A 6,5 350,121 IPO11

oar_circ_0001056 chr16 17928418 17948865 - 20447 20151 ENSOARG00000007013; intron_12:17932279-17948508,intron_11:17928569-17932130,exon_3:17948508-17948869,; LDM_E 3 553 ZSWIM6

oar_circ_0001057 chr16 18430558 18441806 + 11248 10680 ENSOARG00000007073; intron_5:18434203-18436037,intron_6:18436106-18437267,intron_8:18440324-18441681,intron_4:18430682-18434121,intron_7:18437334-18440223,; LDM_E 3 87 ERCC8

oar_circ_0001058 chr16 20210649 20212254 - 1605 1605 -- -- LDM_A 2 0 --

oar_circ_0001059 chr16 22081920 22088595 - 6675 6431 ENSOARG00000007251; intron_3:22085259-22085714,intron_5:22087808-22088410,intron_4:22085882-22087667,exon_6:22088410-22088595,exon_9:22085071-22085259,exon_8:22085714-22085882,intron_2:22082023-22085071,; LDM_A 2 319 GPBP1

oar_circ_0001060 chr16 22085071 22088595 - 3524 3383 ENSOARG00000007251; exon_8:22085714-22085882,exon_9:22085071-22085259,exon_6:22088410-22088595,intron_4:22085882-22087667,intron_5:22087808-22088410,intron_3:22085259-22085714,; LDM_A 6 151 GPBP1

oar_circ_0001061 chr16 22344844 22347526 + 2682 2267 ENSOARG00000007314; intron_4:22344979-22346177,intron_5:22346298-22347178,intron_6:22347264-22347453,; LDM_E 2 129 MIER3

oar_circ_0001062 chr16 22349551 22353556 + 4005 3828 ENSOARG00000007314; intron_9:22352078-22353461,exon_8:22349551-22349703,intron_8:22349703-22351996,; LDM_E 5 177 MIER3

oar_circ_0001063 chr16 23547861 23562512 + 14651 14483 ENSOARG00000007689; intron_7:23552909-23555792,intron_6:23548032-23552849,exon_8:23555792-23555987,exon_6:23547861-23548032,intron_8:23555987-23562404,; LDM_E 2 388 SLC38A9

oar_circ_0001064 chr16 23552849 23562512 + 9663 9495 ENSOARG00000007689; intron_8:23555987-23562404,intron_7:23552909-23555792,exon_8:23555792-23555987,; LDM_A 2 90 SLC38A9

oar_circ_0001065 chr16 23587227 23593536 + 6309 5956 ENSOARG00000007689; intron_11:23587341-23588898,intron_12:23589047-23593446,; LDM_E 2 150 SLC38A9

oar_circ_0001066 chr16 23722375 23756269 + 33894 33894 ENSOARG00000007720; intron_1:23684919-23749358,exon_3:23755988-23756269,exon_2:23749331-23749510,intron_2:23749510-23755988,; LDM_E,LDM_A 29,6 5131,2659 PPAP2A

oar_circ_0001067 chr16 23749358 23756269 + 6911 6911 ENSOARG00000007720; exon_2:23749331-23749510,intron_2:23749510-23755988,exon_3:23755988-23756269,; LDM_E,LDM_A 2,3 1098,605 PPAP2A

oar_circ_0001068 chr16 23749358 23758766 + 9408 9408 ENSOARG00000007720; exon_3:23755988-23756269,intron_3:23756269-23786705,intron_2:23749510-23755988,exon_2:23749331-23749510,; LDM_E,LDM_A 8,15 1475,783 PPAP2A

oar_circ_0001069 chr16 23808338 23815593 - 7255 7092 ENSOARG00000007764; intron_3:23808501-23810691,intron_4:23810748-23815487,exon_25:23808338-23808501,; LDM_E 5 320 SKIV2L2

oar_circ_0001070 chr16 23808338 23828070 - 19732 19320 ENSOARG00000007764; exon_22:23818406-23818570,intron_3:23808501-23810691,intron_7:23823832-23824896,intron_8:23825007-23827941,intron_4:23810748-23815487,intron_6:23818570-23823643,exon_25:23808338-23808501,exon_21:23823643-23823832,intron_5:23815602-23818406,; LDM_E 5 856 SKIV2L2

oar_circ_0001071 chr16 23823643 23828070 - 4427 4187 ENSOARG00000007764; exon_21:23823643-23823832,intron_7:23823832-23824896,intron_8:23825007-23827941,; LDM_E 2 124 SKIV2L2

oar_circ_0001072 chr16 23836711 23862025 - 25314 23722 ENSOARG00000007764; intron_12:23844819-23848633,intron_11:23837534-23844668,intron_21:23860905-23861850,intron_20:23859592-23860814,exon_6:23861850-23862025,intron_15:23852952-23854911,intron_10:23836780-23837361,intron_17:23856683-23857722,intron_13:23848759-23850906,intron_14:23851008-23852841,intron_16:23854991-23856551,exon_17:23837361-23837534,intron_19:23858327-23859467,; LDM_E 6 1066 SKIV2L2

oar_circ_0001073 chr16 23837361 23837532 - 171 171 ENSOARG00000007764; exon_17:23837361-23837534,; LDM_E 2 24 SKIV2L2

oar_circ_0001074 chr16 23859467 23862025 - 2558 2342 ENSOARG00000007764; exon_6:23861850-23862025,intron_20:23859592-23860814,intron_21:23860905-23861850,; LDM_E 4 118 SKIV2L2

oar_circ_0001075 chr16 24304529 24318087 + 13558 13426 ENSOARG00000008055; intron_7:24308428-24317914,intron_6:24304728-24308296,exon_6:24304529-24304728,exon_8:24317914-24318087,; LDM_E 2 284 -

oar_circ_0001076 chr16 26011865 26017654 + 5789 1152 ENSOARG00000008150; intron_2:26011987-26013139,; LDM_E 2 69 -

oar_circ_0001077 chr16 26078929 26085589 - 6660 6022 ENSOARG00000008212; intron_24:26079078-26081571,intron_26:26081968-26085497,; LDM_E 2 161 ITGA2

oar_circ_0001078 chr16 28410682 28414736 - 4054 3720 ENSOARG00000008401; intron_22:28414089-28414646,intron_20:28410777-28412058,intron_21:28412136-28414018,; LDM_E,LDM_A 2,2 154,21 PARP8

oar_circ_0001079 chr16 2845359 2857451 + 12092 11854 ENSOARG00000003427; intron_2:2849054-2857284,intron_1:2845506-2848963,exon_3:2857284-2857451,; LDM_E,LDM_A 4,2 432,110 RANBP17

oar_circ_0001080 chr16 2845359 2862783 + 17424 17120 ENSOARG00000003427; exon_3:2857284-2857451,intron_3:2857451-2862717,intron_2:2849054-2857284,intron_1:2845506-2848963,; LDM_E,LDM_A 8,6 551,136 RANBP17

oar_circ_0001081 chr16 2845359 2880227 + 34868 34118 ENSOARG00000003427; intron_1:2845506-2848963,intron_7:2872888-2875077,intron_5:2869147-2869924,exon_6:2869924-2870090,intron_2:2849054-2857284,intron_6:2870090-2872814,intron_4:2862783-2869042,intron_3:2857451-2862717,exon_10:2877840-2878013,exon_3:2857284-2857451,exon_11:2880033-2880227,intron_10:2878013-2880033,intron_8:2875197-2876541,intron_9:2876688-2877840,; LDM_E,LDM_A 9,4 1296,321 RANBP17

oar_circ_0001082 chr16 2855933 2880227 + 24294 24294 ENSOARG00000003427; intron_8:2875197-2876541,intron_6:2870090-2872814,exon_10:2877840-2878013,intron_2:2849054-2857284,exon_6:2869924-2870090,intron_10:2878013-2880033,intron_3:2857451-2862717,intron_7:2872888-2875077,exon_11:2880033-2880227,intron_5:2869147-2869924,exon_3:2857284-2857451,intron_4:2862783-2869042,intron_9:2876688-2877840,; LDM_E 3 909 RANBP17

oar_circ_0001083 chr16 2857284 2880227 + 22943 22431 ENSOARG00000003427; exon_3:2857284-2857451,exon_10:2877840-2878013,intron_6:2870090-2872814,intron_4:2862783-2869042,intron_3:2857451-2862717,intron_7:2872888-2875077,intron_5:2869147-2869924,exon_6:2869924-2870090,intron_9:2876688-2877840,intron_8:2875197-2876541,intron_10:2878013-2880033,exon_11:2880033-2880227,; LDM_E,LDM_A 37,27 871,214 RANBP17

oar_circ_0001084 chr16 2869042 2880227 + 11185 10739 ENSOARG00000003427; intron_9:2876688-2877840,intron_8:2875197-2876541,intron_10:2878013-2880033,exon_11:2880033-2880227,exon_10:2877840-2878013,intron_6:2870090-2872814,intron_5:2869147-2869924,exon_6:2869924-2870090,intron_7:2872888-2875077,; LDM_E,LDM_A 12,3 462,130 RANBP17

oar_circ_0001085 chr16 2869042 2915174 + 46132 45580 ENSOARG00000003427; intron_10:2878013-2880033,exon_6:2869924-2870090,intron_11:2880227-2915068,intron_6:2870090-2872814,exon_10:2877840-2878013,intron_8:2875197-2876541,intron_9:2876688-2877840,exon_11:2880033-2880227,intron_5:2869147-2869924,intron_7:2872888-2875077,; LDM_E 9 1176 RANBP17

oar_circ_0001086 chr16 2869042 2937633 + 68591 67903 ENSOARG00000003427; intron_9:2876688-2877840,intron_5:2869147-2869924,exon_11:2880033-2880227,intron_7:2872888-2875077,exon_6:2869924-2870090,intron_12:2915174-2937497,intron_10:2878013-2880033,intron_11:2880227-2915068,intron_6:2870090-2872814,exon_10:2877840-2878013,intron_8:2875197-2876541,; LDM_E 16 1391 RANBP17

oar_circ_0001087 chr16 29166947 29167371 + 424 424 -- -- LDM_A 4 0 --

oar_circ_0001088 chr16 31170193 31170879 - 686 552 ENSOARG00000008573; intron_14:31170385-31170745,exon_9:31170193-31170385,; LDM_E,LDM_A 2,2 17,51 NNT

oar_circ_0001089 chr16 31189803 31192769 - 2966 2877 ENSOARG00000008573; intron_16:31189991-31192680,exon_7:31189803-31189991,; LDM_A 3 82 NNT

oar_circ_0001090 chr16 31732105 31732809 + 704 704 -- -- LDM_A 4 0 --

oar_circ_0001091 chr16 32093747 32094627 - 880 880 -- -- LDM_A 34 0 --

oar_circ_0001092 chr16 32093747 32094631 - 884 884 -- -- LDM_A 17 0 --

oar_circ_0001093 chr16 32628764 32630659 + 1895 1695 ENSOARG00000008923; intron_2:32628873-32630568,; LDM_A 8 10 OXCT1

oar_circ_0001094 chr16 32628764 32651910 + 23146 22642 ENSOARG00000008923; intron_5:32642847-32649146,intron_6:32649253-32651849,exon_5:32642697-32642847,intron_4:32639665-32642697,intron_3:32630659-32639529,intron_2:32628873-32630568,; LDM_A 4 146 OXCT1

oar_circ_0001095 chr16 328699 336429 - 7730 7730 ENSOARG00000002732; intron_1:328885-330237,exon_4:336252-336429,exon_7:325917-328885,intron_2:330363-335177,intron_3:335301-336252,; LDM_E,LDM_A 10,4 649,288 PANK3

oar_circ_0001096 chr16 328699 338693 - 9994 9994 ENSOARG00000002732; intron_4:336429-338439,exon_3:338439-338693,exon_4:336252-336429,intron_1:328885-330237,intron_3:335301-336252,exon_7:325917-328885,intron_2:330363-335177,; LDM_A 2 404 PANK3

oar_circ_0001097 chr16 328699 343181 - 14482 14482 ENSOARG00000002732; exon_4:336252-336429,intron_4:336429-338439,exon_2:342828-343181,intron_3:335301-336252,intron_2:330363-335177,intron_1:328885-330237,exon_3:338439-338693,intron_5:338693-342828,exon_7:325917-328885,; LDM_E 2 1296 PANK3

oar_circ_0001098 chr16 330237 336429 - 6192 5942 ENSOARG00000002732; exon_4:336252-336429,intron_2:330363-335177,intron_3:335301-336252,; LDM_E 2 581 PANK3

oar_circ_0001099 chr16 33487688 33499953 - 12265 12041 ENSOARG00000009128; exon_15:33495545-33495737,intron_4:33495737-33499820,intron_2:33487873-33491148,exon_17:33487688-33487873,intron_3:33491239-33495545,; LDM_A 4 123 C7

oar_circ_0001100 chr16 33628262 33634166 + 5904 5523 ENSOARG00000009317; intron_2:33628404-33630080,intron_3:33630174-33634021,; LDM_E,LDM_A 2,15 139,97 PRKAA1

oar_circ_0001101 chr16 33628262 33641334 + 13072 12366 ENSOARG00000009317; exon_8:33640610-33641097,exon_7:33638461-33638686,intron_5:33634166-33636343,intron_2:33628404-33630080,intron_3:33630174-33634021,intron_6:33636431-33638461,intron_7:33638686-33640610,; LDM_E,LDM_A 4,10 443,493 PRKAA1

oar_circ_0001102 chr16 33628262 33643080 + 14818 14146 ENSOARG00000009317; intron_5:33634166-33636343,intron_7:33638686-33640610,intron_9:33641334-33642869,intron_6:33636431-33638461,exon_8:33640610-33641097,intron_2:33628404-33630080,exon_10:33642869-33643114,intron_3:33630174-33634021,exon_7:33638461-33638686,; LDM_A 3 559 PRKAA1

oar_circ_0001103 chr16 34966808 34967353 + 545 545 -- -- LDM_E 3 0 --

oar_circ_0001104 chr16 34976619 34977963 + 1344 1228 ENSOARG00000009431; intron_1:34976812-34977823,exon_1:34976595-34976812,; LDM_E,LDM_A 9,11 94,22 DAB2

oar_circ_0001105 chr16 35364368 35379580 + 15212 14778 ENSOARG00000009682; intron_8:35366845-35371284,intron_9:35371352-35374526,intron_11:35378180-35379492,intron_7:35364495-35366675,intron_10:35374594-35378097,exon_8:35366675-35366845,; LDM_E 14 1204 RICTOR

oar_circ_0001106 chr16 35364400 35379580 + 15180 14778 ENSOARG00000009682; exon_8:35366675-35366845,intron_7:35364495-35366675,intron_10:35374594-35378097,intron_11:35378180-35379492,intron_9:35371352-35374526,intron_8:35366845-35371284,; LDM_E 2 1204 RICTOR

oar_circ_0001107 chr16 35428288 35430868 - 2580 2498 ENSOARG00000009845; exon_14:35429899-35430073,intron_4:35430073-35430691,intron_3:35428370-35429899,exon_13:35430691-35430868,; LDM_A 2 10 OSMR

oar_circ_0001108 chr16 35815312 35819795 + 4483 4231 ENSOARG00000009932; exon_3:35815312-35815473,intron_3:35815473-35817105,intron_4:35817220-35819658,; LDM_E,LDM_A 2,11 227,82 --

oar_circ_0001109 chr16 35815312 35826899 + 11587 11335 ENSOARG00000009932; intron_3:35815473-35817105,intron_4:35817220-35819658,exon_3:35815312-35815473,exon_6:35826735-35826899,intron_5:35819795-35826735,; LDM_E,LDM_A 7,5 570,210 --

oar_circ_0001110 chr16 35826735 35835480 + 8745 8745 ENSOARG00000009932; exon_6:35826735-35826899,exon_7:35833003-35833178,exon_8:35835225-35835480,intron_7:35833178-35835225,intron_6:35826899-35833003,; LDM_A 2 245 --

oar_circ_0001111 chr16 36659392 36665174 - 5782 5458 ENSOARG00000010187; intron_7:36661709-36665074,intron_6:36659531-36661624,; LDM_E 2 65 WDR70

oar_circ_0001112 chr16 36972818 36981199 + 8381 7823 ENSOARG00000010431; exon_11:36979361-36980131,intron_6:36972975-36973403,exon_12:36980707-36980916,intron_7:36973507-36976148,exon_6:36972818-36972975,intron_8:36976331-36978529,intron_11:36980131-36980707,exon_10:36979034-36979190,exon_8:36976148-36976331,intron_9:36978779-36979034,exon_9:36978529-36978779,; LDM_E 3 410 C5orf42

oar_circ_0001113 chr16 37097357 37100223 - 2866 2758 ENSOARG00000010580; intron_7:37098149-37100032,exon_40:37100032-37100223,exon_42:37097357-37097558,intron_6:37097558-37098041,; LDM_E,LDM_A 4,3 144,79 NIPBL

oar_circ_0001114 chr16 37103613 37104612 - 999 155 ENSOARG00000010580; exon_37:37103613-37103768,; LDM_E,LDM_A 8,16 4,1 NIPBL

oar_circ_0001115 chr16 37132237 37140817 - 8580 7899 ENSOARG00000010580; intron_29:37139024-37139589,intron_26:37134210-37136995,intron_24:37132381-37133357,exon_17:37140585-37140817,intron_27:37137134-37138396,intron_25:37133490-37134127,exon_18:37139589-37139741,intron_28:37138497-37138943,intron_30:37139741-37140585,; LDM_E 2 480 NIPBL

oar_circ_0001116 chr16 37174035 37175661 - 1626 1626 ENSOARG00000010580; exon_10:37174035-37175661,; LDM_E 2 725 NIPBL

oar_circ_0001117 chr16 37180978 37199802 - 18824 18195 ENSOARG00000010580; intron_39:37181605-37184924,intron_43:37190990-37195004,intron_44:37195132-37197054,intron_42:37190374-37190890,exon_3:37197054-37197220,intron_40:37185021-37185915,exon_6:37190222-37190374,intron_45:37197220-37199659,exon_9:37180978-37181605,intron_41:37186076-37190222,; LDM_A 2 981 NIPBL

oar_circ_0001118 chr16 37195004 37199802 - 4798 4527 ENSOARG00000010580; exon_3:37197054-37197220,intron_45:37197220-37199659,intron_44:37195132-37197054,; LDM_E 3 379 NIPBL

oar_circ_0001119 chr16 37247212 37249271 - 2059 2059 ENSOARG00000010580; intron_46:37199802-37283557,; LDM_E 3 195 NIPBL

oar_circ_0001120 chr16 37927592 37929630 + 2038 1778 ENSOARG00000010886; intron_3:37928653-37929548,intron_2:37927681-37928564,; LDM_E,LDM_A 14,34 71,10 NADK2

oar_circ_0001121 chr16 37927592 37936022 + 8430 7943 ENSOARG00000010886; intron_4:37929630-37934358,intron_3:37928653-37929548,intron_2:37927681-37928564,intron_5:37934442-37935879,; LDM_E,LDM_A 3,6 216,43 NADK2

oar_circ_0001122 chr16 37927592 37947057 + 19465 18803 ENSOARG00000010886; intron_7:37942631-37946961,intron_2:37927681-37928564,intron_3:37928653-37929548,intron_5:37934442-37935879,intron_4:37929630-37934358,intron_6:37936022-37942552,; LDM_E 3 435 NADK2

oar_circ_0001123 chr16 38016306 38030280 + 13974 13705 ENSOARG00000010980; exon_1:38016306-38016536,intron_2:38016891-38018480,exon_4:38021289-38021457,intron_1:38016536-38016793,exon_5:38022362-38022573,intron_5:38022573-38030205,intron_4:38021457-38022362,intron_3:38018576-38021289,; LDM_E 2 174 LMBRD2

oar_circ_0001124 chr16 3801698 3802402 - 704 704 -- -- LDM_A 3 0 --

oar_circ_0001125 chr16 38035227 38039926 + 4699 4361 ENSOARG00000010980; intron_10:38035361-38039086,intron_11:38039192-38039828,; LDM_A 2 31 LMBRD2

oar_circ_0001126 chr16 3943581 3950906 - 7325 7126 ENSOARG00000003730; intron_16:3943731-3950857,; LDM_E 4 48 STK10

oar_circ_0001127 chr16 39654137 39662359 + 8222 7980 ENSOARG00000011815; intron_3:39656751-39662229,intron_2:39654249-39656596,exon_3:39656596-39656751,; LDM_E 4 341 -

oar_circ_0001128 chr16 39996062 39997596 + 1534 1450 ENSOARG00000011993; intron_2:39996259-39997512,exon_2:39996062-39996259,; LDM_E 2 30 ADAMTS12

oar_circ_0001129 chr16 40078744 40080785 + 2041 1902 ENSOARG00000011993; exon_13:40078744-40078989,intron_13:40078989-40080646,; LDM_E,LDM_A 8,4 53,10 ADAMTS12

oar_circ_0001130 chr16 4045542 4046400 - 858 858 ENSOARG00000003824; intron_1:4030315-4046163,exon_1:4046163-4046401,; LDM_E,LDM_A 5,2 50,14 UBTD2

oar_circ_0001131 chr16 41455010 41463768 - 8758 8332 ENSOARG00000012358; intron_23:41460712-41463625,intron_22:41455160-41460579,; LDM_E,LDM_A 5,2 119,27 -

oar_circ_0001132 chr16 41460579 41463768 - 3189 2913 ENSOARG00000012358; intron_23:41460712-41463625,; LDM_E 2 50 -

oar_circ_0001133 chr16 41734439 41752773 - 18334 18334 -- -- LDM_E 2 0 --

oar_circ_0001134 chr16 41859559 41869060 - 9501 8959 ENSOARG00000012474; intron_4:41863093-41863864,intron_5:41863927-41865871,intron_3:41861632-41862971,exon_4:41865871-41866301,intron_2:41859699-41861565,intron_6:41866301-41868910,; LDM_A 2 154 C5orf22

oar_circ_0001135 chr16 4839120 4844043 + 4923 4797 ENSOARG00000004089; exon_4:4842956-4844043,exon_2:4839120-4839316,intron_3:4841519-4842956,intron_2:4839316-4841393,; LDM_E 5 355 CREBRF

oar_circ_0001136 chr16 4850227 4853969 + 3742 3668 ENSOARG00000004089; intron_5:4850422-4852278,exon_6:4852278-4852468,exon_5:4850227-4850422,intron_6:4852468-4853895,; LDM_A 3 63 CREBRF

oar_circ_0001137 chr16 56276147 56287857 + 11710 10452 ENSOARG00000012972; intron_15:56281150-56282081,intron_10:56277864-56279474,intron_17:56285257-56287776,intron_13:56280048-56280568,intron_16:56282164-56285148,intron_9:56276277-56277745,intron_14:56280661-56281081,; LDM_E 2 398 MYO10

oar_circ_0001138 chr16 5698128 5703710 + 5582 5373 ENSOARG00000004239; intron_5:5698302-5703147,intron_6:5703237-5703591,exon_5:5698128-5698302,; LDM_A 2 273 CPEB4

oar_circ_0001139 chr16 5698128 5709054 + 10926 10608 ENSOARG00000004239; exon_8:5708866-5709054,intron_8:5707755-5708872,intron_6:5703237-5703591,intron_5:5698302-5703147,exon_5:5698128-5698302,intron_7:5703710-5707640,; LDM_E 3 429 CPEB4

oar_circ_0001140 chr16 58451215 58452723 - 1508 1053 ENSOARG00000013160; intron_4:58451365-58452252,exon_3:58452563-58452729,; LDM_A 3 14 FAM105A

oar_circ_0001141 chr16 58624383 58625223 - 840 597 ENSOARG00000013279; intron_31:58624483-58625080,; LDM_E 3 18 TRIO

oar_circ_0001142 chr16 58694493 58704019 - 9526 9403 ENSOARG00000013279; exon_10:58694493-58694685,intron_60:58694685-58703896,; LDM_E 2 499 TRIO

oar_circ_0001143 chr16 62011453 62024574 + 13121 11691 ENSOARG00000013859; intron_13:62011627-62023318,; LDM_A 3 1 CTNND2

oar_circ_0001144 chr16 62439416 62441500 - 2084 2084 ENSOARG00000013954; intron_2:62431013-62441359,; LDM_A 2 38 ANKRD33B

oar_circ_0001145 chr16 62441359 62465984 - 24625 24354 ENSOARG00000013954; intron_3:62441500-62465854,; LDM_A 3 308 ANKRD33B

oar_circ_0001146 chr16 62663566 62665716 - 2150 2080 ENSOARG00000014077; intron_7:62663636-62665511,exon_18:62665511-62665716,; LDM_A 2 132 MARCH6

oar_circ_0001147 chr16 63867742 63877087 + 9345 9209 ENSOARG00000014361; exon_11:63876882-63877087,intron_10:63867878-63876882,; LDM_E 2 111 SEMA5A

oar_circ_0001148 chr16 64010345 64013918 + 3573 3573 ENSOARG00000014361; exon_17:64010345-64010571,intron_17:64010571-64013699,exon_18:64013699-64013918,; LDM_E 3 43 SEMA5A

oar_circ_0001149 chr16 65299098 65301617 - 2519 2264 ENSOARG00000014702; intron_4:65299206-65301470,; LDM_A 4 57 ADCY2

oar_circ_0001150 chr16 65419349 65431138 - 11789 11400 ENSOARG00000014702; intron_19:65419477-65425329,intron_20:65425441-65430989,; LDM_A 2 255 ADCY2

oar_circ_0001151 chr16 66315774 66322142 - 6368 5839 ENSOARG00000014904; intron_11:66321294-66322018,exon_6:66315774-66315988,intron_7:66315988-66318090,intron_10:66320411-66321247,intron_9:66319390-66320290,intron_8:66318219-66319282,; LDM_E 2 265 -

oar_circ_0001152 chr16 66318090 66322142 - 4052 3523 ENSOARG00000014904; intron_8:66318219-66319282,intron_9:66319390-66320290,intron_10:66320411-66321247,intron_11:66321294-66322018,; LDM_E 7 152 -

oar_circ_0001153 chr16 6732928 6736996 + 4068 2267 ENSOARG00000004394; intron_4:6734729-6736824,exon_5:6736824-6736996,; LDM_E,LDM_A 6,2 17,3 FAM169A

oar_circ_0001154 chr16 67784926 67785714 - 788 664 ENSOARG00000015446; exon_15:67784926-67785171,intron_9:67785171-67785590,; LDM_E 2 48 ADAMTS16

oar_circ_0001155 chr16 67824098 67836364 - 12266 12076 ENSOARG00000015446; exon_3:67836084-67836364,intron_17:67824204-67825845,intron_20:67830637-67836084,exon_4:67830437-67830637,exon_6:67825845-67826005,intron_18:67826005-67828446,intron_19:67828530-67830437,; LDM_E 2 428 ADAMTS16

oar_circ_0001156 chr16 6800246 6804506 + 4260 3893 ENSOARG00000004491; intron_3:6800331-6801695,intron_5:6802306-6804380,intron_4:6801753-6802208,; LDM_E 3 155 GFM2

oar_circ_0001157 chr16 6805248 6812240 + 6992 6487 ENSOARG00000004491; intron_8:6810062-6811733,exon_10:6812060-6812240,intron_7:6805337-6809973,; LDM_A 2 58 GFM2

oar_circ_0001158 chr16 6809973 6812240 + 2267 1851 ENSOARG00000004491; exon_10:6812060-6812240,intron_8:6810062-6811733,; LDM_E,LDM_A 11,6 61,12 GFM2

oar_circ_0001159 chr16 6809973 6812245 + 2272 1851 ENSOARG00000004491; exon_10:6812060-6812240,intron_8:6810062-6811733,; LDM_E,LDM_A 4,7 61,12 GFM2

oar_circ_0001160 chr16 6809973 6820348 + 10375 9399 ENSOARG00000004491; exon_15:6820158-6820348,intron_8:6810062-6811733,intron_11:6816100-6817395,intron_14:6819439-6820158,intron_12:6817544-6819109,intron_10:6812240-6816019,exon_10:6812060-6812240,; LDM_A 2 49 GFM2

oar_circ_0001161 chr16 70797798 70801432 + 3634 3228 ENSOARG00000015903; intron_3:70798934-70801323,intron_2:70797926-70798765,; LDM_E 2 143 CEP72

oar_circ_0001162 chr16 70933022 70967025 - 34003 15672 ENSOARG00000016113; intron_16:70965028-70966345,exon_12:70960547-70961020,intron_3:70954057-70954564,exon_3:70966783-70967025,intron_1:70947611-70953505,intron_6:70955681-70959831,exon_14:70959831-70960000,intron_13:70964116-70964587,intron_12:70963493-70964047,intron_4:70954797-70955314,intron_9:70961020-70962398,; LDM_E,LDM_A 18,14 137,80 SLC12A7

oar_circ_0001163 chr16 70953505 70963402 - 9897 7194 ENSOARG00000016113; intron_9:70961020-70962398,exon_12:70960547-70961020,intron_4:70954797-70955314,intron_3:70954057-70954564,intron_6:70955681-70959831,exon_14:70959831-70960000,; LDM_E,LDM_A 6,8 53,35 SLC12A7

oar_circ_0001164 chr16 71353617 71353852 - 235 235 -- -- LDM_A 3 0 --

oar_circ_0001165 chr16 8577597 8586120 - 8523 8148 ENSOARG00000005004; intron_5:8577730-8578091,exon_21:8578091-8578247,intron_7:8581719-8585990,intron_6:8578247-8581607,; LDM_E,LDM_A 2,7 888,322 FCHO2

oar_circ_0001166 chr16 8627055 8645956 - 18901 18293 ENSOARG00000005004; intron_19:8627152-8627735,intron_21:8635182-8640418,intron_23:8640803-8645881,exon_5:8640418-8640571,intron_20:8627834-8635077,; LDM_E 2 681 FCHO2

oar_circ_0001167 chr16 8734080 8738334 - 4254 3883 ENSOARG00000005038; intron_4:8734180-8735060,intron_6:8738041-8738235,intron_5:8735136-8737945,; LDM_A 2 218 TNPO1

oar_circ_0001168 chr16 8740811 8741379 - 568 159 ENSOARG00000005038; exon_17:8740981-8741140,; LDM_E,LDM_A 10,4 15,6 TNPO1

oar_circ_0001169 chr17 10038205 10046342 - 8137 7805 ENSOARG00000007284; intron_14:10038300-10041009,intron_15:10041116-10046212,; LDM_A 2 129 ARHGAP10

oar_circ_0001170 chr17 10277382 10283902 + 6520 6520 ENSOARG00000007667; exon_8:10280023-10280207,intron_8:10280207-10283184,exon_7:10277382-10277575,intron_7:10277575-10280023,exon_9:10283184-10283902,; LDM_E 6 244 PRMT9

oar_circ_0001171 chr17 10283184 10283902 + 718 718 ENSOARG00000007667; exon_9:10283184-10283902,; LDM_E 2 163 PRMT9

oar_circ_0001172 chr17 11530159 11540699 + 10540 10327 ENSOARG00000008411; intron_3:11530296-11540623,; LDM_A 2 5 SLC10A7

oar_circ_0001173 chr17 11762436 11763658 + 1222 1222 -- -- LDM_A 2 0 --

oar_circ_0001174 chr17 12188651 12218594 + 29943 29888 ENSOARG00000008579; exon_7:12215856-12216096,intron_7:12216096-12218539,intron_6:12188885-12215856,exon_6:12188651-12188885,; LDM_E 5 589 ZNF827

oar_circ_0001175 chr17 12510092 12510473 - 381 381 ENSOARG00000009089; intron_6:12509366-12543963,; LDM_E 2 34 SMAD1

oar_circ_0001176 chr17 12874396 12876985 + 2589 2409 ENSOARG00000009303; intron_13:12874559-12876503,exon_13:12874396-12874559,intron_14:12876627-12876929,; LDM_E 4 133 OTUD4

oar_circ_0001177 chr17 14047245 14047701 - 456 456 -- -- LDM_E,LDM_A 23,11 1,0 --

oar_circ_0001178 chr17 14050604 14063368 - 12764 11922 ENSOARG00000010589; exon_6:14062319-14062499,exon_13:14051943-14052096,intron_19:14061049-14062319,intron_15:14054069-14055770,intron_16:14055880-14058579,exon_7:14060893-14061049,intron_14:14052295-14053842,exon_11:14053842-14054069,intron_12:14050737-14051943,intron_20:14062499-14063267,intron_17:14058648-14060663,; LDM_E,LDM_A 2,2 772,485 SMARCA5

oar_circ_0001179 chr17 14062319 14065201 - 2882 2680 ENSOARG00000010589; intron_21:14063368-14065100,exon_6:14062319-14062499,intron_20:14062499-14063267,; LDM_E 2 116 SMARCA5

oar_circ_0001180 chr17 14112245 14126987 - 14742 14438 ENSOARG00000010978; exon_4:14126385-14126987,intron_4:14113702-14124380,intron_7:14124961-14126385,exon_6:14124380-14124684,intron_6:14124684-14124875,intron_3:14112369-14113608,; LDM_A 2 360 GAB1

oar_circ_0001181 chr17 14142161 14142456 - 295 295 ENSOARG00000010978; exon_2:14142161-14142456,; LDM_A 3 26 GAB1

oar_circ_0001182 chr17 14369647 14394453 - 24806 24204 ENSOARG00000011407; exon_10:14379079-14379273,intron_11:14390421-14393549,intron_10:14388999-14390393,intron_4:14376523-14379079,exon_9:14381138-14381297,intron_7:14385673-14387646,intron_9:14387720-14388975,intron_12:14393582-14394317,exon_13:14369647-14371004,intron_6:14381297-14385567,intron_5:14379273-14381138,intron_2:14371004-14371912,intron_3:14372019-14376429,; LDM_A 5 1066 USP38

oar_circ_0001183 chr17 15153729 15156042 + 2313 2077 ENSOARG00000011798; intron_4:15153848-15155925,; LDM_E 3 82 INPP4B

oar_circ_0001184 chr17 15287584 15313331 + 25747 25414 ENSOARG00000011798; intron_9:15296292-15313183,intron_8:15287696-15296219,; LDM_E 3 854 INPP4B

oar_circ_0001185 chr17 15351044 15351632 + 588 352 ENSOARG00000011798; intron_11:15351175-15351527,; LDM_A 2 3 INPP4B

oar_circ_0001186 chr17 15452430 15463296 + 10866 10385 ENSOARG00000011798; intron_20:15461492-15463198,intron_17:15452603-15453832,intron_18:15453956-15455022,exon_17:15452430-15452603,intron_19:15455140-15461351,; LDM_E,LDM_A 3,4 436,158 INPP4B

oar_circ_0001187 chr17 15461351 15495136 + 33785 33433 ENSOARG00000011798; intron_21:15463296-15491454,exon_23:15494981-15495136,intron_20:15461492-15463198,intron_22:15491567-15494981,; LDM_E 11 803 INPP4B

oar_circ_0001188 chr17 1576383 1580079 + 3696 161 ENSOARG00000016252; exon_15:1576383-1576544,; LDM_E 2 1 TLL1

oar_circ_0001189 chr17 16225495 16240101 - 14606 13785 ENSOARG00000012172; intron_3:16228587-16232459,intron_8:16238332-16239955,intron_2:16225613-16228540,intron_6:16236112-16238134,intron_4:16232564-16233200,intron_5:16233327-16236032,; LDM_A 2 116 ZNF330

oar_circ_0001190 chr17 16228540 16233327 - 4787 4508 ENSOARG00000012172; intron_4:16232564-16233200,intron_3:16228587-16232459,; LDM_E,LDM_A 7,2 109,21 ZNF330

oar_circ_0001191 chr17 16484355 16495148 + 10793 10793 ENSOARG00000012236; exon_2:16494897-16495148,intron_1:16328316-16494897,; LDM_A 3 109 RNF150

oar_circ_0001192 chr17 16494897 16516700 + 21803 21648 ENSOARG00000012236; intron_2:16495148-16514383,exon_2:16494897-16495148,intron_3:16514455-16516617,; LDM_A 7 293 RNF150

oar_circ_0001193 chr17 16514383 16516700 + 2317 2162 ENSOARG00000012236; intron_3:16514455-16516617,; LDM_A 8 29 RNF150

oar_circ_0001194 chr17 16789230 16790177 + 947 947 ENSOARG00000012380; exon_8:16790026-16790177,exon_7:16789230-16789401,intron_7:16789401-16790026,; LDM_A 2 4 TBC1D9

oar_circ_0001195 chr17 16991708 16995290 + 3582 3072 ENSOARG00000012717; exon_10:16993701-16993917,intron_9:16992092-16993701,intron_10:16993917-16995164,; LDM_E 2 19 CLGN

oar_circ_0001196 chr17 1719808 1746640 + 26832 25774 ENSOARG00000016382; intron_6:1737783-1739518,intron_4:1730001-1735753,intron_9:1742076-1745906,exon_3:1721814-1722132,intron_7:1739589-1740843,exon_6:1737544-1737783,intron_2:1720768-1721814,intron_1:1719954-1720683,intron_3:1722132-1729771,intron_5:1735847-1737544,intron_8:1741012-1741986,exon_4:1729771-1730001,exon_10:1745906-1746068,exon_8:1740843-1741012,; LDM_E 24 547 MAP9

oar_circ_0001197 chr17 1729771 1746640 + 16869 16042 ENSOARG00000016382; intron_5:1735847-1737544,intron_8:1741012-1741986,intron_6:1737783-1739518,intron_4:1730001-1735753,intron_9:1742076-1745906,intron_7:1739589-1740843,exon_6:1737544-1737783,exon_10:1745906-1746068,exon_8:1740843-1741012,exon_4:1729771-1730001,; LDM_E 4 305 MAP9

oar_circ_0001198 chr17 17840468 17855405 + 14937 14807 ENSOARG00000013062; intron_2:17840598-17855203,exon_3:17855203-17855405,; LDM_E 2 593 SETD7

oar_circ_0001199 chr17 17840468 17860245 + 19777 19647 ENSOARG00000013062; intron_2:17840598-17855203,exon_3:17855203-17855405,exon_4:17860055-17860245,intron_3:17855405-17860055,; LDM_E,LDM_A 11,15 684,955 SETD7

oar_circ_0001200 chr17 17855203 17860245 + 5042 5042 ENSOARG00000013062; intron_3:17855405-17860055,exon_4:17860055-17860245,exon_3:17855203-17855405,; LDM_E,LDM_A 4,8 137,237 SETD7

oar_circ_0001201 chr17 17855203 17870574 + 15371 15171 ENSOARG00000013062; exon_4:17860055-17860245,intron_3:17855405-17860055,intron_5:17866224-17868663,intron_4:17860245-17866142,intron_6:17868781-17870416,exon_3:17855203-17855405,exon_7:17870416-17870574,; LDM_E 2 298 SETD7

oar_circ_0001202 chr17 18116010 18123520 - 7510 7510 -- -- LDM_A 2 0 --

oar_circ_0001203 chr17 18156330 18157937 + 1607 1519 ENSOARG00000014035; intron_2:18156418-18157698,exon_3:18157698-18157937,; LDM_A 5 141 ELF2

oar_circ_0001204 chr17 18156330 18175228 + 18898 18810 ENSOARG00000014035; intron_3:18157937-18175062,exon_3:18157698-18157937,intron_2:18156418-18157698,exon_4:18175062-18175228,; LDM_E,LDM_A 32,19 1303,828 ELF2

oar_circ_0001205 chr17 18156330 18182385 + 26055 26055 ENSOARG00000014035; exon_4:18175062-18175228,intron_4:18175228-18216339,intron_2:18156418-18157698,exon_3:18157698-18157937,intron_3:18157937-18175062,; LDM_E,LDM_A 29,25 1811,1235 ELF2

oar_circ_0001206 chr17 18217976 18223423 + 5447 5360 ENSOARG00000014035; exon_9:18223230-18223423,intron_8:18220920-18223230,intron_6:18218150-18220833,exon_6:18217976-18218150,; LDM_E,LDM_A 3,3 351,219 ELF2

oar_circ_0001207 chr17 28604748 28611490 + 6742 6643 ENSOARG00000014865; intron_12:28604926-28611391,exon_12:28604748-28604926,; LDM_A 2 3 SCLT1

oar_circ_0001208 chr17 28744238 28748927 - 4689 4572 ENSOARG00000015126; exon_5:28748715-28748927,intron_4:28744355-28745571,intron_5:28745739-28748715,exon_6:28745571-28745739,; LDM_A 3 57 JADE1

oar_circ_0001209 chr17 29409844 29417804 - 7960 7338 ENSOARG00000015300; intron_20:29415340-29417323,intron_19:29413978-29415162,exon_4:29415162-29415340,exon_7:29409844-29410010,intron_18:29410372-29413837,intron_21:29417383-29417745,; LDM_A 5 79 LARP1B

oar_circ_0001210 chr17 29415162 29417804 - 2642 2523 ENSOARG00000015300; exon_4:29415162-29415340,intron_20:29415340-29417323,intron_21:29417383-29417745,; LDM_E,LDM_A 10,31 41,49 LARP1B

oar_circ_0001211 chr17 29461637 29462452 - 815 623 ENSOARG00000015500; intron_4:29461739-29462362,; LDM_A 6 46 -

oar_circ_0001212 chr17 29701610 29709319 - 7709 7500 ENSOARG00000016297; exon_4:29709115-29709319,intron_11:29701700-29704924,intron_12:29705043-29709115,; LDM_E 2 65 INTU

oar_circ_0001213 chr17 32075228 32085525 - 10297 10297 ENSOARG00000016689; exon_6:32083381-32083732,intron_13:32076959-32083381,intron_12:32075403-32076036,intron_14:32083732-32085263,exon_5:32085263-32085525,exon_8:32075228-32075403,exon_7:32076036-32076959,; LDM_E 2 941 FAT4

oar_circ_0001214 chr17 32813774 32820603 + 6829 6829 ENSOARG00000016961; exon_3:32817052-32820603,intron_2:32814004-32817052,exon_2:32813774-32814004,; LDM_E 2 3650 ANKRD50

oar_circ_0001215 chr17 34444053 34452571 - 8518 7261 ENSOARG00000017222; intron_11:34445470-34446561,exon_6:34446561-34447206,intron_9:34444177-34445015,intron_13:34447884-34452413,exon_4:34452413-34452571,; LDM_E 4 175 SPATA5

oar_circ_0001216 chr17 34464554 34464705 + 151 151 -- -- LDM_A 4 0 --

oar_circ_0001217 chr17 346457 359124 + 12667 12172 ENSOARG00000016002; intron_9:353362-359006,intron_7:346567-351538,intron_8:351655-353212,; LDM_E 2 545 KLHL2

oar_circ_0001218 chr17 35041640 35042538 - 898 786 ENSOARG00000000316; intron_7:35041858-35042075,exon_66:35042380-35042538,exon_68:35041640-35041858,exon_67:35042075-35042268,; LDM_E,LDM_A 11,2 77,25 KIAA1109

oar_circ_0001219 chr17 35102004 35118157 - 16153 15859 ENSOARG00000000316; exon_34:35105614-35105790,exon_33:35108672-35108848,intron_46:35115375-35117073,intron_43:35110755-35111220,exon_35:35102680-35103387,exon_29:35115134-35115375,intron_42:35108848-35110596,intron_48:35117547-35118005,exon_26:35118005-35118157,intron_40:35103387-35105614,exon_36:35102004-35102244,exon_31:35111220-35111440,exon_28:35117073-35117226,intron_47:35117226-35117396,intron_44:35111440-35113857,intron_41:35105790-35108672,exon_32:35110596-35110755,intron_45:35114000-35115134,intron_39:35102244-35102680,; LDM_E 7 773 KIAA1109

oar_circ_0001220 chr17 35105614 35108848 - 3234 3234 ENSOARG00000000316; exon_33:35108672-35108848,exon_34:35105614-35105790,intron_41:35105790-35108672,; LDM_E 2 118 KIAA1109

oar_circ_0001221 chr17 35110596 35111440 - 844 844 ENSOARG00000000316; exon_31:35111220-35111440,exon_32:35110596-35110755,intron_43:35110755-35111220,; LDM_E 5 62 KIAA1109

oar_circ_0001222 chr17 35110596 35118157 - 7561 7097 ENSOARG00000000316; exon_26:35118005-35118157,exon_28:35117073-35117226,intron_46:35115375-35117073,intron_48:35117547-35118005,intron_43:35110755-35111220,exon_31:35111220-35111440,intron_45:35114000-35115134,exon_32:35110596-35110755,exon_29:35115134-35115375,intron_44:35111440-35113857,; LDM_A 2 94 KIAA1109

oar_circ_0001223 chr17 35163290 35164942 - 1652 1378 ENSOARG00000000316; exon_5:35164763-35164942,intron_68:35163496-35164489,exon_7:35163290-35163496,; LDM_E,LDM_A 5,12 70,26 KIAA1109

oar_circ_0001224 chr17 39421270 39424547 - 3277 3277 ENSOARG00000002302; exon_20:39421270-39421654,intron_12:39423773-39424389,exon_19:39423532-39423773,exon_18:39424389-39424547,intron_11:39421654-39423532,; LDM_E 5 237 RAPGEF2

oar_circ_0001225 chr17 39523184 39525287 - 2103 2027 ENSOARG00000002302; intron_25:39523352-39525211,exon_6:39523184-39523352,; LDM_A 4 122 RAPGEF2

oar_circ_0001226 chr17 3952710 3959040 - 6330 5909 ENSOARG00000002381; intron_32:3952821-3953651,intron_34:3954882-3958971,intron_33:3953768-3954758,; LDM_E,LDM_A 13,8 395,123 KIAA0922

oar_circ_0001227 chr17 4206337 4207136 - 799 695 ENSOARG00000003318; intron_5:4206505-4207032,exon_8:4206337-4206505,; LDM_A 5 7 TRIM2

oar_circ_0001228 chr17 43037474 43041243 + 3769 2972 ENSOARG00000006748; intron_5:43038271-43041075,exon_6:43041075-43041243,; LDM_E,LDM_A 6,2 40,8 CTSO

oar_circ_0001229 chr17 43282171 43289560 - 7389 7327 ENSOARG00000008232; intron_7:43282233-43289168,exon_2:43289168-43289560,; LDM_A 2 140 GUCY1A3

oar_circ_0001230 chr17 43690209 43693692 + 3483 3267 ENSOARG00000008472; intron_2:43692203-43693499,intron_1:43690322-43692100,exon_3:43693499-43693692,; LDM_E 3 313 -

oar_circ_0001231 chr17 43690209 43709179 + 18970 18627 ENSOARG00000008472; exon_3:43693499-43693692,intron_1:43690322-43692100,intron_3:43693692-43709052,intron_2:43692203-43693499,; LDM_E 3 585 -

oar_circ_0001232 chr17 43713428 43713596 - 168 168 -- -- LDM_E 2 0 --

oar_circ_0001233 chr17 44658455 44661058 - 2603 2198 ENSOARG00000012480; intron_28:44658860-44660861,exon_32:44660861-44661058,; LDM_E,LDM_A 2,4 33,26 EP400

oar_circ_0001234 chr17 4629788 4663931 - 34143 33921 ENSOARG00000003643; intron_4:4641762-4648176,exon_6:4641604-4641762,intron_6:4654728-4663822,exon_7:4629788-4629963,exon_5:4648176-4648398,intron_3:4629963-4641604,intron_5:4648398-4654615,; LDM_E 6 965 ARFIP1

oar_circ_0001235 chr17 47927661 47933588 + 5927 5849 ENSOARG00000016772; exon_6:47933419-47933588,intron_5:47932759-47933419,intron_4:47932260-47932681,exon_3:47927661-47927957,exon_4:47932091-47932260,intron_3:47927957-47932091,; LDM_E,LDM_A 2,5 337,169 SLC15A4

oar_circ_0001236 chr17 48309053 48309942 - 889 889 ENSOARG00000016899; exon_1:48309053-48309979,; LDM_E 4 92 TMEM132C

oar_circ_0001237 chr17 48975779 48978573 - 2794 2794 -- -- LDM_A 2 0 --

oar_circ_0001238 chr17 50485250 50485478 + 228 228 ENSOARG00000018177; exon_1:50485202-50485799,; LDM_A 3 922 UBC

oar_circ_0001239 chr17 50485250 50487273 + 2023 2023 ENSOARG00000018177; intron_2:50485869-50486165,exon_4:50486516-50487474,exon_1:50485202-50485799,intron_3:50486182-50486516,; LDM_A 3 15920 UBC

oar_circ_0001240 chr17 50485406 50485634 - 228 228 ENSOARG00000017707; intron_2:50421784-50604149,; LDM_E,LDM_A 6,18 533,1753 BRI3BP

oar_circ_0001241 chr17 50485406 50486745 - 1339 1339 ENSOARG00000017707; intron_2:50421784-50604149,; LDM_E,LDM_A 7,24 2273,7746 BRI3BP

oar_circ_0001242 chr17 50485406 50486973 - 1567 1567 ENSOARG00000017707; intron_2:50421784-50604149,; LDM_E,LDM_A 3,14 3417,12861 BRI3BP

oar_circ_0001243 chr17 50485406 50487201 - 1795 1795 ENSOARG00000017707; intron_2:50421784-50604149,; LDM_E,LDM_A 6,15 6085,23003 BRI3BP

oar_circ_0001244 chr17 50485425 50485653 + 228 228 ENSOARG00000018177; exon_1:50485202-50485799,; LDM_E,LDM_A 6,16 247,1066 UBC

oar_circ_0001245 chr17 50594842 50606607 + 11765 11373 ENSOARG00000018396; intron_10:50605599-50606460,intron_9:50600796-50605547,intron_8:50594961-50600722,; LDM_A 2 40 SCARB1

oar_circ_0001246 chr17 5094737 5095993 + 1256 1256 ENSOARG00000004146; exon_2:5095429-5095993,intron_1:4965731-5095429,; LDM_E 2 127 FBXW7

oar_circ_0001247 chr17 50950072 50952935 + 2863 2863 ENSOARG00000000357; intron_11:50949872-50952966,; LDM_E 2 87 NCOR2

oar_circ_0001248 chr17 51494773 51501628 - 6855 1178 ENSOARG00000002205; intron_66:51497806-51498984,; LDM_E 2 1 DNAH10

oar_circ_0001249 chr17 51553417 51557630 - 4213 3643 ENSOARG00000002788; intron_9:51557005-51557493,exon_15:51554224-51554435,intron_6:51554435-51555207,intron_7:51555326-51556623,exon_12:51556817-51557005,intron_5:51553537-51554224,; LDM_E 3 54 ATP6V0A2

oar_circ_0001250 chr17 51590635 51603904 - 13269 12473 ENSOARG00000002876; intron_8:51603525-51603769,intron_4:51591698-51594950,intron_7:51599671-51603447,intron_5:51595057-51598579,intron_3:51590761-51591541,intron_6:51598691-51599590,; LDM_E 4 174 TCTN2

oar_circ_0001251 chr17 51598579 51603904 - 5325 4919 ENSOARG00000002876; intron_6:51598691-51599590,intron_8:51603525-51603769,intron_7:51599671-51603447,; LDM_E 2 104 TCTN2

oar_circ_0001252 chr17 51631462 51632167 - 705 431 ENSOARG00000003043; intron_10:51631629-51632060,; LDM_A 6 3 GTF2H3

oar_circ_0001253 chr17 51721650 51722653 + 1003 884 ENSOARG00000003562; intron_3:51721769-51722431,exon_4:51722431-51722653,; LDM_E,LDM_A 16,52 15,56 RILPL1

oar_circ_0001254 chr17 51840482 51843228 + 2746 2509 ENSOARG00000004404; exon_3:51842918-51843228,intron_1:51840614-51841170,intron_2:51841275-51842918,; LDM_E 2 129 SBNO1

oar_circ_0001255 chr17 51846893 51866523 + 19630 18106 ENSOARG00000004404; intron_6:51852214-51855332,exon_18:51865739-51865915,intron_8:51855744-51857509,exon_13:51859686-51859869,intron_13:51859869-51860610,exon_17:51865473-51865641,intron_4:51846994-51850564,intron_7:51855466-51855653,intron_5:51850661-51852053,intron_16:51865098-51865473,intron_15:51862480-51864900,exon_9:51857509-51857671,intron_18:51865915-51866391,intron_12:51859267-51859686,exon_16:51864900-51865098,intron_9:51857671-51858652,exon_6:51852053-51852214,intron_14:51860708-51862322,; LDM_A 2 352 SBNO1

oar_circ_0001256 chr17 51862328 51866523 + 4195 3971 ENSOARG00000004404; intron_15:51862480-51864900,intron_16:51865098-51865473,exon_17:51865473-51865641,exon_15:51862322-51862480,intron_18:51865915-51866391,exon_18:51865739-51865915,exon_16:51864900-51865098,; LDM_E 3 179 SBNO1

oar_circ_0001257 chr17 51864900 51866523 + 1623 1393 ENSOARG00000004404; exon_16:51864900-51865098,exon_18:51865739-51865915,intron_18:51865915-51866391,intron_16:51865098-51865473,exon_17:51865473-51865641,; LDM_E 2 53 SBNO1

oar_circ_0001258 chr17 51941610 51947424 + 5814 5492 ENSOARG00000005540; intron_6:51945681-51947317,intron_5:51941734-51945590,; LDM_A 2 67 MPHOSPH9

oar_circ_0001259 chr17 52116135 52117521 + 1386 1264 ENSOARG00000006283; intron_4:52116350-52117399,exon_4:52116135-52116350,; LDM_E,LDM_A 46,14 39,15 PITPNM2

oar_circ_0001260 chr17 52120540 52131188 + 10648 10361 ENSOARG00000006283; exon_7:52123347-52123656,intron_11:52130289-52130964,intron_10:52127993-52130274,exon_6:52120540-52120768,intron_6:52120768-52123347,intron_9:52127751-52127922,intron_7:52123656-52124812,intron_8:52124908-52127646,exon_12:52130964-52131188,; LDM_E 2 120 PITPNM2

oar_circ_0001261 chr17 52168164 52168860 + 696 613 ENSOARG00000007293; exon_1:52168247-52168860,; LDM_A 2 7 ABCB9

oar_circ_0001262 chr17 52303382 52306701 - 3319 3196 ENSOARG00000008130; intron_3:52303505-52306544,exon_10:52306544-52306701,; LDM_E,LDM_A 3,2 18,6 CCDC62

oar_circ_0001263 chr17 52303382 52323934 - 20552 20020 ENSOARG00000008130; intron_9:52318911-52320636,exon_5:52318739-52318911,intron_6:52311881-52312224,intron_8:52316822-52318739,intron_7:52312313-52316720,intron_4:52306701-52309255,intron_5:52309981-52311765,exon_9:52309255-52309981,exon_3:52323767-52323934,exon_10:52306544-52306701,intron_10:52320738-52323767,intron_3:52303505-52306544,; LDM_E 4 631 CCDC62

oar_circ_0001264 chr17 52517041 52521603 + 4562 4518 ENSOARG00000010564; intron_2:52517198-52518265,exon_4:52520744-52520935,intron_4:52520935-52521399,exon_5:52521399-52521603,intron_3:52518309-52520744,exon_2:52517041-52517198,; LDM_A 4 480 RSRC2

oar_circ_0001265 chr17 52539598 52547683 + 8085 7190 ENSOARG00000010728; intron_9:52546633-52547540,intron_7:52545399-52545954,intron_5:52542924-52545116,intron_8:52546024-52546490,intron_4:52541695-52542849,intron_3:52539673-52541589,; LDM_E,LDM_A 15,4 493,268 ZCCHC8

oar_circ_0001266 chr17 52541589 52550497 + 8908 7966 ENSOARG00000010728; intron_10:52547683-52550375,intron_4:52541695-52542849,intron_5:52542924-52545116,intron_9:52546633-52547540,intron_7:52545399-52545954,intron_8:52546024-52546490,; LDM_E,LDM_A 8,7 448,259 ZCCHC8

oar_circ_0001267 chr17 52698209 52698680 - 471 471 -- -- LDM_A 2 0 --

oar_circ_0001268 chr17 52727773 52730173 + 2400 2067 ENSOARG00000011538; intron_9:52729179-52730035,intron_8:52727900-52729111,; LDM_A 3 10 -

oar_circ_0001269 chr17 52730035 52737344 + 7309 7033 ENSOARG00000011538; exon_12:52737175-52737344,intron_11:52733315-52737175,intron_10:52730173-52733177,; LDM_E 10 70 -

oar_circ_0001270 chr17 53122172 53131357 - 9185 8703 ENSOARG00000013181; intron_9:53129260-53131238,intron_7:53125376-53125974,intron_6:53122262-53125280,intron_8:53126034-53129143,; LDM_E 3 573 TMEM120B

oar_circ_0001271 chr17 53343581 53343840 + 259 259 -- -- LDM_E,LDM_A 3,3 0,0 --

oar_circ_0001272 chr17 53679085 53680610 + 1525 1429 ENSOARG00000015713; intron_8:53679249-53680514,exon_8:53679085-53679249,; LDM_E 3 7 IFT81

oar_circ_0001273 chr17 53872274 53890100 - 17826 13645 ENSOARG00000016608; intron_5:53881517-53883096,exon_11:53875545-53875735,intron_6:53883239-53883903,exon_2:53889913-53890100,intron_8:53884441-53887562,intron_3:53878409-53880617,exon_9:53878184-53878409,exon_8:53880617-53880814,intron_1:53875735-53877397,intron_2:53877548-53878184,intron_4:53880814-53881399,intron_9:53887682-53889913,exon_5:53883903-53884063,; LDM_E,LDM_A 2,2 1228,547 ANAPC7

oar_circ_0001274 chr17 53877397 53890100 - 12703 12059 ENSOARG00000016608; exon_9:53878184-53878409,intron_8:53884441-53887562,intron_7:53884063-53884329,intron_6:53883239-53883903,exon_8:53880617-53880814,intron_4:53880814-53881399,intron_5:53881517-53883096,intron_9:53887682-53889913,intron_3:53878409-53880617,intron_2:53877548-53878184,exon_2:53889913-53890100,exon_5:53883903-53884063,; LDM_E 2 1013 ANAPC7

oar_circ_0001275 chr17 546381 558154 + 11773 11655 ENSOARG00000016153; exon_2:546381-546578,intron_2:546578-548249,intron_3:548417-558036,exon_3:548249-548417,; LDM_E 2 302 CPE

oar_circ_0001276 chr17 54764338 54765229 - 891 745 ENSOARG00000018223; exon_22:54764338-54764566,intron_3:54764566-54765083,; LDM_A 10 12 ATXN2

oar_circ_0001277 chr17 54829141 54831592 - 2451 2282 ENSOARG00000018223; intron_23:54830462-54831555,intron_22:54829925-54830402,exon_4:54829141-54829292,intron_21:54829292-54829853,; LDM_E,LDM_A 32,19 263,51 ATXN2

oar_circ_0001278 chr17 54938086 54973063 + 34977 17169 ENSOARG00000018556;ENSOARG00000018772; intron_15:54943614-54944721,intron_17:54945323-54946057,exon_24:54953231-54953404,exon_19:54946577-54947069,intron_25:54955155-54956131,intron_14:54943198-54943548,intron_13:54942247-54943085,intron_18:54946080-54946577,intron_23:54952557-54953231,intron_20:54950627-54951113,intron_10:54939322-54939835,intron_11:54939871-54941258,intron_24:54953404-54955011,intron_12:54941453-54942216,intron_26:54956209-54956800,intron_8:54938228-54939211,intron_22:54951467-54952398,intron_19:54947069-54950486,intron_16:54944793-54945243,;exon_1:54972863-54973063,; LDM_E 7 471 -;-

oar_circ_0001279 chr17 54950486 55006733 + 56247 38802 ENSOARG00000018772;ENSOARG00000018556; intron_11:54998178-55006344,intron_8:54991177-54995645,intron_9:54995796-54997100,intron_3:54979130-54982968,intron_4:54983127-54983845,intron_5:54984005-54985451,intron_6:54985593-54989506,exon_4:54982968-54983127,exon_3:54978935-54979130,exon_12:55006344-55006733,intron_7:54989575-54990995,intron_2:54977566-54978935,exon_5:54983845-54984005,exon_1:54972863-54973063,exon_8:54990995-54991177,exon_9:54995645-54995796,intron_1:54973063-54977417,intron_10:54997246-54998004,exon_11:54998004-54998178,;intron_25:54955155-54956131,exon_24:54953231-54953404,intron_24:54953404-54955011,intron_22:54951467-54952398,intron_23:54952557-54953231,intron_26:54956209-54956800,intron_20:54950627-54951113,; LDM_E 6 638 -;-

oar_circ_0001280 chr17 54951376 55011123 + 59747 42254 ENSOARG00000018772;ENSOARG00000018556; intron_3:54979130-54982968,exon_4:54982968-54983127,exon_12:55006344-55006733,intron_11:54998178-55006344,intron_1:54973063-54977417,exon_3:54978935-54979130,intron_6:54985593-54989506,intron_9:54995796-54997100,exon_9:54995645-54995796,intron_12:55006733-55010355,exon_11:54998004-54998178,exon_5:54983845-54984005,intron_13:55010496-55010985,exon_1:54972863-54973063,intron_4:54983127-54983845,intron_8:54991177-54995645,exon_8:54990995-54991177,intron_7:54989575-54990995,intron_10:54997246-54998004,intron_5:54984005-54985451,intron_2:54977566-54978935,;intron_22:54951467-54952398,intron_25:54955155-54956131,intron_26:54956209-54956800,intron_24:54953404-54955011,intron_23:54952557-54953231,; LDM_A 6 375 -;-

oar_circ_0001281 chr17 55148933 55152452 + 3519 3417 ENSOARG00000000627; exon_3:55148933-55149109,intron_3:55149109-55152350,; LDM_E 2 26 CIT

oar_circ_0001282 chr17 55167011 55179526 + 12515 12124 ENSOARG00000000627; intron_6:55168135-55177697,intron_7:55177901-55179372,exon_7:55177697-55177901,intron_5:55167154-55168041,; LDM_E 4 116 CIT

oar_circ_0001283 chr17 55177697 55179526 + 1829 1675 ENSOARG00000000627; exon_7:55177697-55177901,intron_7:55177901-55179372,; LDM_E 2 25 CIT

oar_circ_0001284 chr17 56436265 56438821 - 2556 2122 ENSOARG00000002352; intron_5:56437759-56438725,intron_3:56436371-56437527,; LDM_E,LDM_A 11,12 71,41 SUDS3

oar_circ_0001285 chr17 56667394 56686032 + 18638 18638 -- -- LDM_E 2 0 --

oar_circ_0001286 chr17 57454486 57474412 + 19926 19381 ENSOARG00000004210; intron_25:57472777-57474290,exon_19:57459063-57459238,intron_18:57454603-57459063,exon_24:57471612-57471823,intron_21:57460724-57467474,exon_23:57469667-57469837,intron_24:57471823-57472689,exon_22:57467474-57467668,intron_19:57459238-57459989,intron_22:57467668-57469667,intron_23:57469837-57471612,intron_20:57460128-57460645,; LDM_A 15 81 NOS1

oar_circ_0001287 chr17 57509274 57517998 + 8724 8181 ENSOARG00000004822; intron_3:57509396-57512372,intron_6:57514069-57517818,intron_4:57512519-57512818,intron_5:57512955-57513932,exon_7:57517818-57517998,; LDM_A 2 92 FBXO21

oar_circ_0001288 chr17 57659643 57662714 - 3071 3071 ENSOARG00000005170; exon_6:57659643-57659850,exon_5:57662517-57662714,intron_5:57659850-57662517,; LDM_E 6 131 FBXW8

oar_circ_0001289 chr17 57689791 57706590 - 16799 16552 ENSOARG00000005170; intron_8:57702507-57706425,intron_7:57689949-57702418,exon_2:57706425-57706590,; LDM_E,LDM_A 4,2 746,299 FBXW8

oar_circ_0001290 chr17 57702418 57706590 - 4172 4083 ENSOARG00000005170; exon_2:57706425-57706590,intron_8:57702507-57706425,; LDM_A 3 98 FBXW8

oar_circ_0001291 chr17 58250938 58251176 + 238 238 -- -- LDM_E 2 0 --

oar_circ_0001292 chr17 5882028 5885585 + 3557 3358 ENSOARG00000004355; intron_6:5882142-5885500,; LDM_A 4 29 GATB

oar_circ_0001293 chr17 5939385 5940204 - 819 680 ENSOARG00000004874; intron_3:5939524-5940025,exon_13:5940025-5940204,; LDM_E,LDM_A 4,14 8,17 FAM160A1

oar_circ_0001294 chr17 5967800 5971235 - 3435 3355 ENSOARG00000004874; intron_9:5967880-5971067,exon_7:5971067-5971235,; LDM_A 2 105 FAM160A1

oar_circ_0001295 chr17 59802701 59802933 - 232 232 ENSOARG00000004691; exon_1:59802414-59803080,; LDM_E,LDM_A 2,11 42,71 RAB21

oar_circ_0001296 chr17 60954744 60956183 - 1439 1105 ENSOARG00000008875; intron_3:60954924-60956029,; LDM_A 4 5 SLC8B1

oar_circ_0001297 chr17 61424640 61427265 - 2625 2324 ENSOARG00000009565; exon_9:61424640-61424799,intron_9:61425003-61427168,; LDM_E,LDM_A 2,8 76,41 PTPN11

oar_circ_0001298 chr17 61799653 61808304 + 8651 8408 ENSOARG00000010548; intron_4:61800246-61808172,intron_3:61799700-61800182,; LDM_E 2 20 TMEM116

oar_circ_0001299 chr17 61913011 61918521 - 5510 4964 ENSOARG00000010578; intron_11:61917008-61917778,intron_12:61917860-61918450,intron_8:61913107-61913878,intron_10:61915527-61916910,intron_9:61913968-61915418,; LDM_E 2 108 MAPKAPK5

oar_circ_0001300 chr17 62670907 62672930 - 2023 1711 ENSOARG00000013244; intron_7:62671994-62672810,intron_6:62671020-62671915,; LDM_E,LDM_A 21,34 105,24 SPPL3

oar_circ_0001301 chr17 62670907 62674829 - 3922 3521 ENSOARG00000013244; intron_8:62672930-62674740,intron_6:62671020-62671915,intron_7:62671994-62672810,; LDM_E,LDM_A 3,2 191,64 SPPL3

oar_circ_0001302 chr17 63366805 63368883 + 2078 1938 ENSOARG00000015859; intron_4:63366892-63367220,intron_5:63367273-63368687,exon_6:63368687-63368883,; LDM_E,LDM_A 3,25 53,31 KCTD10

oar_circ_0001303 chr17 63528090 63528360 - 270 270 ENSOARG00000016430; exon_39:63528090-63528360,; LDM_A 2 63 ACACB

oar_circ_0001304 chr17 63528090 63529089 - 999 876 ENSOARG00000016430; exon_39:63528090-63528360,intron_15:63528360-63528966,; LDM_E,LDM_A 5,9 12,65 ACACB

oar_circ_0001305 chr17 63536234 63547284 - 11050 9894 ENSOARG00000016430; intron_20:63536450-63537756,intron_27:63546297-63546884,intron_26:63543573-63546207,intron_24:63540513-63541643,intron_21:63537798-63538036,intron_25:63541781-63543454,exon_34:63536234-63536450,intron_22:63538093-63540203,; LDM_A 2 353 ACACB

oar_circ_0001306 chr17 63540203 63541781 - 1578 1130 ENSOARG00000016430; intron_24:63540513-63541643,; LDM_A 16 54 ACACB

oar_circ_0001307 chr17 63540203 63543573 - 3370 2803 ENSOARG00000016430; intron_24:63540513-63541643,intron_25:63541781-63543454,; LDM_A 16 67 ACACB

oar_circ_0001308 chr17 63575764 63576228 - 464 191 ENSOARG00000016430; intron_50:63575903-63576094,; LDM_A 3 9 ACACB

oar_circ_0001309 chr17 63603695 63604351 - 656 656 ENSOARG00000016430; exon_1:63603695-63604351,; LDM_A 5 299 ACACB

oar_circ_0001310 chr17 64053201 64059506 + 6305 5896 ENSOARG00000018082; exon_5:64056641-64056826,intron_6:64058167-64059381,intron_5:64056826-64058115,intron_4:64054577-64056641,intron_3:64053328-64054472,; LDM_E 4 170 SART3

oar_circ_0001311 chr17 64484438 64485785 - 1347 1347 -- -- LDM_E 2 0 --

oar_circ_0001312 chr17 65218774 65219113 + 339 339 -- -- LDM_E 2 0 --

oar_circ_0001313 chr17 65234844 65235377 - 533 533 -- -- LDM_A 2 0 --

oar_circ_0001314 chr17 65279051 65292967 + 13916 13596 ENSOARG00000019120; intron_19:65284387-65286439,intron_20:65286587-65292807,exon_18:65280991-65281184,exon_21:65292807-65292967,intron_18:65281184-65284306,intron_17:65279142-65280991,; LDM_E 2 310 MYO18B

oar_circ_0001315 chr17 65333291 65333794 + 503 274 ENSOARG00000019120; intron_29:65333411-65333685,; LDM_A 4 8 MYO18B

oar_circ_0001316 chr17 65363158 65388229 + 25071 22980 ENSOARG00000019120; intron_37:65363311-65379347,exon_37:65363158-65363311,intron_39:65381001-65381765,intron_41:65383502-65384973,intron_38:65379461-65380884,exon_43:65388043-65388229,intron_42:65385096-65388043,; LDM_E 4 468 MYO18B

oar_circ_0001317 chr17 67023244 67026535 - 3291 3032 ENSOARG00000001903; intron_7:67025534-67026443,intron_5:67023328-67024937,intron_6:67025012-67025526,; LDM_E,LDM_A 3,2 118,40 PITPNB

oar_circ_0001318 chr17 6710761 6728301 + 17540 16739 ENSOARG00000005862; intron_6:6725367-6725968,intron_2:6710993-6711205,intron_4:6719957-6725030,intron_7:6726088-6728154,exon_2:6710761-6710993,intron_3:6711306-6719861,; LDM_E,LDM_A 5,3 288,80 LRBA

oar_circ_0001319 chr17 6725030 6728301 + 3271 2667 ENSOARG00000005862; intron_7:6726088-6728154,intron_6:6725367-6725968,; LDM_E 3 42 LRBA

oar_circ_0001320 chr17 67371985 67381096 - 9111 8832 ENSOARG00000002106; intron_26:67372116-67373016,exon_3:67373016-67373289,intron_27:67373289-67380948,; LDM_E,LDM_A 5,2 356,69 TTC28

oar_circ_0001321 chr17 6766348 6769256 + 2908 2624 ENSOARG00000005862; intron_18:6768826-6769174,intron_17:6766441-6768717,; LDM_A 2 10 LRBA

oar_circ_0001322 chr17 67698253 67701158 - 2905 2423 ENSOARG00000002461; intron_11:67698344-67700767,; LDM_E 3 44 CHEK2

oar_circ_0001323 chr17 67875146 67876380 + 1234 1234 -- -- LDM_E 2 0 --

oar_circ_0001324 chr17 67924831 67926418 + 1587 1344 ENSOARG00000003095; intron_3:67924963-67926307,; LDM_E 2 11 ZNRF3

oar_circ_0001325 chr17 6802903 6819068 + 16165 15929 ENSOARG00000005862; intron_27:6810194-6814627,intron_26:6809621-6810086,exon_29:6818620-6819068,intron_25:6803629-6809493,intron_24:6803057-6803448,exon_24:6802903-6803057,exon_25:6803448-6803629,exon_28:6814627-6814784,intron_28:6814784-6818620,; LDM_E 2 283 LRBA

oar_circ_0001326 chr17 68316820 68321555 + 4735 4402 ENSOARG00000004984; intron_2:68316946-68318920,intron_3:68319043-68321471,; LDM_E 8 35 NF2

oar_circ_0001327 chr17 68408514 68409537 + 1023 1023 -- -- LDM_A 2 0 --

oar_circ_0001328 chr17 68408522 68409545 + 1023 1023 -- -- LDM_A 2 0 --

oar_circ_0001329 chr17 68579359 68590897 + 11538 11192 ENSOARG00000005890; intron_2:68579412-68582122,intron_4:68590374-68590780,intron_3:68582208-68590284,; LDM_A 2 185 MTMR3

oar_circ_0001330 chr17 68582122 68590897 + 8775 8482 ENSOARG00000005890; intron_4:68590374-68590780,intron_3:68582208-68590284,; LDM_A 2 146 MTMR3

oar_circ_0001331 chr17 68879550 68879745 - 195 195 -- -- LDM_A 3 0 --

oar_circ_0001332 chr17 69001014 69021324 - 20310 20310 -- -- LDM_A 3 0 --

oar_circ_0001333 chr17 69325794 69326104 + 310 310 -- -- LDM_E,LDM_A 2,2 0,0 --

oar_circ_0001334 chr17 70586653 70594240 + 7587 7587 ENSOARG00000012898; intron_1:70485578-70612174,; LDM_A 4 46 CABIN1

oar_circ_0001335 chr17 70932384 70941117 - 8733 8575 ENSOARG00000014331; exon_5:70932384-70932592,exon_4:70933533-70934727,intron_17:70935144-70938148,exon_3:70934731-70935144,intron_15:70932592-70933533,intron_18:70938302-70940927,exon_1:70940927-70941117,; LDM_A 3 199 SPECC1L

oar_circ_0001336 chr17 71273218 71274975 + 1757 1757 ENSOARG00000015529; exon_2:71274785-71274975,intron_1:71273401-71274785,exon_1:71273218-71273401,; LDM_E,LDM_A 2,2 85,44 MAPK1

oar_circ_0001337 chr17 71273218 71279902 + 6684 6567 ENSOARG00000015529; intron_1:71273401-71274785,exon_1:71273218-71273401,intron_2:71274975-71279785,exon_2:71274785-71274975,; LDM_E,LDM_A 9,5 384,391 MAPK1

oar_circ_0001338 chr17 71273218 71288580 + 15362 14888 ENSOARG00000015529; exon_1:71273218-71273401,intron_1:71273401-71274785,exon_2:71274785-71274975,intron_3:71279902-71283588,intron_5:71284094-71288470,intron_4:71283703-71283962,intron_2:71274975-71279785,; LDM_E 2 678 MAPK1

oar_circ_0001339 chr17 71273218 71289847 + 16629 16029 ENSOARG00000015529; exon_2:71274785-71274975,intron_5:71284094-71288470,intron_2:71274975-71279785,intron_1:71273401-71274785,intron_3:71279902-71283588,intron_4:71283703-71283962,exon_1:71273218-71273401,intron_6:71288580-71289721,; LDM_E 4 688 MAPK1

oar_circ_0001340 chr17 71622064 71641121 + 19057 19057 ENSOARG00000025715; exon_1:71621657-71623107,intron_1:71623107-71642341,; LDM_A 3 896 --

oar_circ_0001341 chr17 72039846 72041111 - 1265 1265 ENSOARG00000017566; intron_1:71881514-72067419,; LDM_E,LDM_A 2,53 11,43 C22orf39

oar_circ_0001342 chr17 72053556 72055660 - 2104 2104 -- -- LDM_E 2 0 --

oar_circ_0001343 chr17 7555328 7555660 - 332 332 ENSOARG00000006787; exon_3:7555328-7555660,; LDM_E,LDM_A 3,2 37,8 DCLK2

oar_circ_0001344 chr17 9393137 9394902 + 1765 1763 ENSOARG00000007064; exon_2:9393137-9394900,; LDM_E 5 609 -

oar_circ_0001345 chr17 9771771 9778831 + 7060 6771 ENSOARG00000007116; intron_5:9771902-9778673,; LDM_E,LDM_A 4,3 104,41 -

oar_circ_0001346 chr17 9946519 10000986 - 54467 54073 ENSOARG00000007284; exon_18:9946519-9946679,intron_6:9946679-9948046,intron_8:9960074-9969014,intron_7:9948152-9960015,intron_10:9975542-10000920,intron_9:9969102-9975467,; LDM_A 12 1791 ARHGAP10

oar_circ_0001347 chr17 9946519 9960074 - 13555 13390 ENSOARG00000007284; exon_18:9946519-9946679,intron_7:9948152-9960015,intron_6:9946679-9948046,; LDM_E,LDM_A 5,4 503,325 ARHGAP10

oar_circ_0001348 chr17 9946519 9969102 - 22583 22330 ENSOARG00000007284; intron_8:9960074-9969014,intron_7:9948152-9960015,exon_18:9946519-9946679,intron_6:9946679-9948046,; LDM_E 12 716 ARHGAP10

oar_circ_0001349 chr17 9946519 9975542 - 29023 28695 ENSOARG00000007284; intron_8:9960074-9969014,intron_6:9946679-9948046,intron_7:9948152-9960015,exon_18:9946519-9946679,intron_9:9969102-9975467,; LDM_E,LDM_A 31,41 1100,732 ARHGAP10

oar_circ_0001350 chr18 1049073 1065908 + 16835 10276 ENSOARG00000026419; intron_1:1049982-1059312,exon_1:1049660-1049982,exon_2:1059312-1059936,; LDM_E 6 468 --

oar_circ_0001351 chr18 1057347 1062534 + 5187 5187 ENSOARG00000026419; exon_2:1059312-1059936,intron_1:1049982-1059312,; LDM_E 2 296 --

oar_circ_0001352 chr18 1229834 1231371 + 1537 1537 -- -- LDM_A 2 0 --

oar_circ_0001353 chr18 13218976 13229370 - 10394 10294 ENSOARG00000010343; exon_33:13229191-13229370,intron_3:13224534-13227134,exon_35:13224320-13224534,intron_4:13227234-13229191,intron_2:13219223-13224320,exon_36:13218976-13219223,; LDM_E 2 652 CHD2

oar_circ_0001354 chr18 13272894 13302172 - 29278 28355 ENSOARG00000010343; intron_33:13286853-13296367,exon_14:13272894-13273085,exon_7:13284091-13284317,intron_34:13296429-13298081,intron_32:13285143-13286712,intron_35:13298168-13301940,intron_27:13279483-13281397,exon_12:13275919-13276136,intron_29:13281768-13282665,exon_11:13281397-13281585,intron_24:13273085-13273952,intron_31:13284317-13285009,intron_30:13282766-13284091,intron_25:13274042-13275919,intron_26:13276136-13279358,exon_2:13301940-13302172,; LDM_E,LDM_A 5,4 2747,1571 CHD2

oar_circ_0001355 chr18 13279358 13285143 - 5785 5242 ENSOARG00000010343; exon_7:13284091-13284317,intron_30:13282766-13284091,intron_29:13281768-13282665,intron_31:13284317-13285009,intron_27:13279483-13281397,exon_11:13281397-13281585,; LDM_A 2 201 CHD2

oar_circ_0001356 chr18 13279358 13286853 - 7495 6811 ENSOARG00000010343; intron_29:13281768-13282665,exon_7:13284091-13284317,intron_27:13279483-13281397,intron_32:13285143-13286712,exon_11:13281397-13281585,intron_30:13282766-13284091,intron_31:13284317-13285009,; LDM_E 2 465 CHD2

oar_circ_0001357 chr18 13279358 13298168 - 18810 17977 ENSOARG00000010343; exon_7:13284091-13284317,intron_34:13296429-13298081,intron_30:13282766-13284091,intron_31:13284317-13285009,intron_33:13286853-13296367,intron_29:13281768-13282665,exon_11:13281397-13281585,intron_32:13285143-13286712,intron_27:13279483-13281397,; LDM_E 3 1511 CHD2

oar_circ_0001358 chr18 13296367 13302172 - 5805 5656 ENSOARG00000010343; exon_2:13301940-13302172,intron_35:13298168-13301940,intron_34:13296429-13298081,; LDM_E,LDM_A 9,14 805,470 CHD2

oar_circ_0001359 chr18 13298081 13314527 - 16446 16446 ENSOARG00000010343; intron_36:13302172-13319999,exon_2:13301940-13302172,intron_35:13298168-13301940,; LDM_A 2 1345 CHD2

oar_circ_0001360 chr18 1334073 1355324 - 21251 20937 ENSOARG00000009094; intron_7:1339378-1347200,exon_8:1334242-1334398,exon_7:1335790-1335955,exon_4:1347200-1348447,intron_4:1334398-1335790,exon_6:1336546-1336752,intron_8:1348447-1355019,intron_6:1336752-1339233,intron_5:1335955-1336546,exon_3:1355019-1355324,; LDM_E 6 5009 UBE3A

oar_circ_0001361 chr18 1355019 1386808 - 31789 29210 ENSOARG00000009094; intron_10:1381011-1384274,exon_3:1355019-1355324,intron_9:1355324-1380966,; LDM_E 4 4358 UBE3A

oar_circ_0001362 chr18 1367636 1386808 - 19172 19172 ENSOARG00000009094; intron_10:1381011-1384274,intron_9:1355324-1380966,; LDM_A 2 2107 UBE3A

oar_circ_0001363 chr18 13705777 13709917 - 4140 4140 ENSOARG00000010518; exon_4:13705777-13706071,intron_2:13706071-13709659,exon_3:13709659-13709917,; LDM_E 2 69 ST8SIA2

oar_circ_0001364 chr18 1380966 1386808 - 5842 3263 ENSOARG00000009094; intron_10:1381011-1384274,; LDM_A 6 447 UBE3A

oar_circ_0001365 chr18 1384274 1386808 - 2534 2534 -- -- LDM_E 2 0 --

oar_circ_0001366 chr18 1385128 1386100 - 972 972 -- -- LDM_E 2 0 --

oar_circ_0001367 chr18 15645047 15666858 + 21811 21526 ENSOARG00000010666; intron_14:15658221-15659934,intron_15:15660067-15666683,exon_13:15645047-15645240,intron_13:15648112-15658178,intron_12:15645240-15648003,exon_17:15666683-15666858,; LDM_A 4 833 AKAP13

oar_circ_0001368 chr18 16268033 16280779 + 12746 11158 ENSOARG00000010742; intron_4:16268242-16279025,exon_5:16279025-16279191,exon_4:16268033-16268242,; LDM_A 2 13 AGBL1

oar_circ_0001369 chr18 16796440 16796651 + 211 211 -- -- LDM_E 2 0 --

oar_circ_0001370 chr18 19723285 19726561 + 3276 3172 ENSOARG00000011286; intron_1:19723389-19726361,exon_2:19726361-19726561,; LDM_A 4 159 ABHD2

oar_circ_0001371 chr18 20391863 20398803 - 6940 6664 ENSOARG00000011951; intron_4:20391935-20397909,intron_5:20398021-20398711,; LDM_A 3 52 -

oar_circ_0001372 chr18 20592975 20593445 - 470 152 ENSOARG00000012014; exon_7:20592975-20593127,; LDM_E,LDM_A 6,12 2,4 IDH2

oar_circ_0001373 chr18 21120849 21133894 - 13045 12699 ENSOARG00000012943; intron_10:21120950-21123030,intron_11:21123093-21124639,intron_12:21124701-21133774,; LDM_E 3 387 CRTC3

oar_circ_0001374 chr18 21214809 21222598 - 7789 7206 ENSOARG00000013053; exon_35:21217795-21218008,intron_8:21220643-21221291,exon_36:21216894-21217061,intron_4:21214932-21216894,intron_6:21218008-21220029,intron_5:21217061-21217795,intron_7:21220117-21220507,intron_9:21221394-21222465,; LDM_A 2 312 IQGAP1

oar_circ_0001375 chr18 21216894 21220643 - 3749 3525 ENSOARG00000013053; exon_36:21216894-21217061,intron_7:21220117-21220507,intron_5:21217061-21217795,intron_6:21218008-21220029,exon_35:21217795-21218008,; LDM_A 2 185 IQGAP1

oar_circ_0001376 chr18 21227511 21232823 - 5312 4886 ENSOARG00000013053; intron_16:21229407-21230468,intron_15:21229015-21229251,exon_20:21232652-21232823,exon_25:21229251-21229407,intron_14:21227736-21228851,exon_26:21228851-21229015,intron_17:21230677-21231070,exon_27:21227511-21227736,intron_20:21231496-21232652,exon_24:21230468-21230677,; LDM_A 2 286 IQGAP1

oar_circ_0001377 chr18 21249049 21251932 - 2883 2673 ENSOARG00000013053; intron_26:21249174-21250540,exon_14:21250540-21250701,intron_27:21250701-21250904,intron_28:21251068-21251847,exon_13:21250904-21251068,; LDM_A 2 72 IQGAP1

oar_circ_0001378 chr18 21560205 21560428 + 223 223 ENSOARG00000013306; intron_3:21559727-21561629,; LDM_A 2 72 ALPK3

oar_circ_0001379 chr18 21577653 21579614 + 1961 1740 ENSOARG00000013306; exon_9:21578672-21579614,exon_8:21578259-21578569,intron_7:21577771-21578259,; LDM_A 2 4976 ALPK3

oar_circ_0001380 chr18 21677633 21679620 + 1987 1372 ENSOARG00000013352; intron_11:21677764-21679136,; LDM_E 3 1 SLC28A1

oar_circ_0001381 chr18 21833525 21835176 + 1651 1594 ENSOARG00000013382; exon_3:21834985-21835176,intron_2:21833582-21834985,; LDM_A 5 100 PDE8A

oar_circ_0001382 chr18 22146430 22151056 - 4626 4359 ENSOARG00000013800; intron_3:22149840-22150922,intron_2:22146613-22149707,exon_11:22146430-22146613,; LDM_A 6 44 FSD2

oar_circ_0001383 chr18 22146430 22165444 - 19014 17844 ENSOARG00000013800; exon_8:22153037-22153188,exon_11:22146430-22146613,intron_10:22162646-22164277,intron_2:22146613-22149707,intron_9:22161242-22162415,intron_5:22153188-22153928,intron_7:22156854-22159811,exon_1:22164508-22165214,intron_4:22151056-22153037,intron_3:22149840-22150922,intron_6:22154063-22156698,exon_3:22162415-22162646,intron_8:22159939-22161219,; LDM_A 2 408 FSD2

oar_circ_0001384 chr18 22150922 22159933 - 9011 8469 ENSOARG00000013800; intron_6:22154063-22156698,exon_6:22156698-22156854,intron_7:22156854-22159811,intron_5:22153188-22153928,intron_4:22151056-22153037,; LDM_E 3 259 FSD2

oar_circ_0001385 chr18 22190471 22191528 + 1057 906 ENSOARG00000013840; intron_2:22190645-22191377,exon_2:22190471-22190645,; LDM_A 3 14 WHAMM

oar_circ_0001386 chr18 22236083 22257713 - 21630 21452 ENSOARG00000013896; intron_7:22250038-22257556,exon_1:22257556-22257760,intron_6:22236176-22249906,; LDM_A 3 473 HOMER2

oar_circ_0001387 chr18 22520416 22532902 - 12486 12380 ENSOARG00000014053; exon_10:22520416-22520614,intron_6:22528118-22532745,intron_5:22520614-22528012,exon_8:22532745-22532902,; LDM_E 5 1901 BTBD1

oar_circ_0001388 chr18 22620648 22621709 - 1061 1061 -- -- LDM_A 2 0 --

oar_circ_0001389 chr18 22635456 22641979 - 6523 6237 ENSOARG00000014131; exon_4:22638970-22639129,intron_2:22639129-22641840,intron_1:22635603-22638970,; LDM_E 4 648 -

oar_circ_0001390 chr18 22635456 22646961 - 11505 11142 ENSOARG00000014131; exon_4:22638970-22639129,intron_2:22639129-22641840,intron_3:22641979-22646884,intron_1:22635603-22638970,; LDM_E 3 1039 -

oar_circ_0001391 chr18 22997837 23012330 + 14493 14145 ENSOARG00000014300; exon_5:23002069-23002228,intron_4:22997971-23002069,intron_5:23002228-23008910,intron_6:23009014-23012220,; LDM_E 4 295 SH3GL3

oar_circ_0001392 chr18 23279413 23292896 + 13483 12948 ENSOARG00000014330; intron_11:23285015-23286060,intron_9:23279525-23283250,intron_10:23283389-23284964,exon_12:23286060-23286265,intron_13:23290396-23292811,intron_12:23286265-23290248,; LDM_E 2 287 ADAMTSL3

oar_circ_0001393 chr18 25098932 25115527 + 16595 16385 ENSOARG00000015252; intron_3:25113196-25114415,intron_2:25099093-25113025,intron_4:25114524-25115426,exon_3:25113025-25113196,exon_2:25098932-25099093,; LDM_A 2 1219 FAH

oar_circ_0001394 chr18 26437635 26440872 - 3237 2884 ENSOARG00000016152; intron_13:26438306-26440735,intron_12:26437778-26438233,; LDM_E 2 63 MTMR10

oar_circ_0001395 chr18 27501187 27512664 - 11477 11249 ENSOARG00000016530; intron_24:27501464-27511505,exon_5:27501187-27501464,intron_25:27511608-27512539,; LDM_A 2 258 TJP1

oar_circ_0001396 chr18 27859195 27864247 + 5052 5052 ENSOARG00000016585; intron_3:27859353-27864072,exon_4:27864072-27864247,exon_3:27859195-27859353,; LDM_E 2 146 TM2D3

oar_circ_0001397 chr18 28057585 28076400 + 18815 18289 ENSOARG00000016657; intron_5:28074512-28076311,intron_3:28071600-28072052,intron_4:28072196-28074435,intron_2:28057690-28071489,; LDM_A 2 107 PCSK6

oar_circ_0001398 chr18 30178352 30198618 + 20266 19624 ENSOARG00000001259; intron_4:30183064-30190123,intron_6:30193137-30196258,intron_2:30178454-30182959,intron_8:30197770-30198527,intron_7:30196343-30197709,intron_5:30190180-30192996,; LDM_E 6 637 UBE2Q2

oar_circ_0001399 chr18 30178352 30207447 + 29095 28394 ENSOARG00000001259; intron_7:30196343-30197709,intron_8:30197770-30198527,intron_5:30190180-30192996,intron_6:30193137-30196258,intron_4:30183064-30190123,intron_2:30178454-30182959,intron_9:30198618-30207388,; LDM_E 5 876 UBE2Q2

oar_circ_0001400 chr18 30268936 30281146 - 12210 11895 ENSOARG00000001330; intron_2:30274665-30280996,intron_1:30269021-30274585,; LDM_A 4 5 NRG4

oar_circ_0001401 chr18 30274585 30281146 - 6561 6331 ENSOARG00000001330; intron_2:30274665-30280996,; LDM_A 2 3 NRG4

oar_circ_0001402 chr18 30432423 30435653 + 3230 3098 ENSOARG00000001439; intron_1:30432555-30435464,exon_2:30435464-30435653,; LDM_A 3 5 C15orf27

oar_circ_0001403 chr18 30902053 30909199 - 7146 6660 ENSOARG00000001531; intron_14:30902890-30904268,intron_15:30904350-30909056,intron_13:30902161-30902737,; LDM_E,LDM_A 3,2 253,76 SCAPER

oar_circ_0001404 chr18 30902053 30929461 - 27408 26669 ENSOARG00000001531; exon_15:30914251-30914407,intron_13:30902161-30902737,intron_14:30902890-30904268,intron_17:30914407-30925520,intron_18:30925661-30929349,intron_16:30909199-30914251,intron_15:30904350-30909056,; LDM_E 4 764 SCAPER

oar_circ_0001405 chr18 31192712 31204593 - 11881 10973 ENSOARG00000001693; exon_5:31199839-31199992,intron_3:31199992-31200935,exon_2:31204401-31204593,intron_4:31201037-31204077,intron_1:31192851-31199496,; LDM_E,LDM_A 2,2 1209,351 TSPAN3

oar_circ_0001406 chr18 31204077 31204593 - 516 192 ENSOARG00000001693; exon_2:31204401-31204593,; LDM_E,LDM_A 23,7 141,103 TSPAN3

oar_circ_0001407 chr18 31419219 31425609 - 6390 6304 ENSOARG00000001750; intron_5:31419382-31425523,exon_2:31419219-31419382,; LDM_E,LDM_A 4,2 177,43 PEAK1

oar_circ_0001408 chr18 31642533 31645702 + 3169 3036 ENSOARG00000001765; intron_4:31642746-31645569,exon_4:31642533-31642746,; LDM_E,LDM_A 2,3 93,68 HMG20A

oar_circ_0001409 chr18 31642533 31646827 + 4294 4129 ENSOARG00000001765; intron_4:31642746-31645569,exon_4:31642533-31642746,intron_5:31645702-31646795,; LDM_E 2 110 HMG20A

oar_circ_0001410 chr18 32239249 32242636 + 3387 3028 ENSOARG00000002056; intron_2:32239393-32241700,intron_3:32241790-32242511,; LDM_A 2 14 PTPN9

oar_circ_0001411 chr18 32293500 32293750 + 250 250 -- -- LDM_E 2 0 --

oar_circ_0001412 chr18 32327242 32332983 + 5741 5741 ENSOARG00000002221; exon_13:32328807-32328991,intron_14:32330642-32332813,exon_12:32327242-32327481,exon_15:32332813-32332983,intron_13:32328991-32330068,exon_14:32330068-32330642,intron_12:32327481-32328807,; LDM_E,LDM_A 3,2 389,120 SIN3A

oar_circ_0001413 chr18 33142323 33143780 + 1457 1264 ENSOARG00000003804; exon_9:33143581-33143780,exon_7:33142323-33142508,intron_7:33142508-33143388,; LDM_E 2 18 SEMA7A

oar_circ_0001414 chr18 39349465 39349972 - 507 507 ENSOARG00000005042; intron_18:39190590-39382020,; LDM_A 2 9 PRKD1

oar_circ_0001415 chr18 40172511 40180985 + 8474 7995 ENSOARG00000005268; intron_10:40177588-40178652,exon_12:40180803-40180985,intron_7:40172618-40173295,intron_9:40177131-40177458,intron_11:40178960-40180803,exon_11:40178652-40178960,intron_8:40173412-40177006,; LDM_E,LDM_A 7,3 531,124 G2E3

oar_circ_0001416 chr18 40509642 40533957 - 24315 23783 ENSOARG00000005730; intron_15:40525727-40533294,intron_13:40511142-40522250,intron_16:40533368-40533853,intron_14:40522424-40525645,exon_4:40522250-40522424,intron_12:40509784-40511012,; LDM_E 8 2095 STRN3

oar_circ_0001417 chr18 40522250 40533957 - 11707 11447 ENSOARG00000005730; intron_14:40522424-40525645,intron_16:40533368-40533853,exon_4:40522250-40522424,intron_15:40525727-40533294,; LDM_E,LDM_A 14,7 1174,639 STRN3

oar_circ_0001418 chr18 40525645 40533957 - 8312 8052 ENSOARG00000005730; intron_16:40533368-40533853,intron_15:40525727-40533294,; LDM_A 4 495 STRN3

oar_circ_0001419 chr18 40773604 40775032 - 1428 1300 ENSOARG00000006260; exon_29:40774783-40775032,intron_7:40773732-40774783,; LDM_E 4 47 HEATR5A

oar_circ_0001420 chr18 40802873 40841439 - 38566 37010 ENSOARG00000006260; intron_32:40835645-40838773,intron_26:40823555-40824538,exon_15:40809701-40809865,exon_9:40826151-40826307,intron_27:40824789-40826151,intron_30:40828532-40834658,intron_19:40803064-40803687,exon_12:40818956-40819109,exon_8:40826898-40827154,exon_6:40834658-40834833,intron_25:40819109-40823443,intron_24:40817992-40818956,exon_16:40809318-40809494,intron_29:40827154-40828374,intron_23:40812426-40817892,intron_34:40840014-40841253,exon_10:40824538-40824789,intron_33:40838882-40839802,intron_22:40809865-40812316,exon_2:40841253-40841439,intron_20:40803793-40809318,exon_7:40828374-40828532,exon_3:40839802-40840014,intron_28:40826307-40826898,exon_18:40802873-40803064,; LDM_E,LDM_A 7,4 991,370 HEATR5A

oar_circ_0001421 chr18 40823443 40841439 - 17996 17625 ENSOARG00000006260; intron_32:40835645-40838773,intron_27:40824789-40826151,intron_26:40823555-40824538,intron_33:40838882-40839802,intron_31:40834833-40835495,intron_29:40827154-40828374,exon_7:40828374-40828532,exon_3:40839802-40840014,intron_34:40840014-40841253,exon_6:40834658-40834833,exon_10:40824538-40824789,exon_2:40841253-40841439,exon_9:40826151-40826307,exon_8:40826898-40827154,intron_28:40826307-40826898,intron_30:40828532-40834658,; LDM_E 2 417 HEATR5A

oar_circ_0001422 chr18 40828374 40841439 - 13065 12144 ENSOARG00000006260; intron_33:40838882-40839802,exon_2:40841253-40841439,exon_7:40828374-40828532,exon_3:40839802-40840014,exon_6:40834658-40834833,intron_32:40835645-40838773,intron_34:40840014-40841253,intron_30:40828532-40834658,; LDM_E,LDM_A 4,4 256,123 HEATR5A

oar_circ_0001423 chr18 40834658 40841439 - 6781 5860 ENSOARG00000006260; intron_34:40840014-40841253,exon_2:40841253-40841439,intron_33:40838882-40839802,intron_32:40835645-40838773,exon_6:40834658-40834833,exon_3:40839802-40840014,; LDM_E,LDM_A 39,29 169,100 HEATR5A

oar_circ_0001424 chr18 41435463 41439351 + 3888 3888 ENSOARG00000006439; exon_1:41435463-41439351,; LDM_E,LDM_A 5,4 3634,1806 ARHGAP5

oar_circ_0001425 chr18 41435463 41460809 + 25346 25198 ENSOARG00000006439; exon_1:41435463-41439351,intron_1:41439351-41460661,; LDM_E,LDM_A 10,4 4850,2220 ARHGAP5

oar_circ_0001426 chr18 41460661 41486076 + 25415 24951 ENSOARG00000006439; intron_3:41481201-41483710,intron_4:41483842-41485970,intron_2:41460809-41481123,; LDM_E,LDM_A 4,5 1435,456 ARHGAP5

oar_circ_0001427 chr18 41892258 41893639 + 1381 1284 ENSOARG00000006557; intron_6:41892355-41893475,exon_7:41893475-41893639,; LDM_A 2 40 AKAP6

oar_circ_0001428 chr18 42062140 42063231 + 1091 1091 ENSOARG00000006557; intron_11:42046321-42071772,; LDM_E 2 38 AKAP6

oar_circ_0001429 chr18 42403785 42405429 - 1644 1644 -- -- LDM_E 2 0 --

oar_circ_0001430 chr18 42532531 42532935 + 404 404 -- -- LDM_E 7 0 --

oar_circ_0001431 chr18 4352461 4352957 + 496 496 ENSOARG00000009413; exon_2:4352461-4352957,; LDM_E,LDM_A 2,2 102,44 CHSY1

oar_circ_0001432 chr18 43699776 43700711 - 935 935 -- -- LDM_A 2 0 --

oar_circ_0001433 chr18 43853049 43855839 - 2790 2579 ENSOARG00000006719; exon_2:43855657-43855839,intron_3:43853167-43854474,intron_4:43854567-43855657,; LDM_E 3 37 EAPP

oar_circ_0001434 chr18 44043236 44046676 - 3440 3315 ENSOARG00000006876; exon_17:44043236-44043833,intron_10:44043833-44046551,; LDM_E,LDM_A 2,2 129,106 BAZ1A

oar_circ_0001435 chr18 44094537 44094816 - 279 279 ENSOARG00000006876; exon_2:44094537-44094816,; LDM_A 2 26 BAZ1A

oar_circ_0001436 chr18 44755700 44763617 - 7917 7447 ENSOARG00000007701; intron_15:44759122-44760521,intron_16:44760650-44763461,intron_14:44755759-44758996,; LDM_E,LDM_A 29,11 244,122 RALGAPA1

oar_circ_0001437 chr18 44758996 44764871 - 5875 5521 ENSOARG00000007701; intron_15:44759122-44760521,intron_18:44764440-44764677,exon_23:44764677-44764871,intron_17:44763617-44764341,intron_16:44760650-44763461,exon_25:44763461-44763617,; LDM_E 2 153 RALGAPA1

oar_circ_0001438 chr18 44796713 44836566 - 39853 39853 ENSOARG00000007701; intron_37:44829265-44832974,intron_30:44800889-44803217,intron_38:44833032-44835859,exon_11:44803217-44803415,intron_33:44815936-44816962,intron_34:44821817-44825062,intron_36:44824155-44829221,exon_10:44813683-44813923,intron_34:44817101-44821701,exon_9:44815727-44815936,intron_29:44796862-44800751,intron_39:44835909-44836455,intron_31:44803415-44813683,intron_32:44813923-44815727,; LDM_E 5 1854 RALGAPA1

oar_circ_0001439 chr18 44829221 44836566 - 7345 7082 ENSOARG00000007701; intron_39:44835909-44836455,intron_37:44829265-44832974,intron_38:44833032-44835859,; LDM_A 4 151 RALGAPA1

oar_circ_0001440 chr18 44929064 44933837 + 4773 3253 ENSOARG00000007853; intron_6:44930328-44930946,intron_7:44931035-44931744,intron_8:44931784-44933710,; LDM_E,LDM_A 4,4 132,47 BRMS1L

oar_circ_0001441 chr18 45377108 45382934 - 5826 5477 ENSOARG00000007986; intron_3:45377206-45379725,exon_3:45382709-45382934,intron_5:45380176-45382524,intron_4:45379878-45380110,exon_6:45379725-45379878,; LDM_A 2 167 MBIP

oar_circ_0001442 chr18 45377108 45384800 - 7692 7223 ENSOARG00000007986; intron_4:45379878-45380110,intron_5:45380176-45382524,exon_6:45379725-45379878,exon_3:45382709-45382934,intron_7:45382934-45384680,intron_3:45377206-45379725,; LDM_A 3 178 MBIP

oar_circ_0001443 chr18 46376589 46397942 + 21353 19643 ENSOARG00000008191; intron_3:46394761-46396449,intron_1:46376727-46377302,intron_2:46377381-46394529,exon_3:46394529-46394761,; LDM_A 10 105 MIPOL1

oar_circ_0001444 chr18 46377302 46397942 + 20640 19068 ENSOARG00000008191; intron_3:46394761-46396449,intron_2:46377381-46394529,exon_3:46394529-46394761,; LDM_A 24 104 MIPOL1

oar_circ_0001445 chr18 46798914 46799307 + 393 393 -- -- LDM_E 2 0 --

oar_circ_0001446 chr18 48130469 48132328 + 1859 1615 ENSOARG00000008691; intron_13:48130592-48132207,; LDM_E,LDM_A 13,21 59,30 -

oar_circ_0001447 chr18 48130469 48137247 + 6778 6257 ENSOARG00000008691; intron_15:48133811-48134552,intron_16:48134643-48137108,intron_14:48132328-48133764,intron_13:48130592-48132207,; LDM_E,LDM_A 8,4 273,144 -

oar_circ_0001448 chr18 48137890 48149380 + 11490 11011 ENSOARG00000008691; intron_18:48137992-48138999,intron_19:48139060-48139595,intron_22:48144420-48146007,intron_20:48139631-48143076,exon_24:48149221-48149380,intron_23:48146136-48149221,intron_21:48143144-48144337,; LDM_E 5 286 -

oar_circ_0001449 chr18 5130911 5133227 - 2316 2192 ENSOARG00000009658; intron_3:5131176-5133103,exon_3:5130911-5131176,; LDM_E,LDM_A 32,50 90,46 ASB7

oar_circ_0001450 chr18 5165056 5171833 + 6777 6777 ENSOARG00000009711; intron_6:5167985-5169218,intron_4:5165757-5166587,intron_5:5166729-5167391,intron_7:5169390-5220463,exon_3:5165056-5165557,exon_7:5169218-5169390,exon_6:5167391-5167985,; LDM_A 2 364 CERS3

oar_circ_0001451 chr18 5165261 5171833 + 6572 6572 ENSOARG00000009711; exon_6:5167391-5167985,intron_7:5169390-5220463,intron_4:5165757-5166587,exon_7:5169218-5169390,exon_3:5165056-5165557,intron_6:5167985-5169218,intron_5:5166729-5167391,; LDM_E 2 506 CERS3

oar_circ_0001452 chr18 5167391 5167985 + 594 594 ENSOARG00000009711; exon_6:5167391-5167985,; LDM_E 4 108 CERS3

oar_circ_0001453 chr18 53702195 53711260 + 9065 8964 ENSOARG00000009198; exon_4:53702195-53702489,intron_4:53702489-53703980,exon_5:53703980-53704243,intron_5:53704243-53706610,intron_6:53706843-53711159,exon_6:53706610-53706843,; LDM_E 2 470 FAM179B

oar_circ_0001454 chr18 53702195 53717648 + 15453 15199 ENSOARG00000009198; exon_5:53703980-53704243,intron_4:53702489-53703980,exon_4:53702195-53702489,intron_7:53711260-53717495,intron_6:53706843-53711159,intron_5:53704243-53706610,exon_6:53706610-53706843,; LDM_E 2 614 FAM179B

oar_circ_0001455 chr18 53702195 53731466 + 29271 28918 ENSOARG00000009198; intron_7:53711260-53717495,exon_11:53728096-53728250,intron_5:53704243-53706610,exon_12:53731315-53731466,exon_6:53706610-53706843,intron_9:53726233-53727435,exon_5:53703980-53704243,intron_4:53702489-53703980,exon_4:53702195-53702489,intron_11:53728250-53731315,exon_10:53727435-53727608,intron_6:53706843-53711159,intron_10:53727608-53728096,intron_8:53717648-53726134,; LDM_E 5 793 FAM179B

oar_circ_0001456 chr18 53711159 53728250 + 17091 16737 ENSOARG00000009198; exon_8:53717495-53717648,intron_8:53717648-53726134,intron_9:53726233-53727435,exon_10:53727435-53727608,intron_10:53727608-53728096,intron_7:53711260-53717495,; LDM_A 2 116 FAM179B

oar_circ_0001457 chr18 53770454 53775745 + 5291 5017 ENSOARG00000009248; intron_2:53771802-53772451,intron_1:53770801-53771676,exon_4:53775577-53775745,intron_3:53772570-53775577,exon_1:53770483-53770801,; LDM_E 3 838 PRPF39

oar_circ_0001458 chr18 53770454 53782645 + 12191 11806 ENSOARG00000009248; intron_3:53772570-53775577,intron_4:53775745-53780423,exon_5:53780423-53780589,intron_5:53780589-53781006,exon_1:53770483-53770801,exon_7:53782480-53782645,exon_4:53775577-53775745,intron_6:53781117-53782480,intron_1:53770801-53771676,intron_2:53771802-53772451,; LDM_E 2 1354 PRPF39

oar_circ_0001459 chr18 53831692 53840976 + 9284 9128 ENSOARG00000009429; intron_10:53831899-53833625,intron_11:53833839-53839616,exon_12:53839616-53839774,exon_10:53831692-53831899,intron_12:53839774-53840820,exon_11:53833625-53833839,; LDM_A 4 39 FANCM

oar_circ_0001460 chr18 53846831 53848743 + 1912 1912 ENSOARG00000009429; exon_14:53846831-53848743,; LDM_E,LDM_A 36,14 598,514 FANCM

oar_circ_0001461 chr18 53846831 53853645 + 6814 6553 ENSOARG00000009429; intron_14:53848743-53853384,exon_14:53846831-53848743,; LDM_E,LDM_A 7,15 802,568 FANCM

oar_circ_0001462 chr18 54173331 54176993 - 3662 3437 ENSOARG00000010147; intron_18:54173537-54176064,exon_10:54173331-54173537,intron_19:54176159-54176863,; LDM_A 3 12 TP53BP1

oar_circ_0001463 chr18 54417464 54419185 + 1721 1361 ENSOARG00000011351; intron_5:54417591-54418952,; LDM_A 2 1 WDR76

oar_circ_0001464 chr18 5445519 5445736 + 217 217 -- -- LDM_E 2 0 --

oar_circ_0001465 chr18 55005952 55015357 - 9405 9025 ENSOARG00000011731; exon_22:55012887-55013074,exon_25:55005952-55006191,intron_7:55006191-55007773,intron_8:55007863-55010527,intron_9:55010673-55012887,intron_10:55013074-55015213,; LDM_E,LDM_A 2,2 139,79 CCDC88C

oar_circ_0001466 chr18 55108054 55108918 - 864 864 ENSOARG00000011731; intron_28:55068611-55121542,; LDM_A 2 4 CCDC88C

oar_circ_0001467 chr18 55205220 55219079 + 13859 13859 -- -- LDM_E 2 0 --

oar_circ_0001468 chr18 55435955 55453350 - 17395 16726 ENSOARG00000012362; intron_5:55449350-55449736,intron_2:55436155-55438382,intron_4:55444458-55449233,exon_8:55444266-55444458,intron_6:55449837-55450600,intron_3:55438497-55444266,exon_10:55435955-55436155,intron_8:55451841-55453182,intron_7:55450676-55451749,; LDM_E 2 150 TC2N

oar_circ_0001469 chr18 55660114 55674565 - 14451 14100 ENSOARG00000012626; intron_17:55662099-55664400,intron_19:55668179-55674394,exon_4:55664400-55664679,exon_6:55660114-55660280,intron_16:55660280-55662030,intron_18:55664679-55668068,; LDM_E 2 565 TRIP11

oar_circ_0001470 chr18 55773128 55773778 + 650 201 ENSOARG00000012889; exon_10:55773577-55773778,; LDM_E 2 21 CPSF2

oar_circ_0001471 chr18 5684703 5685932 + 1229 981 ENSOARG00000009766; intron_10:5684805-5685786,; LDM_E 2 3 ADAMTS17

oar_circ_0001472 chr18 5726262 5726504 - 242 242 -- -- LDM_E 2 0 --

oar_circ_0001473 chr18 5752079 5758004 + 5925 5789 ENSOARG00000009766; exon_16:5752079-5752237,intron_16:5752237-5755361,exon_17:5755361-5755521,intron_17:5755521-5757868,; LDM_E 4 92 ADAMTS17

oar_circ_0001474 chr18 58687619 58694896 - 7277 7011 ENSOARG00000015465; exon_7:58689387-58689556,exon_3:58694733-58694896,intron_22:58691886-58692334,intron_21:58689556-58691725,exon_8:58687619-58688092,intron_24:58693762-58694733,intron_20:58688092-58689387,exon_6:58691725-58691886,intron_23:58692469-58693631,; LDM_A 2 357 DICER1

oar_circ_0001475 chr18 58687619 58695887 - 8268 8002 ENSOARG00000015465; exon_7:58689387-58689556,exon_3:58694733-58694896,intron_22:58691886-58692334,intron_21:58689556-58691725,exon_8:58687619-58688092,exon_2:58695698-58695887,intron_25:58694896-58695698,intron_24:58693762-58694733,intron_20:58688092-58689387,intron_23:58692469-58693631,exon_6:58691725-58691886,; LDM_E,LDM_A 12,2 678,378 DICER1

oar_circ_0001476 chr18 59876290 59883034 - 6744 6563 ENSOARG00000016088; intron_7:59876498-59877565,exon_36:59876290-59876498,intron_8:59877644-59882932,; LDM_E 2 224 ATG2B

oar_circ_0001477 chr18 59910230 59931582 - 21352 20277 ENSOARG00000016088; exon_8:59917480-59917666,intron_40:59928007-59931419,intron_30:59913038-59913811,intron_35:59917666-59918161,intron_39:59924175-59927854,exon_6:59922619-59922799,intron_36:59918258-59922619,exon_5:59923599-59923776,intron_34:59916487-59917480,exon_12:59913811-59913962,exon_2:59931419-59931582,exon_14:59912619-59912808,exon_11:59915196-59915458,intron_37:59922799-59923599,exon_9:59916332-59916487,intron_32:59915458-59916136,exon_15:59910230-59910504,intron_28:59910504-59912619,intron_31:59913962-59915196,; LDM_E 4 516 ATG2B

oar_circ_0001478 chr18 59927854 59931582 - 3728 3575 ENSOARG00000016088; intron_40:59928007-59931419,exon_2:59931419-59931582,; LDM_E 2 81 ATG2B

oar_circ_0001479 chr18 60079544 60098014 + 18470 17420 ENSOARG00000016579; exon_19:60089844-60090065,intron_18:60089649-60089844,intron_20:60093823-60095679,intron_14:60080256-60081376,intron_21:60095754-60097752,intron_19:60090065-60093760,intron_16:60084008-60087077,intron_13:60079598-60080136,intron_17:60087220-60089548,intron_15:60081486-60083886,; LDM_A 2 1344 PAPOLA

oar_circ_0001480 chr18 6009428 6030125 - 20697 20697 ENSOARG00000009878; intron_5:6009616-6026659,intron_7:6027679-6029790,intron_6:6026719-6027459,exon_7:6009428-6009616,intron_8:6029928-6052837,exon_5:6027459-6027679,; LDM_E 3 996 MEF2A

oar_circ_0001481 chr18 60358444 60363401 + 4957 4901 ENSOARG00000000106; exon_2:60358444-60358609,intron_2:60358609-60363345,; LDM_E,LDM_A 6,9 101,23 VRK1

oar_circ_0001482 chr18 60358444 60384683 + 26239 24318 ENSOARG00000000106; intron_10:60380821-60384504,intron_2:60358609-60363345,intron_4:60371780-60372649,exon_11:60384504-60384683,exon_2:60358444-60358609,intron_5:60372737-60376754,intron_7:60377074-60379434,intron_3:60363401-60371710,; LDM_E,LDM_A 17,8 745,131 VRK1

oar_circ_0001483 chr18 60358444 60400508 + 42064 41126 ENSOARG00000000106; intron_5:60372737-60376754,intron_9:60380467-60380762,intron_11:60384683-60400417,intron_2:60358609-60363345,intron_10:60380821-60384504,intron_7:60377074-60379434,intron_3:60363401-60371710,exon_11:60384504-60384683,intron_8:60379567-60380346,exon_2:60358444-60358609,intron_4:60371780-60372649,; LDM_E 7 1164 VRK1

oar_circ_0001484 chr18 60379434 60384683 + 5249 3862 ENSOARG00000000106; intron_10:60380821-60384504,exon_11:60384504-60384683,; LDM_A 2 22 VRK1

oar_circ_0001485 chr18 6074185 6074873 - 688 688 ENSOARG00000009878; exon_2:6074185-6074377,intron_10:6074377-6166066,; LDM_A 2 44 MEF2A

oar_circ_0001486 chr18 63126679 63129937 - 3258 3036 ENSOARG00000000401; exon_6:63126679-63126936,intron_10:63127316-63129788,intron_9:63126936-63127243,; LDM_A 5 99 SETD3

oar_circ_0001487 chr18 63126679 63133857 - 7178 6753 ENSOARG00000000401; intron_11:63129937-63132149,exon_6:63126679-63126936,intron_12:63132242-63133747,intron_9:63126936-63127243,intron_10:63127316-63129788,; LDM_E,LDM_A 34,15 442,142 SETD3

oar_circ_0001488 chr18 63164753 63167385 + 2632 2418 ENSOARG00000000495; intron_2:63165003-63165669,intron_3:63165751-63167253,exon_2:63164753-63165003,; LDM_E,LDM_A 11,6 58,32 CCNK

oar_circ_0001489 chr18 63466953 63472893 + 5940 193 ENSOARG00000001154; exon_1:63472700-63472893,; LDM_E 2 16 EML1

oar_circ_0001490 chr18 63540239 63542716 + 2477 2351 ENSOARG00000001154; exon_12:63540239-63540394,intron_12:63540394-63542590,; LDM_E,LDM_A 2,4 8,7 EML1

oar_circ_0001491 chr18 63701321 63711401 + 10080 10080 ENSOARG00000001442; exon_2:63701321-63701490,intron_2:63701490-63711223,exon_3:63711223-63711401,; LDM_E 2 401 EVL

oar_circ_0001492 chr18 63794280 63794592 - 312 312 -- -- LDM_E 2 0 --

oar_circ_0001493 chr18 64429435 64443645 + 14210 14210 -- -- LDM_E,LDM_A 11,2 0,0 --

oar_circ_0001494 chr18 64524617 64525157 + 540 540 -- -- LDM_E 2 0 --

oar_circ_0001495 chr18 64560040 64560410 + 370 370 -- -- LDM_E 2 0 --

oar_circ_0001496 chr18 64560090 64568429 + 8339 8339 -- -- LDM_E 2 0 --

oar_circ_0001497 chr18 64565781 64568429 + 2648 2648 -- -- LDM_E 3 0 --

oar_circ_0001498 chr18 64565781 64576177 + 10396 10396 -- -- LDM_E 3 0 --

oar_circ_0001499 chr18 64583168 64591224 + 8056 8056 -- -- LDM_E 2 0 --

oar_circ_0001500 chr18 64587665 64592314 + 4649 4649 -- -- LDM_E 24 0 --

oar_circ_0001501 chr18 64587665 64601482 + 13817 13817 -- -- LDM_E 2 0 --

oar_circ_0001502 chr18 64589281 64592314 + 3033 3033 -- -- LDM_E 4 0 --

oar_circ_0001503 chr18 64602277 64607518 + 5241 5241 -- -- LDM_E,LDM_A 19,2 0,0 --

oar_circ_0001504 chr18 64623542 64629839 + 6297 6297 -- -- LDM_E 9 0 --

oar_circ_0001505 chr18 64629148 64629839 + 691 691 -- -- LDM_E 66 0 --

oar_circ_0001506 chr18 64649667 64654187 + 4520 4520 -- -- LDM_E 2 0 --

oar_circ_0001507 chr18 65500458 65506410 + 5952 5649 ENSOARG00000002427; intron_11:65504707-65506337,intron_9:65500629-65501927,intron_10:65502055-65504605,exon_9:65500458-65500629,; LDM_E 3 142 PPP2R5C

oar_circ_0001508 chr18 65559936 65562603 + 2667 2579 ENSOARG00000003064; intron_3:65562123-65562347,intron_2:65560024-65561949,exon_3:65561949-65562123,exon_4:65562347-65562603,; LDM_E,LDM_A 2,6 315,214 DYNC1H1

oar_circ_0001509 chr18 65639973 65640201 + 228 228 -- -- LDM_E,LDM_A 2,2 0,0 --

oar_circ_0001510 chr18 6609858 6628646 + 18788 18198 ENSOARG00000009906; intron_2:6623084-6628180,intron_1:6610058-6622960,exon_1:6609858-6610058,; LDM_E,LDM_A 14,12 349,74 TTC23

oar_circ_0001511 chr18 66131858 66138384 + 6526 6168 ENSOARG00000004898; intron_6:66132053-66137141,exon_9:66138151-66138384,intron_8:66137499-66138151,exon_6:66131858-66132053,; LDM_A 2 101 RCOR1

oar_circ_0001512 chr18 6622960 6628646 + 5686 5096 ENSOARG00000009906; intron_2:6623084-6628180,; LDM_A 5 8 TTC23

oar_circ_0001513 chr18 6628180 6628646 + 466 466 -- -- LDM_E,LDM_A 3,2 1,0 --

oar_circ_0001514 chr18 66371660 66387825 - 16165 15744 ENSOARG00000005283; intron_35:66382286-66387733,intron_33:66371809-66380033,intron_34:66380129-66382202,; LDM_E 2 336 CDC42BPB

oar_circ_0001515 chr18 66676756 66681832 + 5076 4116 ENSOARG00000005628; intron_7:66680502-66680763,intron_2:66677232-66677726,intron_3:66677899-66678164,exon_7:66680340-66680502,exon_8:66680763-66680928,exon_6:66679709-66679868,intron_5:66679448-66679709,intron_8:66680928-66681225,intron_9:66681360-66681738,intron_6:66679868-66680340,intron_4:66678276-66679305,exon_3:66677726-66677899,; LDM_E,LDM_A 4,7 264,505 EIF5

oar_circ_0001516 chr18 66679305 66681832 + 2527 2155 ENSOARG00000005628; intron_8:66680928-66681225,intron_9:66681360-66681738,intron_6:66679868-66680340,intron_5:66679448-66679709,exon_6:66679709-66679868,exon_8:66680763-66680928,intron_7:66680502-66680763,exon_7:66680340-66680502,; LDM_A 2 236 EIF5

oar_circ_0001517 chr18 66679716 66679868 + 152 152 ENSOARG00000005628; exon_6:66679709-66679868,; LDM_A 2 2 EIF5

oar_circ_0001518 chr18 66736431 66736623 + 192 192 ENSOARG00000005700; exon_3:66736431-66736623,; LDM_E,LDM_A 2,3 43,13 MARK3

oar_circ_0001519 chr18 66736431 66753040 + 16609 16555 ENSOARG00000005700; intron_3:66736623-66752986,exon_3:66736431-66736623,; LDM_E,LDM_A 6,6 626,190 MARK3

oar_circ_0001520 chr18 66736431 66789278 + 52847 52607 ENSOARG00000005700; exon_3:66736431-66736623,intron_5:66783183-66785556,intron_4:66753040-66783134,intron_6:66785622-66789207,intron_3:66736623-66752986,; LDM_E 11 3663 MARK3

oar_circ_0001521 chr18 66891387 66899098 + 7711 7559 ENSOARG00000005896; exon_1:66891384-66891585,intron_1:66891585-66892282,intron_2:66892346-66899007,; LDM_E 2 79 APOPT1

oar_circ_0001522 chr18 66945349 66947677 + 2328 2327 ENSOARG00000005991; intron_1:66945611-66947446,exon_1:66945350-66945611,exon_2:66947446-66947677,; LDM_E,LDM_A 14,37 230,216 -

oar_circ_0001523 chr18 66945349 66949293 + 3944 3944 ENSOARG00000005991; exon_1:66945350-66945611,intron_1:66945611-66947446,intron_2:66947677-66952286,exon_2:66947446-66947677,; LDM_E,LDM_A 14,44 302,274 -

oar_circ_0001524 chr18 66945349 66963803 + 18454 16865 ENSOARG00000005991; exon_1:66945350-66945611,intron_1:66945611-66947446,intron_2:66947677-66952286,exon_2:66947446-66947677,intron_5:66952784-66960280,intron_11:66961676-66963694,intron_7:66960712-66961127,; LDM_A 2 373 -

oar_circ_0001525 chr18 66945349 66975896 + 30547 28827 ENSOARG00000005991; intron_2:66947677-66952286,intron_13:66969637-66975765,intron_5:66952784-66960280,intron_12:66963803-66969475,intron_11:66961676-66963694,exon_13:66969475-66969637,exon_1:66945350-66945611,intron_1:66945611-66947446,exon_2:66947446-66947677,intron_7:66960712-66961127,; LDM_E,LDM_A 7,7 1758,1266 -

oar_circ_0001526 chr18 67019289 67021903 - 2614 2512 ENSOARG00000006393; exon_5:67019289-67019464,intron_13:67019464-67021801,; LDM_E 4 33 PPP1R13B

oar_circ_0001527 chr18 67019289 67033848 - 14559 14380 ENSOARG00000006393; intron_14:67021903-67033771,exon_5:67019289-67019464,intron_13:67019464-67021801,; LDM_E 2 229 PPP1R13B

oar_circ_0001528 chr18 67033771 67043689 - 9918 9573 ENSOARG00000006393; intron_15:67033848-67038426,intron_16:67038546-67043541,; LDM_E 5 201 PPP1R13B

oar_circ_0001529 chr18 68256539 68259331 - 2792 1809 ENSOARG00000008460; intron_12:68258172-68259181,intron_11:68257278-68258078,; LDM_A 3 10 BRF1

oar_circ_0001530 chr18 6973772 6974318 + 546 546 -- -- LDM_E,LDM_A 54,52 0,0 --

oar_circ_0001531 chr18 6989137 6989344 - 207 207 -- -- LDM_E 2 0 --

oar_circ_0001532 chr18 964556 968053 + 3497 3275 ENSOARG00000009075; intron_1:964652-967927,; LDM_E,LDM_A 4,5 503,154 -

oar_circ_0001533 chr19 10150628 10169811 + 19183 18745 ENSOARG00000016427; intron_7:10150693-10152199,intron_8:10152288-10169527,; LDM_E 3 166 STAC

oar_circ_0001534 chr19 10691553 10696282 + 4729 4176 ENSOARG00000000094; exon_3:10693073-10693241,exon_4:10694170-10698178,; LDM_E 2 9 DCLK3

oar_circ_0001535 chr19 10757364 10771175 + 13811 13245 ENSOARG00000000286; intron_6:10759870-10766225,exon_9:10768301-10768489,intron_7:10766324-10766672,exon_4:10757364-10757679,intron_5:10758455-10759813,intron_4:10757679-10758407,intron_9:10768489-10768778,intron_10:10768862-10771027,intron_8:10766802-10768301,; LDM_A 2 807 GOLGA4

oar_circ_0001536 chr19 10781353 10792479 + 11126 10997 ENSOARG00000000286; intron_14:10786701-10788235,exon_12:10781353-10781532,intron_13:10783689-10786545,exon_15:10788235-10792479,intron_12:10781532-10783560,exon_14:10786545-10786701,; LDM_A 7 6452 GOLGA4

oar_circ_0001537 chr19 10805227 10812086 + 6859 6592 ENSOARG00000000286; intron_21:10805303-10807340,intron_22:10807444-10811999,; LDM_E,LDM_A 3,39 372,384 GOLGA4

oar_circ_0001538 chr19 10864460 10877338 + 12878 12519 ENSOARG00000000498; intron_2:10864588-10866240,intron_3:10866347-10877214,; LDM_E 2 164 ITGA9

oar_circ_0001539 chr19 11806080 11813323 + 7243 6761 ENSOARG00000001455; intron_3:11809138-11810894,intron_4:11810971-11813208,intron_2:11806230-11808998,; LDM_E 3 116 EXOG

oar_circ_0001540 chr19 11808998 11813323 + 4325 3993 ENSOARG00000001455; intron_4:11810971-11813208,intron_3:11809138-11810894,; LDM_E 2 66 EXOG

oar_circ_0001541 chr19 11983157 11983586 - 429 429 ENSOARG00000001512; intron_31:11977485-11983465,; LDM_E 3 1 SCN5A

oar_circ_0001542 chr19 12312934 12316266 + 3332 2782 ENSOARG00000001873; intron_2:12313075-12315129,intron_3:12315208-12315936,; LDM_E 2 68 WDR48

oar_circ_0001543 chr19 12312934 12318920 + 5986 5245 ENSOARG00000001873; intron_3:12315208-12315936,intron_5:12316266-12318350,intron_2:12313075-12315129,intron_6:12318439-12318818,; LDM_E 6 180 WDR48

oar_circ_0001544 chr19 12318350 12330430 + 12080 11613 ENSOARG00000001873; intron_7:12318920-12324802,intron_8:12325027-12326662,intron_10:12328543-12330332,intron_9:12326737-12328440,exon_8:12324802-12325027,intron_6:12318439-12318818,; LDM_E 3 278 WDR48

oar_circ_0001545 chr19 14461437 14464796 + 3359 3069 ENSOARG00000003229; intron_9:14461512-14462061,intron_10:14462199-14464719,; LDM_E,LDM_A 2,3 85,31 TRAK1

oar_circ_0001546 chr19 14678110 14688944 + 10834 10570 ENSOARG00000003525; intron_6:14687179-14687634,intron_5:14678198-14687149,exon_8:14688721-14688944,intron_7:14687780-14688721,; LDM_A 2 1448 NKTR

oar_circ_0001547 chr19 15165037 15169392 + 4355 589 ENSOARG00000004010; exon_1:15168803-15169392,; LDM_E,LDM_A 19,17 562,404 SNRK

oar_circ_0001548 chr19 15168703 15169392 + 689 589 ENSOARG00000004010; exon_1:15168803-15169392,; LDM_A 7 404 SNRK

oar_circ_0001549 chr19 15205955 15212707 + 6752 6475 ENSOARG00000004010; exon_3:15209619-15209832,intron_3:15209832-15212572,intron_2:15206097-15209619,; LDM_E,LDM_A 3,2 293,207 SNRK

oar_circ_0001550 chr19 15209619 15212707 + 3088 2953 ENSOARG00000004010; exon_3:15209619-15209832,intron_3:15209832-15212572,; LDM_A 2 89 SNRK

oar_circ_0001551 chr19 15427732 15432507 - 4775 4487 ENSOARG00000004094; intron_10:15427867-15428985,intron_11:15429183-15432354,exon_2:15428985-15429183,; LDM_E 4 39 ANO10

oar_circ_0001552 chr19 16171970 16180219 + 8249 7886 ENSOARG00000004419; intron_2:16172043-16177302,intron_3:16177438-16180065,; LDM_E 2 88 TCAIM

oar_circ_0001553 chr19 16426644 16429185 + 2541 1955 ENSOARG00000004780; exon_8:16426644-16426854,intron_8:16426854-16428599,; LDM_E 3 51 KIF15

oar_circ_0001554 chr19 16534553 16536602 - 2049 1921 ENSOARG00000004960; intron_1:16534729-16535147,exon_7:16534553-16534729,intron_2:16535331-16536474,exon_6:16535147-16535331,; LDM_E 5 19 -

oar_circ_0001555 chr19 1674438 1679561 - 5123 4893 ENSOARG00000014224; intron_3:1674536-1679429,; LDM_E 3 101 SLC4A7

oar_circ_0001556 chr19 1679429 1686778 - 7349 7148 ENSOARG00000014224; intron_4:1679561-1685250,intron_5:1685424-1686709,exon_23:1685250-1685424,; LDM_A 3 76 SLC4A7

oar_circ_0001557 chr19 1697849 1711165 - 13316 12822 ENSOARG00000014224; exon_14:1704855-1705030,intron_14:1705030-1707227,intron_15:1707334-1711018,intron_13:1701306-1704855,exon_16:1699758-1700004,intron_11:1697955-1699758,intron_12:1700004-1701172,; LDM_E,LDM_A 10,4 701,239 SLC4A7

oar_circ_0001558 chr19 17041440 17048713 - 7273 7143 ENSOARG00000006977; exon_18:17041440-17041685,intron_6:17041685-17048583,; LDM_A 2 492 SETD5

oar_circ_0001559 chr19 17128836 17147382 - 18546 17594 ENSOARG00000007139; intron_9:17146053-17147093,exon_3:17139870-17140347,exon_1:17147093-17147382,intron_3:17129282-17130029,intron_4:17130140-17131133,intron_7:17137812-17139870,intron_5:17131249-17134731,intron_6:17134801-17137681,intron_8:17140347-17145975,; LDM_E,LDM_A 2,2 362,321 THUMPD3

oar_circ_0001560 chr19 17671860 17683831 + 11971 11971 -- -- LDM_E 2 0 --

oar_circ_0001561 chr19 21096551 21100383 - 3832 3540 ENSOARG00000007760; exon_10:21099625-21099796,intron_3:21096648-21097544,intron_4:21097618-21099625,intron_5:21099796-21100262,; LDM_A 6 106 EDEM1

oar_circ_0001562 chr19 21134355 21136568 - 2213 1964 ENSOARG00000007805; intron_5:21135157-21136487,intron_4:21134449-21135083,; LDM_E,LDM_A 4,3 131,151 ARL8B

oar_circ_0001563 chr19 21308896 21309066 + 170 170 -- -- LDM_A 2 0 --

oar_circ_0001564 chr19 21583981 21584431 - 450 337 ENSOARG00000008132; exon_26:21584265-21584431,exon_27:21583981-21584152,; LDM_E 7 30 ITPR1

oar_circ_0001565 chr19 21709993 21712319 + 2326 2326 -- -- LDM_A 2 0 --

oar_circ_0001566 chr19 21813102 21817134 + 4032 3957 ENSOARG00000008479; exon_2:21813102-21813276,intron_2:21813276-21817059,; LDM_A 3 35 SUMF1

oar_circ_0001567 chr19 21838704 21847680 + 8976 8541 ENSOARG00000008479; intron_6:21842499-21847566,intron_4:21838787-21841537,intron_5:21841660-21842384,; LDM_E 2 152 SUMF1

oar_circ_0001568 chr19 2759798 2791041 + 31243 31118 ENSOARG00000014615; intron_1:2760149-2767626,intron_2:2767786-2790929,exon_1:2759811-2760149,exon_2:2767626-2767786,; LDM_E 10 450 ZCWPW2

oar_circ_0001569 chr19 285085 292767 + 7682 6347 ENSOARG00000013624; exon_7:290025-290177,intron_11:291751-292000,intron_4:287709-288650,intron_2:285141-286937,intron_3:287050-287634,intron_6:289687-290025,intron_8:290348-290751,intron_9:290845-291440,intron_5:288797-289609,intron_12:292097-292574,; LDM_E 2 654 DBNL

oar_circ_0001570 chr19 29890035 29932685 + 42650 42047 ENSOARG00000009622; intron_4:29920675-29924186,exon_6:29927935-29928091,intron_3:29919043-29920614,intron_2:29908861-29918948,intron_5:29924256-29927935,intron_6:29928091-29932527,intron_1:29890181-29908788,; LDM_E 5 1387 EIF4E3

oar_circ_0001571 chr19 29908788 29928091 + 19303 19004 ENSOARG00000009622; intron_4:29920675-29924186,exon_6:29927935-29928091,intron_2:29908861-29918948,intron_5:29924256-29927935,intron_3:29919043-29920614,; LDM_E,LDM_A 3,4 437,232 EIF4E3

oar_circ_0001572 chr19 29932527 29935825 + 3298 3298 -- -- LDM_A 2 0 --

oar_circ_0001573 chr19 30560552 30572866 + 12314 12089 ENSOARG00000009693; exon_6:30567137-30567291,intron_6:30567291-30572661,intron_4:30560690-30561728,exon_7:30572661-30572866,intron_5:30561815-30567137,; LDM_E,LDM_A 5,26 721,301 FOXP1

oar_circ_0001574 chr19 30560552 30599909 + 39357 39027 ENSOARG00000009693; intron_4:30560690-30561728,intron_7:30572866-30599804,intron_6:30567291-30572661,exon_6:30567137-30567291,intron_5:30561815-30567137,exon_7:30572661-30572866,; LDM_A 5 952 FOXP1

oar_circ_0001575 chr19 30567137 30572866 + 5729 5729 ENSOARG00000009693; intron_6:30567291-30572661,exon_7:30572661-30572866,exon_6:30567137-30567291,; LDM_E 6 291 FOXP1

oar_circ_0001576 chr19 32357780 32358592 + 812 812 ENSOARG00000009914; exon_21:32357780-32358592,; LDM_E 5 103 FRMD4B

oar_circ_0001577 chr19 32479868 32484133 + 4265 4040 ENSOARG00000010235; intron_3:32483363-32484006,intron_2:32481061-32483259,exon_2:32479862-32481061,; LDM_A 3 713 TMF1

oar_circ_0001578 chr19 32494200 32506489 + 12289 11250 ENSOARG00000010235; intron_9:32499535-32500245,intron_12:32504775-32505002,exon_11:32503679-32503872,exon_8:32495052-32495209,intron_13:32505095-32505948,exon_15:32506336-32506489,exon_10:32500245-32500402,intron_8:32495209-32499442,exon_7:32494595-32494762,intron_11:32503872-32504705,intron_10:32500402-32503679,intron_7:32494762-32495052,; LDM_A 3 303 TMF1

oar_circ_0001579 chr19 34012035 34027397 + 15362 15122 ENSOARG00000010433; intron_7:34012132-34024960,intron_8:34025122-34027254,exon_8:34024960-34025122,; LDM_E,LDM_A 33,25 721,269 SUCLG2

oar_circ_0001580 chr19 35083860 35084181 + 321 321 ENSOARG00000010549; exon_14:35083860-35084181,; LDM_E,LDM_A 46,10 139,21 LRIG1

oar_circ_0001581 chr19 35202827 35223555 - 20728 20465 ENSOARG00000010656; intron_6:35202875-35204142,intron_7:35204247-35223445,; LDM_E 5 163 SLC25A26

oar_circ_0001582 chr19 35202827 35227047 - 24220 23957 ENSOARG00000010656; intron_7:35204247-35223445,intron_6:35202875-35204142,exon_2:35226890-35227047,intron_8:35223555-35226890,; LDM_E 3 194 SLC25A26

oar_circ_0001583 chr19 35969487 35970790 + 1303 1303 ENSOARG00000010733; intron_11:35969670-35970169,exon_11:35969487-35969670,exon_12:35970169-35970790,; LDM_E 2 120 MAGI1

oar_circ_0001584 chr19 3608442 3620277 + 11835 11835 -- -- LDM_E 2 0 --

oar_circ_0001585 chr19 36811047 36821228 + 10181 9330 ENSOARG00000010962; intron_6:36813874-36814877,exon_4:36811047-36811331,intron_8:36815138-36819518,intron_9:36819665-36821086,intron_4:36811331-36813573,; LDM_E 6 1069 ADAMTS9

oar_circ_0001586 chr19 36821086 36831385 + 10299 9903 ENSOARG00000010962; intron_10:36821228-36825035,intron_11:36825143-36831239,; LDM_E,LDM_A 8,2 568,199 ADAMTS9

oar_circ_0001587 chr19 39371370 39388114 - 16744 16641 ENSOARG00000011877; exon_1:39387934-39388160,intron_3:39371519-39387934,; LDM_A 2 170 -

oar_circ_0001588 chr19 4260864 4290127 + 29263 29263 ENSOARG00000014684; exon_5:4268466-4268624,intron_4:4107104-4268466,intron_5:4268624-4290047,; LDM_E 3 1721 RBMS3

oar_circ_0001589 chr19 42986060 42995052 - 8992 8893 ENSOARG00000012334; exon_3:42986060-42986247,intron_15:42986247-42992975,intron_16:42993023-42995001,; LDM_A 7 69 PXK

oar_circ_0001590 chr19 43226386 43231391 - 5005 4759 ENSOARG00000012724; intron_47:43229069-43231142,intron_46:43226534-43228971,exon_2:43231142-43231391,; LDM_E 2 194 FLNB

oar_circ_0001591 chr19 43406044 43432970 - 26926 26605 ENSOARG00000013142; intron_19:43406107-43407710,intron_21:43414649-43432822,intron_20:43407747-43414576,; LDM_A 3 1179 SLMAP

oar_circ_0001592 chr19 43483719 43483888 - 169 169 ENSOARG00000013142; intron_22:43432970-43498429,; LDM_E 2 2 SLMAP

oar_circ_0001593 chr19 43629121 43636485 + 7364 6975 ENSOARG00000013565; intron_3:43629777-43634852,intron_4:43634924-43636359,intron_2:43629202-43629667,; LDM_E 2 639 ARF4

oar_circ_0001594 chr19 43630789 43631083 - 294 294 -- -- LDM_A 2 0 --

oar_circ_0001595 chr19 43991190 43994885 + 3695 3311 ENSOARG00000014676; intron_9:43993868-43994700,exon_10:43994700-43994885,intron_7:43992656-43993208,intron_6:43991342-43992590,intron_8:43993263-43993757,; LDM_E 16 111 IL17RD

oar_circ_0001596 chr19 44357492 44397048 + 39556 37742 ENSOARG00000015004; exon_18:44389644-44390576,intron_13:44376488-44377916,exon_15:44382156-44382563,intron_15:44382563-44383005,intron_21:44395330-44396524,intron_18:44390576-44393488,intron_20:44394775-44395221,exon_20:44394615-44394775,intron_2:44357638-44359692,exon_14:44377916-44378615,exon_6:44361701-44361863,exon_22:44396524-44396703,intron_11:44366508-44376243,intron_3:44359785-44360570,intron_17:44384649-44389644,exon_23:44396883-44397048,intron_16:44383147-44384589,intron_8:44364570-44365313,intron_5:44361242-44361701,intron_4:44360643-44361150,intron_9:44365419-44366220,intron_19:44393611-44394615,intron_14:44378615-44382156,intron_7:44363293-44364538,intron_6:44361863-44363168,; LDM_E 9 3485 FAM208A

oar_circ_0001597 chr19 44357492 44397767 + 40275 38532 ENSOARG00000015004; intron_8:44364570-44365313,intron_16:44383147-44384589,intron_17:44384649-44389644,exon_23:44396883-44397048,intron_4:44360643-44361150,intron_5:44361242-44361701,intron_19:44393611-44394615,intron_9:44365419-44366220,intron_7:44363293-44364538,intron_6:44361863-44363168,intron_23:44397048-44397838,intron_14:44378615-44382156,intron_21:44395330-44396524,exon_15:44382156-44382563,intron_15:44382563-44383005,intron_13:44376488-44377916,exon_18:44389644-44390576,intron_18:44390576-44393488,intron_20:44394775-44395221,exon_14:44377916-44378615,intron_2:44357638-44359692,exon_20:44394615-44394775,intron_11:44366508-44376243,exon_22:44396524-44396703,intron_3:44359785-44360570,exon_6:44361701-44361863,; LDM_E 23 3579 FAM208A

oar_circ_0001598 chr19 44357492 44399866 + 42374 39071 ENSOARG00000015004; intron_5:44361242-44361701,intron_4:44360643-44361150,intron_8:44364570-44365313,intron_17:44384649-44389644,exon_23:44396883-44397048,exon_24:44397838-44398377,intron_16:44383147-44384589,intron_14:44378615-44382156,intron_6:44361863-44363168,intron_7:44363293-44364538,intron_23:44397048-44397838,intron_19:44393611-44394615,intron_9:44365419-44366220,intron_18:44390576-44393488,intron_20:44394775-44395221,intron_21:44395330-44396524,intron_13:44376488-44377916,exon_18:44389644-44390576,exon_15:44382156-44382563,intron_15:44382563-44383005,exon_6:44361701-44361863,intron_11:44366508-44376243,exon_22:44396524-44396703,intron_3:44359785-44360570,exon_14:44377916-44378615,exon_20:44394615-44394775,intron_2:44357638-44359692,; LDM_E 5 3912 FAM208A

oar_circ_0001599 chr19 44364538 44383147 + 18609 17796 ENSOARG00000015004; intron_11:44366508-44376243,intron_14:44378615-44382156,intron_9:44365419-44366220,exon_14:44377916-44378615,intron_13:44376488-44377916,intron_15:44382563-44383005,exon_15:44382156-44382563,intron_8:44364570-44365313,; LDM_E 4 1821 FAM208A

oar_circ_0001600 chr19 44367172 44367424 + 252 252 ENSOARG00000015004; intron_11:44366508-44376243,; LDM_A 2 6 FAM208A

oar_circ_0001601 chr19 44433340 44436072 - 2732 2732 ENSOARG00000015275; intron_13:44434519-44435968,intron_11:44413030-44434397,; LDM_E 2 31 CCDC66

oar_circ_0001602 chr19 44433340 44439874 - 6534 6534 ENSOARG00000015275; intron_14:44436072-44437645,exon_4:44439432-44439874,intron_13:44434519-44435968,intron_11:44413030-44434397,exon_5:44437645-44437811,intron_15:44437811-44439432,; LDM_E,LDM_A 11,9 212,89 CCDC66

oar_circ_0001603 chr19 47665007 47667171 + 2164 1911 ENSOARG00000016637; intron_5:47665146-47667057,; LDM_E 2 39 DCP1A

oar_circ_0001604 chr19 47923692 47932306 + 8614 8519 ENSOARG00000000458; intron_2:47923847-47932211,exon_2:47923692-47923847,; LDM_A 3 126 SFMBT1

oar_circ_0001605 chr19 48038805 48043722 + 4917 3965 ENSOARG00000000729; intron_7:48043089-48043346,intron_6:48042310-48042972,exon_8:48043346-48043515,intron_2:48038972-48041036,exon_2:48038805-48038972,exon_4:48041286-48041449,intron_5:48041698-48042181,; LDM_A 2 76 ITIH4

oar_circ_0001606 chr19 48119068 48119849 + 781 631 ENSOARG00000001955; exon_8:48119699-48119849,intron_7:48119218-48119699,; LDM_A 2 3 NEK4

oar_circ_0001607 chr19 48140672 48142894 + 2222 2123 ENSOARG00000002179; intron_2:48140771-48142680,exon_3:48142680-48142894,; LDM_E,LDM_A 9,9 55,22 GLT8D1

oar_circ_0001608 chr19 48140672 48142962 + 2290 2123 ENSOARG00000002179; intron_2:48140771-48142680,exon_3:48142680-48142894,; LDM_E 4 55 GLT8D1

oar_circ_0001609 chr19 48144397 48146668 + 2271 1370 ENSOARG00000002179; intron_4:48144515-48144738,exon_9:48146242-48146433,exon_7:48145725-48145892,intron_5:48144823-48145304,intron_6:48145417-48145725,; LDM_E 6 40 GLT8D1

oar_circ_0001610 chr19 48156927 48160205 + 3278 2889 ENSOARG00000002751; intron_2:48157065-48159025,intron_3:48159178-48160107,; LDM_A 3 35 PBRM1

oar_circ_0001611 chr19 48159025 48172212 + 13187 12797 ENSOARG00000002751; intron_3:48159178-48160107,intron_5:48166695-48172068,intron_4:48160205-48166547,exon_3:48159025-48159178,; LDM_E 2 298 PBRM1

oar_circ_0001612 chr19 48176263 48177460 + 1197 1029 ENSOARG00000002751; intron_8:48176332-48177361,; LDM_E 4 37 PBRM1

oar_circ_0001613 chr19 48663740 48664906 - 1166 1016 ENSOARG00000006209; exon_3:48664657-48664906,intron_8:48663890-48664657,; LDM_E,LDM_A 4,2 47,53 ALAS1

oar_circ_0001614 chr19 49260403 49265089 + 4686 4421 ENSOARG00000007546; intron_3:49260480-49262601,intron_4:49262675-49264975,; LDM_A 2 66 VPRBP

oar_circ_0001615 chr19 50121532 50121918 - 386 386 ENSOARG00000010649; intron_13:50120263-50121825,; LDM_E 2 5 SEMA3F

oar_circ_0001616 chr19 50121546 50121918 - 372 372 ENSOARG00000010649; intron_13:50120263-50121825,; LDM_E,LDM_A 4,28 4,8 SEMA3F

oar_circ_0001617 chr19 50173822 50180027 - 6205 5218 ENSOARG00000010923; intron_8:50174866-50176493,intron_15:50179416-50179866,intron_7:50174126-50174704,exon_9:50179866-50180027,intron_10:50176875-50177263,intron_14:50178706-50179318,intron_13:50178456-50178618,intron_11:50177349-50178222,exon_17:50174704-50174866,intron_9:50176585-50176790,; LDM_E,LDM_A 28,15 479,260 RBM5

oar_circ_0001618 chr19 50174704 50180027 - 5323 4640 ENSOARG00000010923; intron_10:50176875-50177263,exon_9:50179866-50180027,intron_14:50178706-50179318,intron_8:50174866-50176493,intron_15:50179416-50179866,intron_9:50176585-50176790,intron_11:50177349-50178222,intron_13:50178456-50178618,exon_17:50174704-50174866,; LDM_E,LDM_A 5,3 459,240 RBM5

oar_circ_0001619 chr19 50175502 50175793 + 291 291 -- -- LDM_A 2 0 --

oar_circ_0001620 chr19 50257298 50261940 - 4642 4482 ENSOARG00000011125; exon_3:50260661-50261940,intron_18:50259002-50260661,intron_17:50257368-50258912,; LDM_E 3 753 RBM6

oar_circ_0001621 chr19 50574065 50574468 - 403 403 ENSOARG00000013645; exon_1:50574065-50574468,; LDM_A 6 357 DAG1

oar_circ_0001622 chr19 5059750 5064413 + 4663 4663 ENSOARG00000014726; exon_1:5059653-5059919,intron_1:5059919-5064222,exon_2:5064222-5064413,; LDM_E,LDM_A 30,14 184,115 TGFBR2

oar_circ_0001623 chr19 50709988 50731692 + 21704 20722 ENSOARG00000013960; intron_8:50727171-50728315,intron_3:50714289-50715651,intron_4:50715778-50716364,exon_11:50731467-50731692,intron_6:50716701-50726008,intron_2:50710116-50714158,exon_9:50728315-50728489,exon_10:50730186-50730345,intron_9:50728489-50730186,intron_10:50730345-50731467,intron_7:50726149-50727053,; LDM_E 8 782 USP4

oar_circ_0001624 chr19 51282630 51283539 + 909 762 ENSOARG00000017897; intron_8:51282838-51283392,exon_8:51282630-51282838,; LDM_A 2 94 PFKFB4

oar_circ_0001625 chr19 516133 521799 + 5666 5451 ENSOARG00000013649; intron_1:516211-521662,; LDM_E 2 108 VOPP1

oar_circ_0001626 chr19 51757669 51790193 + 32524 32218 ENSOARG00000002647; intron_2:51757738-51787488,intron_3:51787611-51790079,; LDM_E 5 3448 MAP4

oar_circ_0001627 chr19 51787488 51795253 + 7765 7273 ENSOARG00000002647; intron_6:51794408-51795133,intron_5:51792765-51793343,intron_4:51790193-51792630,exon_6:51793343-51794408,intron_3:51787611-51790079,; LDM_E,LDM_A 2,2 2569,2387 MAP4

oar_circ_0001628 chr19 51922160 51930418 + 8258 7991 ENSOARG00000003267; intron_6:51922225-51925469,intron_7:51925545-51930292,; LDM_E 2 220 SMARCC1

oar_circ_0001629 chr19 51944302 51958907 + 14605 13969 ENSOARG00000003267; exon_19:51958648-51958907,intron_13:51944424-51947697,intron_14:51947769-51948475,intron_17:51950296-51952137,intron_15:51948589-51949139,intron_16:51949293-51950182,intron_18:51952197-51958648,; LDM_E 2 987 SMARCC1

oar_circ_0001630 chr19 51947697 51959771 + 12074 11560 ENSOARG00000003267; intron_18:51952197-51958648,intron_17:51950296-51952137,intron_15:51948589-51949139,intron_14:51947769-51948475,intron_19:51958907-51959609,exon_19:51958648-51958907,intron_16:51949293-51950182,exon_20:51959609-51959771,; LDM_E 3 776 SMARCC1

oar_circ_0001631 chr19 51966975 51970976 + 4001 4001 ENSOARG00000003267; intron_20:51959771-51977596,; LDM_E 2 228 SMARCC1

oar_circ_0001632 chr19 51985762 51997039 + 11277 11142 ENSOARG00000003267; exon_25:51996777-51997039,intron_24:51985897-51996777,; LDM_E 2 638 SMARCC1

oar_circ_0001633 chr19 52333150 52337705 - 4555 4555 -- -- LDM_E 2 0 --

oar_circ_0001634 chr19 52399148 52402412 + 3264 3009 ENSOARG00000005054; exon_11:52401749-52402412,intron_10:52400134-52401749,intron_9:52399283-52400014,; LDM_E 4 338 SETD2

oar_circ_0001635 chr19 5281642 5299616 - 17974 17291 ENSOARG00000014735; exon_1:5299443-5299616,intron_11:5288457-5291784,intron_10:5287669-5288350,intron_13:5294754-5299443,intron_9:5281898-5287553,intron_12:5291875-5294641,; LDM_A 2 465 GADL1

oar_circ_0001636 chr19 52830332 52836620 + 6288 5868 ENSOARG00000008245; intron_5:52832226-52833825,intron_4:52830469-52832080,exon_6:52833825-52833981,intron_6:52833981-52836483,; LDM_A 2 530 LRRC2

oar_circ_0001637 chr19 53300743 53305972 + 5229 5133 ENSOARG00000009139; intron_18:53300839-53305761,exon_19:53305761-53305972,; LDM_E,LDM_A 6,9 72,164 FYCO1

oar_circ_0001638 chr19 54127208 54139069 + 11861 11736 ENSOARG00000011364; exon_1:54127208-54127538,intron_1:54127538-54138944,; LDM_A 3 42 ZDHHC3

oar_circ_0001639 chr19 55495812 55506139 - 10327 1084 ENSOARG00000012867; exon_2:55495812-55496002,intron_8:55496002-55496896,; LDM_E 2 43 VGLL4

oar_circ_0001640 chr19 55689506 55690994 - 1488 1425 ENSOARG00000013015; exon_2:55690828-55690994,intron_2:55689569-55690828,; LDM_E 3 19 -

oar_circ_0001641 chr19 55811551 55817072 + 5521 5521 -- -- LDM_E,LDM_A 4,3 0,0 --

oar_circ_0001642 chr19 55811551 55854020 + 42469 573 ENSOARG00000013199; exon_1:55853431-55853832,exon_2:55853848-55854020,; LDM_E 5 81 TMCC1

oar_circ_0001643 chr19 55816827 55854020 + 37193 573 ENSOARG00000013199; exon_1:55853431-55853832,exon_2:55853848-55854020,; LDM_E 9 81 TMCC1

oar_circ_0001644 chr19 55849378 55854020 + 4642 573 ENSOARG00000013199; exon_1:55853431-55853832,exon_2:55853848-55854020,; LDM_E 11 81 TMCC1

oar_circ_0001645 chr19 55939600 55940535 + 935 935 ENSOARG00000013199; exon_3:55939600-55940535,; LDM_E,LDM_A 6,2 235,540 TMCC1

oar_circ_0001646 chr19 56206848 56207043 - 195 195 -- -- LDM_A 2 0 --

oar_circ_0001647 chr19 56405906 56412585 - 6679 6439 ENSOARG00000015759; intron_3:56406017-56408482,intron_5:56410406-56411394,exon_4:56410101-56410406,intron_6:56411576-56412456,intron_4:56408697-56410101,exon_3:56411394-56411576,exon_5:56408482-56408697,; LDM_A 2 213 MKRN2

oar_circ_0001648 chr19 57090114 57091743 + 1629 1629 ENSOARG00000017225; intron_2:57090221-57100053,; LDM_A 4 15 MRPS25

oar_circ_0001649 chr19 57584039 57584467 + 428 170 ENSOARG00000018568; exon_8:57584297-57584467,; LDM_E,LDM_A 25,7 5,9 GRIP2

oar_circ_0001650 chr19 57655647 57665143 - 9496 9363 ENSOARG00000000248; intron_14:57655780-57664908,exon_4:57664908-57665143,; LDM_A 3 16 SLC6A6

oar_circ_0001651 chr19 58335709 58349905 + 14196 11962 ENSOARG00000001964; intron_1:58335846-58337815,intron_3:58339064-58343401,intron_8:58346718-58348704,intron_9:58348756-58349772,intron_7:58344046-58346700,; LDM_A 4 11 NUP210

oar_circ_0001652 chr19 58686186 58700847 + 14661 7941 ENSOARG00000002529;ENSOARG00000026667; exon_3:58693328-58694608,intron_2:58691722-58693328,intron_1:58687852-58691560,exon_1:58687597-58687852,;exon_2:58686570-58686814,exon_1:58685722-58686414,intron_1:58686414-58686570,; LDM_A 2 62 -;--

oar_circ_0001653 chr19 58827594 58827804 + 210 210 -- -- LDM_A 2 0 --

oar_circ_0001654 chr19 58827616 58827826 - 210 210 -- -- LDM_A 2 0 --

oar_circ_0001655 chr19 58865536 58865895 - 359 359 -- -- LDM_A 2 0 --

oar_circ_0001656 chr19 58931238 58933677 + 2439 976 ENSOARG00000003751; intron_7:58932701-58933519,exon_8:58933519-58933677,; LDM_E,LDM_A 100,98 14,3 COPG1

oar_circ_0001657 chr19 59224896 59225941 - 1045 948 ENSOARG00000004600; exon_4:59224896-59225061,intron_2:59225061-59225844,; LDM_E,LDM_A 42,30 6,5 EEFSEC

oar_circ_0001658 chr19 59224896 59234085 - 9189 9092 ENSOARG00000004600; exon_4:59224896-59225061,intron_2:59225061-59225844,exon_2:59233877-59234085,intron_3:59225941-59233877,; LDM_E,LDM_A 14,8 52,34 EEFSEC

oar_circ_0001659 chr19 59405582 59407253 - 1671 1671 ENSOARG00000005461; exon_2:59406075-59407253,intron_4:59401885-59406075,; LDM_A 2 1514 KBTBD12

oar_circ_0001660 chr19 59406075 59407253 - 1178 1178 ENSOARG00000005461; exon_2:59406075-59407253,; LDM_E,LDM_A 9,171 107,1509 KBTBD12

oar_circ_0001661 chr19 59601759 59610496 + 8737 8399 ENSOARG00000005578; intron_3:59601896-59603300,intron_4:59603411-59610406,; LDM_A 2 69 MGLL

oar_circ_0001662 chr19 59835505 59836569 - 1064 788 ENSOARG00000006991; intron_11:59835987-59836775,; LDM_E 7 3 -

oar_circ_0001663 chr19 6010920 6021427 + 10507 3869 ENSOARG00000014854; exon_3:6021139-6021427,intron_2:6017558-6021139,; LDM_E 2 437 STT3B

oar_circ_0001664 chr19 6017449 6021427 + 3978 3869 ENSOARG00000014854; exon_3:6021139-6021427,intron_2:6017558-6021139,; LDM_E,LDM_A 26,10 437,140 STT3B

oar_circ_0001665 chr19 60283829 60288883 - 5054 4730 ENSOARG00000007886; intron_3:60283946-60285516,intron_4:60285614-60288774,; LDM_E 5 16 -

oar_circ_0001666 chr19 6156718 6170314 - 13596 13435 ENSOARG00000014924; exon_4:6170103-6170314,intron_2:6156879-6170103,; LDM_A 4 32 OSBPL10

oar_circ_0001667 chr19 6259216 6260013 - 797 797 ENSOARG00000014924; intron_3:6170314-6259895,; LDM_A 2 2 OSBPL10

oar_circ_0001668 chr19 6887540 6893474 - 5934 5586 ENSOARG00000015181; intron_10:6891053-6893304,exon_3:6893304-6893474,intron_6:6887652-6888354,intron_9:6890219-6890940,intron_7:6888430-6889297,intron_8:6889309-6890184,; LDM_E 9 325 DYNC1LI1

oar_circ_0001669 chr19 7326934 7330771 - 3837 3142 ENSOARG00000015477; exon_2:7330601-7330771,intron_12:7327029-7328211,exon_3:7328811-7328962,intron_14:7328962-7330601,; LDM_A 3 12 GLB1

oar_circ_0001670 chr19 7535828 7544766 + 8938 8938 ENSOARG00000015767; intron_6:7538299-7544671,intron_5:7537897-7538224,intron_4:7535445-7537842,; LDM_A 2 81 FBXL2

oar_circ_0001671 chr19 7601070 7627261 - 26191 25749 ENSOARG00000015881; intron_13:7601177-7601590,intron_15:7605174-7627109,intron_14:7601696-7605097,; LDM_E 3 2430 UBP1

oar_circ_0001672 chr19 7675357 7679352 - 3995 2903 ENSOARG00000016028; exon_35:7675357-7675564,exon_33:7679158-7679352,intron_3:7675564-7678066,; LDM_A 2 68 CLASP2

oar_circ_0001673 chr19 7686712 7692504 - 5792 5685 ENSOARG00000016028; intron_7:7690536-7692397,exon_32:7686712-7686941,intron_6:7686941-7690382,exon_31:7690382-7690536,; LDM_E,LDM_A 2,2 240,146 CLASP2

oar_circ_0001674 chr19 7686712 7699995 - 13283 12888 ENSOARG00000016028; intron_7:7690536-7692397,exon_32:7686712-7686941,intron_6:7686941-7690382,exon_31:7690382-7690536,intron_9:7695489-7699851,intron_8:7692504-7695345,; LDM_E,LDM_A 2,3 559,264 CLASP2

oar_circ_0001675 chr19 7757765 7762182 - 4417 4208 ENSOARG00000016028; intron_24:7759988-7762111,intron_23:7758677-7759928,exon_16:7757765-7757933,intron_22:7757933-7758599,; LDM_E,LDM_A 2,3 184,61 CLASP2

oar_circ_0001676 chr19 7769608 7778982 - 9374 8919 ENSOARG00000016028; intron_26:7769744-7772550,intron_28:7776251-7778902,intron_27:7772696-7776158,; LDM_E 3 222 CLASP2

oar_circ_0001677 chr19 7827275 7839841 - 12566 12117 ENSOARG00000016028; intron_35:7832080-7839762,intron_34:7830283-7831976,intron_33:7828980-7830191,intron_32:7827373-7828904,; LDM_E,LDM_A 3,2 243,119 CLASP2

oar_circ_0001678 chr19 7969285 7986387 + 17102 16632 ENSOARG00000016185; intron_9:7969413-7971621,intron_11:7973027-7975318,intron_13:7984977-7986263,exon_10:7971621-7971775,intron_12:7975435-7984754,exon_13:7984754-7984977,intron_10:7971775-7972926,; LDM_A 2 935 PDCD6IP

oar_circ_0001679 chr19 9521055 9521256 - 201 201 -- -- LDM_A 2 0 --

oar_circ_0001680 chr19 9567083 9578030 + 10947 8591 ENSOARG00000016371; intron_2:9570661-9571436,intron_3:9571526-9575577,exon_1:9569023-9569190,intron_4:9575722-9577240,intron_1:9569190-9570619,intron_5:9577319-9577970,; LDM_E 2 628 ARPP21

oar_circ_0001681 chr19 9567083 9578895 + 11812 9315 ENSOARG00000016371; exon_1:9569023-9569190,intron_6:9578030-9578754,intron_1:9569190-9570619,intron_5:9577319-9577970,intron_2:9570661-9571436,intron_3:9571526-9575577,intron_4:9575722-9577240,; LDM_E,LDM_A 14,18 714,1841 ARPP21

oar_circ_0001682 chr19 9567083 9594043 + 26960 26960 ENSOARG00000016371; intron_7:9578895-9592016,intron_4:9575722-9577240,intron_3:9571526-9575577,intron_8:9592125-9600256,intron_2:9570661-9571436,intron_5:9577319-9577970,intron_1:9569190-9570619,intron_6:9578030-9578754,exon_1:9569023-9569190,; LDM_E 4 2241 ARPP21

oar_circ_0001683 chr19 9606211 9615150 + 8939 8939 ENSOARG00000016371; exon_12:9606211-9606441,exon_13:9614916-9615150,intron_12:9606441-9614916,; LDM_A 2 693 ARPP21

oar_circ_0001684 chr19 9681768 9683379 + 1611 1400 ENSOARG00000016371; intron_17:9681917-9683059,exon_18:9683059-9683317,; LDM_E,LDM_A 5,2 47,84 ARPP21

oar_circ_0001685 chr1 100282086 100283344 - 1258 974 ENSOARG00000021007; intron_17:100282245-100283219,; LDM_E,LDM_A 2,8 32,8 POGZ

oar_circ_0001686 chr1 100464120 100464824 - 704 704 -- -- LDM_A 2 0 --

oar_circ_0001687 chr1 100480325 100514492 + 34167 33918 ENSOARG00000021022; intron_5:100501928-100503995,intron_4:100500302-100501863,exon_8:100514328-100514492,exon_4:100500109-100500302,exon_3:100480325-100480557,intron_3:100480557-100500109,intron_7:100507144-100514328,intron_6:100504100-100507065,; LDM_A 5 334 SNX27

oar_circ_0001688 chr1 100500109 100507144 + 7035 6786 ENSOARG00000021022; intron_6:100504100-100507065,intron_4:100500302-100501863,intron_5:100501928-100503995,exon_4:100500109-100500302,; LDM_A 5 107 SNX27

oar_circ_0001689 chr1 100500109 100514492 + 14383 14134 ENSOARG00000021022; intron_5:100501928-100503995,intron_6:100504100-100507065,intron_4:100500302-100501863,intron_7:100507144-100514328,exon_8:100514328-100514492,exon_4:100500109-100500302,; LDM_E,LDM_A 5,5 344,139 SNX27

oar_circ_0001690 chr1 100500109 100524742 + 24633 24294 ENSOARG00000021022; intron_7:100507144-100514328,intron_6:100504100-100507065,exon_4:100500109-100500302,intron_4:100500302-100501863,intron_5:100501928-100503995,exon_8:100514328-100514492,intron_8:100514492-100524652,; LDM_E 3 6026 SNX27

oar_circ_0001691 chr1 100503995 100514492 + 10497 10313 ENSOARG00000021022; exon_8:100514328-100514492,intron_7:100507144-100514328,intron_6:100504100-100507065,; LDM_E 6 197 SNX27

oar_circ_0001692 chr1 100531141 100532173 + 1032 321 ENSOARG00000021022; intron_10:100531291-100531612,; LDM_A 3 2 SNX27

oar_circ_0001693 chr1 10099322 10102102 + 2780 2718 ENSOARG00000019470; intron_3:10099847-10101518,exon_3:10099322-10099847,exon_5:10101931-10102102,intron_4:10101580-10101931,; LDM_E,LDM_A 19,64 493,193 ZMYM4

oar_circ_0001694 chr1 10099322 10114068 + 14746 14487 ENSOARG00000019470; exon_8:10113568-10113743,intron_4:10101580-10101931,exon_5:10101931-10102102,intron_6:10109010-10109246,intron_7:10109502-10113568,exon_9:10113855-10114068,intron_3:10099847-10101518,exon_7:10109246-10109502,intron_5:10102102-10108925,exon_3:10099322-10099847,; LDM_E,LDM_A 19,4 1874,517 ZMYM4

oar_circ_0001695 chr1 10145929 10158759 + 12830 12436 ENSOARG00000019470; exon_28:10158510-10158759,intron_26:10148759-10156936,exon_26:10148607-10148759,intron_27:10157048-10158510,intron_25:10146383-10148607,exon_24:10145929-10146101,; LDM_E,LDM_A 45,13 600,146 ZMYM4

oar_circ_0001696 chr1 10156936 10158759 + 1823 1711 ENSOARG00000019470; intron_27:10157048-10158510,exon_28:10158510-10158759,; LDM_E,LDM_A 9,3 88,21 ZMYM4

oar_circ_0001697 chr1 10222193 10227405 - 5212 4986 ENSOARG00000019494; intron_12:10222422-10225424,intron_13:10225557-10227312,exon_10:10222193-10222422,; LDM_E 2 330 KIAA0319L

oar_circ_0001698 chr1 10237506 10240349 - 2843 2741 ENSOARG00000019494; exon_4:10240105-10240349,intron_17:10237608-10240105,; LDM_E,LDM_A 4,5 65,38 KIAA0319L

oar_circ_0001699 chr1 10263654 10264166 - 512 512 ENSOARG00000019494; exon_3:10263654-10264166,; LDM_E 3 126 KIAA0319L

oar_circ_0001700 chr1 103412376 103417541 - 5165 4761 ENSOARG00000002952; exon_1:103416090-103417556,exon_5:103412376-103412567,intron_11:103412837-103413408,intron_12:103413557-103414035,intron_13:103414219-103416090,exon_2:103414035-103414219,; LDM_E 2 612 ADAR

oar_circ_0001701 chr1 104065023 104066530 + 1507 1048 ENSOARG00000004341; intron_5:104065146-104065520,intron_7:104065778-104066452,; LDM_E 2 24 RUSC1

oar_circ_0001702 chr1 104120523 104120992 - 469 207 ENSOARG00000004448; exon_12:104120785-104120992,; LDM_E 2 22 ASH1L

oar_circ_0001703 chr1 104175906 104201083 - 25177 25075 ENSOARG00000004448; exon_7:104175906-104176086,intron_24:104184998-104200981,exon_6:104184253-104184998,intron_23:104176086-104184253,; LDM_E 4 1688 ASH1L

oar_circ_0001704 chr1 104184253 104184998 - 745 745 ENSOARG00000004448; exon_6:104184253-104184998,; LDM_E 3 370 ASH1L

oar_circ_0001705 chr1 104184253 104201083 - 16830 16728 ENSOARG00000004448; exon_6:104184253-104184998,intron_24:104184998-104200981,; LDM_E,LDM_A 4,9 1303,646 ASH1L

oar_circ_0001706 chr1 104200981 104256059 - 55078 54976 ENSOARG00000004448; exon_4:104218736-104223300,exon_3:104255538-104256059,intron_26:104223300-104255538,intron_25:104201083-104218736,; LDM_A 6 4468 ASH1L

oar_circ_0001707 chr1 104218736 104256059 - 37323 37323 ENSOARG00000004448; intron_26:104223300-104255538,exon_4:104218736-104223300,exon_3:104255538-104256059,; LDM_E 6 6240 ASH1L

oar_circ_0001708 chr1 104255538 104256059 - 521 521 ENSOARG00000004448; exon_3:104255538-104256059,; LDM_E,LDM_A 26,8 472,356 ASH1L

oar_circ_0001709 chr1 104255538 104270542 - 15004 14850 ENSOARG00000004448; exon_3:104255538-104256059,intron_27:104256059-104270388,; LDM_E,LDM_A 9,7 1935,1022 ASH1L

oar_circ_0001710 chr1 104356256 104444527 - 88271 61962 ENSOARG00000004747; intron_30:104392642-104413044,intron_20:104364335-104364939,intron_26:104388451-104389079,intron_22:104372835-104381157,intron_31:104413236-104418886,intron_25:104387305-104388355,intron_24:104386593-104387185,intron_27:104389130-104391432,intron_21:104364980-104372733,intron_19:104361493-104364231,intron_29:104391723-104392448,exon_9:104386422-104386593,intron_15:104356407-104357586,exon_2:104413044-104413236,exon_14:104361294-104361493,intron_16:104357731-104359474,intron_18:104360769-104361294,exon_10:104381157-104381350,intron_23:104381350-104386422,exon_3:104392448-104392642,intron_17:104359646-104360682,exon_1:104418886-104419406,exon_16:104359474-104359646,; LDM_E 15 1679 -

oar_circ_0001711 chr1 104418886 104458727 - 39841 520 ENSOARG00000004747; exon_1:104418886-104419406,; LDM_E,LDM_A 5,4 76,70 -

oar_circ_0001712 chr1 104419208 104473769 + 54561 54561 -- -- LDM_E 12 0 --

oar_circ_0001713 chr1 104421145 104473967 - 52822 52822 -- -- LDM_E,LDM_A 18,7 0,0 --

oar_circ_0001714 chr1 105028982 105029989 - 1007 816 ENSOARG00000005974; intron_11:105029186-105029798,exon_2:105028982-105029186,; LDM_A 2 61 MEF2D

oar_circ_0001715 chr1 105206256 105206466 - 210 210 ENSOARG00000006200; exon_6:105204576-105206856,; LDM_E 4 469 NES

oar_circ_0001716 chr1 105206900 105207155 + 255 255 -- -- LDM_E 3 0 --

oar_circ_0001717 chr1 105206935 105207097 - 162 162 ENSOARG00000006200; intron_1:105206856-105207369,; LDM_E 12 96 NES

oar_circ_0001718 chr1 105206935 105207316 - 381 381 ENSOARG00000006200; intron_1:105206856-105207369,; LDM_E 2 1597 NES

oar_circ_0001719 chr1 105207155 105207347 + 192 192 -- -- LDM_E 4 0 --

oar_circ_0001720 chr1 105207155 105207413 + 258 258 -- -- LDM_E 3 0 --

oar_circ_0001721 chr1 105207188 105207413 + 225 225 -- -- LDM_E 2 0 --

oar_circ_0001722 chr1 105207316 105207475 - 159 159 -- -- LDM_E 4 0 --

oar_circ_0001723 chr1 105207316 105207535 - 219 150 ENSOARG00000006200; exon_5:105207369-105207519,; LDM_E 3 5 NES

oar_circ_0001724 chr1 105207316 105207628 - 312 312 -- -- LDM_A 2 0 --

oar_circ_0001725 chr1 105207382 105207724 - 342 342 -- -- LDM_E 2 0 --

oar_circ_0001726 chr1 105207440 105207659 + 219 219 -- -- LDM_E 2 0 --

oar_circ_0001727 chr1 105207473 105207659 + 186 186 -- -- LDM_E 8 0 --

oar_circ_0001728 chr1 105207475 105207724 - 249 249 -- -- LDM_E 3 0 --

oar_circ_0001729 chr1 105207506 105207659 + 153 153 -- -- LDM_E 6 0 --

oar_circ_0001730 chr1 105207535 105207691 - 156 156 -- -- LDM_E 2 0 --

oar_circ_0001731 chr1 105208051 105208213 - 162 162 ENSOARG00000006200; exon_4:105207546-105209072,; LDM_E 10 137 NES

oar_circ_0001732 chr1 105208051 105208312 - 261 261 ENSOARG00000006200; exon_4:105207546-105209072,; LDM_E 2 1127 NES

oar_circ_0001733 chr1 105280539 105280763 - 224 224 ENSOARG00000006368; exon_4:105280539-105280763,; LDM_A 2 74 HDGF

oar_circ_0001734 chr1 10545722 10546257 + 535 200 ENSOARG00000019568; exon_16:10546053-10546253,; LDM_A 5 16 AGO4

oar_circ_0001735 chr1 10671649 10678568 + 6919 6661 ENSOARG00000019609; exon_4:10677273-10677482,intron_3:10671770-10677273,intron_4:10677482-10678431,; LDM_A 2 75 AGO3

oar_circ_0001736 chr1 10732897 10736190 + 3293 2786 ENSOARG00000019609; intron_16:10733497-10736088,exon_15:10732897-10733092,; LDM_A 2 55 AGO3

oar_circ_0001737 chr1 109525841 109531096 + 5255 5255 ENSOARG00000008386; intron_14:109530505-109530791,intron_13:109529997-109530354,intron_12:109523921-109529860,; LDM_E 2 60 ATP1A4

oar_circ_0001738 chr1 10957209 10957506 + 297 297 -- -- LDM_E 3 0 --

oar_circ_0001739 chr1 110719063 110719423 + 360 360 -- -- LDM_E 2 0 --

oar_circ_0001740 chr1 112393558 112396468 + 2910 2910 ENSOARG00000010852; exon_16:112396318-112396468,intron_15:112394448-112396318,exon_14:112393558-112393750,exon_15:112394213-112394448,intron_14:112393750-112394213,; LDM_E 4 152 DDR2

oar_circ_0001741 chr1 113098396 113113615 + 15219 14691 ENSOARG00000011189; intron_3:113101078-113101573,intron_5:113112379-113113541,intron_4:113101635-113112281,intron_2:113100066-113101001,intron_1:113098538-113099991,; LDM_E 8 309 NUF2

oar_circ_0001742 chr1 113098403 113113615 + 15212 14691 ENSOARG00000011189; intron_1:113098538-113099991,intron_2:113100066-113101001,intron_3:113101078-113101573,intron_4:113101635-113112281,intron_5:113112379-113113541,; LDM_E 5 309 NUF2

oar_circ_0001743 chr1 115820986 115833747 - 12761 11529 ENSOARG00000011512; intron_2:115823317-115826062,intron_1:115821462-115823204,intron_3:115826204-115829738,exon_8:115830107-115830296,intron_5:115830296-115833615,; LDM_A 2 126 ALDH9A1

oar_circ_0001744 chr1 117042964 117044455 - 1491 1417 ENSOARG00000011878; exon_1:117044122-117044501,intron_9:117043084-117044122,; LDM_E 5 22 ILDR2

oar_circ_0001745 chr1 117315548 117319734 + 4186 4186 ENSOARG00000011991; intron_4:117315780-117319516,exon_4:117315548-117315780,exon_5:117319516-117319734,; LDM_A 2 244 DUSP27

oar_circ_0001746 chr1 117906008 117906753 + 745 745 ENSOARG00000012185; intron_1:117882218-117931170,; LDM_A 2 9 RCSD1

oar_circ_0001747 chr1 118032420 118036240 + 3820 3584 ENSOARG00000012200; exon_3:118032420-118032634,intron_3:118032634-118033569,intron_4:118033702-118036137,; LDM_E 3 906 MPZL1

oar_circ_0001748 chr1 118271904 118285448 + 13544 13451 ENSOARG00000012425; exon_4:118285262-118285448,intron_3:118271997-118285262,; LDM_A 2 777 DCAF6

oar_circ_0001749 chr1 118305520 118320724 + 15204 14740 ENSOARG00000012425; intron_7:118310489-118317444,exon_10:118320463-118320724,intron_6:118308217-118310274,intron_5:118305634-118308081,intron_9:118318933-118320463,intron_8:118317538-118318813,exon_7:118310274-118310489,; LDM_A 4 1613 DCAF6

oar_circ_0001750 chr1 118317444 118320724 + 3280 3066 ENSOARG00000012425; intron_9:118318933-118320463,intron_8:118317538-118318813,exon_10:118320463-118320724,; LDM_A 2 537 DCAF6

oar_circ_0001751 chr1 118317444 118336301 + 18857 18857 ENSOARG00000012425; intron_9:118318933-118320463,exon_11:118332072-118332243,intron_8:118317538-118318813,exon_10:118320463-118320724,intron_11:118332243-118339871,intron_10:118320724-118332072,; LDM_E,LDM_A 4,3 2141,2493 DCAF6

oar_circ_0001752 chr1 118332072 118336301 + 4229 4229 ENSOARG00000012425; exon_11:118332072-118332243,intron_11:118332243-118339871,; LDM_A 4 632 DCAF6

oar_circ_0001753 chr1 118332072 118339931 + 7859 7799 ENSOARG00000012425; intron_11:118332243-118339871,exon_11:118332072-118332243,; LDM_A 6 1455 DCAF6

oar_circ_0001754 chr1 118332072 118360480 + 28408 28030 ENSOARG00000012425; intron_14:118358526-118359845,intron_15:118359929-118360152,intron_11:118332243-118339871,intron_12:118339931-118353677,intron_13:118353795-118358410,exon_11:118332072-118332243,exon_16:118360152-118360480,; LDM_A 4 6336 DCAF6

oar_circ_0001755 chr1 118353677 118360480 + 6803 6485 ENSOARG00000012425; intron_14:118358526-118359845,exon_16:118360152-118360480,intron_15:118359929-118360152,intron_13:118353795-118358410,; LDM_E 3 957 DCAF6

oar_circ_0001756 chr1 118380001 118382901 + 2900 2672 ENSOARG00000012425; exon_19:118382021-118382178,intron_19:118382178-118382812,intron_18:118380140-118382021,; LDM_A 2 394 DCAF6

oar_circ_0001757 chr1 118380001 118391396 + 11395 11061 ENSOARG00000012425; exon_19:118382021-118382178,intron_19:118382178-118382812,intron_18:118380140-118382021,intron_20:118382901-118391290,; LDM_E,LDM_A 3,4 1076,1341 DCAF6

oar_circ_0001758 chr1 118382021 118382901 + 880 791 ENSOARG00000012425; exon_19:118382021-118382178,intron_19:118382178-118382812,; LDM_A 9 124 DCAF6

oar_circ_0001759 chr1 118382021 118391396 + 9375 9180 ENSOARG00000012425; intron_20:118382901-118391290,exon_19:118382021-118382178,intron_19:118382178-118382812,; LDM_E,LDM_A 3,6 865,1071 DCAF6

oar_circ_0001760 chr1 118421706 118432966 - 11260 11260 ENSOARG00000012502; exon_2:118421706-118422431,intron_4:118422431-118432548,exon_1:118432548-118432966,; LDM_E 3 479 GPR161

oar_circ_0001761 chr1 118519715 118532433 + 12718 12491 ENSOARG00000012519; intron_2:118520801-118532301,exon_1:118519710-118519895,intron_1:118519895-118520701,; LDM_A 4 62 TIPRL

oar_circ_0001762 chr1 119324972 119331003 - 6031 177 ENSOARG00000007650; exon_1:119324985-119325162,; LDM_E 2 16 SMIM11

oar_circ_0001763 chr1 120017383 120033887 - 16504 15988 ENSOARG00000012903; intron_33:120028367-120031874,intron_34:120031971-120033707,intron_30:120017499-120019063,intron_31:120019201-120021867,intron_32:120021931-120028266,exon_5:120033707-120033887,; LDM_A 2 174 ITSN1

oar_circ_0001764 chr1 120043465 120057810 - 14345 14188 ENSOARG00000012903; intron_37:120056739-120057717,intron_36:120043626-120056675,exon_4:120043465-120043626,; LDM_A 2 177 ITSN1

oar_circ_0001765 chr1 120043465 120060109 - 16644 16427 ENSOARG00000012903; intron_37:120056739-120057717,intron_36:120043626-120056675,intron_38:120057810-120060049,exon_4:120043465-120043626,; LDM_E 2 448 ITSN1

oar_circ_0001766 chr1 120185743 120194611 - 8868 8493 ENSOARG00000013038; intron_6:120186984-120194082,intron_5:120185860-120186873,intron_7:120194271-120194464,exon_6:120194082-120194271,; LDM_E 2 1267 SON

oar_circ_0001767 chr1 120185743 120196695 - 10952 10577 ENSOARG00000013038; intron_6:120186984-120194082,exon_4:120196534-120196695,intron_8:120194611-120196534,exon_6:120194082-120194271,intron_7:120194271-120194464,intron_5:120185860-120186873,; LDM_E 2 1720 SON

oar_circ_0001768 chr1 120194082 120194611 - 529 382 ENSOARG00000013038; intron_7:120194271-120194464,exon_6:120194082-120194271,; LDM_A 5 50 SON

oar_circ_0001769 chr1 120194082 120196695 - 2613 2466 ENSOARG00000013038; exon_6:120194082-120194271,intron_7:120194271-120194464,exon_4:120196534-120196695,intron_8:120194611-120196534,; LDM_E 2 529 SON

oar_circ_0001770 chr1 120212691 120215882 + 3191 3095 ENSOARG00000013139; exon_2:120212691-120212877,intron_3:120215232-120215707,exon_4:120215707-120215882,intron_2:120212877-120215136,; LDM_E 5 35 GART

oar_circ_0001771 chr1 120290671 120294239 + 3568 3247 ENSOARG00000013154; intron_2:120290811-120292819,intron_3:120292932-120294171,; LDM_E,LDM_A 2,7 20,12 TMEM50B

oar_circ_0001772 chr1 120431722 120436223 - 4501 4138 ENSOARG00000013188; exon_4:120433611-120433769,intron_6:120431837-120433363,intron_8:120433769-120436041,exon_3:120436041-120436223,; LDM_E 3 72 IFNAR1

oar_circ_0001773 chr1 120564345 120566072 - 1727 1572 ENSOARG00000013309; intron_3:120564514-120565917,exon_5:120564345-120564514,; LDM_A 2 9 IFNAR2

oar_circ_0001774 chr1 121130887 121133482 + 2595 2510 ENSOARG00000013414; intron_15:121131079-121133397,exon_15:121130887-121131079,; LDM_E,LDM_A 3,2 35,53 SYNJ1

oar_circ_0001775 chr1 121130887 121136666 + 5779 5553 ENSOARG00000013414; intron_15:121131079-121133397,exon_15:121130887-121131079,intron_16:121133482-121136525,; LDM_E,LDM_A 2,2 91,136 SYNJ1

oar_circ_0001776 chr1 121130887 121151949 + 21062 20185 ENSOARG00000013414; exon_19:121138138-121138297,exon_20:121139452-121139609,intron_26:121150644-121151805,intron_20:121139609-121140192,intron_22:121140625-121144105,intron_18:121137905-121138138,intron_15:121131079-121133397,exon_15:121130887-121131079,exon_24:121146526-121146689,exon_22:121140409-121140625,intron_19:121138297-121139452,intron_17:121136666-121137712,intron_24:121146689-121150433,intron_16:121133482-121136525,intron_23:121144184-121146526,exon_18:121137712-121137905,; LDM_E,LDM_A 6,9 361,376 SYNJ1

oar_circ_0001777 chr1 121139452 121140625 + 1173 956 ENSOARG00000013414; intron_20:121139609-121140192,exon_20:121139452-121139609,exon_22:121140409-121140625,; LDM_A 3 13 SYNJ1

oar_circ_0001778 chr1 121282528 121285972 - 3444 3444 ENSOARG00000013569; exon_1:121285775-121286324,intron_6:121282652-121285775,; LDM_A 2 11 EVA1C

oar_circ_0001779 chr1 121362954 121382001 + 19047 18342 ENSOARG00000013679; intron_3:121363106-121364009,intron_8:121377693-121381799,intron_4:121364128-121371040,intron_5:121371137-121373107,intron_7:121374876-121377568,exon_10:121381799-121382001,intron_6:121373193-121374750,; LDM_E 2 107 URB1

oar_circ_0001780 chr1 121803929 121810822 - 6893 6757 ENSOARG00000013875; exon_7:121803929-121804092,intron_5:121804092-121810686,; LDM_E 3 38 HUNK

oar_circ_0001781 chr1 122090838 122094591 - 3753 459 ENSOARG00000007880; exon_1:122094295-122094754,; LDM_A 2 197 -

oar_circ_0001782 chr1 122090883 122094636 - 3753 459 ENSOARG00000007880; exon_1:122094295-122094754,; LDM_A 3 276 -

oar_circ_0001783 chr1 122538790 122546305 + 7515 7388 ENSOARG00000014016; exon_4:122546132-122546305,exon_3:122538917-122539221,intron_2:122539221-122546132,; LDM_E 2 105 TIAM1

oar_circ_0001784 chr1 122636941 122639004 + 2063 1868 ENSOARG00000014016; intron_15:122638599-122638878,exon_15:122636941-122637121,intron_14:122637121-122638530,; LDM_E 8 25 TIAM1

oar_circ_0001785 chr1 122636941 122644860 + 7919 7615 ENSOARG00000014016; intron_16:122639004-122644751,intron_15:122638599-122638878,intron_14:122637121-122638530,exon_15:122636941-122637121,; LDM_E 2 170 TIAM1

oar_circ_0001786 chr1 122644751 122654915 + 10164 9731 ENSOARG00000014016; exon_19:122649442-122649622,intron_19:122649837-122654806,intron_17:122644860-122649442,; LDM_E 2 222 TIAM1

oar_circ_0001787 chr1 12442007 12445294 - 3287 3070 ENSOARG00000019926; intron_15:12443972-12445126,exon_12:12445126-12445294,intron_14:12442122-12443870,; LDM_A 2 8 INPP5B

oar_circ_0001788 chr1 12515554 12516177 - 623 349 ENSOARG00000019952; exon_2:12515554-12515729,exon_1:12516003-12516177,; LDM_A 2 61 FHL3

oar_circ_0001789 chr1 127493460 127495729 - 2269 2269 -- -- LDM_A 4 0 --

oar_circ_0001790 chr1 127606620 127619220 + 12600 12299 ENSOARG00000014877; intron_3:127606753-127619052,; LDM_E,LDM_A 6,6 287,99 ADAMTS5

oar_circ_0001791 chr1 128542344 128545046 + 2702 2589 ENSOARG00000015056; intron_4:128542457-128544852,exon_5:128544852-128545046,; LDM_E,LDM_A 4,5 404,129 APP

oar_circ_0001792 chr1 128602496 128628562 + 26066 25800 ENSOARG00000015056; exon_11:128628403-128628562,exon_7:128602496-128602664,intron_9:128618654-128626936,intron_8:128605403-128618520,intron_10:128627011-128628403,intron_7:128602664-128605346,; LDM_E 8 2454 APP

oar_circ_0001793 chr1 128605346 128628562 + 23216 22950 ENSOARG00000015056; intron_8:128605403-128618520,intron_10:128627011-128628403,exon_11:128628403-128628562,intron_9:128618654-128626936,; LDM_E 4 2058 APP

oar_circ_0001794 chr1 128618520 128628562 + 10042 9833 ENSOARG00000015056; exon_11:128628403-128628562,intron_9:128618654-128626936,intron_10:128627011-128628403,; LDM_E,LDM_A 4,3 264,46 APP

oar_circ_0001795 chr1 128701342 128711456 + 10114 9812 ENSOARG00000015056; intron_15:128701396-128707869,intron_16:128707970-128711309,; LDM_A 2 204 APP

oar_circ_0001796 chr1 128849521 128867040 - 17519 16991 ENSOARG00000015173; intron_4:128856545-128858690,exon_5:128854376-128854571,intron_2:128850507-128854376,intron_3:128854571-128856299,intron_1:128849662-128850453,intron_6:128863151-128866937,exon_4:128856299-128856545,intron_5:128858775-128863006,; LDM_A 2 390 GABPA

oar_circ_0001797 chr1 128902053 128907678 - 5625 5417 ENSOARG00000015207; intron_4:128902161-128904877,exon_7:128907475-128907678,intron_5:128904977-128907475,; LDM_E,LDM_A 8,3 267,50 JAM2

oar_circ_0001798 chr1 128902053 128913904 - 11851 11535 ENSOARG00000015207; intron_6:128907678-128912692,intron_4:128902161-128904877,exon_6:128912692-128912845,exon_7:128907475-128907678,intron_5:128904977-128907475,intron_7:128912845-128913796,; LDM_E,LDM_A 58,14 539,94 JAM2

oar_circ_0001799 chr1 128912692 128921761 - 9069 8895 ENSOARG00000015207; intron_7:128912845-128913796,exon_6:128912692-128912845,intron_8:128913904-128921695,; LDM_E 8 284 JAM2

oar_circ_0001800 chr1 137110715 137122280 - 11565 11023 ENSOARG00000015708; intron_3:137116296-137121668,exon_3:137121668-137121826,intron_1:137110923-137113993,intron_2:137114096-137116209,exon_2:137121970-137122280,; LDM_E 2 849 CHODL

oar_circ_0001801 chr1 138911031 138911184 + 153 153 -- -- LDM_A 2 0 --

oar_circ_0001802 chr1 139565342 139580855 - 15513 15230 ENSOARG00000016027; intron_3:139569360-139573476,exon_24:139565342-139565538,intron_5:139578886-139580676,exon_20:139580676-139580855,intron_2:139565538-139569236,intron_4:139573556-139578807,; LDM_A 2 1023 USP25

oar_circ_0001803 chr1 139573476 139580855 - 7379 7220 ENSOARG00000016027; intron_4:139573556-139578807,exon_20:139580676-139580855,intron_5:139578886-139580676,; LDM_A 13 488 USP25

oar_circ_0001804 chr1 139601952 139609821 - 7869 7869 ENSOARG00000016027; intron_6:139580855-139609681,; LDM_A 7 533 USP25

oar_circ_0001805 chr1 139618185 139633213 - 15028 14584 ENSOARG00000016027; exon_13:139623691-139623918,intron_13:139623918-139624535,exon_16:139618185-139618386,intron_10:139618386-139619824,exon_12:139624535-139624697,intron_15:139626090-139626421,intron_11:139620053-139620734,intron_16:139626549-139633064,intron_14:139624697-139625994,exon_15:139619824-139620053,intron_12:139620805-139623691,; LDM_A 2 965 USP25

oar_circ_0001806 chr1 139633064 139637907 - 4843 4620 ENSOARG00000016027; intron_17:139633213-139637833,; LDM_E,LDM_A 2,4 124,101 USP25

oar_circ_0001807 chr1 139633064 139646272 - 13208 12770 ENSOARG00000016027; intron_19:139643649-139646134,intron_17:139633213-139637833,intron_18:139637907-139643572,; LDM_A 4 574 USP25

oar_circ_0001808 chr1 139633064 139665150 - 32086 31561 ENSOARG00000016027; intron_21:139656657-139664987,intron_17:139633213-139637833,intron_18:139637907-139643572,intron_19:139643649-139646134,exon_4:139664987-139665150,intron_20:139646272-139656570,; LDM_E 5 1531 USP25

oar_circ_0001809 chr1 139633064 139680510 - 47446 46797 ENSOARG00000016027; intron_20:139646272-139656570,intron_22:139665150-139680386,intron_19:139643649-139646134,intron_21:139656657-139664987,intron_17:139633213-139637833,exon_4:139664987-139665150,intron_18:139637907-139643572,; LDM_E,LDM_A 20,6 2537,3057 USP25

oar_circ_0001810 chr1 139680386 139699574 - 19188 18841 ENSOARG00000016027; intron_23:139680510-139695352,intron_24:139695497-139699496,; LDM_E,LDM_A 3,2 765,905 USP25

oar_circ_0001811 chr1 139695352 139699574 - 4222 3999 ENSOARG00000016027; intron_24:139695497-139699496,; LDM_A 3 181 USP25

oar_circ_0001812 chr1 140524252 140551407 + 27155 27155 -- -- LDM_A 9 0 --

oar_circ_0001813 chr1 140557171 140557327 - 156 156 -- -- LDM_E 2 0 --

oar_circ_0001814 chr1 14073816 14075145 - 1329 1199 ENSOARG00000020090; exon_2:14074977-14075145,intron_4:14073946-14074977,; LDM_A 2 17 NT5C1A

oar_circ_0001815 chr1 141138066 141148404 - 10338 10338 -- -- LDM_E 3 0 --

oar_circ_0001816 chr1 14248890 14252957 - 4067 3678 ENSOARG00000020140; intron_8:14249036-14249759,intron_9:14249858-14252813,; LDM_E,LDM_A 9,3 138,67 TRIT1

oar_circ_0001817 chr1 143316576 143319826 + 3250 2971 ENSOARG00000016559; intron_2:143316734-143319705,; LDM_E 2 8 ROBO2

oar_circ_0001818 chr1 14448403 14455020 - 6617 5790 ENSOARG00000020179; intron_5:14452497-14454006,intron_6:14454077-14454557,intron_4:14449428-14452394,intron_3:14448502-14449337,; LDM_E 3 21 PPT1

oar_circ_0001819 chr1 144725115 144728868 - 3753 3365 ENSOARG00000016739; intron_25:144728244-144728747,intron_24:144725243-144728105,; LDM_E 4 47 -

oar_circ_0001820 chr1 14545129 14548762 + 3633 3418 ENSOARG00000020187; intron_5:14546274-14548629,exon_4:14545129-14545284,intron_4:14545284-14546192,; LDM_E 16 109 RLF

oar_circ_0001821 chr1 14545129 14556476 + 11347 11132 ENSOARG00000020187; exon_7:14556273-14556476,intron_6:14548762-14556273,intron_5:14546274-14548629,exon_4:14545129-14545284,intron_4:14545284-14546192,; LDM_E 14 377 RLF

oar_circ_0001822 chr1 146393 147479 - 1086 1086 ENSOARG00000017577; intron_1:23440-169733,; LDM_E 3 19 ING5

oar_circ_0001823 chr1 15038167 15051848 + 13681 13399 ENSOARG00000020294; exon_6:15048184-15048358,intron_4:15039718-15043070,intron_5:15043166-15048184,exon_7:15051689-15051848,intron_6:15048358-15051689,intron_3:15038239-15039604,; LDM_A 2 268 NFYC

oar_circ_0001824 chr1 15358665 15362832 - 4167 4040 ENSOARG00000020350; exon_9:15362599-15362832,intron_6:15358792-15362599,; LDM_E,LDM_A 10,47 73,41 SCMH1

oar_circ_0001825 chr1 15441208 15448343 - 7135 7064 ENSOARG00000020350; intron_10:15441443-15448272,exon_6:15441208-15441443,; LDM_E,LDM_A 2,3 206,80 SCMH1

oar_circ_0001826 chr1 15455756 15457414 - 1658 1658 ENSOARG00000020350; intron_12:15456913-15457345,intron_11:15448343-15456886,; LDM_A 3 29 SCMH1

oar_circ_0001827 chr1 154739500 154741584 + 2084 576 ENSOARG00000017138; exon_3:154739500-154740076,; LDM_E 2 202 C3orf38

oar_circ_0001828 chr1 155779284 155779945 + 661 661 ENSOARG00000017153; exon_3:155779284-155779945,; LDM_E 2 140 EPHA3

oar_circ_0001829 chr1 155924996 155926171 + 1175 1019 ENSOARG00000017153; intron_4:155925152-155925835,exon_5:155925835-155926171,; LDM_E 5 60 EPHA3

oar_circ_0001830 chr1 156009980 156022058 + 12078 12016 ENSOARG00000017153; exon_13:156021848-156022058,intron_11:156010166-156019923,exon_11:156009980-156010166,intron_12:156019985-156021848,; LDM_E 2 163 EPHA3

oar_circ_0001831 chr1 156816069 156826664 + 10595 10524 ENSOARG00000017337; intron_1:156816140-156826414,exon_2:156826414-156826664,; LDM_E,LDM_A 8,2 296,62 ARL13B

oar_circ_0001832 chr1 160632721 160642684 + 9963 9387 ENSOARG00000017529; intron_7:160640183-160642550,intron_6:160638995-160640141,intron_5:160637629-160638880,intron_4:160632869-160637492,; LDM_E 2 560 CRYBG3

oar_circ_0001833 chr1 161534722 161547044 + 12322 11887 ENSOARG00000017765; intron_4:161541457-161545904,intron_5:161546008-161546980,intron_2:161534811-161537004,intron_3:161537104-161541379,; LDM_A 6 410 ST3GAL6

oar_circ_0001834 chr1 161537004 161546008 + 9004 8722 ENSOARG00000017765; intron_3:161537104-161541379,intron_4:161541457-161545904,; LDM_A 2 310 ST3GAL6

oar_circ_0001835 chr1 161564715 161568457 + 3742 3398 ENSOARG00000017765; exon_8:161568022-161568209,intron_7:161564811-161568022,; LDM_A 2 116 ST3GAL6

oar_circ_0001836 chr1 161581990 161595436 - 13446 12792 ENSOARG00000017823; intron_6:161594468-161595285,intron_3:161582040-161585868,intron_4:161585962-161594109,; LDM_A 2 93 DCBLD2

oar_circ_0001837 chr1 161618861 161636922 - 18061 17923 ENSOARG00000017823; intron_14:161618999-161636694,exon_2:161636694-161636922,; LDM_E 3 1064 DCBLD2

oar_circ_0001838 chr1 162589732 162592508 - 2776 2776 ENSOARG00000017888; exon_4:162589732-162592508,; LDM_E,LDM_A 10,21 2210,2604 FILIP1L

oar_circ_0001839 chr1 163066802 163071083 + 4281 4128 ENSOARG00000017987; intron_11:163066973-163068902,intron_12:163068945-163070973,exon_11:163066802-163066973,; LDM_E,LDM_A 3,2 135,52 TBC1D23

oar_circ_0001840 chr1 163079653 163082264 + 2611 2386 ENSOARG00000017987; intron_16:163079742-163082128,; LDM_A 3 59 TBC1D23

oar_circ_0001841 chr1 163132366 163150885 - 18519 17552 ENSOARG00000018010; intron_4:163137914-163138781,intron_2:163132489-163133094,intron_9:163146506-163148129,exon_6:163142297-163142505,intron_6:163140084-163142297,intron_5:163138889-163139949,exon_2:163150711-163150885,intron_8:163145629-163146396,intron_10:163148256-163150711,intron_3:163133192-163137797,intron_7:163142505-163145480,; LDM_E 13 698 TOMM70A

oar_circ_0001842 chr1 163137797 163150885 - 13088 12342 ENSOARG00000018010; intron_4:163137914-163138781,intron_5:163138889-163139949,exon_2:163150711-163150885,intron_6:163140084-163142297,intron_8:163145629-163146396,intron_7:163142505-163145480,exon_6:163142297-163142505,intron_9:163146506-163148129,intron_10:163148256-163150711,; LDM_E,LDM_A 7,4 549,302 TOMM70A

oar_circ_0001843 chr1 163145480 163148256 - 2776 2390 ENSOARG00000018010; intron_8:163145629-163146396,intron_9:163146506-163148129,; LDM_E 5 71 TOMM70A

oar_circ_0001844 chr1 163145480 163150885 - 5405 5019 ENSOARG00000018010; exon_2:163150711-163150885,intron_8:163145629-163146396,intron_10:163148256-163150711,intron_9:163146506-163148129,; LDM_E,LDM_A 5,5 144,68 TOMM70A

oar_circ_0001845 chr1 163446789 163462882 + 16093 15947 ENSOARG00000018044; intron_2:163446981-163448249,exon_3:163448249-163448462,intron_3:163448462-163450278,exon_4:163450278-163450463,intron_4:163450463-163462736,exon_2:163446789-163446981,; LDM_E 2 345 -

oar_circ_0001846 chr1 164276487 164280782 - 4295 3990 ENSOARG00000018358; intron_15:164279594-164280442,exon_9:164280442-164280782,intron_14:164276634-164279436,; LDM_E,LDM_A 15,9 279,123 SENP7

oar_circ_0001847 chr1 164359398 164379256 - 19858 19858 ENSOARG00000018358; intron_22:164359451-164379373,; LDM_E 6 697 SENP7

oar_circ_0001848 chr1 164499289 164504250 + 4961 4722 ENSOARG00000018600; exon_6:164499953-164500120,intron_6:164500120-164503065,intron_7:164503230-164504125,exon_7:164503065-164503230,intron_5:164499403-164499953,; LDM_E 2 106 CEP97

oar_circ_0001849 chr1 164610656 164618024 + 7368 6171 ENSOARG00000018628; intron_1:164610796-164611286,intron_8:164614690-164615612,intron_4:164612415-164613226,intron_6:164613974-164614167,exon_8:164614520-164614690,intron_7:164614231-164614520,intron_5:164613332-164613827,exon_4:164611639-164612415,intron_10:164616019-164617876,exon_10:164615851-164616019,; LDM_E 4 188 NFKBIZ

oar_circ_0001850 chr1 16568154 16584135 - 15981 15981 ENSOARG00000020401; exon_2:16583810-16584153,intron_10:16571938-16583810,intron_9:16535468-16571863,; LDM_E 2 1241 FOXJ3

oar_circ_0001851 chr1 16571863 16584135 - 12272 12215 ENSOARG00000020401; intron_10:16571938-16583810,exon_2:16583810-16584153,; LDM_E,LDM_A 13,25 942,404 FOXJ3

oar_circ_0001852 chr1 16571863 16584247 - 12384 12215 ENSOARG00000020401; intron_10:16571938-16583810,exon_2:16583810-16584153,; LDM_E,LDM_A 2,5 949,406 FOXJ3

oar_circ_0001853 chr1 16813700 16817040 + 3340 3219 ENSOARG00000020435; exon_1:16813700-16813872,intron_1:16813872-16816919,; LDM_E 2 12 CCDC30

oar_circ_0001854 chr1 16813700 16833166 + 19466 19117 ENSOARG00000020435; intron_1:16813872-16816919,intron_2:16817040-16823874,intron_3:16824062-16831639,intron_4:16831729-16833028,exon_1:16813700-16813872,exon_3:16823874-16824062,; LDM_E 2 47 CCDC30

oar_circ_0001855 chr1 16813700 16850993 + 37293 36641 ENSOARG00000020435; exon_3:16823874-16824062,intron_2:16817040-16823874,intron_4:16831729-16833028,intron_6:16843366-16848457,intron_1:16813872-16816919,exon_1:16813700-16813872,intron_3:16824062-16831639,intron_5:16833182-16843294,intron_7:16848573-16850894,; LDM_A 4 65 CCDC30

oar_circ_0001856 chr1 16843294 16850993 + 7699 7412 ENSOARG00000020435; intron_7:16848573-16850894,intron_6:16843366-16848457,; LDM_A 2 10 CCDC30

oar_circ_0001857 chr1 168728036 168741499 - 13463 12712 ENSOARG00000018775; intron_5:168737598-168741352,intron_2:168728156-168734300,intron_3:168734441-168737255,; LDM_E 5 1363 CBLB

oar_circ_0001858 chr1 168787880 168806613 - 18733 18513 ENSOARG00000018775; intron_10:168788084-168803516,intron_11:168803648-168806525,exon_9:168787880-168788084,; LDM_E 2 1704 CBLB

oar_circ_0001859 chr1 168787880 168813460 - 25580 25100 ENSOARG00000018775; exon_9:168787880-168788084,intron_11:168803648-168806525,intron_12:168806613-168810231,intron_10:168788084-168803516,intron_13:168810369-168813338,; LDM_E 3 2498 CBLB

oar_circ_0001860 chr1 168803516 168813460 - 9944 9464 ENSOARG00000018775; intron_11:168803648-168806525,intron_12:168806613-168810231,intron_13:168810369-168813338,; LDM_E 5 1084 CBLB

oar_circ_0001861 chr1 168810231 168813460 - 3229 2969 ENSOARG00000018775; intron_13:168810369-168813338,; LDM_E 2 378 CBLB

oar_circ_0001862 chr1 168810231 168819679 - 9448 9188 ENSOARG00000018775; exon_4:168819522-168819679,intron_13:168810369-168813338,intron_14:168813460-168819522,; LDM_E 2 1549 CBLB

oar_circ_0001863 chr1 169943657 169957750 - 14093 14093 -- -- LDM_E 5 0 --

oar_circ_0001864 chr1 17013188 17013896 - 708 443 ENSOARG00000020454; intron_8:17013310-17013484,intron_9:17013537-17013806,; LDM_E 3 6 P3H1

oar_circ_0001865 chr1 171776269 171789146 - 12877 11638 ENSOARG00000018970; intron_12:171782874-171784290,intron_9:171778461-171779905,intron_7:171776549-171777217,exon_14:171777217-171777413,intron_13:171784509-171787306,exon_9:171784290-171784509,exon_10:171782708-171782874,intron_8:171777413-171778342,intron_10:171780005-171781328,intron_11:171781473-171782708,intron_14:171787388-171787754,intron_16:171788170-171789049,; LDM_E 2 632 KIAA1524

oar_circ_0001866 chr1 171777217 171780005 - 2788 2569 ENSOARG00000018970; intron_9:171778461-171779905,exon_14:171777217-171777413,intron_8:171777413-171778342,; LDM_E 2 107 KIAA1524

oar_circ_0001867 chr1 171810468 171825719 + 15251 14806 ENSOARG00000019010; intron_1:171810570-171815627,intron_3:171819789-171825602,intron_2:171815697-171819633,; LDM_E 2 176 DZIP3

oar_circ_0001868 chr1 171863909 171870213 + 6304 6149 ENSOARG00000019010; intron_15:171869365-171870168,exon_15:171869198-171869365,intron_12:171863986-171865632,exon_13:171865632-171866250,intron_14:171868162-171869198,intron_13:171866250-171868129,; LDM_E,LDM_A 4,2 756,145 DZIP3

oar_circ_0001869 chr1 171879045 171879201 + 156 156 ENSOARG00000019010; intron_16:171870213-171882970,; LDM_E 2 3 DZIP3

oar_circ_0001870 chr1 171897579 171904003 + 6424 6108 ENSOARG00000019010; intron_24:171897667-171899894,intron_25:171899996-171903877,; LDM_E 2 261 DZIP3

oar_circ_0001871 chr1 174332731 174355278 + 22547 22547 ENSOARG00000019175; intron_1:174313936-174354936,exon_2:174354936-174355278,; LDM_E 3 961 PVRL3

oar_circ_0001872 chr1 174354936 174355278 + 342 342 ENSOARG00000019175; exon_2:174354936-174355278,; LDM_E 6 145 PVRL3

oar_circ_0001873 chr1 174354936 174366756 + 11820 11702 ENSOARG00000019175; exon_3:174362836-174363133,exon_2:174354936-174355278,intron_2:174355278-174362836,intron_3:174363133-174366638,; LDM_A 4 217 PVRL3

oar_circ_0001874 chr1 174354936 174371022 + 16086 15816 ENSOARG00000019175; exon_3:174362836-174363133,exon_2:174354936-174355278,intron_3:174363133-174366638,intron_2:174355278-174362836,intron_4:174366756-174370870,; LDM_E 6 1466 PVRL3

oar_circ_0001875 chr1 175044259 175056244 + 11985 11703 ENSOARG00000019206; exon_3:175044259-175044640,intron_4:175055074-175056106,intron_3:175044640-175054930,; LDM_E,LDM_A 2,2 589,104 PHLDB2

oar_circ_0001876 chr1 175054930 175056244 + 1314 1032 ENSOARG00000019206; intron_4:175055074-175056106,; LDM_E,LDM_A 47,60 108,26 PHLDB2

oar_circ_0001877 chr1 175059474 175059863 - 389 389 -- -- LDM_A 2 0 --

oar_circ_0001878 chr1 175108607 175113257 + 4650 4564 ENSOARG00000019206; intron_16:175108827-175113171,exon_16:175108607-175108827,; LDM_E 2 157 PHLDB2

oar_circ_0001879 chr1 175127989 175128957 + 968 968 ENSOARG00000019221; intron_2:175124614-175128303,intron_3:175128415-175128819,; LDM_E,LDM_A 3,2 4,4 ABHD10

oar_circ_0001880 chr1 175935663 175950197 + 14534 14534 -- -- LDM_E 3 0 --

oar_circ_0001881 chr1 175935677 175950211 + 14534 14534 -- -- LDM_E 3 0 --

oar_circ_0001882 chr1 176345710 176346835 + 1125 1122 ENSOARG00000019381; intron_2:176345892-176346556,exon_3:176346556-176346835,exon_2:176345713-176345892,; LDM_E,LDM_A 4,2 50,9 BOC

oar_circ_0001883 chr1 176718959 176733538 - 14579 14189 ENSOARG00000019468; intron_3:176726106-176729142,intron_5:176730496-176733422,intron_2:176719056-176726023,intron_4:176729171-176730431,; LDM_A 2 219 KIAA2018

oar_circ_0001884 chr1 176771243 176772283 - 1040 716 ENSOARG00000019484; intron_2:176771310-176771614,intron_3:176771734-176772146,; LDM_E,LDM_A 2,16 27,27 NAA50

oar_circ_0001885 chr1 176841606 176842415 + 809 700 ENSOARG00000019488; intron_8:176841769-176842306,exon_8:176841606-176841769,; LDM_A 2 13 ATP6V1A

oar_circ_0001886 chr1 177514548 177515170 + 622 622 -- -- LDM_A 2 0 --

oar_circ_0001887 chr1 177833358 177835545 - 2187 2187 -- -- LDM_A 2 0 --

oar_circ_0001888 chr1 182334853 182349877 - 15024 15024 ENSOARG00000019700; exon_2:182345936-182346169,intron_3:182337610-182343467,exon_3:182343467-182343655,intron_3:182335666-182337487,exon_1:182349485-182349877,intron_5:182346169-182349485,intron_4:182343655-182345936,intron_1:182333573-182336853,; LDM_E 7 367 B4GALT4

oar_circ_0001889 chr1 182334867 182349877 - 15010 15010 ENSOARG00000019700; exon_1:182349485-182349877,intron_3:182335666-182337487,exon_3:182343467-182343655,intron_3:182337610-182343467,exon_2:182345936-182346169,intron_1:182333573-182336853,intron_4:182343655-182345936,intron_5:182346169-182349485,; LDM_E 2 367 B4GALT4

oar_circ_0001890 chr1 182731014 182731470 + 456 456 -- -- LDM_A 2 0 --

oar_circ_0001891 chr1 182789580 182802984 + 13404 10395 ENSOARG00000019790; intron_9:182801557-182802841,intron_6:182789762-182796332,intron_8:182799016-182801374,exon_9:182801374-182801557,; LDM_A 2 6 MAATS1

oar_circ_0001892 chr1 182958743 182972449 - 13706 13607 ENSOARG00000019835; intron_2:182958842-182972262,exon_10:182972262-182972449,; LDM_E 2 1193 GSK3B

oar_circ_0001893 chr1 183024427 183024620 + 193 193 -- -- LDM_E 2 0 --

oar_circ_0001894 chr1 183719831 183720292 + 461 461 ENSOARG00000019957; exon_1:183719831-183720292,; LDM_E,LDM_A 6,20 53,86 GTF2E1

oar_circ_0001895 chr1 184230532 184235767 - 5235 2840 ENSOARG00000020009; intron_1:184232651-184233163,intron_2:184233285-184235613,; LDM_E 2 15 POLQ

oar_circ_0001896 chr1 184385373 184385621 + 248 248 ENSOARG00000020029; exon_4:184384645-184386568,; LDM_A 2 150 FBXO40

oar_circ_0001897 chr1 184442130 184452660 - 10530 10049 ENSOARG00000020062; exon_14:184452481-184452666,intron_7:184442617-184452481,; LDM_E,LDM_A 6,5 122,36 GOLGB1

oar_circ_0001898 chr1 184485301 184498324 - 13023 12125 ENSOARG00000020062; intron_17:184492520-184495311,intron_13:184485420-184486469,intron_18:184495440-184496543,intron_15:184488689-184489844,intron_20:184497047-184498229,exon_2:184496894-184497047,exon_8:184486469-184486872,intron_14:184486872-184488575,intron_16:184489967-184492400,exon_3:184496543-184496696,; LDM_E 2 857 GOLGB1

oar_circ_0001899 chr1 184527097 184527266 + 169 169 -- -- LDM_E 2 0 --

oar_circ_0001900 chr1 184542948 184543768 - 820 600 ENSOARG00000020068; intron_6:184543058-184543658,; LDM_E 2 7 IQCB1

oar_circ_0001901 chr1 185062820 185071019 - 8199 8199 ENSOARG00000020125; intron_2:185066258-185070872,intron_1:185062611-185066198,; LDM_E,LDM_A 22,42 47,62 CCDC58

oar_circ_0001902 chr1 185162889 185168489 - 5600 5600 ENSOARG00000020139; intron_9:185158067-185166051,intron_10:185166161-185168389,; LDM_A 3 425 KPNA1

oar_circ_0001903 chr1 185166051 185178644 - 12593 12141 ENSOARG00000020139; intron_10:185166161-185168389,intron_11:185168489-185173444,intron_12:185173552-185178510,; LDM_E 2 474 KPNA1

oar_circ_0001904 chr1 185312166 185317675 + 5509 5375 ENSOARG00000020160; intron_14:185316084-185317541,exon_13:185312166-185312370,intron_13:185312370-185315484,exon_14:185315484-185316084,; LDM_A 2 47 PARP14

oar_circ_0001905 chr1 185448058 185463359 + 15301 14996 ENSOARG00000020167; intron_9:185458718-185463231,intron_8:185448167-185458650,; LDM_E,LDM_A 2,7 347,244 DIRC2

oar_circ_0001906 chr1 18703745 18742496 - 38751 38409 ENSOARG00000000823; intron_3:18703818-18731198,intron_5:18735675-18742379,intron_4:18731290-18735615,; LDM_E,LDM_A 9,5 680,264 ERI3

oar_circ_0001907 chr1 18731198 18742496 - 11298 11029 ENSOARG00000000823; intron_4:18731290-18735615,intron_5:18735675-18742379,; LDM_E,LDM_A 5,2 268,81 ERI3

oar_circ_0001908 chr1 18731198 18763110 - 31912 31643 ENSOARG00000000823; intron_4:18731290-18735615,intron_5:18735675-18742379,exon_3:18762832-18763110,intron_6:18742496-18762832,; LDM_E 10 1168 ERI3

oar_circ_0001909 chr1 187427284 187433602 + 6318 6203 ENSOARG00000020220; exon_2:187427284-187427438,intron_2:187427438-187429491,intron_3:187430163-187431539,exon_3:187429491-187430163,exon_4:187431539-187431715,intron_4:187431715-187433487,; LDM_E 6 215 UMPS

oar_circ_0001910 chr1 187477481 187483835 - 6354 6130 ENSOARG00000020222; intron_4:187479908-187483612,exon_12:187483612-187483835,intron_3:187477601-187479804,; LDM_E 2 63 ITGB5

oar_circ_0001911 chr1 18762832 18763110 - 278 278 ENSOARG00000000823; exon_3:18762832-18763110,; LDM_E 2 109 ERI3

oar_circ_0001912 chr1 187994265 188007916 - 13651 13317 ENSOARG00000020237; intron_4:187995709-188007790,intron_3:187994349-187995585,; LDM_E 2 1176 ZNF148

oar_circ_0001913 chr1 187994265 188034958 - 40693 40359 ENSOARG00000020237; intron_4:187995709-188007790,exon_3:188034609-188034958,intron_5:188007916-188034609,intron_3:187994349-187995585,; LDM_E 12 2936 ZNF148

oar_circ_0001914 chr1 188034609 188044591 - 9982 9845 ENSOARG00000020237; intron_6:188034958-188044454,exon_3:188034609-188034958,; LDM_E,LDM_A 6,5 1439,631 ZNF148

oar_circ_0001915 chr1 188034609 188056039 - 21430 21212 ENSOARG00000020237; intron_7:188044591-188055958,exon_3:188034609-188034958,intron_6:188034958-188044454,; LDM_E,LDM_A 9,7 1781,727 ZNF148

oar_circ_0001916 chr1 188163467 188178300 - 14833 14107 ENSOARG00000020240; intron_6:188174622-188178238,intron_5:188170326-188174556,intron_1:188163614-188165305,intron_4:188168439-188170236,intron_2:188165420-188166480,intron_3:188166626-188168339,; LDM_A 2 125 -

oar_circ_0001917 chr1 188263468 188294079 - 30611 30244 ENSOARG00000020246; intron_6:188273696-188280855,exon_9:188266627-188267126,exon_10:188263468-188263655,intron_5:188267126-188273555,exon_6:188283603-188283805,intron_10:188293091-188293903,intron_9:188291487-188293011,intron_8:188283805-188291310,intron_7:188281001-188283603,exon_5:188291310-188291487,intron_4:188263655-188266627,exon_3:188293903-188294079,; LDM_E 4 750 OSBPL11

oar_circ_0001918 chr1 188284555 188294079 - 9524 9524 ENSOARG00000020246; intron_9:188291487-188293011,exon_3:188293903-188294079,intron_10:188293091-188293903,exon_5:188291310-188291487,intron_8:188283805-188291310,; LDM_E 2 270 OSBPL11

oar_circ_0001919 chr1 188517556 188535534 - 17978 17407 ENSOARG00000020271; intron_5:188520062-188520855,exon_12:188534950-188535100,intron_8:188528835-188530358,intron_9:188530409-188534950,intron_3:188517694-188518252,intron_6:188520904-188528781,intron_4:188518371-188519954,intron_10:188535100-188535482,; LDM_E 7 1768 LRCH3

oar_circ_0001920 chr1 188534950 188561440 - 26490 25372 ENSOARG00000020271; intron_16:188554202-188555424,intron_13:188547726-188549232,exon_12:188534950-188535100,intron_17:188555561-188559002,intron_18:188559108-188559570,intron_14:188549353-188550562,intron_12:188542921-188547577,intron_15:188550656-188554092,intron_19:188559697-188561295,intron_10:188535534-188542844,intron_10:188535100-188535482,; LDM_E 5 2311 LRCH3

oar_circ_0001921 chr1 188547577 188549353 - 1776 1506 ENSOARG00000020271; intron_13:188547726-188549232,; LDM_A 2 57 LRCH3

oar_circ_0001922 chr1 188550562 188561440 - 10878 10159 ENSOARG00000020271; intron_18:188559108-188559570,intron_17:188555561-188559002,intron_15:188550656-188554092,intron_19:188559697-188561295,intron_16:188554202-188555424,; LDM_A 2 263 LRCH3

oar_circ_0001923 chr1 188554092 188561440 - 7348 6723 ENSOARG00000020271; intron_18:188559108-188559570,intron_19:188559697-188561295,intron_17:188555561-188559002,intron_16:188554202-188555424,; LDM_E 2 509 LRCH3

oar_circ_0001924 chr1 188622520 188637063 - 14543 14152 ENSOARG00000020275; intron_6:188622633-188628862,intron_7:188629011-188636934,; LDM_E 2 729 -

oar_circ_0001925 chr1 188922007 188922994 - 987 867 ENSOARG00000020307; intron_9:188922127-188922836,exon_9:188922836-188922994,; LDM_E,LDM_A 3,6 6,6 TFRC

oar_circ_0001926 chr1 189213358 189216890 - 3532 3316 ENSOARG00000020328; intron_9:189213426-189216742,; LDM_A 3 83 UBXN7

oar_circ_0001927 chr1 189271273 189271561 + 288 288 -- -- LDM_E 2 0 --

oar_circ_0001928 chr1 189489624 189489942 + 318 318 -- -- LDM_A 2 0 --

oar_circ_0001929 chr1 189565507 189565812 - 305 305 -- -- LDM_A 2 0 --

oar_circ_0001930 chr1 189565512 189565817 - 305 305 -- -- LDM_A 2 0 --

oar_circ_0001931 chr1 189660345 189661506 - 1161 659 ENSOARG00000020371; intron_2:189660553-189661212,; LDM_A 4 2 PIGZ

oar_circ_0001932 chr1 189821878 189839939 - 18061 17837 ENSOARG00000020377; intron_13:189834946-189839818,intron_12:189821981-189834789,exon_14:189834789-189834946,; LDM_E,LDM_A 13,3 937,205 DLG1

oar_circ_0001933 chr1 189834789 189839939 - 5150 5029 ENSOARG00000020377; exon_14:189834789-189834946,intron_13:189834946-189839818,; LDM_E,LDM_A 7,11 283,61 DLG1

oar_circ_0001934 chr1 190509719 190512662 + 2943 2884 ENSOARG00000020394; intron_5:190509778-190512478,exon_6:190512478-190512662,; LDM_E,LDM_A 5,25 88,30 ACAP2

oar_circ_0001935 chr1 190512478 190521280 + 8802 8661 ENSOARG00000020394; intron_6:190512662-190516354,exon_6:190512478-190512662,intron_7:190516399-190521184,; LDM_E 3 436 ACAP2

oar_circ_0001936 chr1 190521184 190543438 + 22254 21711 ENSOARG00000020394; intron_8:190521280-190528140,intron_12:190542738-190543332,intron_11:190541364-190542637,intron_10:190533949-190541312,intron_9:190528215-190533836,; LDM_E,LDM_A 7,3 1034,394 ACAP2

oar_circ_0001937 chr1 191363953 191367627 + 3674 3268 ENSOARG00000020416; exon_21:191366637-191366813,intron_21:191366813-191367346,intron_20:191364078-191366637,; LDM_E 3 89 ATP13A3
[truncated: 800,328 more chars]
